# Enantioselective Copper-Catalyzed Synthesis of Trifluoromethyl-Cyclopropylboronates

**DOI:** 10.1021/acs.orglett.1c02420

**Published:** 2021-07-28

**Authors:** Julia Altarejos, David Sucunza, Juan J. Vaquero, Javier Carreras

**Affiliations:** † Universidad de Alcalá, Departamento de Química Orgánica y Química Inorgánica,, Alcalá de Henares 28805, Spain; § Instituto de Investigación Química Andrés Manuel del Río (IQAR), Universidad de Alcalá, Alcalá de Henares 28805, Spain; ‡ Instituto Ramón y Cajal de Investigación Sanitaria (IRYCIS), Madrid 28034, Spain

## Abstract

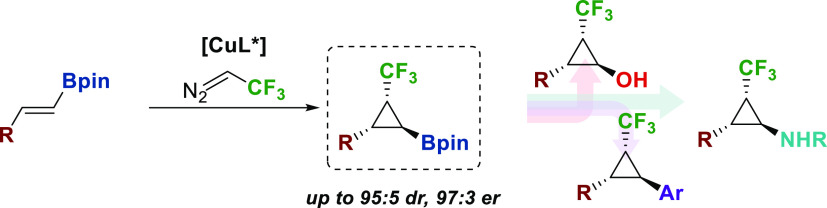

A copper-catalyzed enantioselective cyclopropanation involving trifluorodiazoethane in the presence of alkenyl boronates has been developed. This transformation enables the preparation of 2-substituted-3-(trifluoromethyl)cyclopropylboronates with high levels of stereocontrol. The products are valuable synthetic intermediates by transformation of the boronate group. This methodology can be applied to the synthesis of novel trifluoromethylated analogues of *trans*-2-arylcyclopropylamines, which are prevalent motifs in biologically active compounds.

Cyclopropanes are widespread carbocycles in bioactive natural and synthetic compounds.^1^ It is currently a standard fragment in drug discovery, which allows one to modulate properties such as lipophilicity, metabolic stability, p*K*_a_ or binding, among others.^2^ Nowadays, it is present in numerous drugs, for example *Ticagrelor*,^3^ which is active against cardiovascular diseases, or *Tezacaftor*,^4^ which is used to treat cystic fibrosis.

Numerous methods have been described for the synthesis of substituted cyclopropanes.^5^ Among all the different possibilities, the preparation of cyclopropanes with fluorinated groups, in particular trifluoromethyl, is of special interest.^6^ This functional group is present in a vast number of therapeutic compounds.^7^ However, the enantioselective procedures for the preparation of trifluoromethylcyclopropanes are scarce in the literature.^8^ All the existing protocols, which are summarized in Scheme 1a, led to cyclopropanes with an unsubstituted carbon on the three-membered ring. For this reason, there is still a need to develop efficient enantioselective methodologies to prepare all-carbon-substituted trifluoromethylcyclopropanes.

**Scheme 1:**
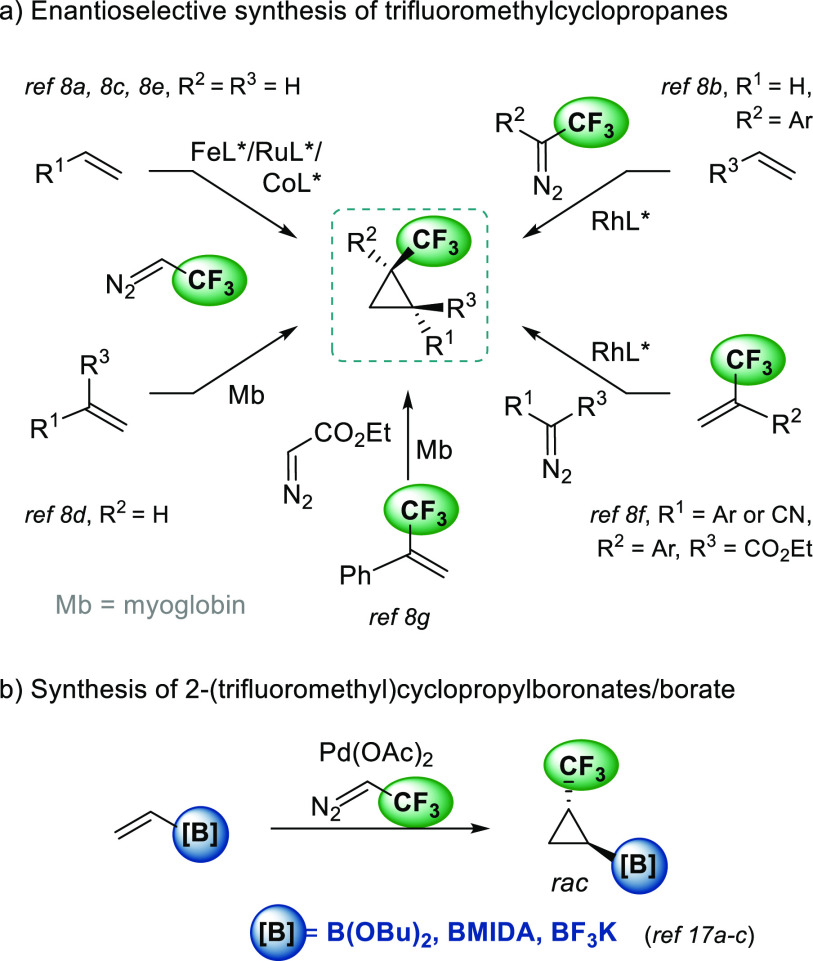
Previous Synthesis of Trifluoromethylcyclopropanes and Trifluoromethyl-Cyclopropylboronates

On the other hand, the synthesis of versatile cyclopropanes, such as cyclopropylboronates, has also attracted the interest of the synthetic community.^9^ A boronate group can be easily transformed into a wide range of different functional groups.^10^ This allows the generation of compound libraries from a common structure. In this area, several strategies have been recently developed to prepare optically active cyclopropylboronates, including cyclopropanation of alkenyl boronates with diazo compounds,^11^ borylative cyclization of allylic carbonates, phosphonates,^12^ or epoxides,^13^ hydroboration of cyclopropenes,^14^ zinco-cyclopropanation of allylic alcohols^15^ and C–H borylation.^16^

In this context, we focused our attention on the enantioselective preparation of cyclopropanes that include simultaneously a trifluoromethyl group and a pinacol boronate as substituents. These versatile compounds would give access to a wide range of trifluoromethyl–cyclopropane derivatives. In the literature, there are only three examples of these types of compounds, all of them have been obtained as racemates from monosubstituted vinyl boron derivatives (see Scheme 1b).^17^

Herein, we report the enantioselective cyclopropanation of *trans*-alkenyl boronates with trifluorodiazoethane catalyzed by a copper(I)-bisoxazoline complex to obtain versatile 2-substituted-3-(trifluoromethyl)cyclopropylboronates. It is worth mentioning that the reactivity between alkenyl boroxines and trifluorodiazoethane has been recently reported to prepare α-trifluoromethyl allylboronic acids,^18^ by formation of highly electrophilic BINOL boronate derivatives in a metal-free procedure.

The cyclopropanation was initially studied with (*E*)-styryl pinacolboronate (**1a**) as a model substrate. We commenced using Cu(I)-*t*BuBOX (5 mol %) as a catalyst formed *in situ* in DCE. Initial experiments showed that alkenyl boronate was not fully consumed with 2 equiv of diazo added over the course of 2 h (Table 1, entry 1). This point was crucial from a practical point of view, as cyclopropane **2a** was not easily separable from the starting material by column chromatography. Further increases in the amount of the diazo compound (4 equiv) combined with a longer reaction time (6 h) raised the conversion to 90% (Table 1, entries 2–4). The relative configuration of cyclopropane **2a** was determined by ^1^H NMR experiments (see the Supporting Information).

**Table 1:**
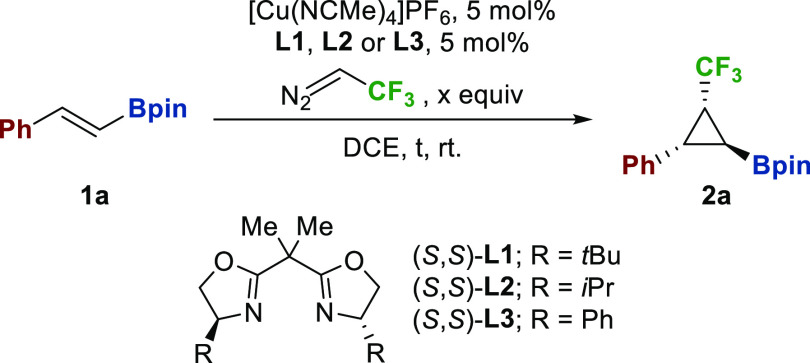
Optimization of the Reaction Conditions^a^

entry	ligand	diazo (equiv)	*t* (h)	conv (%)	dr	er
1	**L1**	2	2	72	92:8	–
2	**L1**	2	6	58	92:8	–
3	**L1**	4	2	89	92:8	–
4	**L1**	4	6	90	92:8	95:5
5	**L2**	4	6	72	79:21	88:12
6	**L3**	4	6	87	94:6	95:5
7	**L3**	4^b^	6	100	94:6	95:5
8	**L3**	2^b^	6	100 (69)^c^	94:6	95:5

a Reaction conditions: **1** (0.4 mmol), [Cu(NCMe)_4_]PF_6_ (0.02 mmol, 5 mol %), **L** (0.02 mmol, 5 mol %), DCE (1 mL), inert atmosphere, trifluorodiazoethane (0.5 M DCE, 2–4 equiv) 6 h slow addition. Conversion measured by ^1^H NMR. Diastereomeric ratio (dr) determined by ^19^F NMR analysis of the crude reaction mixture. Enantiomeric ratio (er) determined by HPLC analysis of the isolated product.

b Trifluorodiazoethane (1.06 M DCE).

c Isolated yield.

Gratifyingly, good results of diastereo- and enantiocontrol were obtained under these catalytic conditions (92:8 dr, 95:5 er). We examined different organic solvents such as THF or toluene (see SI). Toluene significantly reduced reactivity and diastereoselectivity, and THF led to no conversion of the olefin. Subsequently we investigated different com-mercially available BOX ligands. Whereas the iPrBOX (**L2**) ligand decreased the conversion and stereocontrol of the reaction, PhBOX (**L3**) slightly improved the diastereoselec-tivity (entries 5-6). At this stage, concentration of trifluorodiazoethane was increased from ca. 0.5 to 1 M, con-ducting to complete conversion (entry 7). Furthermore, the amount of diazo compound could be reduced to 2 equiva-lents (entry 8).

Under the optimized conditions, using 5 mol % of [Cu(NCMe)_4_]PF_6_ and *t*BuBOX as the catalyst and 2 equiv of trifluorodiazoethane added during 6 h, 69% of cyclopropylboronate **2a** was isolated, with high level of stereocontrol (94:6 dr, 95:5 er).

With the optimized conditions in hand, the scope of the cyclopropanation was examined (Scheme 2). The procedure was successful with a variety of (*E*)-alkenyl boronates, considering electron-withdrawing and electron-donating groups (alkyl, halogens, trifluoromethyl, ether and ester substituents) at different positions in the aromatic substituent of the olefin. Moderate to good yields were obtained for the entire series (40%–77%) and high stereoselectivity was also achieved, in terms of diastereoselectivity (up to 95:5) and enantioselectivity (up to 97:3). Notably, both parameters increase as the electron density of the aromatic ring decreases. A similar result was obtained with an electron-rich heterocycle such as thiophene (**2l**), with moderate enantioselectivity (90:10 er). Furthermore, an aliphatic-substituted cyclopropane (**2m**) was also accessible with moderate yield and levels of enantioinduction. In several substrates, an increase of the equivalents of trifluorodiazoethane was necessary to achieve complete conversion, whereas the reaction was suppressed in the presence of functional groups such as nitrile or nitro. The absolute configuration of the stereogenic centers of the cyclopropane were determined by single-crystal X-ray diffraction (XRD) analysis of *p*-bromo and *p*-methoxy derivatives **2i** and **2l** (Scheme 2).^19^

**Scheme 2:**
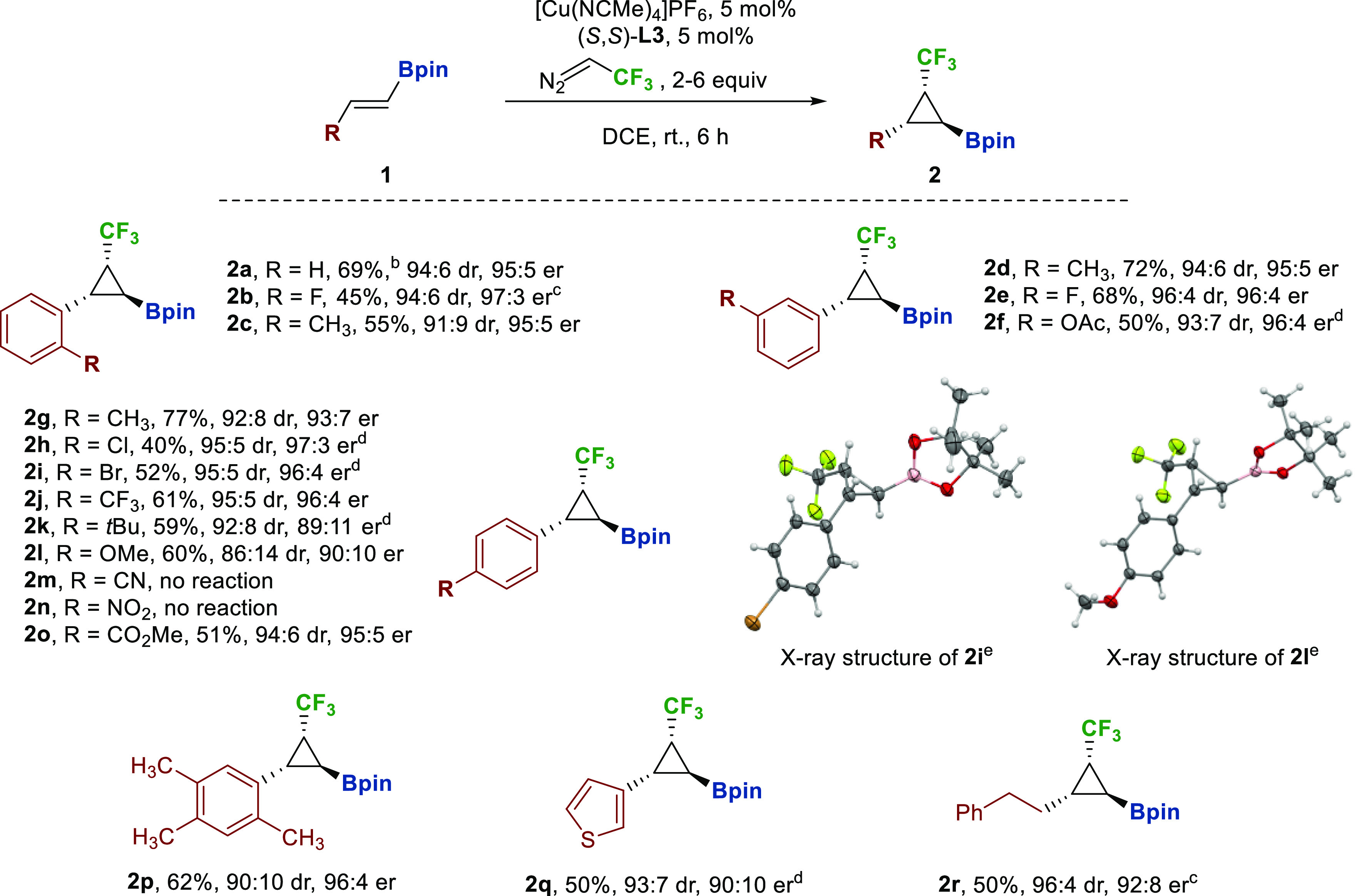
Substrate Scope of Copper-Catalyzed Cyclopropanation of Alkenyl Boronates Reaction conditions: **1** (0.61 mmol), [Cu(NCMe)_4_]PF_6_ (0.03 mmol, 5 mol %), (*S,S*)-**L3** (0.03 mmol, 5 mol %), DCE (1.5 mL), inert atmosphere trifluorodiazoethane in DCE (2 equiv), 6 h slow addition. Isolated yields. 76% at 1.25 mmol scale. Trifluorodiazoethane (6 equiv). Trifluorodiazoethane (4 equiv). Thermal ellipsoids are drawn at the 50% probability level.

As mentioned above, cyclopropylboronates are versatile intermediates in organic synthesis by the transformation of the C–B bond. To highlight the synthetic utility of the new compounds, we performed several transformations of the pinacol boronate group, following reported methodologies (Scheme 3). Boronic acid **3** was smoothly obtained by treatment with methylboronic acid.^20^ Standard conditions of Suzuki–Miyaura cross-coupling led to 3-trifluoromethyl-1,2-diarylsubstituted cyclopropane **4** in good yield. Furthermore, oxidation of the boronate group could be achieved under basic conditions to get alcohol **5**.^10^ Finally, amination of the cyclopropylboronate was accomplished by using BCl_3_ and BnN_3_ to get the benzylamine derivative in good yield (**6**).^21^ The latter transformations gave access to substituted *trans*-2-trifluoromethylcyclopropan-1-amine and *trans*-2-trifluoromethylcyclopropanol, rarely described in the literature in an enantioselective manner.^22^

**Scheme 3:**
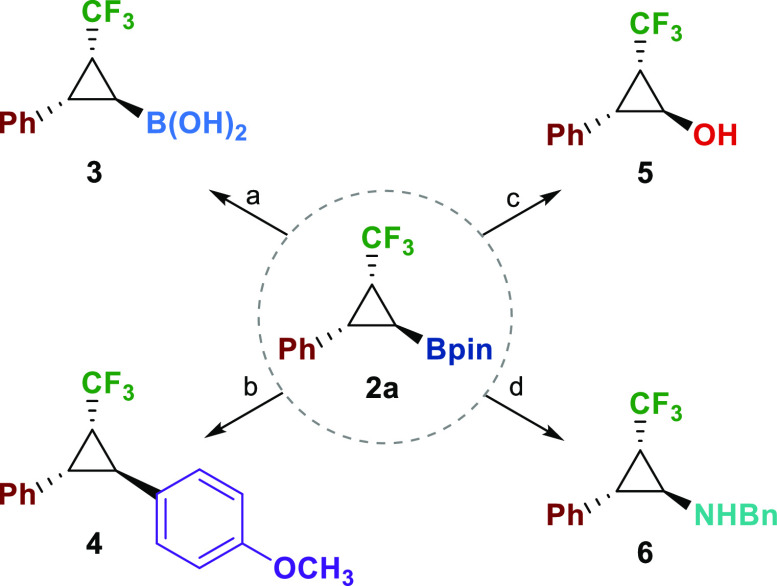
Transformations of Cyclopropylboronate Ester Reaction conditions: (a) MeB(OH)_2_ (5 equiv), TFA (5%)/DCM, 8 h, 72%. (b) 4-iodoanisole (1.5 equiv), Pd_2_(dba)_3_·CHCl_3_ (10 mol %), PPh_3_ (1 equiv), Ag_2_O (1.5 equiv), THF, 70 °C, 24 h, 45%. (c) 3 M NaOH 30% H_2_O_2_ THF, 30 min, 68%. (d) BCl_3_ (5.0 equiv, CH_2_Cl_2_, 25 °C, 1.5 h), then BnN_3_ (3.0 equiv, CH_2_Cl_2_, from 0 to 25 °C, 2 h), 51%.

Then, we focused our interest in amine derivative **6**, as a trifluoromethylated analogue of *trans*-2-arylcyclopropylamines. This scaffold is common to numerous biological active compounds^23^ and is present in drugs such as *Tranylcypromine* (an antidepressant), *Ticagrelor* (a platelet aggregation inhibitor), or candidates under clinical trials for the treatment of cancer and neurodegenerative diseases.^23,24^ Because of the implication of F atoms in the properties of bioactive compounds,^25^ we targeted the enantioselective synthesis of a CF_3_ analogue of a lysine-specific demethylase 1 (LSD1) inhibitor (Scheme 4). The amination of cyclopropylboronate **2a** with 3-(azidomethyl)-2-methoxypyridine (**7**) allowed us to obtain the trifluoromethyl analogue **8** of LSD1 inhibitor in a good yield.

**Scheme 4:**
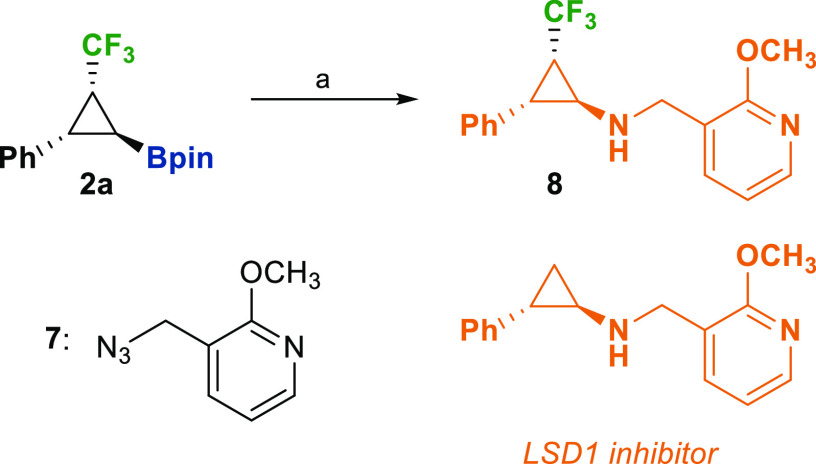
Preparation of a Trifluoromethyl Analogue of LSD1 Inhibitor Reaction conditions: (a) BCl_3_ (5.0 equiv, CH_2_Cl_2_, 25 °C, 1.5 h), then **7** (3.0 equiv, CH_2_Cl_2_, from 0 to 25 °C, 4 h), 55%.

In summary, we have developed a catalytic approach for the preparation of enantiomerically enriched 2-substituted-3-(trifluoromethyl)cyclopropylboronates by cyclopropanation of (*E*)-alkenyl boronates with trifluorodiazoethane. This methodology is general for a variety of substrates, using commercially available copper catalyst and ligand. Valuable synthetic intermediates can be obtained by the functionalization of the C–B bond. This route provides straightforward access to enantioenriched 2-aryl-3-(trifluoromethyl)cyclopropylamines, a relevant scaffold in medicinal chemistry.

## Supporting Information Available

Experimental procedures; characterization data; ^1^H, ^13^C, ^11^B and ^19^F NMR spectral data; HPLC; mass spectrometry data of new compounds and X-ray crystallographic data for **2i** and **2l** (PDF)

## Accession Codes

CCDC 2079480 and 2079481 contain the supplementary crystallographic data for this paper. These data can be obtained free of charge via www.ccdc.cam.ac.uk/data_request/cif, or by emailing data_request@ccdc.cam.ac.uk, or by contacting The Cambridge Crystallographic Data Centre, 12 Union Road, Cambridge CB2 1EZ, UK; fax: + 44 1223 336033.
