# Supplementary material for: Enantioselective Copper-Catalyzed Synthesis of Trifluoromethyl-Cyclopropylboronates
Source: Org Lett. 2021 Jul 28;23(15):6174–8. doi: 10.1021/acs.orglett.1c02420 (PMC8895459; doi:10.1021/acs.orglett.1c02420)
Supplement: Supplementary file 1 — ol1c02420_si_001.pdf [file ol1c02420_si_001.pdf]

Supporting Information

Enantioselective copper-catalyzed synthesis of trifluoromethyl-cyclopropylboronates

Julia Altarejos, David Sucunza, Juan J. Vaquero, Javier Carreras*

Universidad de Alcalá. Departamento de Química Orgánica y Química Inorgánica, Instituto de Investigación Química Andrés Manuel del Río (IQAR), Campus Científico-Tecnológico, Facultad de Farmacia. 28805 Alcalá de Henares, Madrid, Spain; Instituto Ramón y Cajal de Investigación Sanitaria (IRYCIS), Madrid 28034, Spain.

E-mail Javier Carreras: javier.carreras@uah.es

Table of Contents

Experimental procedures S-3

Optimization details S-4

Characterization data S-5

1. Alkenyl boronates S-5

2. Borocyclopropanes S-6

3. Derivatization products S-14

NMR spectra S-17

HPLC analyses of borocyclopropanes S-64

X-ray crystallographic data S-81

Experimental procedures

All commercially available compounds were used as received unless stated otherwise. Bisoxazolines L1, L2 and L3, as well as $[Cu(NCMe)_4]PF_6$ were purchased from Merck (Sigma-Aldrich). Unless otherwise stated, reactions were carried out under nitrogen atmosphere with standard Schlenck techniques. Solvents were purchased from commercial sources or dried in a solvent purification system (MB SPS-800, MBRAUN). Analytical thin layer chromatography was carried out using TLC-aluminium sheets with 0.2 mm of silica gel (Merck 60 F254) and UV light as visualizing agent or phosphomolybdic acid solution as developing agent. Chromatography purifications were carried out using silica gel (40-63 µm, 60 Å).

NMR spectra were recorded at 298 K using either a Varian Mercury VX-300, Varian Unity 300, or Varian Unity 500 MHz spectrometer. Chemical shift values for ^{1}H and ^{13}C are reported as δ values (ppm) relative to the deuterated solvent ($CDCl_3$: 7.26 ppm, 77.16 ppm; CD_3OD: 3.31 ppm, 49.00 ppm) and coupling constants (*J*) in Hz. The following abbreviations are used in reporting NMR data: s, singlet; bs, broad singlet; d, doublet; t, triplet; q, quartet; dd, doublet of doublets; appt, apparent triplet; m, multiplet.

Optical rotations were obtained using a PerkinElmer 341 polarimeter and concentrations are given in g/100mL.

All melting points were determined in open capillary tubes using a Stuart Scientific SMP3 melting point apparatus.

High-resolution analysis (HRMS) were performed using an Agilent 6210 TOF LC/MS system.

HPLC data were recorded in Agilent 1200 including a diode array detector (DAD 61315D).

Single crystal structure determination was performed by Dr. Christopher Golz at the University of Göttingen using a Bruker D8 Venture four-circle-diffractometer equipped with an Oxford Cryosystems low-temperature device.

Optimization details

Additional experiments in the optimization of the cyclopropanation.

Table S1. Optimization of the cyclopropanation reaction using (*E*)-styryl pinacolboronate (**1a**).[a]

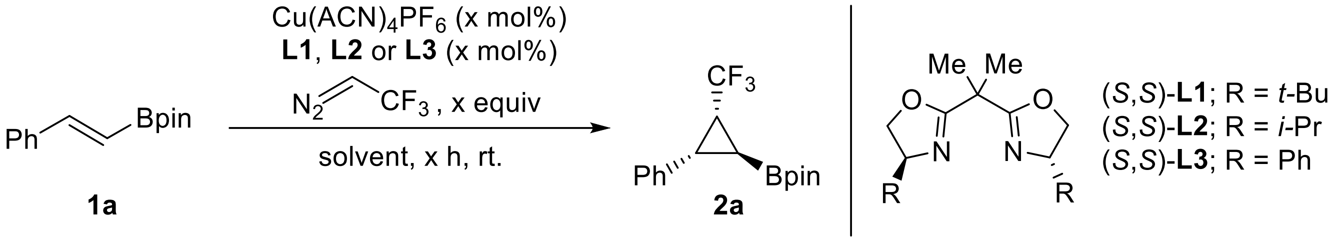

	ligand (mol%)	diazo equiv. (conc)	Solv	t [h]	Conv. [%][b]	d.r.[c]	e.r.[d]
**1**	**L1** (5)	2 (0.60M)	DCE	2	72	92:8	
**2**	**L1** (5)	2 (0.46M)	DCE	6	58	92:8	
**3**	**L1** (5)	2 (0.46M)	DCE	15	70	92:8	
**4**	**L1** (5)	4 (0.46M)	DCE	2	89	92:8	
**5**	**L1** (10)	4 (0.50M)	DCE	2	82	92:8	
**6**	**L1** (5)	4 (0.50M)	THF	2	0	-	
**7**	**L1** (5)	4 (0.50M)	Toluene	2	71	83:17	
**8**	**L1** (5)	4 (0.50M)	DCM	2	87	91:9	
**9**	**L1** (5)	4 (0.50M)	DCM	6	91	91:9	
**10**	**L1** (5)	4 (0.30M)	DCE	6	0	-	
**11**	**L1** (5)	4 (0.52M)	DCE	6	90	92:8	95:5
**12**	**L2** (5)	4 (0.52M)	DCE	6	72	79:21	88:12
**13**	**L3** (5)	4 (0.52M)	DCE	6	87	94:6	95:5
**14**	**L3** (5)	4 (1.06M)	DCE	6	100	94:6	95:5
**15**[e]	**L3** (5)	4 (1.06M)	DCE	6	100	95:5	94:6
**16**	**L3** (5)	2 (1.06M)	DCE	6	100	94:6	95:5

[a] Reaction conditions: alkenyl boronate (0.4 mmol), trifluoromethyldiazoethane (slow addition), catalytic system, solvent (1 mL). [b] Conversion determined by ^{1}H NMR [c] d.r. determined by ^{19}F NMR. [d] e.r. determined by HPLC. [e] Reaction performed at 0°C.

Characterization data

1. Alkenyl boronates

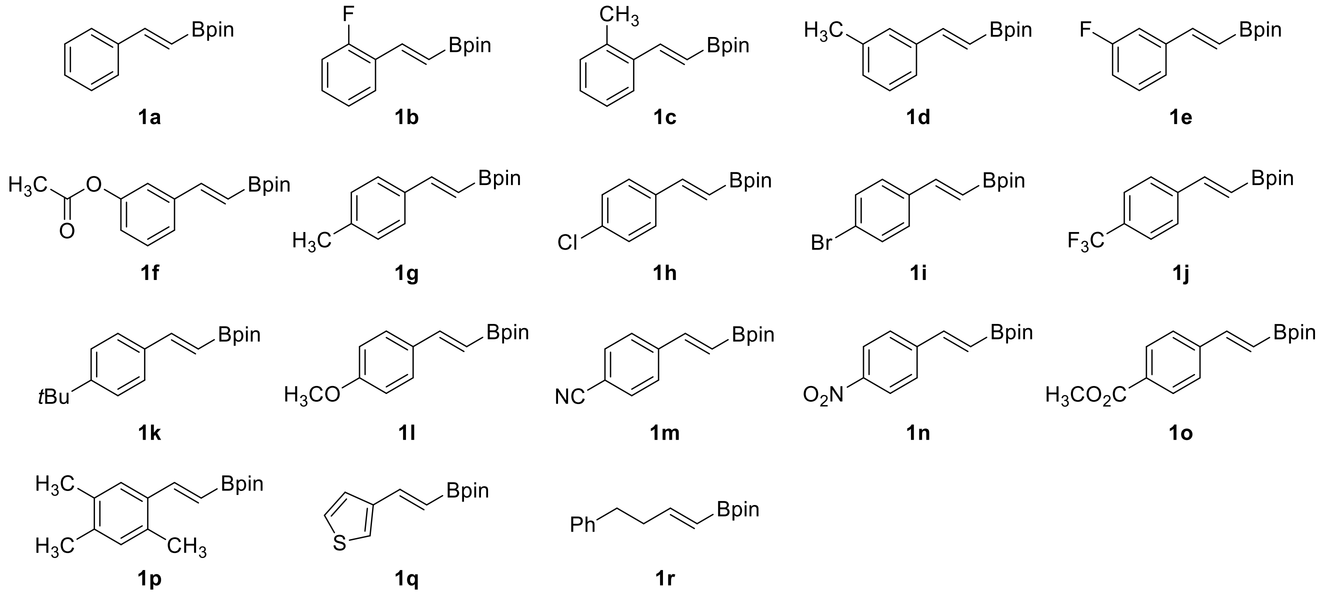

Scheme S1. Alkenyl boronates used on the cyclopropanation.

Alkenyl boronates were synthesized following reported procedures (**1a**-**1l**, **1o**, **1p**-**1r**[S1a], **1m**, [S1b] **1n**[S1c]) and NMR data were in accordance with those previously reported.[S1,S2]

Hydroboration, general procedure: [S1a]

The corresponding alkyne (1.0 equiv) and pinacolborane (2.0 equiv) were mixed in a microwave vial and heated at 215 °C for 18 minutes by microwave irradiation. The crude was purified by flash column chromatography (hexane/AcOEt) to obtain the (*E*)-alkenyl boronate.

(*E*)-4,4,5,5-tetramethyl-2-(2,4,5-trimethylstyryl)-1,3,2-dioxaborolane (1p)

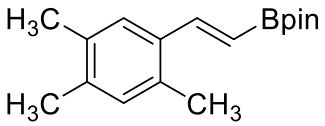

1p

Prepared from 1-ethynyl-2,4,5-trimethylbenzene (433 mg, 3 mmol) and pinacolborane (890 µL, 6 mmol) following the general procedure and purified by flash chromatography (hexane/EtOAc, 95/5). Yellow oil (789 mg, 96%).

^{1}H NMR (400 MHz, $CDCl_3$) δ 7.52 (d, *J* = 18.2 Hz, 1H), 7.25 (s, 1H), 6.82 (s, 1H), 5.95 (d, *J* = 18.5 Hz, 1H), 2.26 (s, 3H), 2.13 (s, 6H), 1.22 (s, 12H) ppm.

^{13}C NMR (101 MHz, $CDCl_3$) δ 147.3, 137.4, 134.2, 134.1, 133.8, 131.9, 127.1, 116.8 (bs), 83.3, 24.9, 19.5, 19.5, 19.2 ppm.

^{11}B NMR (160 MHz, $CDCl_3$) δ 30.1 ppm.

HRMS-ESI m/z calcd for $C_{17}H_{26}BO_2$ $[M+H]^+$ 273.2020, found 273.2024.

[S1] (a) J. Altarejos, D. Sucunza, J. J. Vaquero, J. Carreras, *Eur. J. Org. Chem.* **2020**, 3024–3029. (b) S. Chen, L. Yang, D. Yi, Q. Fu, Z. Zhang, W. Liang, Q. Zhang, J. Ji, W. Wei, *RSC Adv.* **2017**, *7*, 26070–26073. (c) J. J. Molloy, C. P. Seath, M. J. West, C. McLaughlin, N. J. Fazakerley, A. R. Kennedy, D. J. Nelson, A. J. B. Watson, *J. Am. Chem. Soc.* **2018**, *140*, 126–130.

[S2] (a) D. Yoshii, X. Jin, N. Mizuno, K. Yamaguchi, *ACS Catal.* **2019**, *9*, 3011–3016. (b) D. Zhu, S. Gan, R. L.-Y. Bao, L. Shi, *Org. Biomol. Chem.* **2020**, *18*, 5567–5570.

2. Borocyclopropanes

General procedure:

(*S*,*S*)-**L3** (0.05 equiv) and $Cu(NCMe)_4PF_6$ (0.05 equiv) were mixed in dichloroethane (0.5 mL) under inert atmosphere and stirred for 20 minutes. The corresponding alkenyl boronate (1 equiv) was added to the schlenk solved in dichloroethane (2 x 0.5 mL). Then, a solution of trifluorodiazoethane in dichloroethane (0.7-1.2 M, 2-6 equiv.) was added dropwise for 6 hours. At the end of the reaction, the volatiles were removed under vacuum and the crude was purified by flash column chromatography (hexane/EtOAc, 95:5) to obtain the corresponding trifluoromethylcyclopropylboronate.

For the synthesis of the racemic derivatives for HPLC analyses, an analogous procedure was followed using $Pd(OAc)_2$ (0.1 equiv) as catalyst. The diazo compound (4 equiv.) was added dropwise for 4 hours. The 1:1 diastereomeric mixtures of cyclopropanes were purified by flash column chromatography. The desired racemic cyclopropane (or a mixture in high dr) was used as HPLC standard.

2-((1*S*,2*S*,3*R*)-2-Phenyl-3-(trifluoromethyl)cyclopropyl)-4,4,5,5-tetramethyl-1,3,2-dioxoborolane (2a)

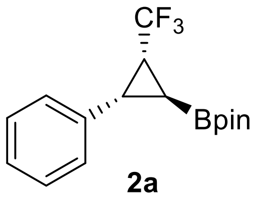

2a

Prepared from alkenyl boronate **1a** (150 mg, 0.61 mmol), trifluoromethyldiazoethane (1.73 mL (0.70 M), 1.22 mmol), $Cu(NCMe)_4PF_6$ (11.3 mg, 0.03 mmol) and (*S*,*S*)-**L3** (10.1 mg, 0.03 mmol) following the general procedure and purified by flash chromatography (hexane/AcOEt 95:5). Yellow oil (143 mg, 69%).

^{1}H NMR (500 MHz, $CDCl_3$) δ 7.33 – 7.28 (m, 4H), 7.28 – 7.21 (m, 1H), 2.60 (appt, *J* = 8.2 Hz, 1H), 2.09 – 1.99 (m, 1H), 1.27 (s, 6H), 1.27 (s, 6H), 1.04 (appt, *J* = 7.0 Hz, 1H) ppm.

^{13}C NMR (126 MHz, $CDCl_3$) δ 135.4, 129.4, 128.2, 127.0, 126.1 (q, *J* = 272.6 Hz), 84.1, 25.7 (q, *J* = 1.7 Hz), 25.5 (q, *J* = 35.5 Hz), 24.9, 24.8, 2.2 (bs) ppm.

^{19}F NMR (282 MHz, $CDCl_3$) δ -55.5 (d, *J* = 7.5 Hz) ppm.

^{11}B NMR (160 MHz, $CDCl_3$) δ 32.4 ppm.

HRMS-ESI m/z calcd for $C_{16}H_{20}BF_3O_2$ $[M+H]^+$ 313.1581, found 313.1576.

$[\alpha]_D^{25}$ = -57.9 (c = 0.93, $CHCl_3$).

Procedure for the synthesis of 2a on 1.25 mmol scale:

Prepared from alkenyl boronate **1a** (288 mg, 1.25 mmol), trifluoromethyldiazoethane (3.68 mL (0.70 M), 2.50 mmol), $Cu(NCMe)_4PF_6$ (23.3 mg, 0.06 mmol) and (*S*,*S*)-**L3** (20.9 mg, 0.06 mmol) following the general procedure and purified by flash chromatography (hexane/AcOEt 95:5). Yellow oil (297 mg, 76%).

2-((1*R*,2*R*,3*R*)- and (1*S*,2*S*,3*S*)-2-Phenyl-3-(trifluoromethyl)cyclopropyl)-4,4,5,5-tetramethyl-1,3,2-dioxoborolane ((±)-2a´)

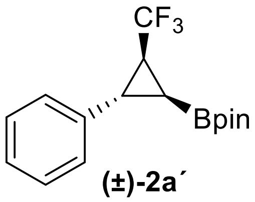

(±)-2a´

Purified by flash chromatography (hexane/Et_2O 95:5) from $Pd(OAc)_2$ catalyzed reaction to obtain **(±)-2a´** as a pale yellow solid compound. **M.p.** 55-56 °C.

^{1}H NMR (500 MHz, $CDCl_3$) δ 7.31 – 7.27 (m, 2H), 7.23 – 7.19 (m, 1H), 7.14 – 7.11 (m, 2H), 2.62 (dd, *J* = 7.8, 5.2 Hz, 1H), 2.07 – 1.97 (dqd, *J* = 10.6, 7.8, 5.2 Hz, 1H), 1.27 (s, 6H), 1.25 (s, 6H), 0.79 (dd, *J* = 10.6, 7.8 Hz, 1H).

^{13}C NMR (126 MHz, $CDCl_3$) δ 139.5, 128.5, 126.7, 126.2, 125.9 (q, *J* = 272.3 Hz), 83.9, 28.2 (q, *J* = 37.3 Hz), 24.8, 24.5, 23.7 (q, *J* = 2.7 Hz) ppm. (C-B signal not observed due to low intensity)

^{19}F NMR (282 MHz, $CDCl_3$) δ -57.4 (d, *J* = 7.0 Hz) ppm.

^{11}B NMR (160 MHz, $CDCl_3$) δ 31.7 ppm.

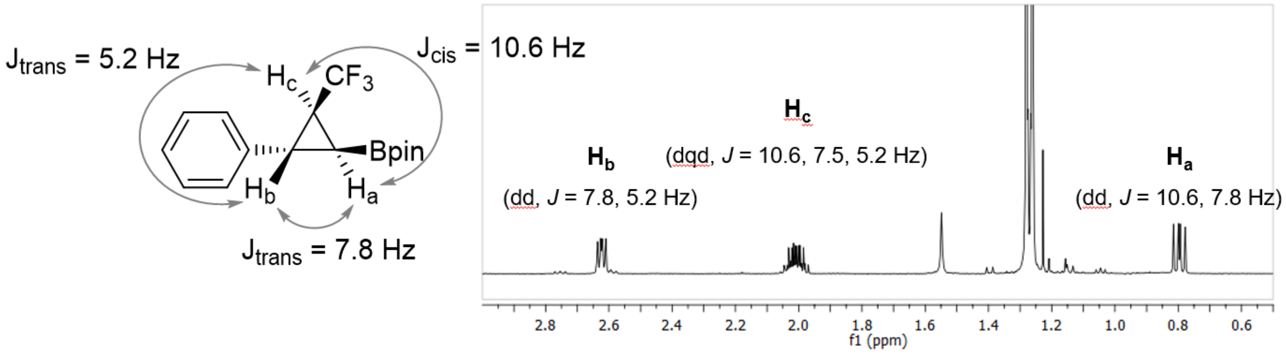

Figure S1. Coupling constants in the ^{1}H NMR of compound **(±)-2a´**.

2-((1*S*,2*S*,3*R*)-2-(2-Fluorophenyl)-3-(trifluoromethyl)cyclopropyl)-4,4,5,5-tetramethyl-1,3,2-dioxoborolane (2b)

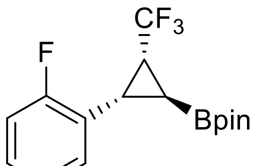

2b

Prepared from alkenylboronate **1b** (150 mg, 0.61 mmol), trifluoromethyldiazoethane (3.21 mL (1.13 M), 3.63 mmol), $Cu(NCMe)_4PF_6$ (11.3 mg, 0.03 mmol) and (*S*,*S*)-**L3** (10.1 mg, 0.03 mmol) following the general procedure and purified by flash chromatography. Yellow oil (89 mg, 45%).

^{1}H NMR (300 MHz, $CDCl_3$) δ 7.25 – 7.14 (m, 2H), 7.10 – 6.98 (m, 2H), 2.54 (appt, *J* = 8.2 Hz, 1H), 2.09 (m, 1H), 1.26 (s, 12H), 1.05 (appt, *J* = 7.1 Hz, 1H) ppm.

^{13}C NMR (75 MHz, $CDCl_3$) δ 162.6 (d, *J* = 247.1 Hz), 130.0 (bs),128.8 (d, *J* = 8.1 Hz), 125.8 (q, *J* = 272.6 Hz), 123.7 (d, *J* = 3.3 Hz), 122.8 (d, *J* = 15.2 Hz), 114.9 (d, *J* = 21.5 Hz), 84.2, 24.9 (q, *J* = 36.1 Hz), 24.8, 24.7, 19.9 (bs), 1.6 (bs) ppm.

^{19}F NMR (282 MHz, $CDCl_3$) δ -56.4 (d, *J* = 6.9 Hz, 3F), -110.2 (bs, 1F) ppm.

^{11}B NMR (160 MHz, $CDCl_3$) δ 32.2 ppm.

HRMS-ESI m/z calcd for $C_{16}H_{19}BF_4NaO_2$ $[M+Na]^+$ 353.1306, found 353.1333.

$[\alpha]_D^{25}$ = -33.7 (c = 1.14, $CHCl_3$).

2-((1*S*,2*S*,3*R*)-2-(*o*-Tolyl)-3-(trifluoromethyl)cyclopropyl)-4,4,5,5-tetramethyl-1,3,2-dioxoborolane (2c)

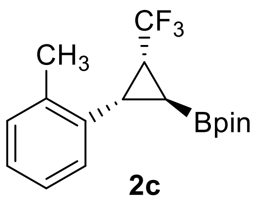

2c

Prepared from alkenyl boronate **1c** (148 mg, 0.61 mmol), trifluoromethyldiazoethane (3.56 mL (0.68 M), 1.22 mmol), $Cu(NCMe)_4PF_6$ (11.3 mg, 0.03 mmol) and (*S*,*S*)-**L3** (10.1 mg, 0.03 mmol) following the general procedure and purified by flash chromatography (hexane/AcOEt 95:5). Yellow oil (108 mg, 55%).

^{1}H NMR (300 MHz, $CDCl_3$) δ 7.19 – 7.10 (m, 4H), 2.49 – 2.43 (m, 1H), 2.41 (s, 3H), 2.16 – 2.07 (m, 1H), 1.27 (s, 6H), 1.27 (s, 6H), 1.11 (appt, *J* = 7.0 Hz, 1H) ppm.

^{13}C NMR (101 MHz, $CDCl_3$) δ 138.6, 133.7, 129.8, 128.3, 127.2, 126.1 (q, *J* = 272.4 Hz), 125.6, 84.1, 25.3 (q, *J* = 35.4 Hz), 24.9, 24.8, 19.5, 1.5 (bs) ppm.

^{19}F NMR (376 MHz, $CDCl_3$) δ -61.8 (d, *J* = 7.3 Hz, 3F) ppm.

^{11}B NMR (160 MHz, $CDCl_3$) δ 32.4 ppm.

HRMS-ESI m/z calcd for $C_{17}H_{23}BF_3O_2$ $[M+H]^+$ 327.1738, found 327.1742.

$[\alpha]_D^{25}$ = -39.4 (c = 1.03, $CHCl_3$).

2-((1*S*,2*S*,3*R*)-2-(*m*-Tolyl)-3-(trifluoromethyl)cyclopropyl)-4,4,5,5-tetramethyl-1,3,2-dioxoborolane (2d)

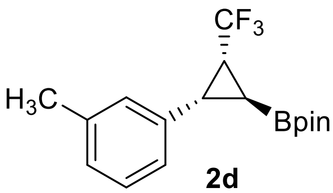

2d

Prepared from alkenylboronate **1d** (148 mg, 0.61 mmol), trifluoromethyldiazoethane (0.98 mL, 1.22 mmol), $Cu(NCMe)_4PF_6$ (11.3 mg, 0.03 mmol) and (*S*,*S*)-**L3** (10.1 mg, 0.03 mmol) following the general procedure and purified by flash chromatography. Yellow oil (142 mg, 72%).

^{1}H NMR (300 MHz, $CDCl_3$) δ 7.24 – 7.00 (m, 4H), 2.56 (appt, *J* = 8.1 Hz, 1H), 2.33 (s, 3H), 2.06 – 1.98 (m, 1H), 1.27 (s, 6H), 1.26 (s, 6H), 1.03 (appt, *J* = 7.0 Hz, 1H) ppm.

^{13}C NMR (75 MHz, $CDCl_3$) δ 137.6, 135.2, 130.0, 127.9, 127.7, 126.4, 126.0 (q, *J* = 271.9 Hz), 84.0, 25.6 (q, *J* = 35.2 Hz), 25.8, 25.0, 24.9, 21.6 (q, *J* = 3.3 Hz), 2.3 (bs) ppm.

^{19}F NMR (282 MHz, $CDCl_3$) δ -55.5 (d, *J* = 7.6 Hz) ppm.

^{11}B NMR (160 MHz, $CDCl_3$) δ 32.3 ppm.

HRMS-ESI m/z calcd for $C_{17}H_{22}BF_3O_2$ $[M+H]^+$ 327.1738, found 327.1746.

$[\alpha]_D^{25}$ = -54.3 (c = 1.00, $CHCl_3$).

2-((1*S*,2*S*,3*R*)-2-(3-Fluorophenyl)-3-(trifluoromethyl)cyclopropyl)-4,4,5,5-tetramethyl-1,3,2-dioxoborolane (2e)

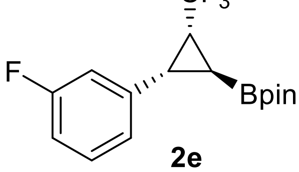

2e

Prepared from alkenylboronate **1e** (150 mg, 0.61 mmol), trifluoromethyldiazoethane (1.73 mL (0.70 M), 1.22 mmol), $Cu(NCMe)_4PF_6$ (11.3 mg, 0.03 mmol) and (*S*,*S*)-**L3** (10.1 mg, 0.03 mmol) following the general procedure and purified by flash chromatography. Yellow solid (135 mg, 68%). **M.p.** 62-63 °C.

^{1}H NMR (300 MHz, $CDCl_3$) δ 7.29 – 7.19 (m, 1H), 7.08 (d, *J* = 7.7 Hz, 1H), 7.03 – 6.96 (m, 1H), 6.96 – 6.88 (m, 1H), 2.56 (appt, *J* = 8.1 Hz, 1H), 2.10 – 1.98 (m, 1H), 1.27 (s, 6H), 1.26 (s, 6H), 1.01 (appt, *J* = 7.0 Hz, 1H).

^{13}C NMR (75 MHz, $CDCl_3$) δ 162.7 (d, *J* = 245.2 Hz), 138.1 (d, *J* = 7.8 Hz), 129.6 (d, *J* = 8.5 Hz), 125.9 (q, *J* = 272,2 Hz) 125.1, 116.4 (d, *J* = 21.8 Hz), 114.1 (d, *J* = 21.1 Hz), 84.3, 25.6 (q, *J* = 35.8 Hz), 25.2 (bs), 24.9, 24.8, 2.5.

^{19}F NMR (282 MHz, $CDCl_3$) δ -55.6 (d, *J* = 7.3 Hz, 3F), -108.1 – -108.4 (m, 1F) ppm.

^{11}B NMR (160 MHz, $CDCl_3$) δ 32.2 ppm.

HRMS-ESI m/z calcd for $C_{16}H_{19}BF_4O_2$ $[M+H]^+$ 331.1487, found 331.1492

$[\alpha]_D^{25}$ = -27.1 (c = 1.05, $CHCl_3$).

3-((1*S*,2*S*,3*R*)-2--4,4,5,5-Tetramethyl-1,3,2-dioxoborolane-3-(trifluoromethyl)cyclopropyl) phenyl acetate (2f)

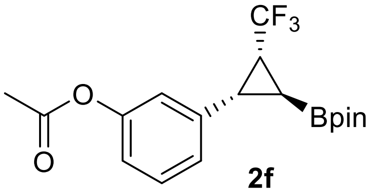

2f

Prepared from alkenylboronate **1f** (143 mg, 0.61 mmol), trifluoromethyldiazoethane (2.44 mL (0.99 M), 2.42 mmol) , $Cu(NCMe)_4PF_6$ (11.3 mg, 0.03 mmol) and (*S*,*S*)-**L3** (10.1 mg, 0.03 mmol) following the general procedure and purified by flash chromatography. Yellow oil (112 mg, 50%).

^{1}H NMR (300 MHz, $CDCl_3$) δ 7.29 (t, *J* = 7.9 Hz, 1H), 7.18 (d, *J* = 7.6 Hz, 1H), 7.01 (s, 1H), 6.97 (d, *J* = 8.1 Hz, 1H), 2.59 (appt, *J* = 8.1 Hz, 1H), 2.29 (s, 3H), 2.10 – 1.96 (m, 1H), 1.26 (s, 6H), 1.25 (s, 6H), 1.00 (appt, *J* = 7.0 Hz, 1H) ppm.

^{13}C NMR (126 MHz, $CDCl_3$) δ 169.5, 150.4, 137.1, 129.0, 126.9, 125.7 (q, *J* = 271.5 Hz), 122.4, 120.2, 84.2, 25.4 (q, *J* = 35.6 Hz), 25.2, 24.8, 24.6, 21.1 ppm. (C-B signal not observed due to low intensity)

^{19}F NMR (282 MHz, $CDCl_3$) δ -55.6 (d, *J* = 7.4 Hz) ppm.

^{11}B NMR (160 MHz, $CDCl_3$) δ 32.3 ppm.

HRMS-ESI m/z calcd for $C_{18}H_{22}BF_3O_4$ $[M+H]^+$ 371.1636, found 371.1643.

$[\alpha]_D^{25}$ = -39.9 (c = 0.97, $CHCl_3$).

2-((1*S*,2*S*,3*R*)-2-(*p*-Tolyl)-3-(trifluoromethyl)cyclopropyl)-4,4,5,5-tetramethyl-1,3,2-dioxoborolane (2g)

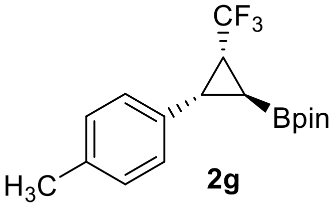

2g

Prepared from alkenylboronate **1g** (148 mg, 0.61 mmol), trifluoromethyldiazoethane (0.98 mL (1.23 M), 1.21), $Cu(NCMe)_4PF_6$ (11.3 mg, 0.03 mmol) and (*S*,*S*)-**L3** (10.1 mg, 0.03 mmol) following the general procedure and purified by flash chromatography. Yellow oil (153 mg, 77%).

^{1}H NMR (300 MHz, $CDCl_3$) δ 7.18 (d, *J* = 8.0 Hz, 2H), 7.09 (d, *J* = 8.0 Hz, 2H), 2.54 (appt, *J* = 8.1 Hz, 1H), 2.32 (s, 3H), 2.06-1.96 (m, 1H), 1.27 (s, 6H), 1.26 (s, 6H), 1.01 (appt, *J* = 6.9 Hz, 1H) ppm.

^{13}C NMR (126 MHz, $CDCl_3$) δ 136.6, 132.3, 129.4, 129.3, 126.2 (q, *J* = 272.0 Hz), 84.0, 25.4 (q, *J* = 35.3 Hz), 25.3 (bs), 24.9, 24.8, 21.2, 2.3 (bs) ppm.

^{19}F NMR (282 MHz, $CDCl_3$) δ -55.4 (d, *J* = 7.3 Hz) ppm.

^{11}B NMR (160 MHz, $CDCl_3$) δ 32.3 ppm.

HRMS-ESI m/z calcd for $C_{17}H_{23}BF_3O_2$ $[M+H]^+$ 327.1738, found 327.1751.

$[\alpha]_D^{25}$ = -33.2 (c = 0.94, $CHCl_3$).

2-((1*S*,2*S*,3*R*)-2-(*p*-Chlorophenyl)-3-(trifluoromethyl)cyclopropyl)-4,4,5,5-tetramethyl-1,3,2-dioxoborolane (2h)

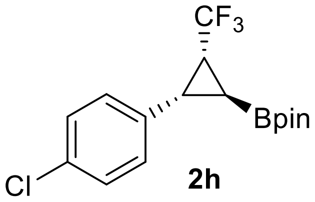

2h

Prepared from alkenylboronate **1h** (160 mg, 0.61 mmol), trifluoromethyldiazoethane (2.44 mL (0.99 M), 2.44 mmol), $Cu(NCMe)_4PF_6$ (11.3 mg, 0.03 mmol) and (*S*,*S*)-**L3** (10.1 mg, 0.03 mmol) following the general procedure and purified by flash chromatography. Yellow solid (84 mg, 40%). **M.p.** 67-68 °C.

^{1}H NMR (500 MHz, $CDCl_3$) δ 7.28 – 7.25 (m, 2H), 7.24 – 7.21 (m, 2H), 2.54 (appt, *J* = 8.2 Hz, 1H), 2.08 – 2.00 (m, 1H), 1.27 (s, 6H), 1.27 (s, 6H), 1.00 (appt, *J* = 6.9 Hz, 1H) ppm.

^{13}C NMR (126 MHz, $CDCl_3$) δ 134.0, 132.9, 130.8, 128.4, 125.9 (q, *J* = 272.7 Hz), 84.2, 25.5 (q, *J* = 35.7 Hz), 24.9 (q, *J* = 1.9 Hz), 24.9, 24.8, 2.5 (bs) ppm.

^{19}F NMR (282 MHz, $CDCl_3$) δ -55.6 (d, *J* = 7.4 Hz) ppm.

^{11}B NMR (160 MHz, $CDCl_3$) δ 32.3 ppm.

HRMS-ESI m/z calcd for $C_{16}H_{19}BClF_3O_2$ $[M+H]^+$ 347.1191, found 347.1201.

$[\alpha]_D^{25}$ = -50.1 (c = 1.03, $CHCl_3$).

2-((1*S*,2*S*,3*R*)-2-(*p*-Bromophenyl)-3-(trifluoromethyl)cyclopropyl)-4,4,5,5-tetramethyl-1,3,2-dioxoborolane (2i)

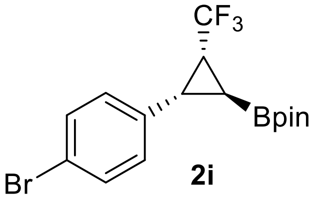

2i

Prepared from alkenylboronate **1i** (187 mg, 0.61 mmol), trifluoromethyldiazoethane (2.42 mL (1.04 M), 2.44 mmol), $Cu(NCMe)_4PF_6$ (11.3 mg, 0.03 mmol) and (*S*,*S*)-**L3** (10.1 mg, 0.03 mmol) following the general procedure and purified by flash chromatography. Yellow solid (123 mg, 52%). **M.p.** 70-71°C.

^{1}H NMR (300 MHz, $CDCl_3$) δ 7.40 (d, *J* = 8.3 Hz, 2H), 7.16 (d, *J* = 8.3 Hz, 2H), 2.52 (appt, *J* = 8.1 Hz, 1H), 2.10 – 1.97 (m, 1H), 1.28 (s, 6H), 1.27 (s, 6H), 1.00 (appt, *J* = 6.9 Hz, 1H) ppm.

^{13}C NMR (126 MHz, $CDCl_3$) δ 134.6, 131.3, 131.1, 125.9 (q, *J* = 272.7 Hz), 121.0, 84.2, 25.4 (q, *J* = 35.7 Hz), 25.0 (bs), 24.9, 24.8, 2.3 (bs) ppm.

^{19}F NMR (282 MHz, $CDCl_3$) δ -55.6 (d, *J* = 7.4 Hz) ppm.

^{11}B NMR (160 MHz, $CDCl_3$) δ 32.3 ppm.

HRMS-ESI m/z calcd for $C_{16}H_{19}BrBF_3O_2$ $[M+H]^+$ 391.0686, found 391.0692.

$[\alpha]_D^{25}$ = -52.1 (c = 1.03, $CHCl_3$).

2-((1*S*,2*S*,3*R*)-2-(*p*-Trifluoromethylphenyl)-3-(trifluoromethyl)cyclopropyl)-4,4,5,5-tetramethyl-1,3,2-dioxoborolane (2j)

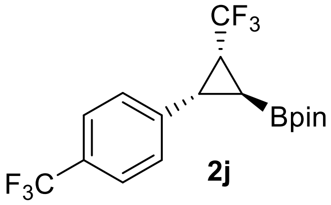

2j

Prepared from alkenylboronate **1j** (180 mg, 0.61 mmol), trifluoromethyldiazoethane (2 mL (0.60 M), 1.22 mmol), $Cu(NCMe)_4PF_6$ (11.3 mg, 0.03 mmol) and (*S*,*S*)-**L3** (10.1 mg, 0.03 mmol) following the general procedure and purified by flash chromatography. Yellow oil (140 mg, 61%).

^{1}H NMR (500 MHz, $CDCl_3$) δ 7.54 (d, *J* = 8.1 Hz, 2H), 7.41 (d, *J* = 8.0 Hz, 2H), 2.60 (appt, *J* = 8.2 Hz, 1H), 2.12 – 2.05 (m, 1H), 1.27 (s, 6H), 1.27 (s, 6H), 1.07 (appt, *J* = 7.0 Hz, 1H) ppm.

^{13}C NMR (126 MHz, $CDCl_3$) δ 139.7, 129.8, 129.4 (q, *J* = 32.4 Hz), 125.8 (q, *J* = 272.6 Hz), 125.2 (q, *J* = 3.8 Hz), 124.3 (q, *J* = 271.9 Hz), 84.3, 25.6 (q, *J* = 35.9 Hz), 25.3 (bs), 24.9, 24.8, 2.4 (bs) ppm.

^{19}F NMR (282 MHz, $CDCl_3$) δ -55.7 (d, *J* = 7.4 Hz, 3F), -56.9 (s, 3F) ppm.

^{11}B NMR (160 MHz, $CDCl_3$) δ 32.2 ppm.

HRMS-ESI m/z calcd for $C_{17}H_{19}BF_6O_2$ $[M+H]^+$ 381.1445, found 381.1457.

$[\alpha]_D^{25}$ = -42.7 (c = 0.99, $CHCl_3$).

2-((1*S*,2*S*,3*R*)-2-(*p*-*Tert*butylphenyl)-3-(trifluoromethyl)cyclopropyl)-4,4,5,5-tetramethyl-1,3,2-dioxoborolane (2k)

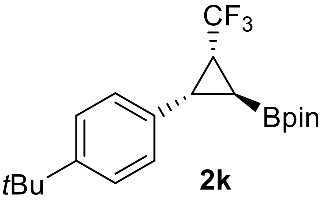

Prepared from alkenylboronate **1k** (173 mg, 0.61 mmol), trifluoromethyldiazoethane (2.42 mL (1.04 M), 2.44 mmol), $Cu(NCMe)_4PF_6$ (11.3 mg, 0.03 mmol) and (*S*,*S*)-**L3** (10.1 mg, 0.03 mmol) following the general procedure and purified by flash chromatography. Yellow solid (131 mg, 59%). **M.p.** 58-59 °C.

^{1}H NMR (300 MHz, $CDCl_3$) δ 7.31 (d, *J* = 8.3 Hz, 2H), 7.23 (d, *J* = 8.2 Hz, 2H), 2.55 (appt, *J* = 8.1 Hz, 1H), 2.07 – 1.98 (m, 1H), 1.31 (s, 9H), 1.27 (s, 6H), 1.26 (s, 6H), 1.03 (appt, *J* = 7.0 Hz, 1H) ppm.

^{13}C NMR (126 MHz, $CDCl_3$) δ 149.9, 132.3, 129.0, 126.2 (q, *J* = 278.8 Hz), 125.1, 84.0, 34.6, 31.5, 25.6 (q, *J* = 35.6 Hz), 25.4 (bs), 24.9, 24.8, 2.4 (bs) ppm.

^{19}F NMR (282 MHz, $CDCl_3$) δ -53.4 (d, *J* = 7.7 Hz) ppm.

^{11}B NMR (160 MHz, $CDCl_3$) δ 32.3 ppm.

HRMS-ESI m/z calcd for $C_{20}H_{28}BF_3O_2$ $[M+H]^+$ 369.2207, found 369.2210.

$[\alpha]_D^{25}$ = -27.7 (c = 1.00, $CHCl_3$).

2-((1*S*,2*S*,3*R*)-2-(*p*-Methoxy)-3-(trifluoromethyl)cyclopropyl)-4,4,5,5-tetramethyl-1,3,2-dioxoborolane (2l)

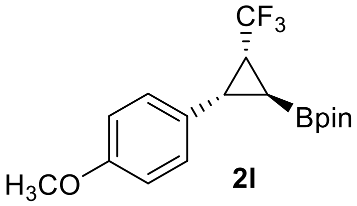

Prepared from alkenylboronate **1l** (157 mg, 0.61 mmol), trifluoromethyldiazoethane (1 mL, 1.21 mmol), $Cu(NCMe)_4PF_6$ (11.3 mg, 0.03 mmol) and (*S*,*S*)-**L3** (10.1 mg, 0.03 mmol) following the general procedure and purified by flash chromatography. Yellow solid (124 mg, 60%). **M.p.** 65-66 °C.

^{1}H NMR (500 MHz, $CDCl_3$) δ 7.22 (d, *J* = 8.7 Hz, 2H), 6.83 (d, *J* = 8.0 Hz, 2H), 3.79 (s, 1H), 2.53 (appt, *J* = 8.0 Hz, 1H), 2.02 – 1.95 (m, 1H), 1.27 (s, 6H), 1.27 (s, 6H), 0.99 (appt, *J* = 6.9 Hz, 1H) ppm.

^{13}C NMR (126 MHz, $CDCl_3$) δ 158.7, 130.4, 127.4, 126.2 (q, *J* = 272.7 Hz), 113.6, 84.0, 55.3, 25.4 (q, *J* = 35.1 Hz), 24.9 (bs), 24.9, 24.8, 2.2 (bs) ppm.

^{19}F NMR (282 MHz, $CDCl_3$) δ -55.4 (d, *J* = 7.4 Hz) ppm.

^{11}B NMR (160 MHz, $CDCl_3$) δ 32.6 ppm.

HRMS-ESI m/z calcd for $C_{17}H_{22}BF_3O_3$ $[M+H]^+$ 343.1687, found 343.1695.

$[\alpha]_D^{25}$ = -13.5 (c = 1.01, $CHCl_3$).

Methyl 4-((1*S*,2*S*,3*R*)-2-(4,4,5,5-tetramethyl-1,3,2-dioxoborolan-2-yl)-3-(trifluoromethyl)cyclopropyl)benzoate (2o)

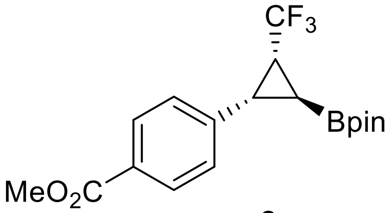

2o

Prepared from alkenyl boronate **1o** (174 mg, 0.61 mmol), trifluoromethyldiazoethane (1.89 mL (1.28 M), 1.22 mmol), $Cu(NCMe)_4PF_6$ (11.3 mg, 0.03 mmol) and (*S*,*S*)-**L3** (10.1 mg, 0.03 mmol) following the general procedure and purified by flash chromatography (hexane/AcOEt 95:5). White solid (113 mg, 51%). **M.p.** 77-78 °C.

^{1}H NMR (400 MHz, $CDCl_3$) δ 7.96 (d, *J* = 7.9 Hz, 2H), 7.36 (d, *J* = 7.9 Hz, 2H), 3.90 (s, 3H), 2.60 (appt, *J* = 8.4 Hz, 1H), 2.13 – 2.02 (m, 1H), 1.27 (s, 12H), 1.08 (appt, *J* = 6.8 Hz, 1H) ppm.

^{13}C NMR (101 MHz, $CDCl_3$) δ 167.1, 140.9, 129.5, 129.0, 125.8 (q, *J* = 272.8 Hz), 84.2, 52.2, 25.7 (q, *J* = 35.8 Hz), 25.5 (bs), 24.9, 24.8, 2.6 (bs) ppm.

^{19}F NMR (376 MHz, $CDCl_3$) δ -61.2 (d, *J* = 7.3 Hz, 3F) ppm.

^{11}B NMR (160 MHz, $CDCl_3$) δ 31.7 ppm.

HRMS-ESI m/z calcd for $C_{18}H_{23}BF_3O_4$ $[M+H]^+$ 371.1636, found 371.1640.

$[\alpha]_D^{25}$ = -58.0 (c = 0.93, $CHCl_3$).

2-((1*S*,2*S*,3*R*)-2-(2,4,5-trimethylphenyl)-3-(trifluoromethyl)cyclopropyl)-4,4,5,5-tetramethyl-1,3,2-dioxoborolane (2p)

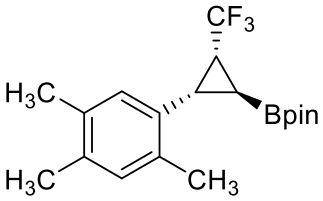

2p

Prepared from alkenyl boronate **1p** (165 mg, 0.61 mmol), trifluoromethyldiazoethane (2.55 mL (0.95 M), 1.22 mmol), $Cu(NCMe)_4PF_6$ (11.3 mg, 0.03 mmol) and (*S*,*S*)-**L3** (10.1 mg, 0.03 mmol) following the general procedure and purified by flash chromatography (hexane/AcOEt 95:5). Yellow oil (134 mg, 62%).

^{1}H NMR (400 MHz, $CDCl_3$) δ 6.95 (s, 1H), 6.92 (s, 1H), 2.40 – 2.38 (m, 1H), 2.35 (s, 3H), 2.21 (s, 6H), 2.13 – 2.04 (m, 1H), 1.28 (s, 12H), 1.09 (appt, *J* = 7.2 Hz, 1H) ppm.

^{13}C NMR (101 MHz, $CDCl_3$) δ 135.6, 135.2, 133.5, 131.3, 130.8, 129.6, 126.2 (q, *J* = 272.7 Hz), 84.1, 25.3 (q, *J* = 35.2 Hz), 25.0, 24.8, 24.6 (bs), 19.4, 18.8, 1.7 (bs) ppm.

^{19}F NMR (376 MHz, $CDCl_3$) δ -61.7 (d, *J* = 7.6 Hz, 3F) ppm.

^{11}B NMR (160 MHz, $CDCl_3$) δ 32.6 ppm.

HRMS-ESI m/z calcd for $C_{19}H_{27}BF_3O_2$ $[M+H]^+$ 355.2051, found 355.2054.

$[\alpha]_D^{25}$ = -77.8 (c = 1.08, $CHCl_3$).

2-((1*S*,2*S*,3*R*)-2-(Thiophen-3-yl)-3-(trifluoromethyl)cyclopropyl)-4,4,5,5-tetramethyl-1,3,2-dioxoborolane (2q)

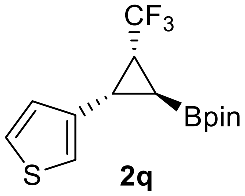

2q

Prepared from alkenylboronate **1q** (143 mg, 0.61 mmol), trifluoromethyldiazoethane (2.44 mL (0.99 M), 2.44 mmol), $Cu(NCMe)_4PF_6$ (11.3 mg, 0.03 mmol) and (*S*,*S*)-**L3** (10.1 mg, 0.03 mmol) following the general procedure and purified by flash chromatography. Yellow oil (96 mg, 50%).

^{1}H NMR (500 MHz, $CDCl_3$) δ 7.24 (dd, *J* = 4.9, 3.0 Hz, 1H), 7.09 – 7.07 (m, 1H), 7.02 (d, *J* = 4.9 Hz, 1H), 2.46 (appt, *J* = 7.9 Hz, 1H), 2.01 – 1.90 (m, 1H), 1.27 (s, 6H), 1.26 (s, 6H), 0.98 (appt, *J* = 6.9 Hz, 1H) ppm.

^{13}C NMR (126 MHz, $CDCl_3$) δ 136.4, 128.9, 126.0 (q, *J* = 272.4 Hz), 125.2, 122.9 (q, *J* = 1.7 Hz), 84.1, 25.3 (q, *J* = 35.6 Hz), 24.9, 24.8, 20.7 (q, *J* = 1.9 Hz), 3.7 (bs) ppm.

^{19}**F NMR** (282 MHz, $CDCl_3$) δ -55.7 (d, *J* = 7.8 Hz) ppm.

^{11}**B NMR** (160 MHz, $CDCl_3$) δ 32.2 ppm.

HRMS-ESI m/z calcd for $C_{14}H_{18}BF_3O_2S$ $[M+H]^+$ 319.1145, found 319.1154.

$[\alpha]_D^{25}$ = -34.9 (c = 1.00, $CHCl_3$).

2-((1*S*,2*S*,3*R*)-2-Phenethyl-3-(trifluoromethyl)cyclopropyl)-4,4,5,5-tetramethyl-1,3,2-dioxoborolane (2r)

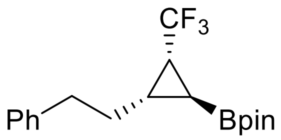

2r

Prepared from alkenylboronate **1r** (156 mg, 0.61 mmol), trifluoromethyldiazoethane (4.03 mL (0.90 M), 3.66 mmol)), $Cu(NCMe)_4PF_6$ (11.3 mg, 0.03 mmol) and (*S*,*S*)-**L3** (10.1 mg, 0.03 mmol) following the general procedure and purified by flash chromatography. Yellow oil (103 mg, 50%).

^{1}**H NMR** (500 MHz, $CDCl_3$) δ 7.32 – 7.27 (m, 2H), 7.23 – 7.17 (m, 3H), 2.76 (t, *J* = 7.7 Hz, 2H), 1.94 (td, *J* = 14.2, 7.3 Hz, 1H), 1.82 – 1.67 (m, 2H), 1.23 (s, 6H), 1.21 (s, 6H), 1.22 – 1.17 (m, 1H), 0.25 (appt, *J* = 6.7 Hz, 1H) ppm.

^{13}**C NMR** (126 MHz, $CDCl_3$) δ 141.8, 128.6, 128.5, 127.0 (q, *J* = 272.5 Hz), 126.0, 83.8, 36.0, 30.0, 24.9, 24.8, 23.6 (q, *J* = 36.1 Hz), 21.6, 4.8 (bs) ppm.

^{19}**F NMR** (282 MHz, $CDCl_3$) δ -54.0 (d, *J* = 7.3 Hz) ppm.

^{11}**B NMR** (160 MHz, CDCl3) δ 32.2 ppm.

HRMS-ESI m/z calcd for $C_{18}H_{24}BF_3O_2$ $[M+Na]^+$ 363.1714, found 363.1726.

$[\alpha]_D^{25}$ = -4.2 (c = 1.03, $CHCl_3$).

3. Derivatization products

(1*S*,2*S*,3*R*)-2-Phenyl-3-(trifluoromethyl)cyclopropyl)boronic acid (3)

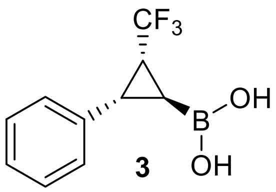

Pinacol boronate **2a** (50 mg, 0.16 mmol) and methylboronic acid (48 mg, 0.80 mmol) were added and dissolved in a solution of TFA [5% in DCM] (0.05 mL TFA, 1 mL DCM). The suspension was stirred at room temperature for 8 h. After full conversion, the mixture was concentrated under vacuum and the residue was redissolved in 0.1 N HCl (2 mL) to avoid mixed anhydride formation. The solution was then evaporated and dried to obtain boronic acid **3** (27 mg, 72%).

^{1}H NMR (300 MHz, CD_3OD) δ 7.34 – 7.16 (m, 5H), 2.54 (appt, *J* = 8.2 Hz, 1H), 2.06 – 1.94 (m, 1H), 1.14 – 1.04 (m, 1H).

^{13}C NMR (126 MHz, CD_3OD) δ 137.2, 130.3, 129.1, 127.8, 127.8 (q, *J* = 271.6 Hz), 26.6 (bs), 26.2 (q, *J* = 35.3 Hz), 3.6 (bs) ppm.

^{19}F NMR (282 MHz, CD_3OD) δ -56.1 (d, *J* = 7.9 Hz) ppm.

^{11}B NMR (160 MHz, CD_3OD) δ 29.9 ppm.

HRMS-ESI m/z calcd for $C_{10}H_9BF_3O_2$ $[M-H]^-$ 229.0653, found 229.0647.

$[\alpha]_D^{25}$ = -31.5 (c = 1.03, $CHCl_3$).

1-Methoxy-4-((1*S*, 2*S*, 3*R*)-2-phenyl-3-(trifluoromethyl)cyclopropyl)benzene (4)

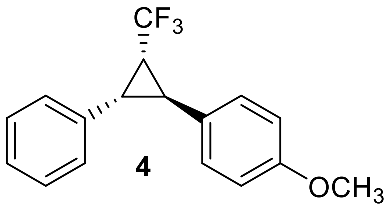

Pinacol boronate **2a** (55 mg, 0.18 mmol), $Pd_2(dba)_3{\cdot}CHCl_3$ (18.6 mg, 0.02 mmol), PPh_3 (46 mg, 0.18 mmol) and Ag_2O (61 mg, 0.26 mmol) were dissolved in deoxygenated THF (4 mL). 4-iodoanisole (62 mg, 0.26 mmol) was then added under inert atmosphere and the mixture was stirred at 70 °C for 24 h. The solvent was evaporated and the crude was purified by flash column chromatography (hexane/DCM 8:2) to obtain cyclopropane **4** as a yellow solid (23 mg, 45%). **M.p.** 69-70 °C.

^{1}H NMR (500 MHz, $CDCl_3$) δ 7.41 – 7.38 (m, 2H), 7.36 – 7.32 (m, 2H), 7.30 – 7.26 (m, 1H), 7.22 (d, *J* = 8.7 Hz, 2H), 6.90 (d, *J* = 8.7 Hz, 2H), 3.82 (s, 3H), 2.93 (appt, *J* = 6.1 Hz, 1H), 2.78 (appt, *J* = 8.0 Hz 1H), 2.22 – 2.06 (m, 1H) ppm.

^{13}C NMR (126 MHz, $CDCl_3$) δ 158.9, 135.2, 130.8, 129.4, 128.4, 128.3, 127.3, 125.9 (q, *J* = 272.7 Hz), 114.3, 55.5, 29.4 (q, *J* = 1.5 Hz), 29.0 (q, *J* = 34.9 Hz), 24.2 (q, *J* = 2.8 Hz) ppm.

^{19}F NMR (282 MHz, $CDCl_3$) δ -54.9 (d, *J* = 7.4 Hz) ppm.

HRMS-ESI m/z calcd for $C_{17}H_{16}F_3O$ $[M+H]^+$ 293.1148, found 293.1157.

$[\alpha]_D^{25}$ = -107.7 (c = 1.07, $CHCl_3$).

(1*S*,2*S*,3*R*)-2-Phenyl-3-(trifluoromethyl)cyclopropanol (5)

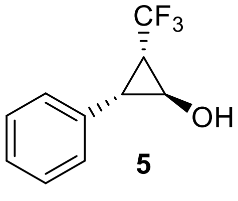

5

Pinacol boronate **2a** (32 mg, 0.10 mmol) was dissolved in THF (1.5 mL) and a solution of 3 M NaOH (0.07 mL) and 30% H_2O_2 (0.035 mL) was added at 0 ºC under vigorous stirring. The reaction was warmed to rt and stirring continued for 30 min. After completion of the reaction, the mixture was extracted with AcOEt and washed with saturated NH_4Cl. Then it was dried with Na_2SO_4 and concentrated under vacuum. The crude was purified by a short flash column chromatography (hexane/ EtOAc, 9:1) to obtain alcohol **5** as a yellow oil (14 mg, 68%).

^{1}H NMR (300 MHz, $CDCl_3$) δ 7.32 – 7.23 (m, 5H), 4.29 (t, *J* = 3.7 Hz, 1H), 2.76 (dd, *J* = 10.7, 4.3 Hz, 1H), 2.48-2.22 (bs, 1H), 2.16 – 2.01 (m, 1H) ppm.

^{13}C NMR (75 MHz, $CDCl_3$) δ 133.4, 129.2, 128.5, 127.4, 125.1 (q, *J* = 272.3 Hz), 52.7 (q, *J* = 3.7 Hz), 31.1, 29.7 (q, *J* = 34.8 Hz) ppm.

^{19}F NMR (282 MHz, $CDCl_3$) δ -54.1 (d, *J* = 8.1 Hz) ppm.

HRMS-ESI m/z calcd for $C_{10}H_9F_3O$ $[M+H]^+$ 203.0678, found 203.0656.

$[\alpha]_D^{25}$ = -49.9 (c = 1.06, $CHCl_3$).

(1*S*,2*S*,3*S*)-*N*-Benzyl-2-phenyl-3-(trifluoromethyl)cyclopropan-1-amine (6)

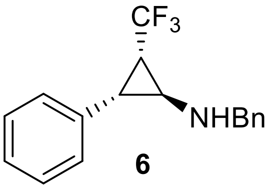

6

Pinacol boronate **2a** (100 mg, 0.32 mmol) was dissolved in DCM (2 mL) and BCl_3 (1.75 mL, 1.6 mmol, 1M in Heptane) was added slowly. After 1.5 h at rt, the volatiles compounds were pumped off and the mixture was dissolved in 2 mL of DCM. The reaction was cooled at 0 ºC and benzyl azide (134 mg, 0.96 mmol) was added. The mixture was then warmed to rt for 2 h and the organic phase was extracted with Et_2O/NaOH (2 M in H_2O). The crude was purified by flash column chromatography (DCM to DCM:MeOH 95:5) to obtain the product **6** as a yelow oil (47 mg, 51%).

^{1}H NMR (300 MHz, $CDCl_3$) δ 7.43 – 7.32 (m, 4H), 7.32 – 7.20 (m, 6H), 3.96 (s, 2H), 3.12 (dd, *J* = 5.0, 3.8 Hz, 1H), 2.64 (dd, *J* = 9.9, 5.0 Hz, 1H), 2.12 (bs, 1H), 2.06 – 1.86 (m, 1H) ppm.

^{13}C NMR (126 MHz, $CDCl_3$) δ 139.7, 134.6, 129.3, 128.8, 128.4, 128.3, 127.5, 127.1, 125.7 (q, *J* = 272.4 Hz), 53.4, 38.3 (q, *J* = 2.9 Hz), 30.0 (bs), 29.4 (q, *J* = 34.6 Hz) ppm.

^{19}F NMR (282 MHz, $CDCl_3$) δ -65.3 (d, *J* = 7.8 Hz) ppm.

HRMS-ESI m/z calcd for $C_{17}H_{17}F_3N$ $[M+H]^+$ 292.1308, found 292.1313.

$[\alpha]_D^{25}$ = -49.3 (c = 1.00, $CHCl_3$).

3-(Azidomethyl)-2-methoxypyridine (7)[S3]

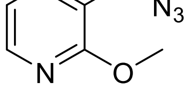

7

(2-methoxypyridin-3-yl)methanol (0.5 g, 3.59 mmol) and diphenyl phosphoryl azide (0.9 mL, 4.13 mmol) were dissolved in dry toluene (9 mL). The reaction was cooled to 0 °C and DBU (0.7 mL, 4.38 mmol) was added dropwise. The mixture continued at 0 °C for 2 hours and then is warmed to room temperature for 48 hours. The reaction mixture was washed with 1N HCl and brine. Then it was dried with Na_2SO_4 and concentrated under vacuum. The crude was purified by column chromatography (hexane/ EtOAc, 9:1) to obtain azide **7** as a colorless oil (307 mg, 52%).

[S3] Following a described procedure: Odlo, K.; Fournier-Dit-Chabert, J.; Ducki, S.; Gani, O. A. B. S. M.; Sylte, I.; Hansen, T. V. *Bioorg. Med. Chem.* **2010**, *18*, 6874–6885.

^{1}H NMR (300 MHz, $CDCl_3$) δ 8.14 (d, *J* = 4.7 Hz, 1H), 7.54 (d, *J* = 7.2 Hz, 1H), 6.89 (t, *J* = 5.5 Hz, 1H), 4.35 (s, 2H), 4.00 (s, 3H) ppm.

^{13}C NMR (75 MHz, $CDCl_3$) δ 161.7, 146.6, 137.7, 118.4, 116.6, 53.5, 49.7 ppm.

HRMS-ESI m/z calcd for $C_7H_8N_4O$ $[M+H]^+$ 165.0771, found 165.0773.

(1*S*,2*S*,3*S*)-*N*-((2-Methoxypyridin-3-yl)methyl)-2-phenyl-3-(trifluoromethyl)cyclopropan-1-amine (8)

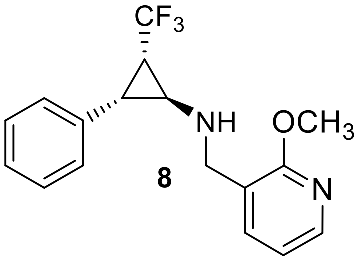

8

Pinacol boronate **2a** (92 mg, 0.30 mmol) was dissolved in DCM (2 mL) and BCl_3 (1.50 mL, 1.5 mmol, 1M in heptane) was added slowly. After 1.5 h at rt, the volatiles compounds were pumped off and the mixture was dissolved in 2 mL of DCM. The reaction was cooled at 0 ºC and azide **7** (146 mg, 0.89 mmol) was added. The mixture was then warmed to rt for 4 h and the organic phase was extracted with Et_2O/NaOH 2 M. The crude was purified by flash column chromatography (DCM:AcOEt 95:5 to DCM:AcOEt 9:1) to obtain the product **8** as a yelow oil (52 mg, 55%).

^{1}H NMR (500 MHz, $CDCl_3$) δ 8.13 (dd, *J* = 5.0, 1.9 Hz, 1H), 7.54 (dd, *J* = 7.2, 1.9 Hz, 1H), 7.32 – 7.21 (m, 5H), 6.88 (dd, *J* = 7.1, 5.0 Hz, 1H), 4.02 (s, 3H), 3.95 (d, *J* = 13.8 Hz, 1H), 3.88 (d, *J* = 13.8 Hz, 1H), 3.05 (dd, *J* = 5.1, 3.8 Hz, 1H), 2.66 (dd, *J* = 10.1, 5.0 Hz, 1H), 2.04 – 1.95 (m, 1H) ppm. ppm.

^{13}C NMR (126 MHz, $CDCl_3$) δ 162.3, 146.4, 138.6, 134.1, 129.2, 128.5, 128.4, 127.3, 125.5 (q, *J* = 272.7 Hz), 117.0, 53.6, 48.3, 37.7 (bs), 29.5 (bs), 28.9 (q, *J* = 34.8 Hz) ppm.

^{19}F NMR (282 MHz, $CDCl_3$) δ -65.3 (d, *J* = 7.7 Hz) ppm.

HRMS-ESI m/z calcd for $C_{17}H_{17}F_3N_2O$ $[M+H]^+$ 323.1366, found 323.1374.

$[\alpha]_D^{25}$ = -46.2 (c = 1.09, $CHCl_3$).

NMR spectra

(*E*)-4,4,5,5-tetramethyl-2-(2,4,5-trimethylstyryl)-1,3,2-dioxaborolane (1p)

^{1}H NMR (400 MHz, $CDCl_3$)

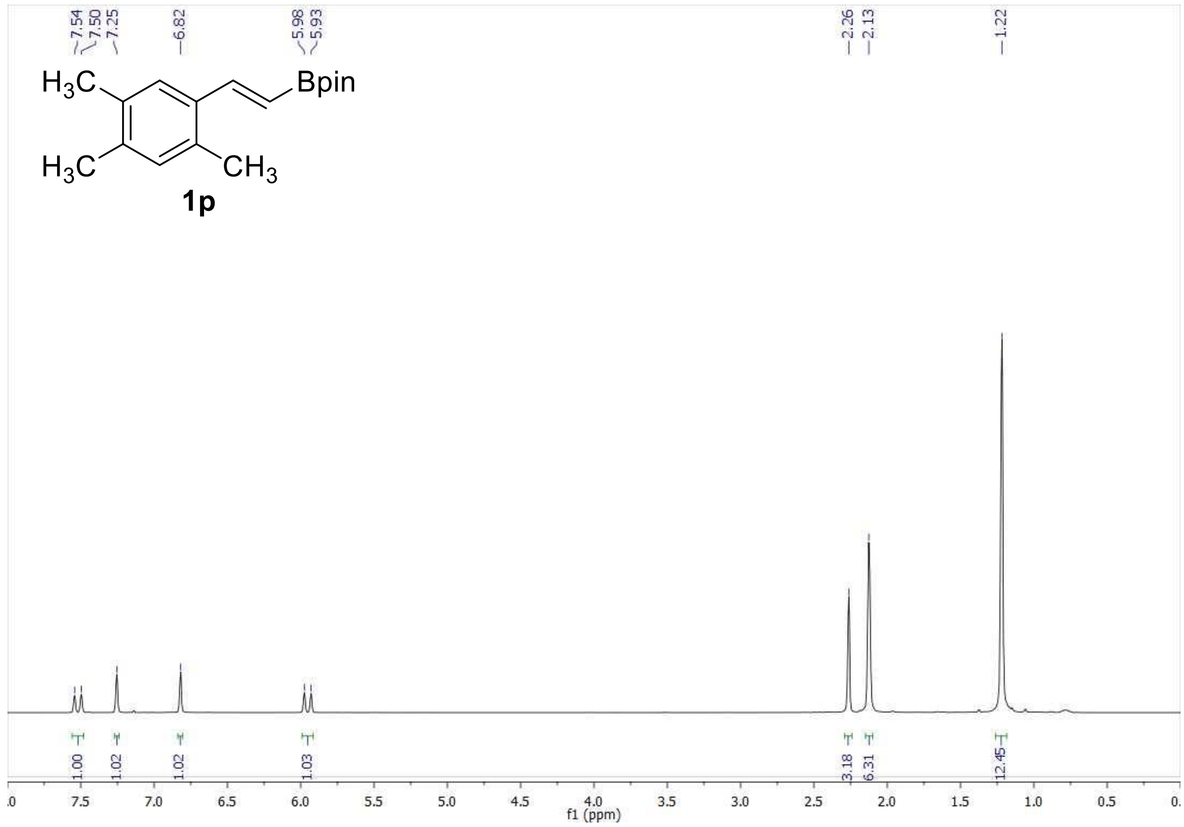

^{13}C NMR (101 MHz, $CDCl_3$)

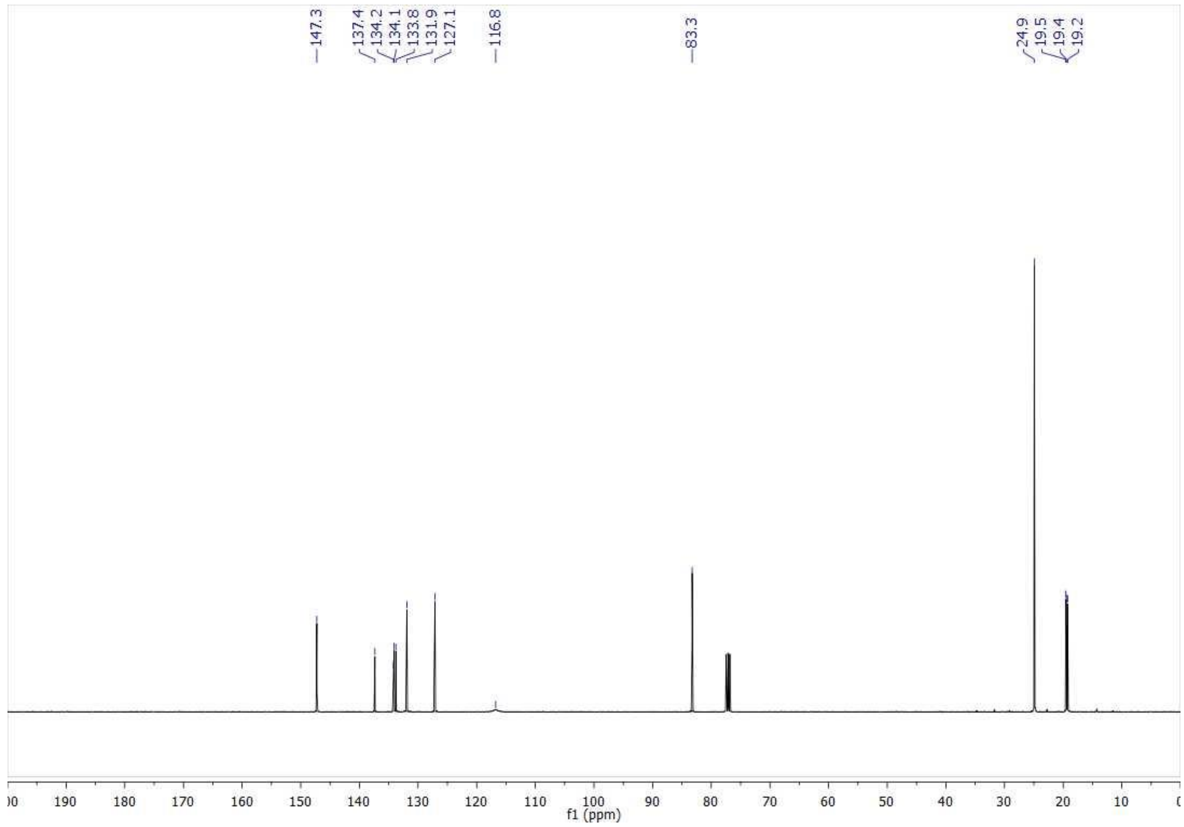

^{11}B NMR (160 MHz, $CDCl_3$)

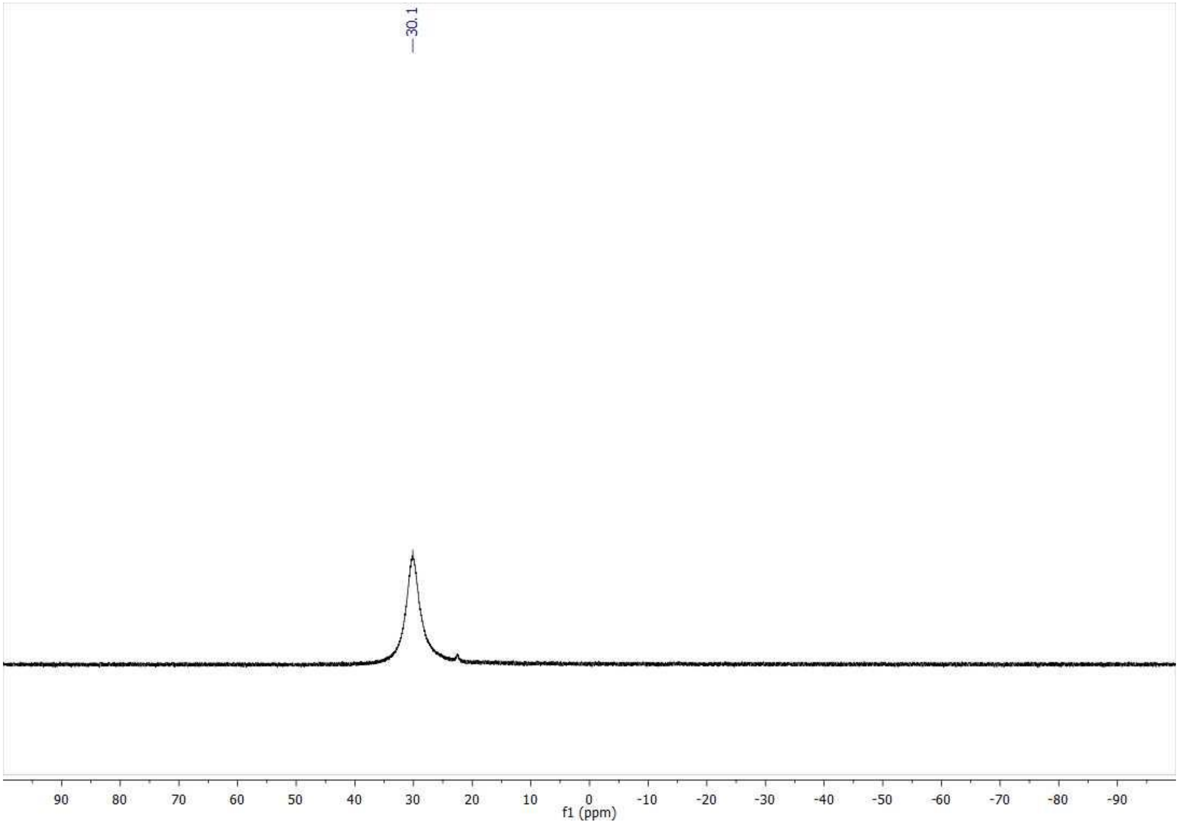

2-((1*S*,2*S*,3*R*)-2-Phenyl-3-(trifluoromethyl)cyclopropyl)-4,4,5,5-tetramethyl-1,3,2-dioxoborolane (2a)

^{1}H NMR (500 MHz, $CDCl_3$)

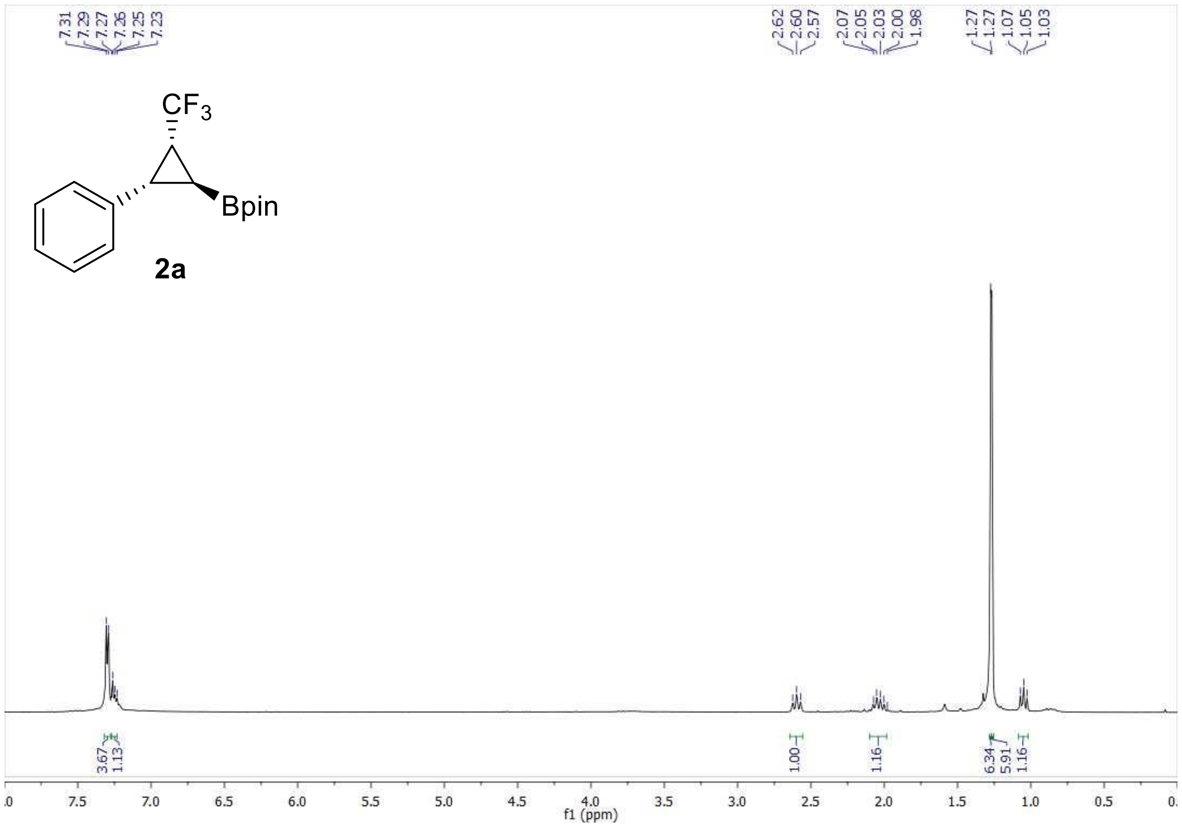

^{13}C NMR (126 MHz, $CDCl_3$)

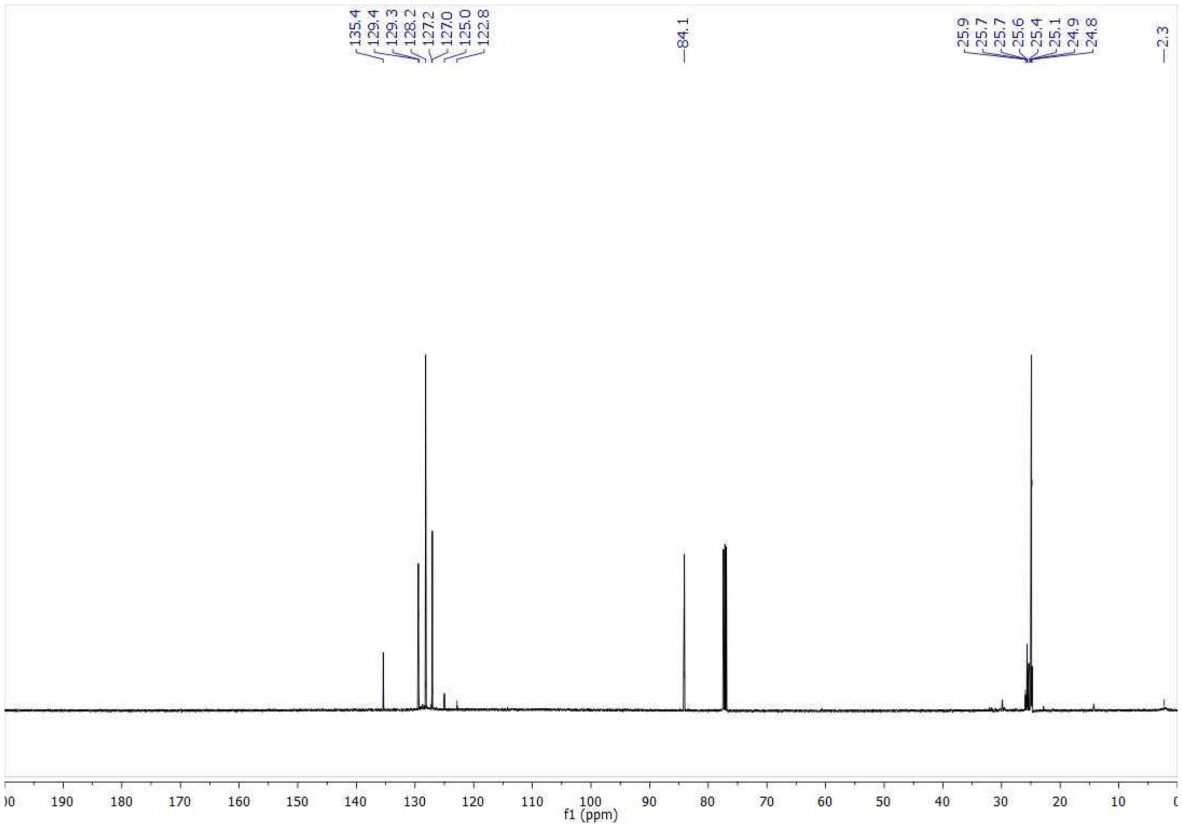

^{19}F NMR (282 MHz, $CDCl_3$)

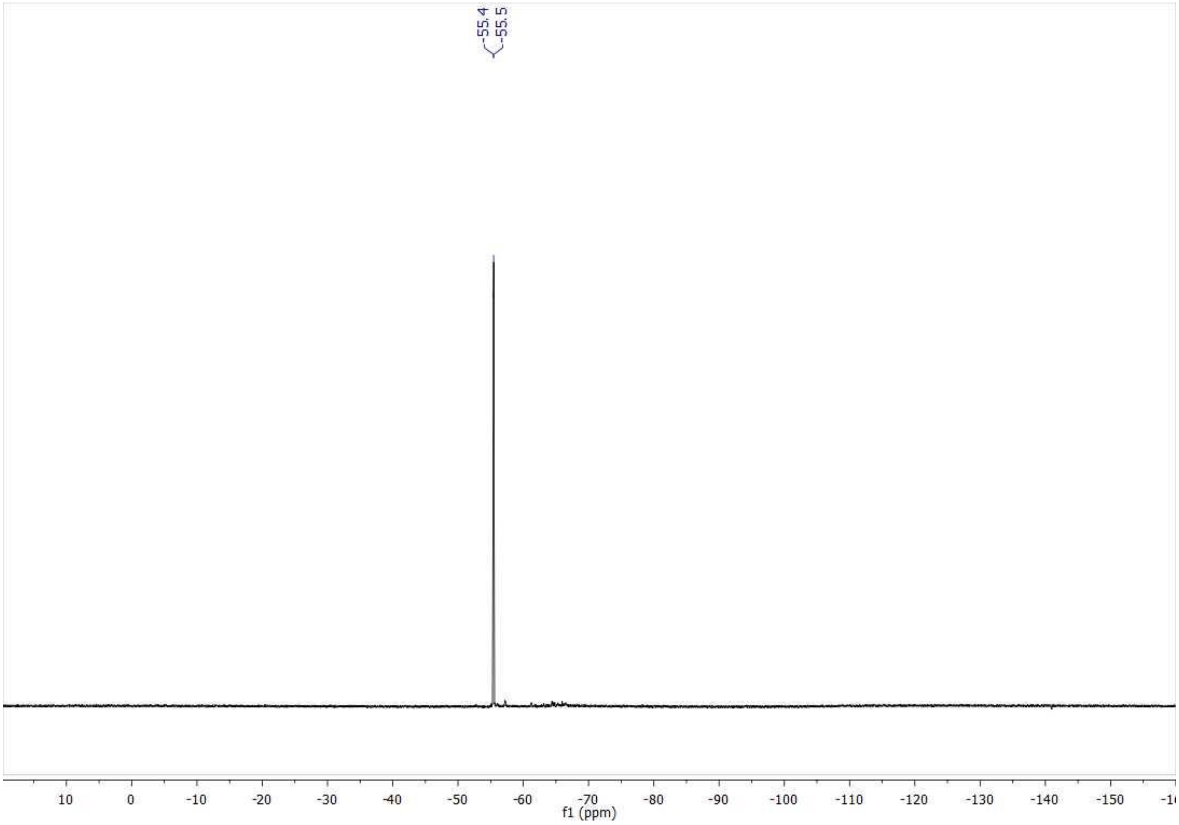

^{11}B NMR (160 MHz, $CDCl_3$)

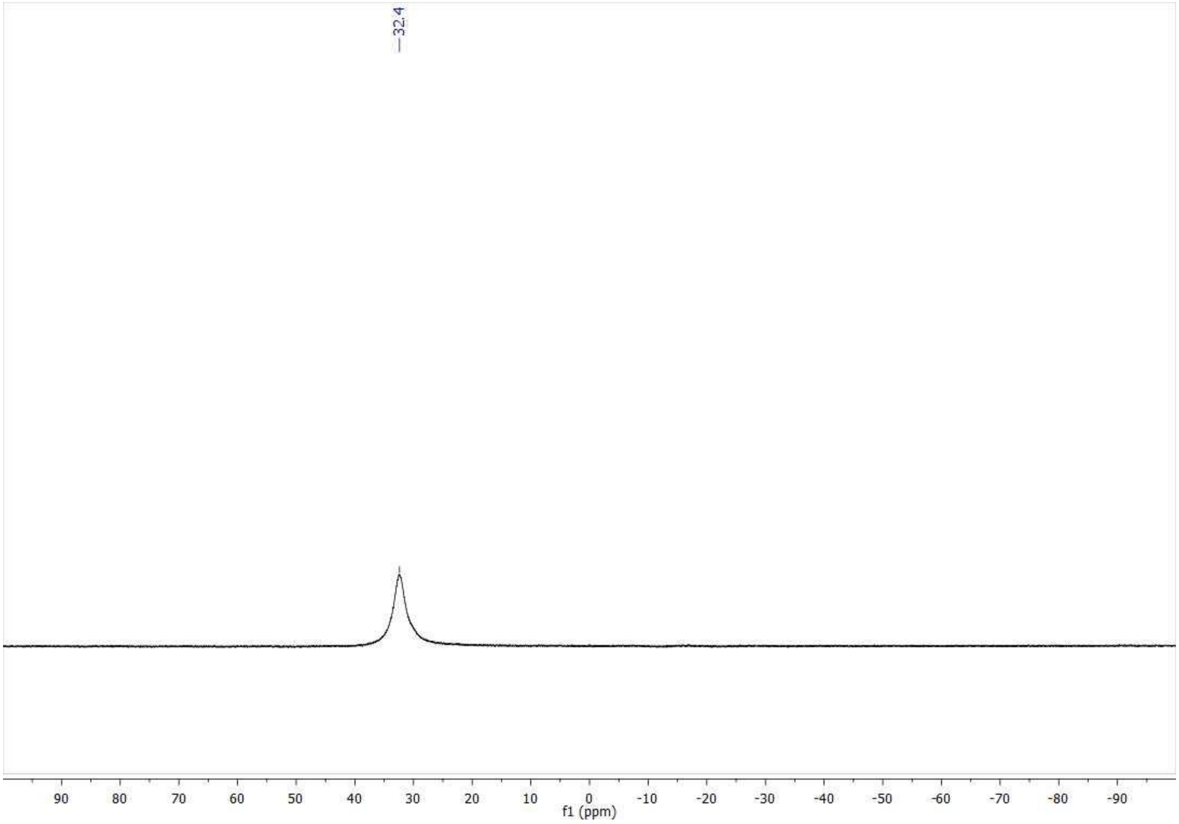

2-((1*R*,2*R*,3*R*)- and (1*S*,2*S*,3*S*)-2-Phenyl-3-(trifluoromethyl)cyclopropyl)-4,4,5,5-tetramethyl-1,3,2-dioxoborolane ((±)-2a´)

^{1}H NMR (500 MHz, $CDCl_3$

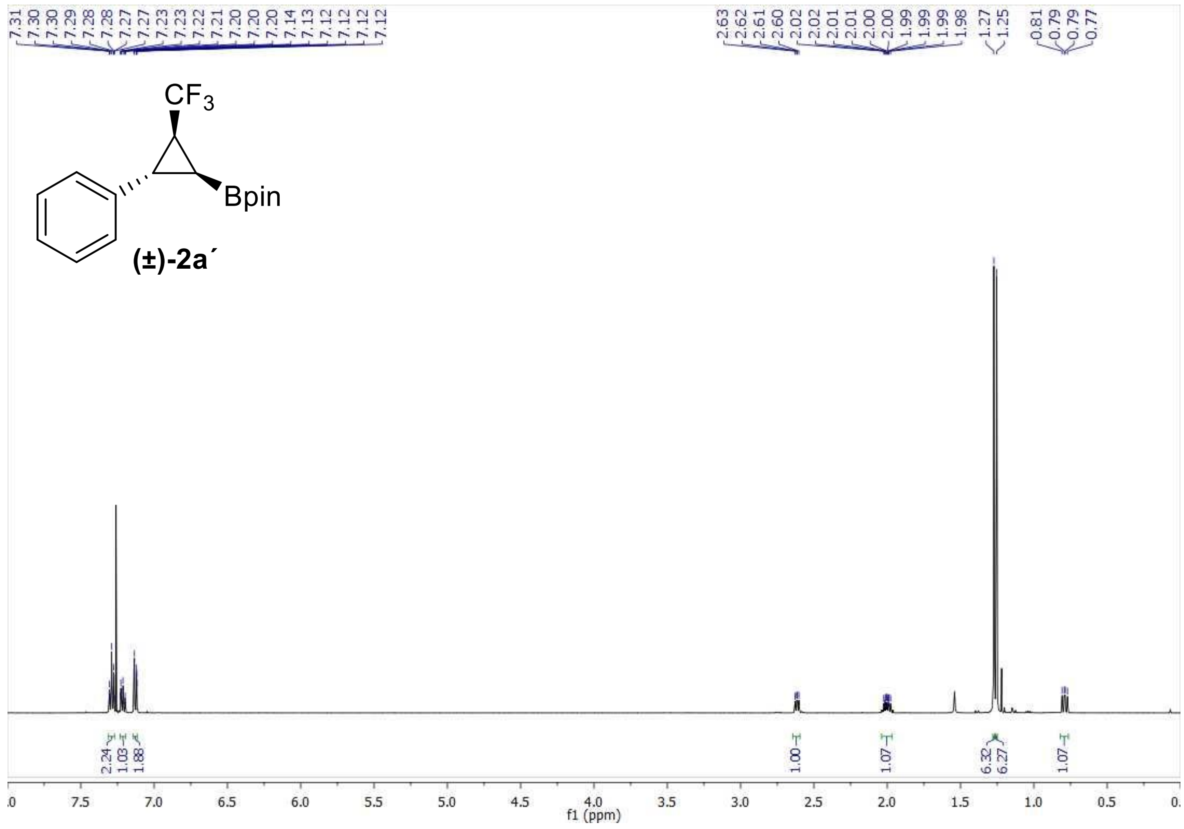

^{13}C NMR (126 MHz, $CDCl_3$)

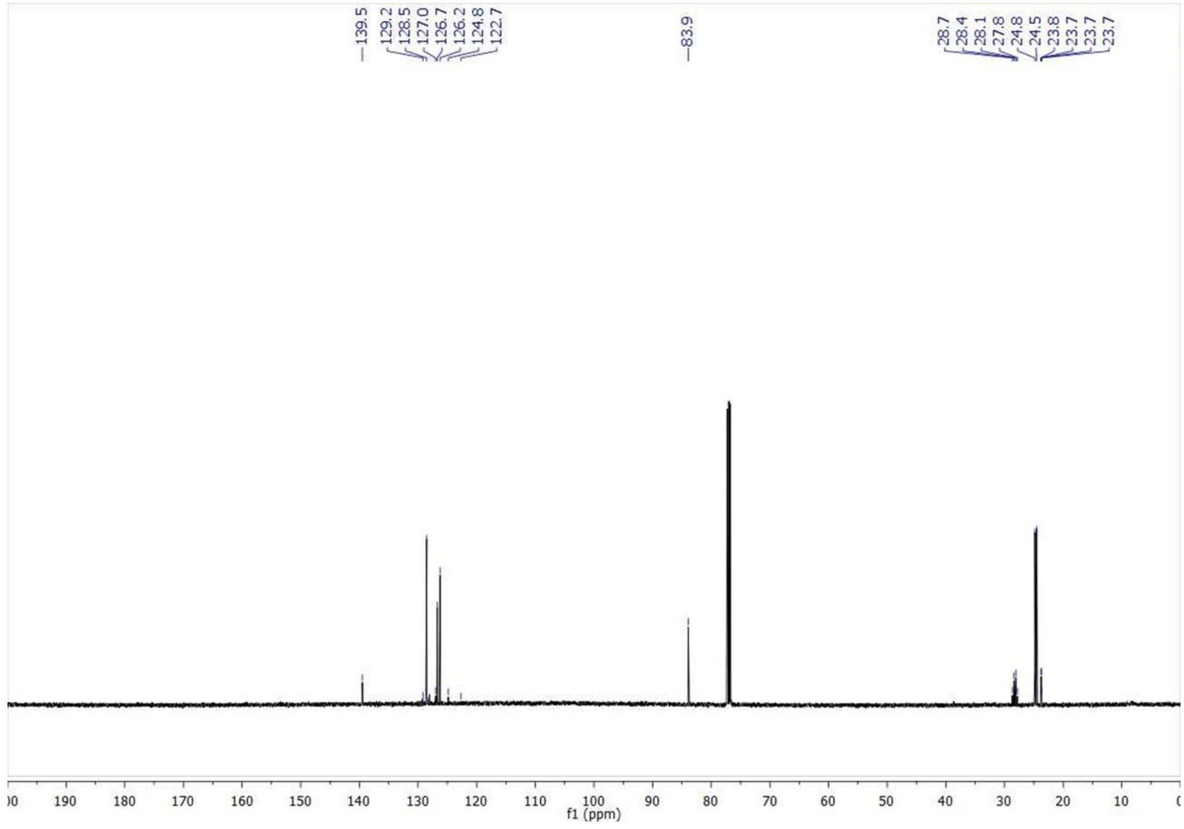

^{19}F NMR (282 MHz, $CDCl_3$)

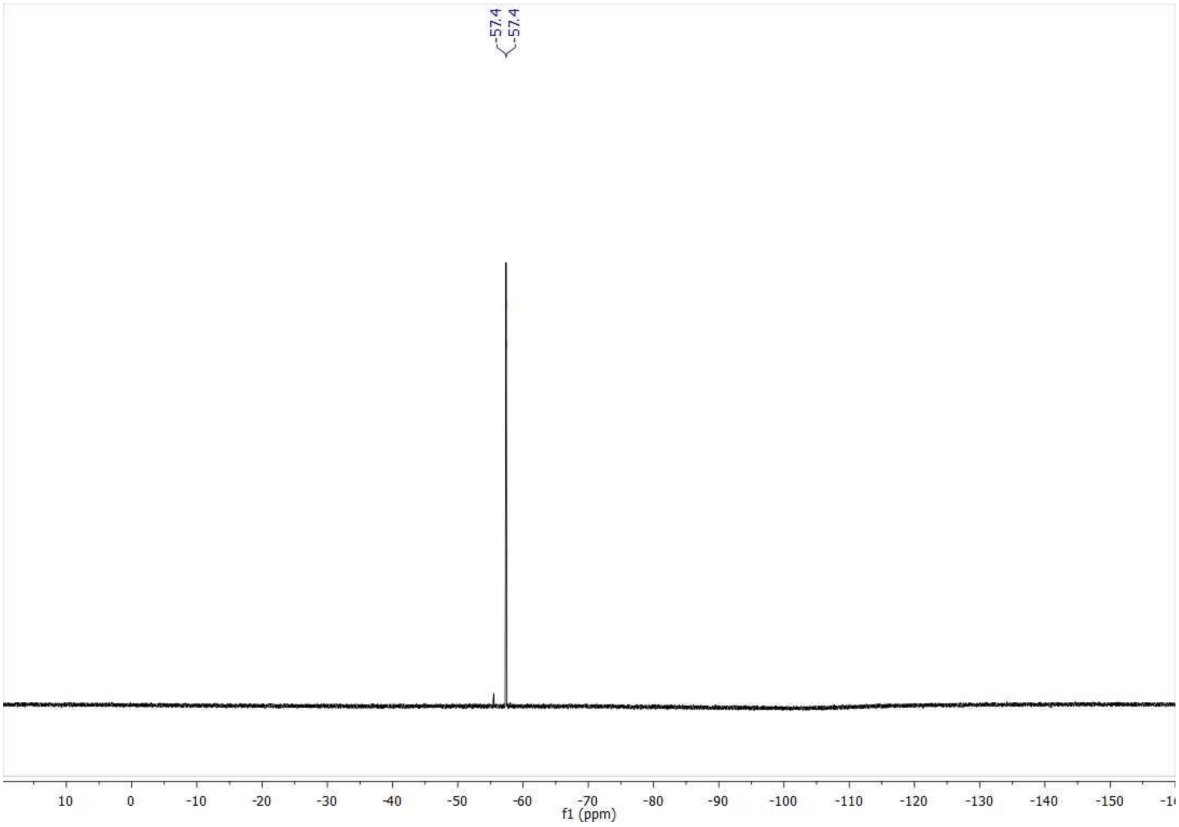

^{11}B NMR (160 MHz, $CDCl_3$)

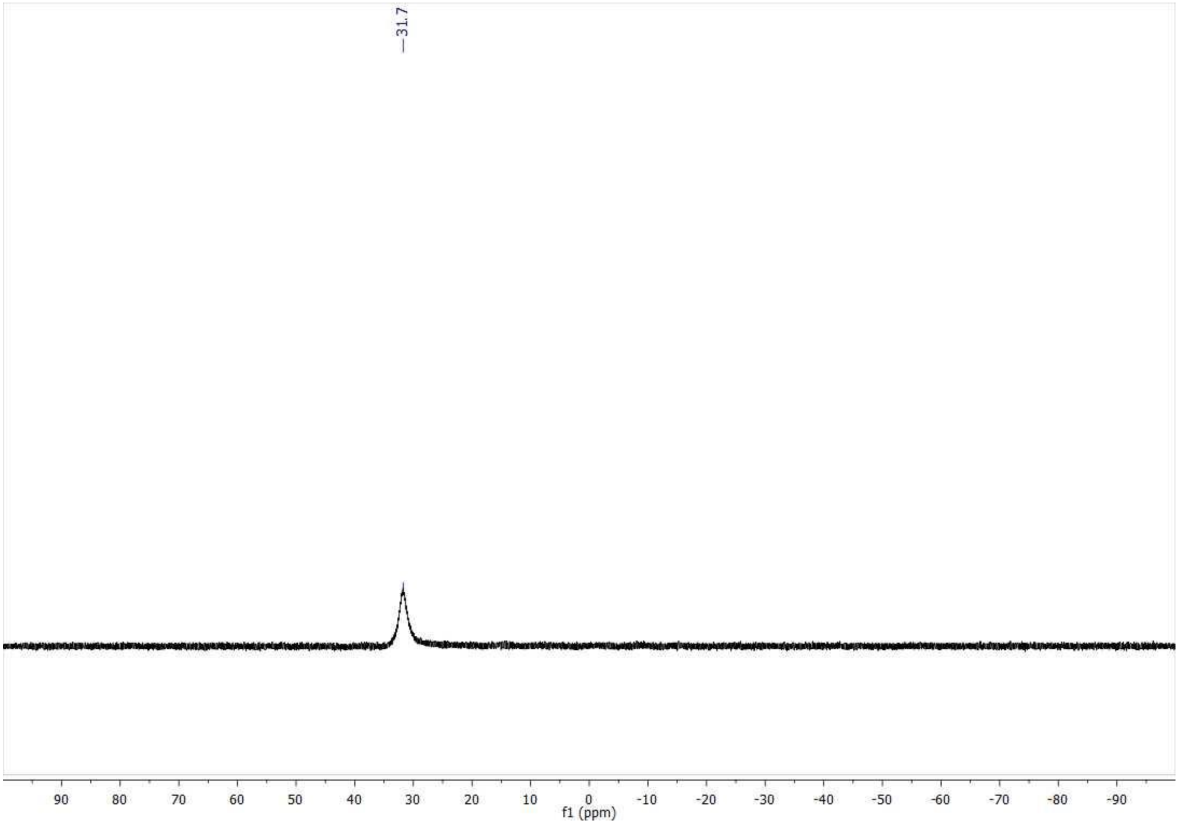

2-((1*S*,2*S*,3*R*)-2-(2-Fluorophenyl)-3-(trifluoromethyl)cyclopropyl)-4,4,5,5-tetramethyl-1,3,2-dioxoborolane (2b)

^{1}H NMR (300 MHz, $CDCl_3$)

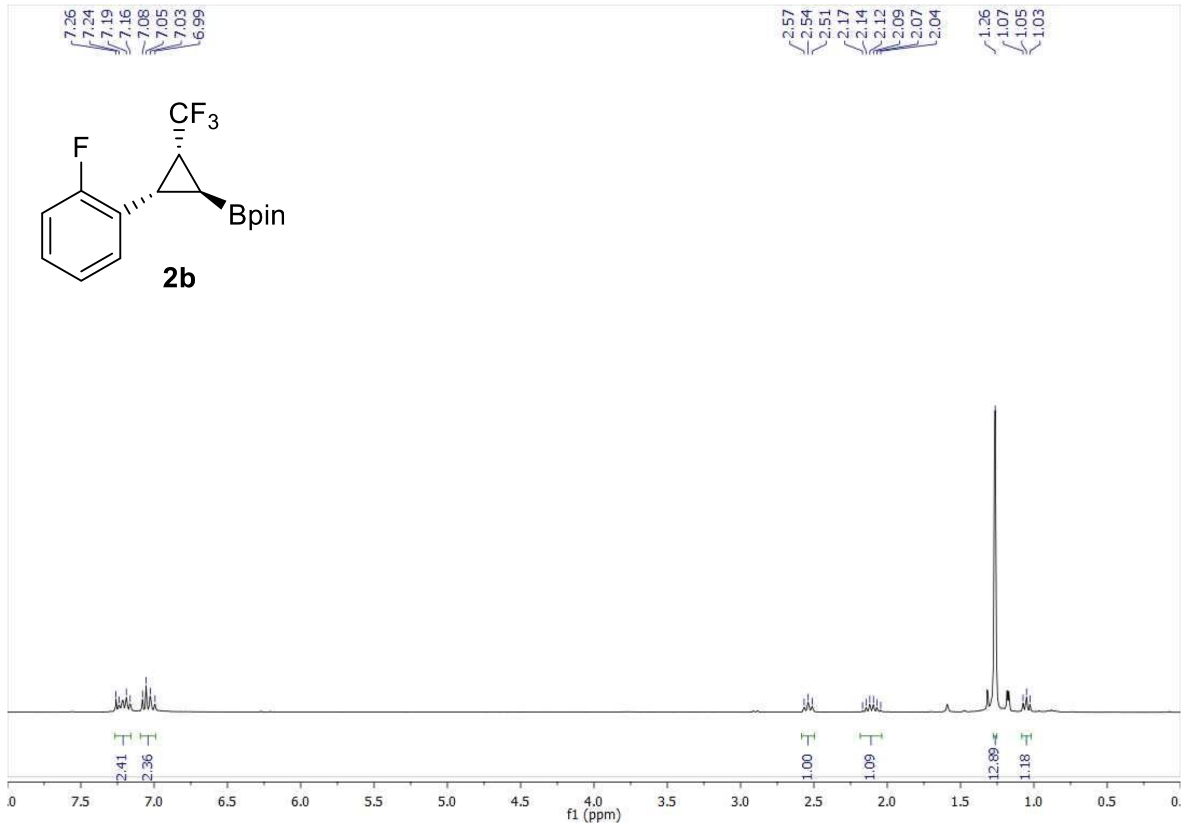

^{13}C NMR (75 MHz, $CDCl_3$)

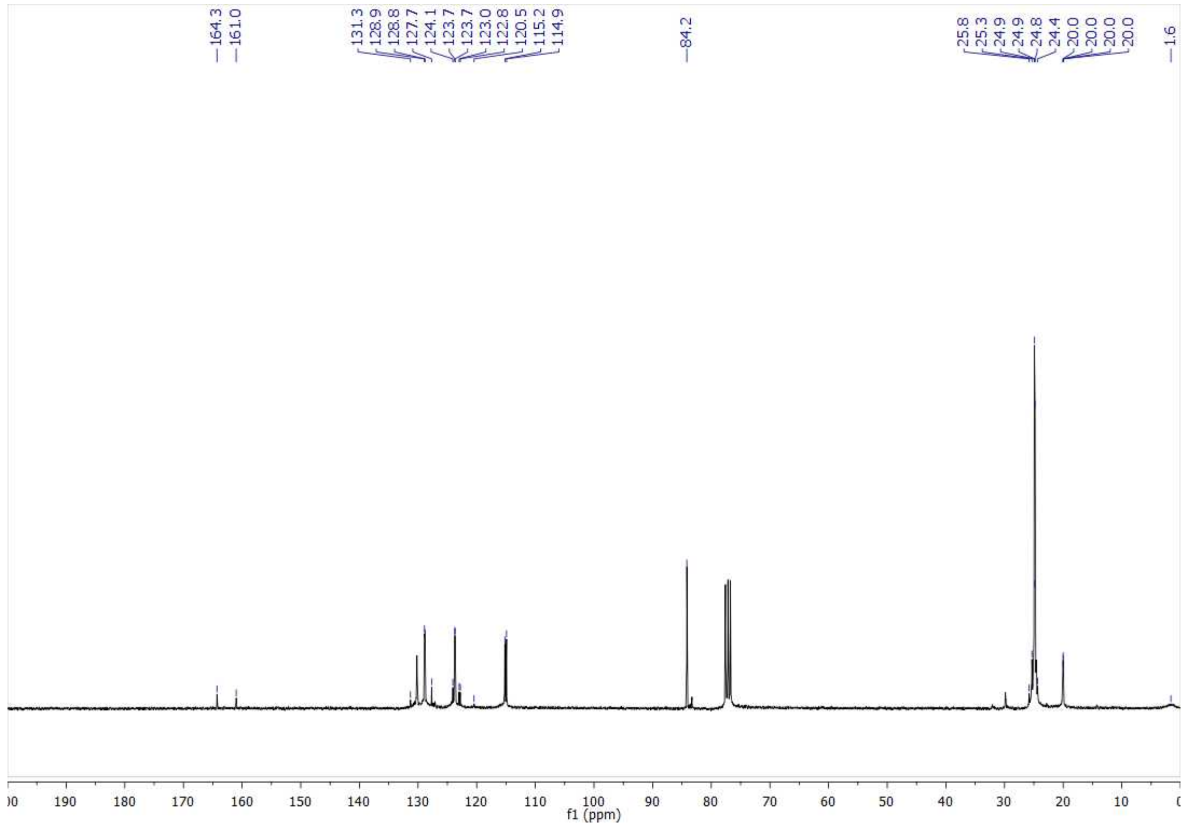

^{19}F NMR (282 MHz, $CDCl_3$)

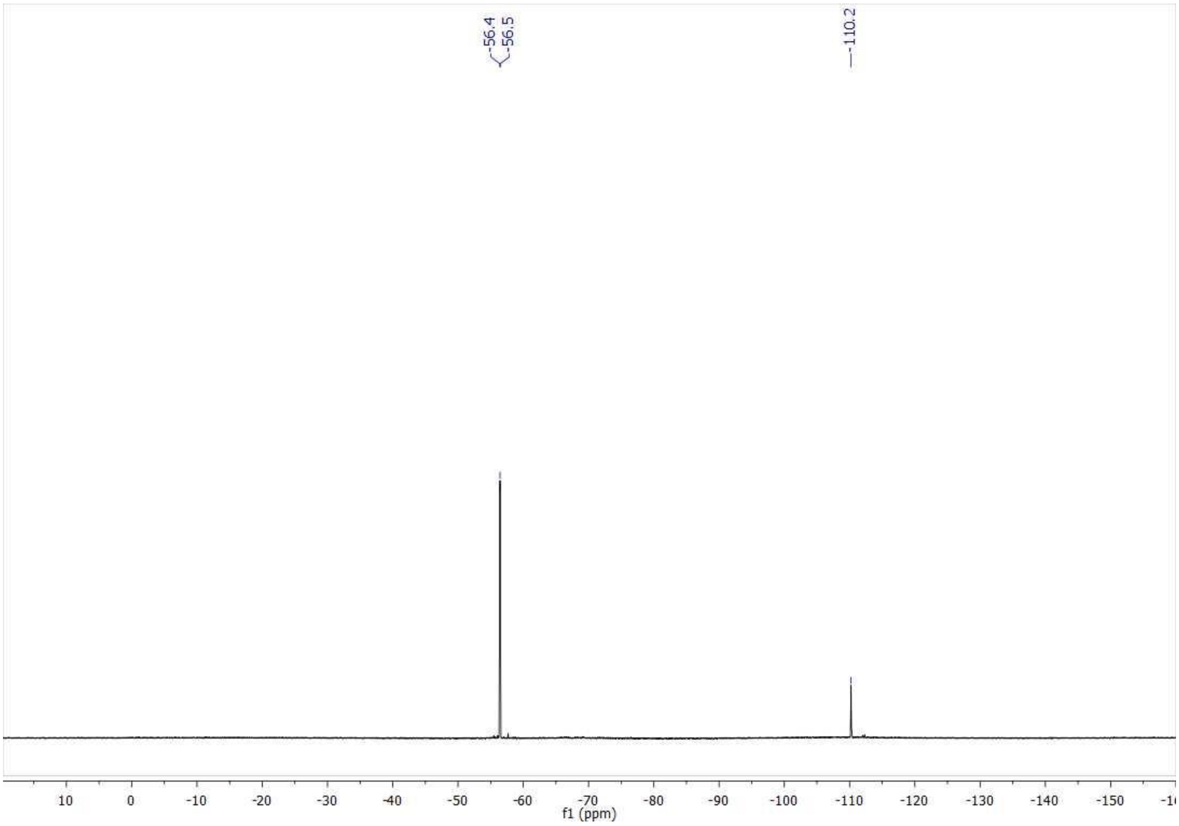

^{11}B NMR (160 MHz, $CDCl_3$)

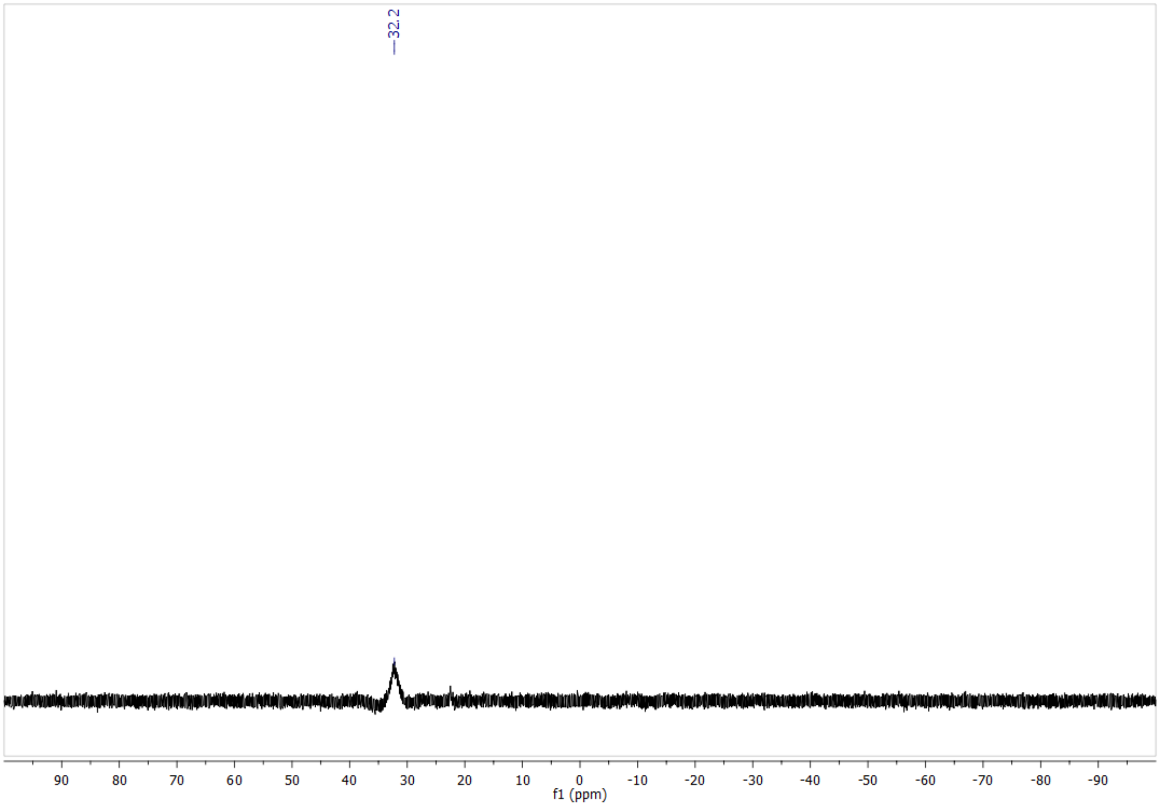

2-((1*S*,2*S*,3*R*)-2-(*o*-Tolyl)-3-(trifluoromethyl)cyclopropyl)-4,4,5,5-tetramethyl-1,3,2-dioxoborolane (2c)

^{1}H NMR (300 MHz, $CDCl_3$)

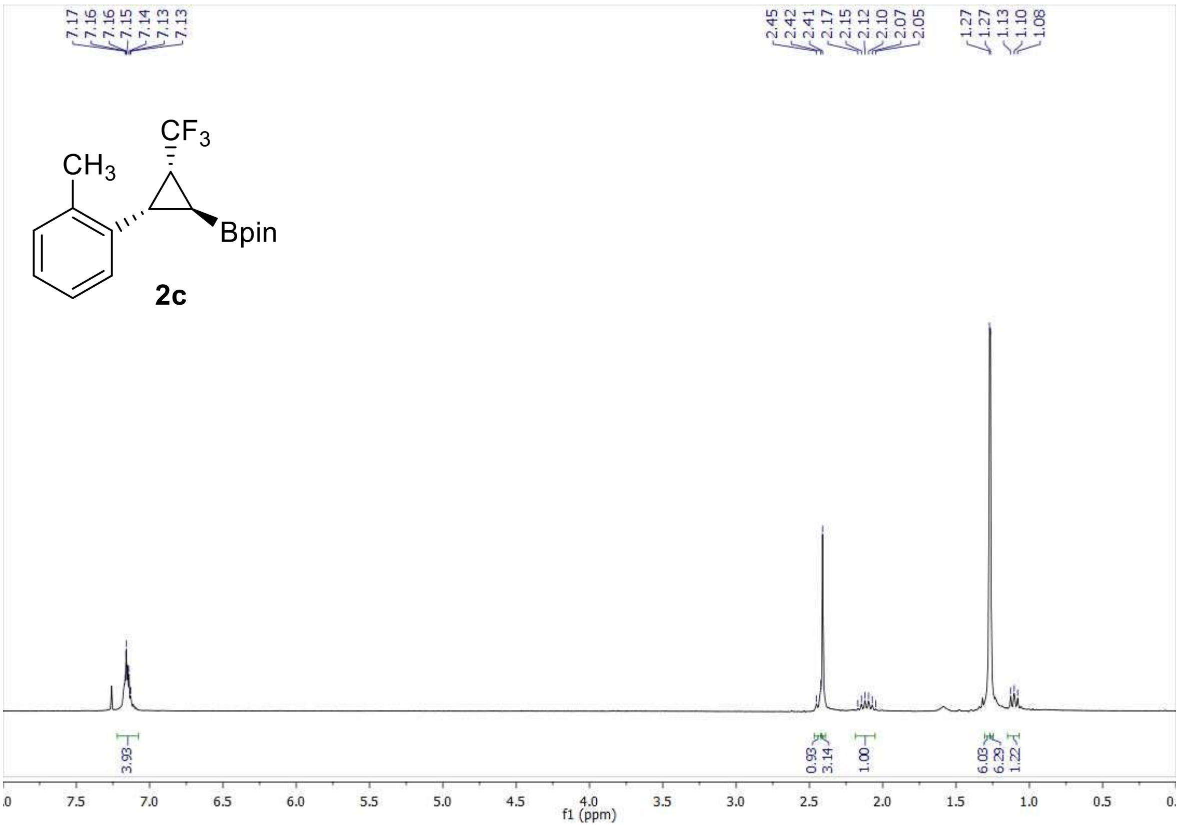

^{13}C NMR (101 MHz, $CDCl_3$)

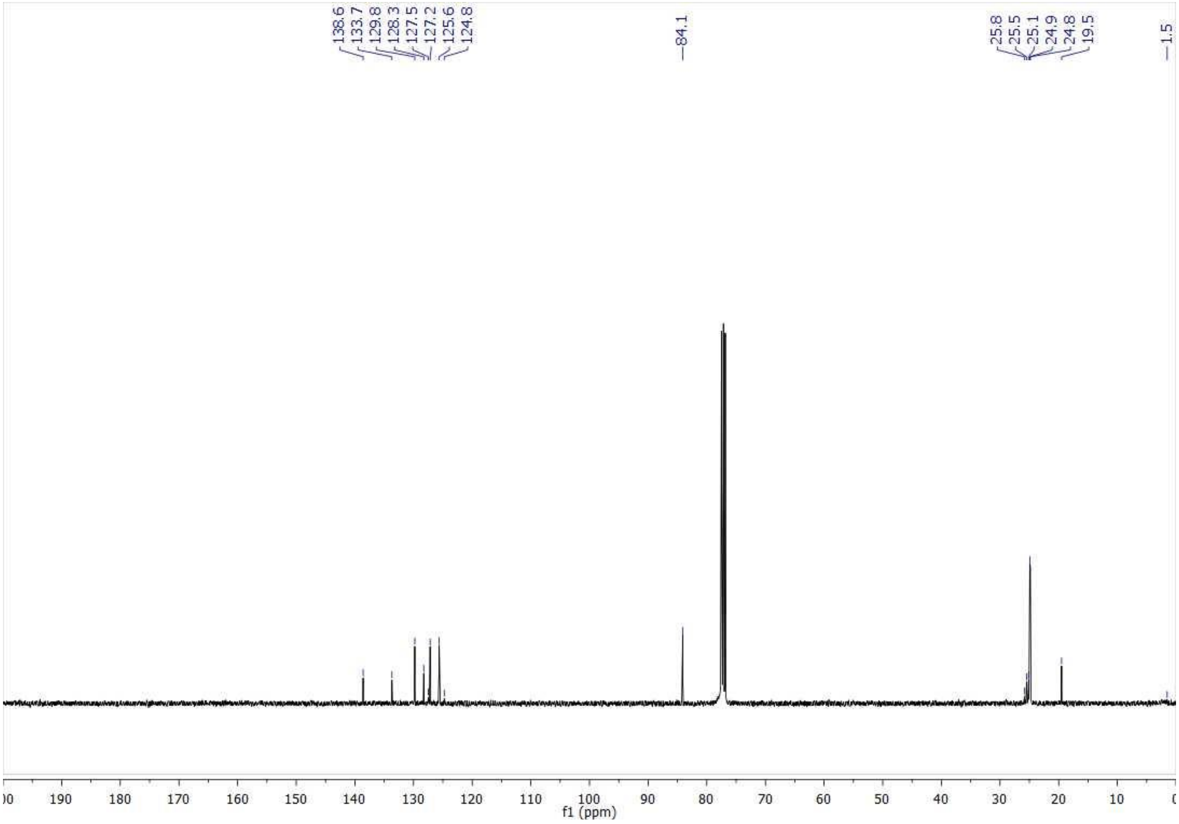

^{19}F NMR (376 MHz, $CDCl_3$)

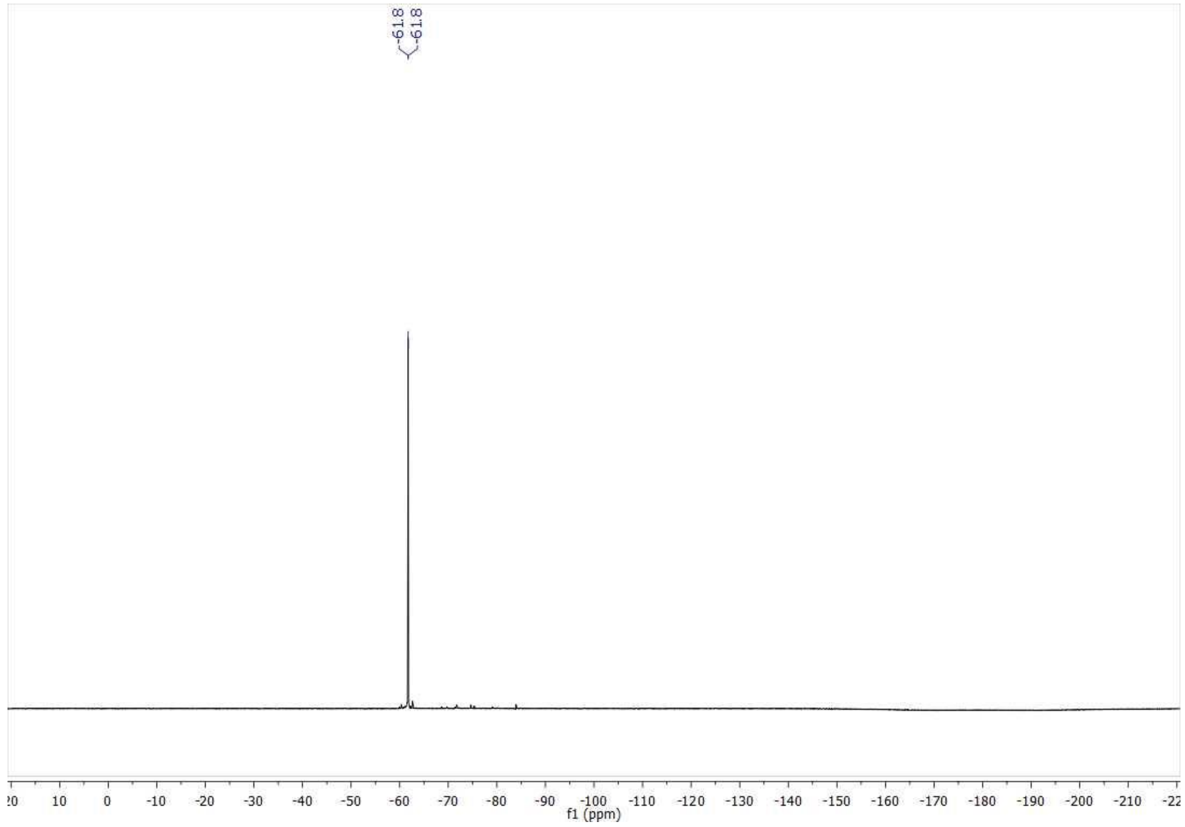

^{11}B NMR (160 MHz, $CDCl_3$)

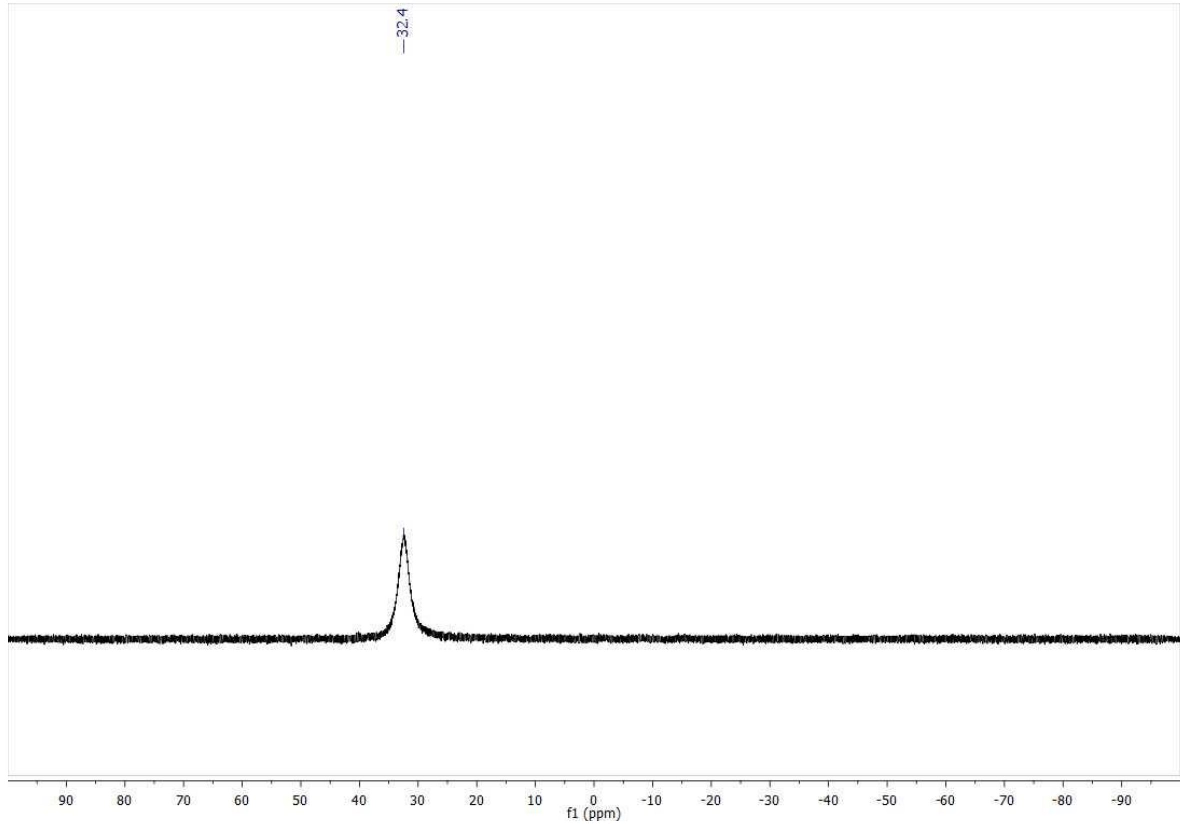

2-((1*S*,2*S*,3*R*)-2-(*m*-Tolyl)-3-(trifluoromethyl)cyclopropyl)-4,4,5,5-tetramethyl-1,3,2-dioxoborolane (2d)

^{1}H NMR (300 MHz, $CDCl_3$)

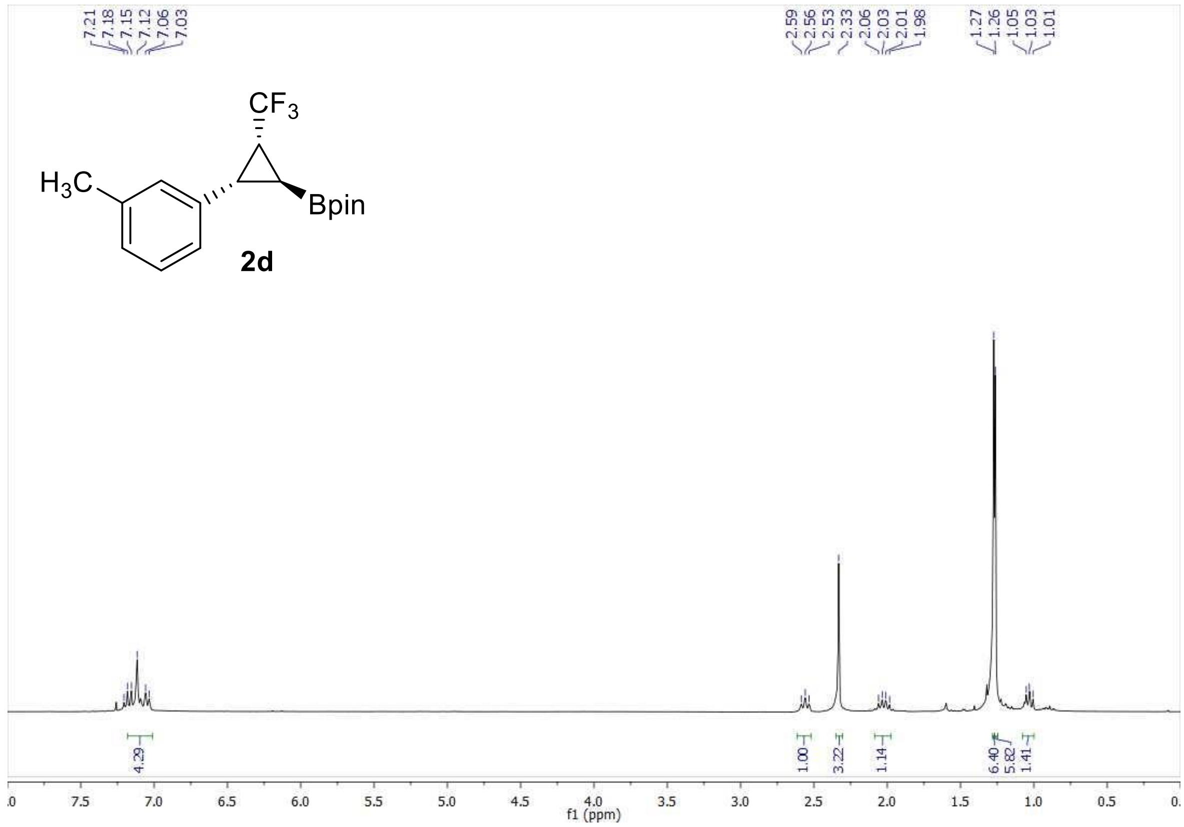

^{13}C NMR (75 MHz, $CDCl_3$)

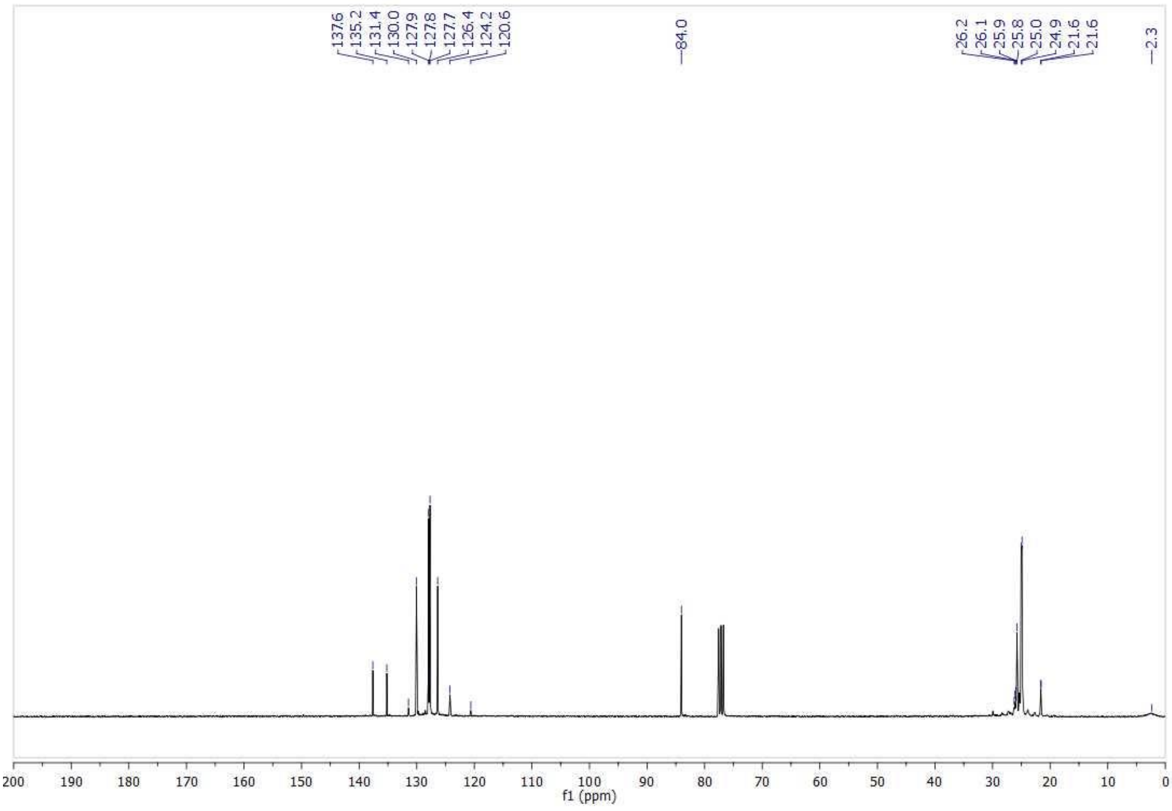

^{19}F NMR (282 MHz, $CDCl_3$)

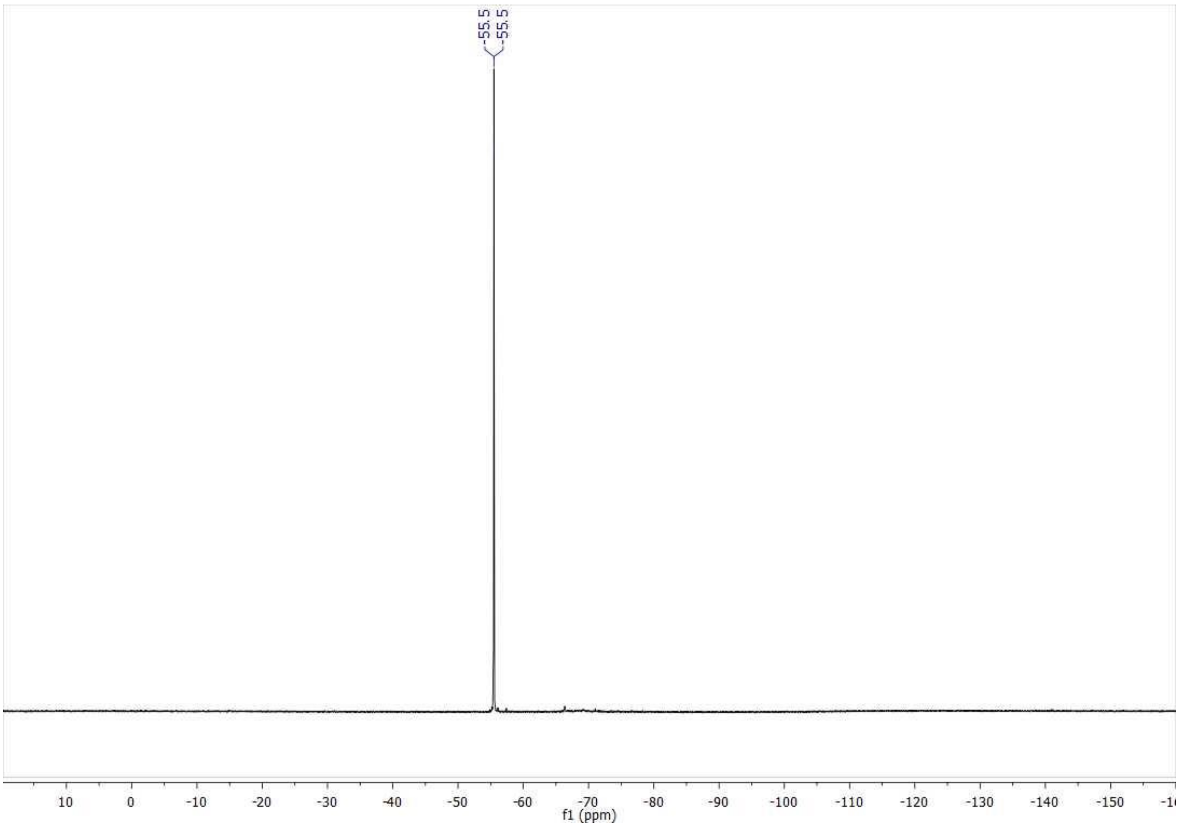

^{11}B NMR (160 MHz, $CDCl_3$)

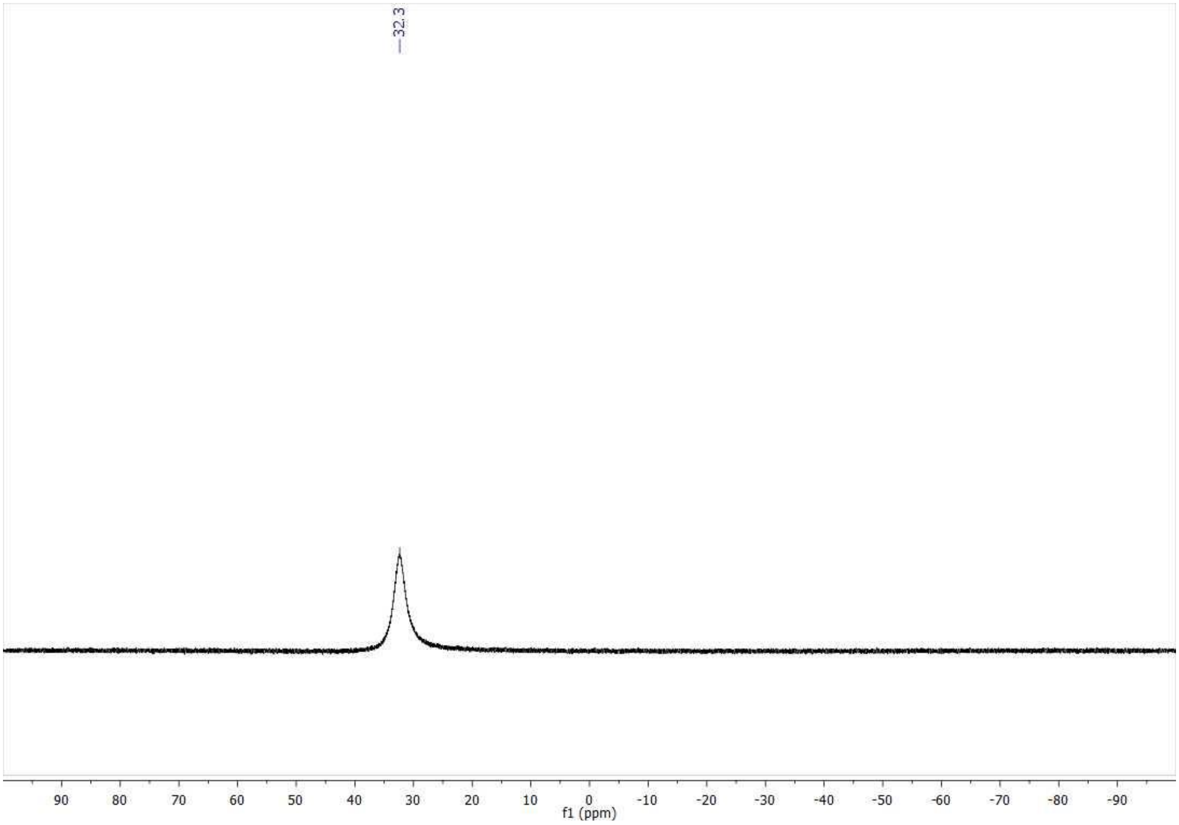

2-((1*S*,2*S*,3*R*)-2-(3-Fluorophenyl)-3-(trifluoromethyl)cyclopropyl)-4,4,5,5-tetramethyl-1,3,2-dioxoborolane (2e)

^{1}H NMR (300 MHz, $CDCl_3$)

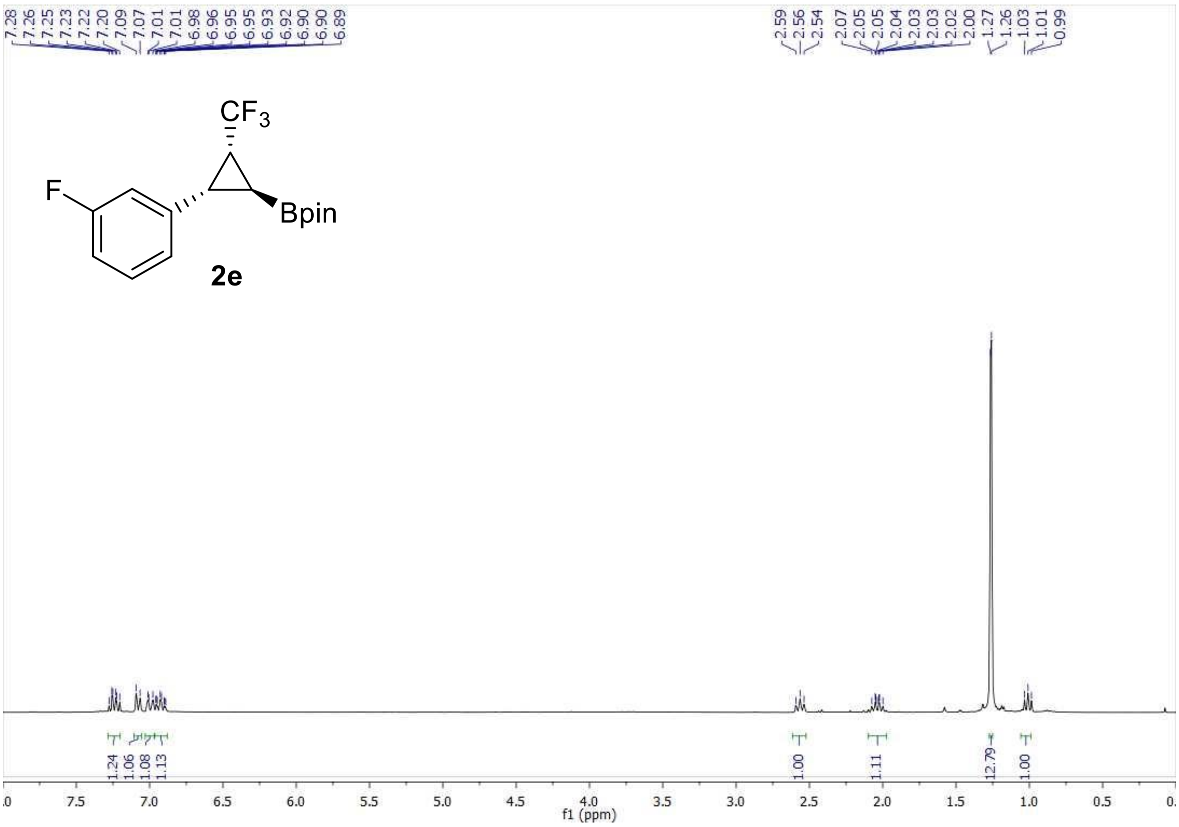

^{13}C NMR (75 MHz, $CDCl_3$)

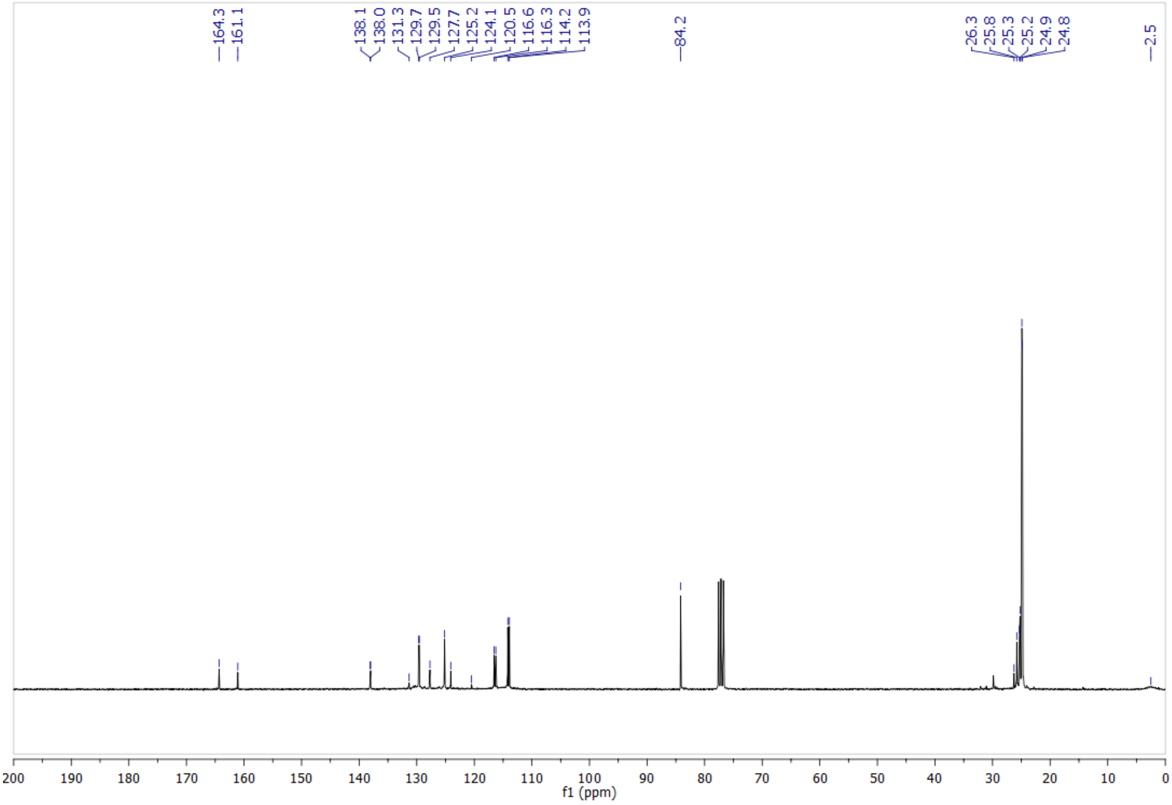

^{19}F NMR (282 MHz, $CDCl_3$)

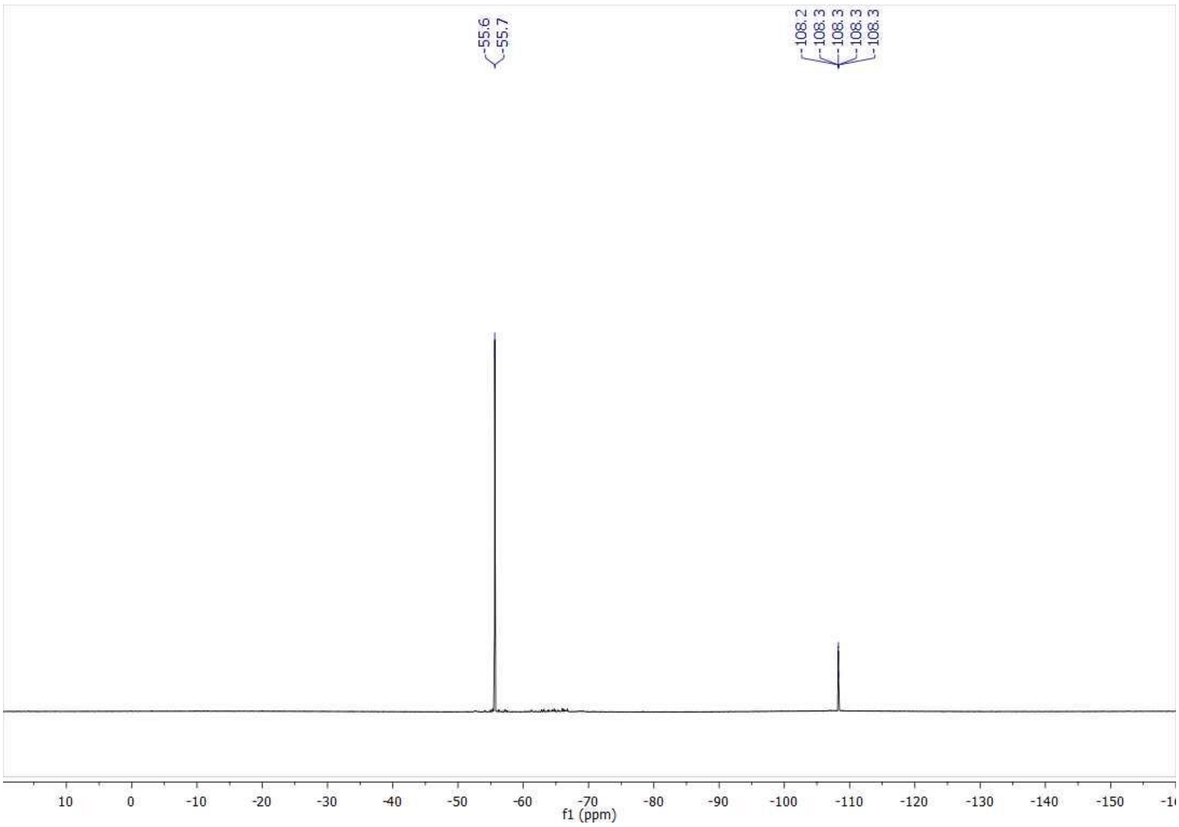

^{11}B NMR (160 MHz, $CDCl_3$)

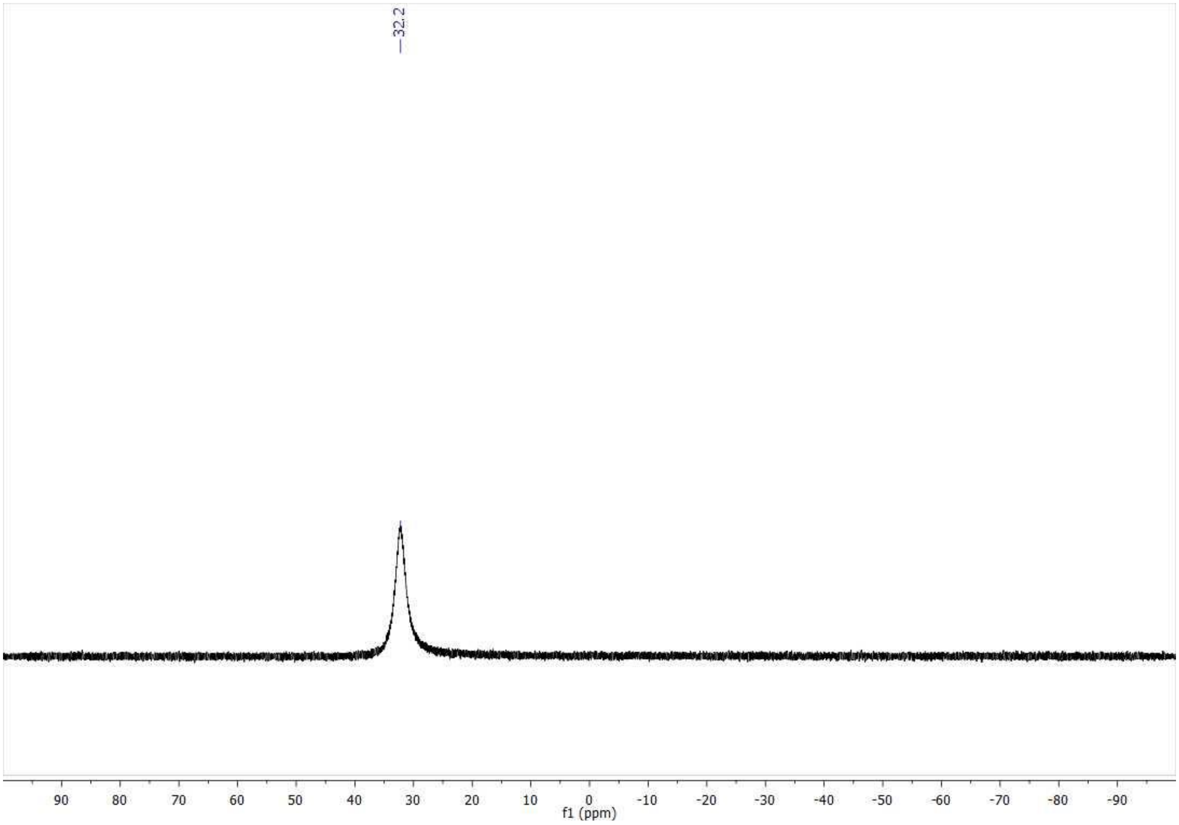

3-((1*S*,2*S*,3*R*)-2--4,4,5,5-Tetramethyl-1,3,2-dioxoborolane-3-(trifluoromethyl)cyclopropyl) phenyl acetate (2f)

^{1}H NMR (300 MHz, $CDCl_3$)

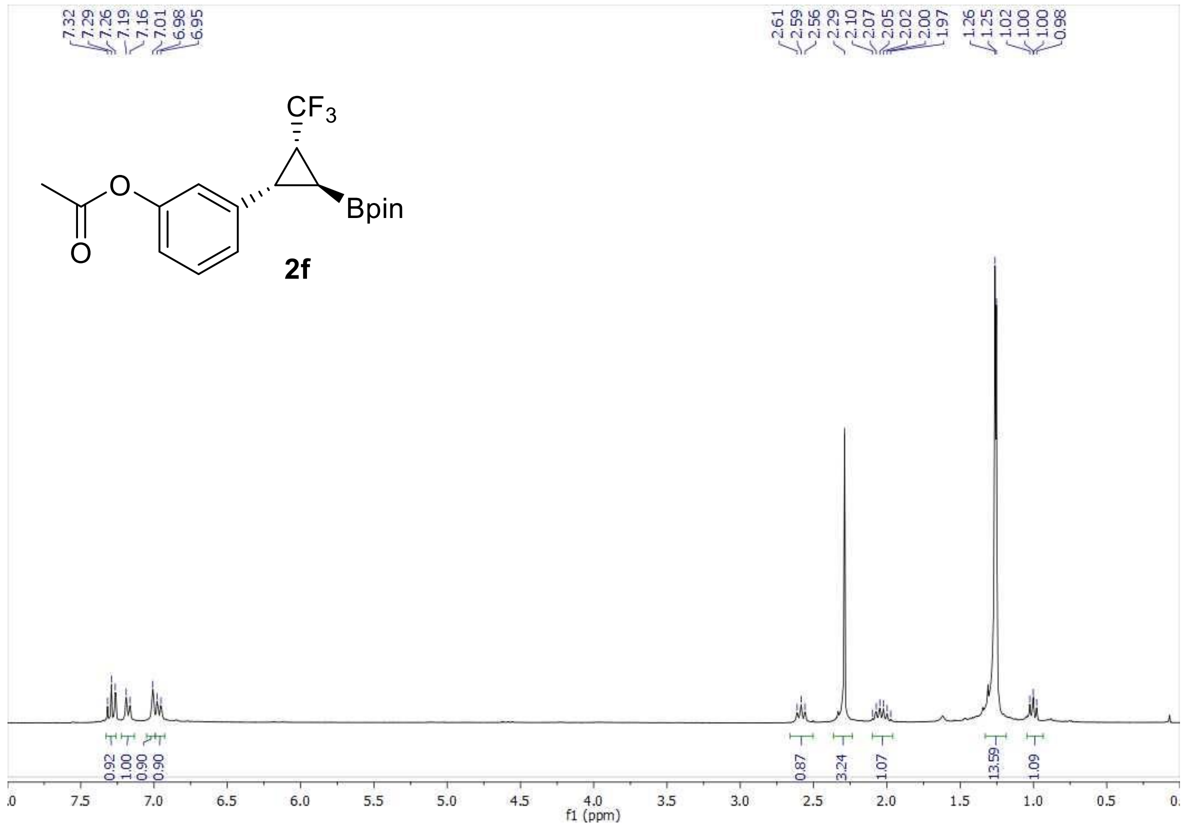

^{13}C NMR (126 MHz, $CDCl_3$)

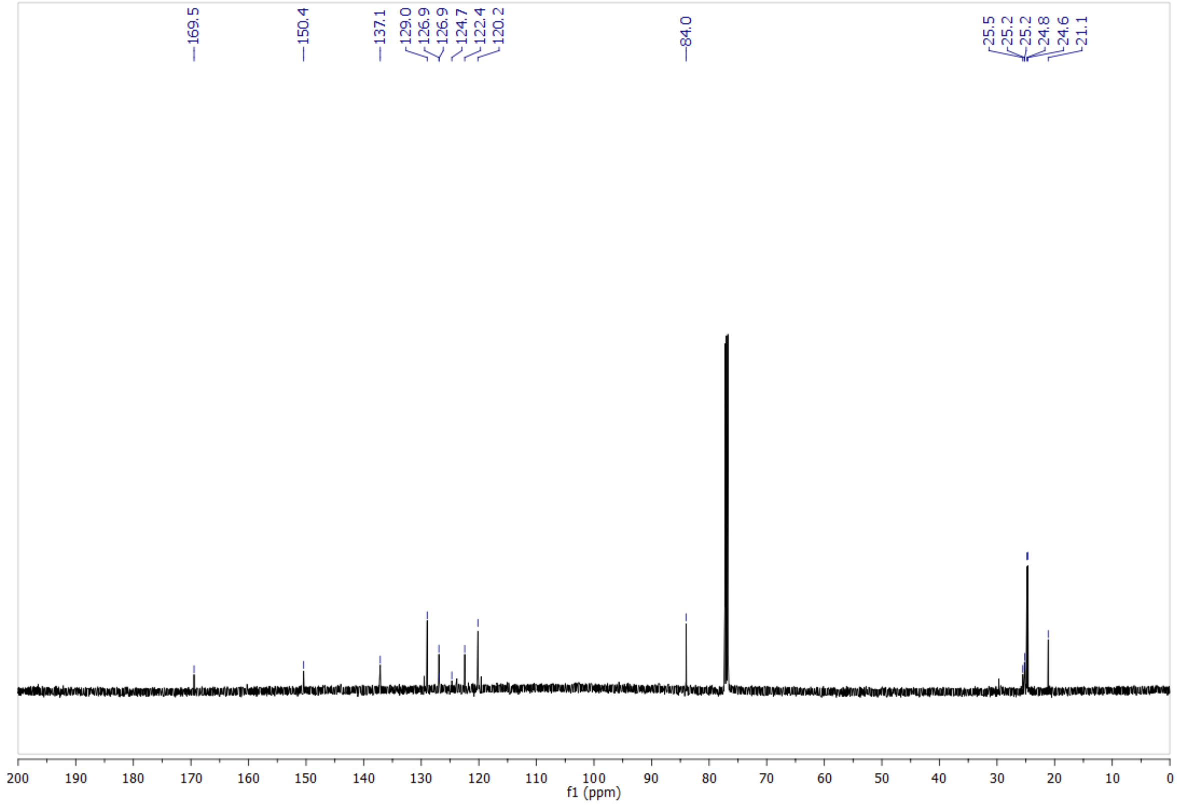

^{19}F NMR (282 MHz, $CDCl_3$)

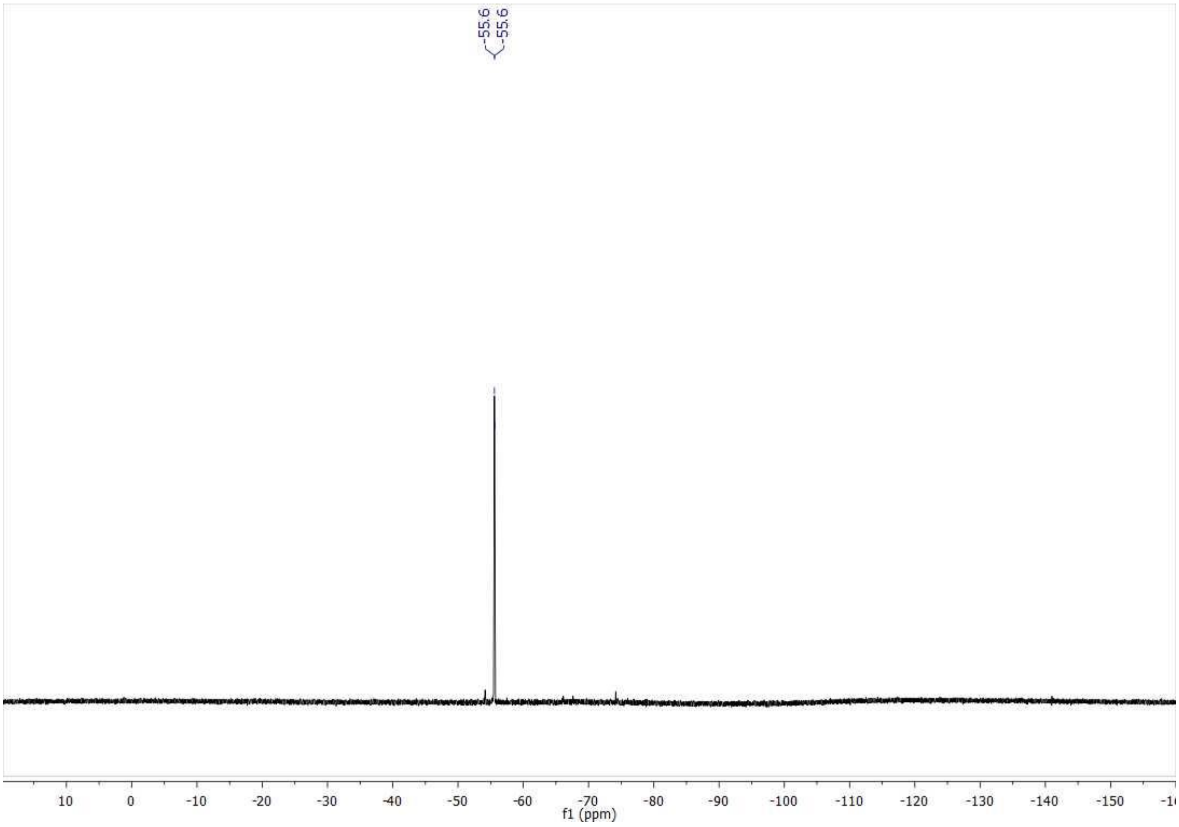

^{11}B NMR (160 MHz, $CDCl_3$)

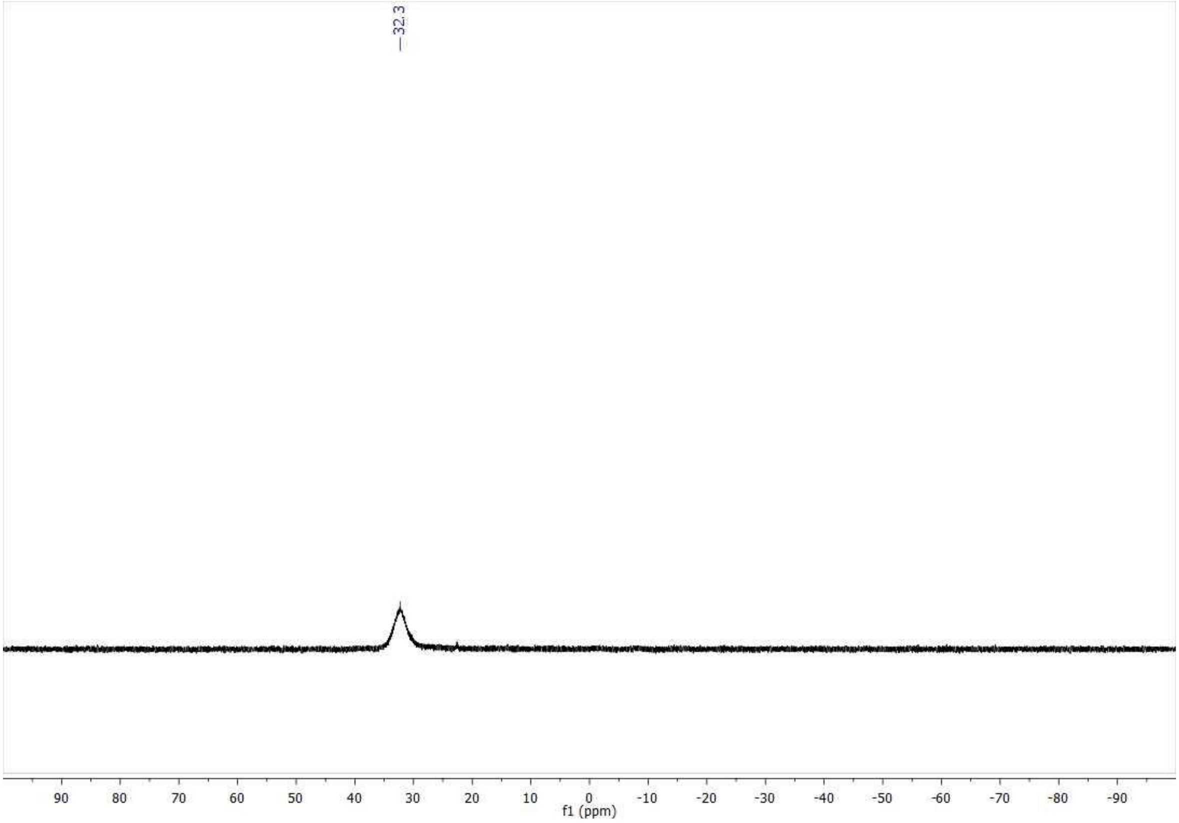

2-((1*S*,2*S*,3*R*)-2-(*p*-Tolyl)-3-(trifluoromethyl)cyclopropyl)-4,4,5,5-tetramethyl-1,3,2-dioxoborolane (2g)

^{1}H NMR (300 MHz, $CDCl_3$)

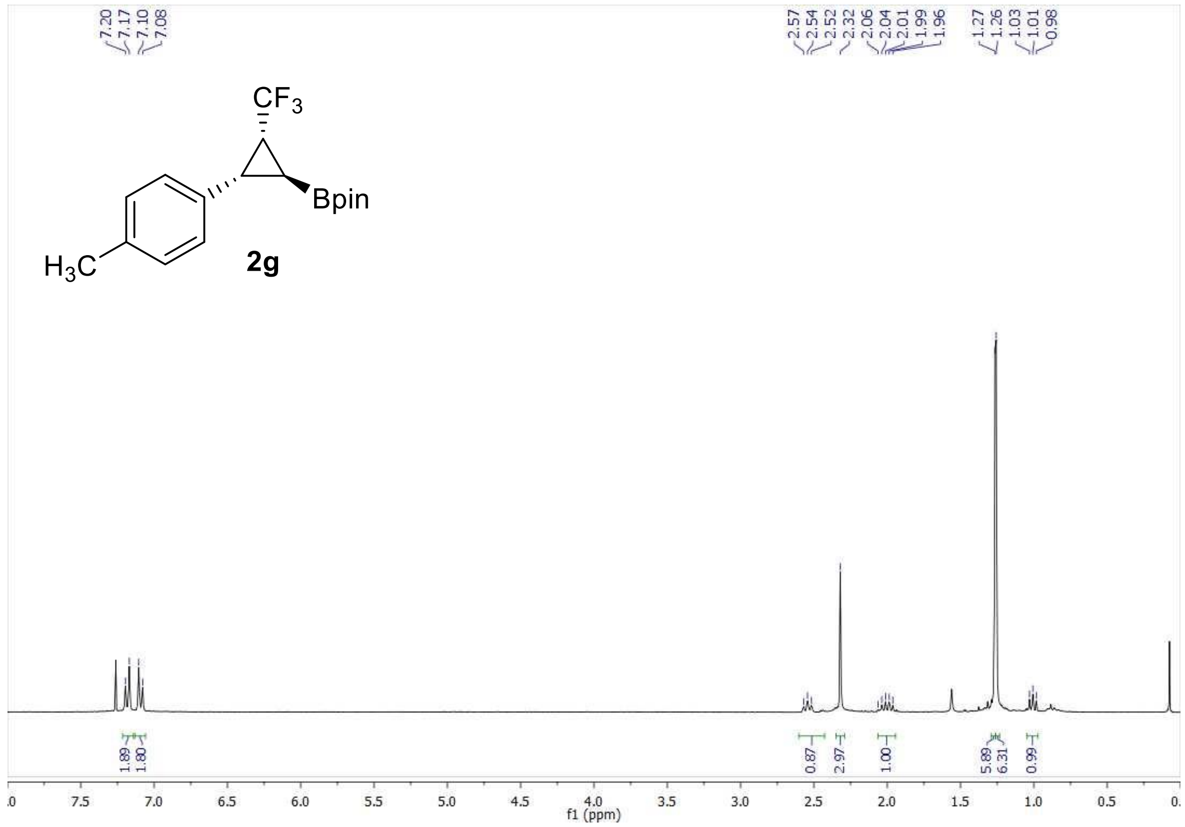

^{13}C NMR (126 MHz, $CDCl_3$)

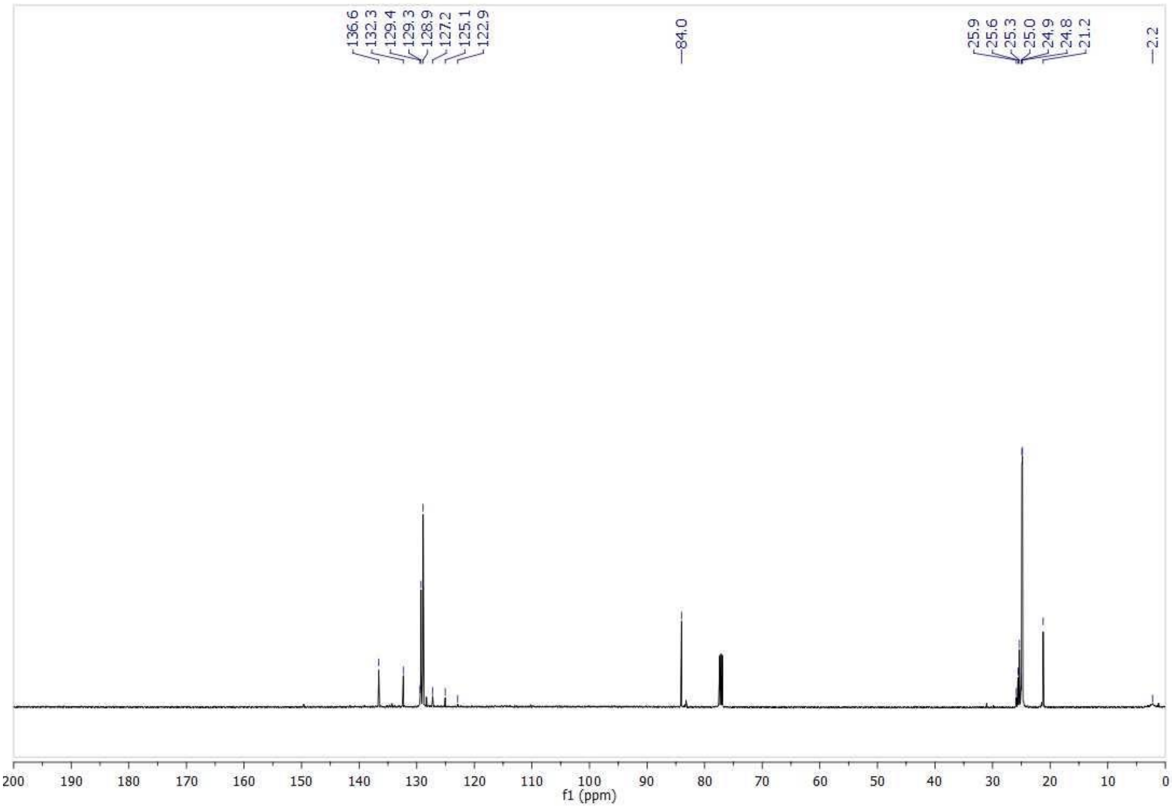

^{19}F NMR (282 MHz, $CDCl_3$)

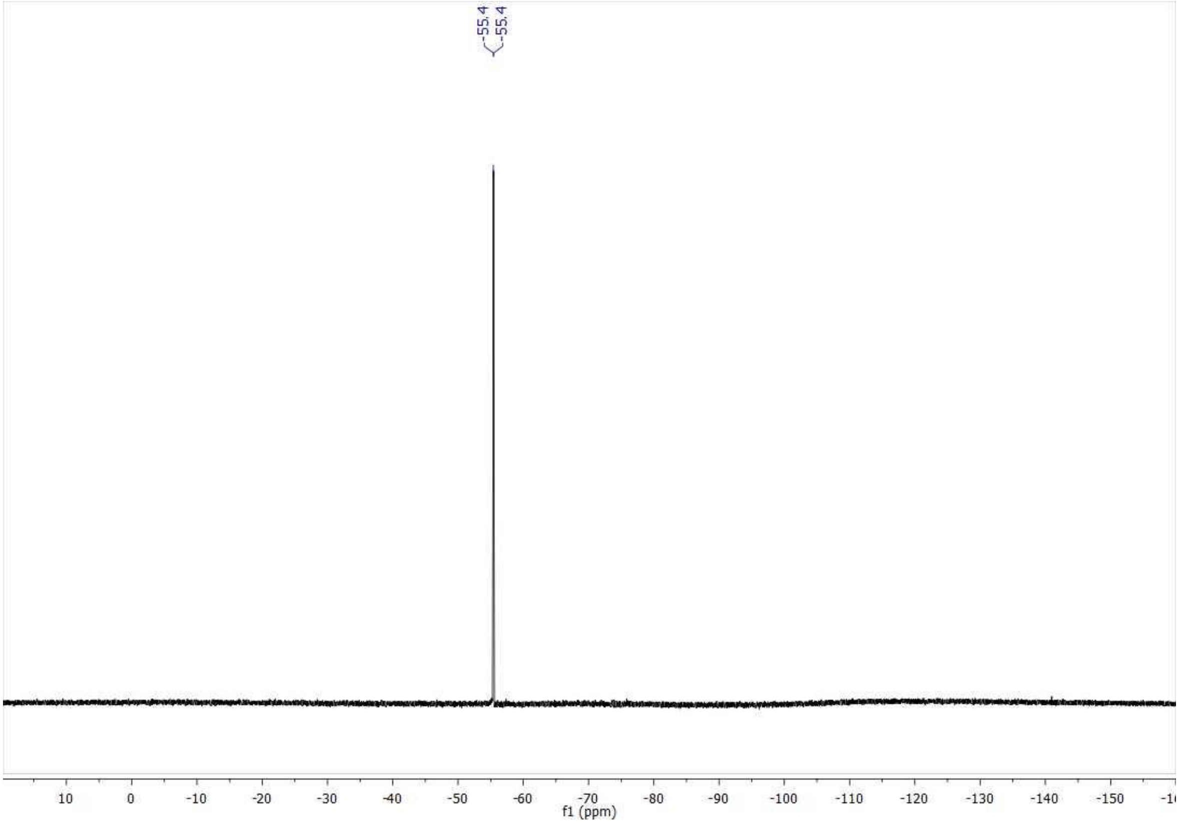

^{11}B NMR (160 MHz, $CDCl_3$)

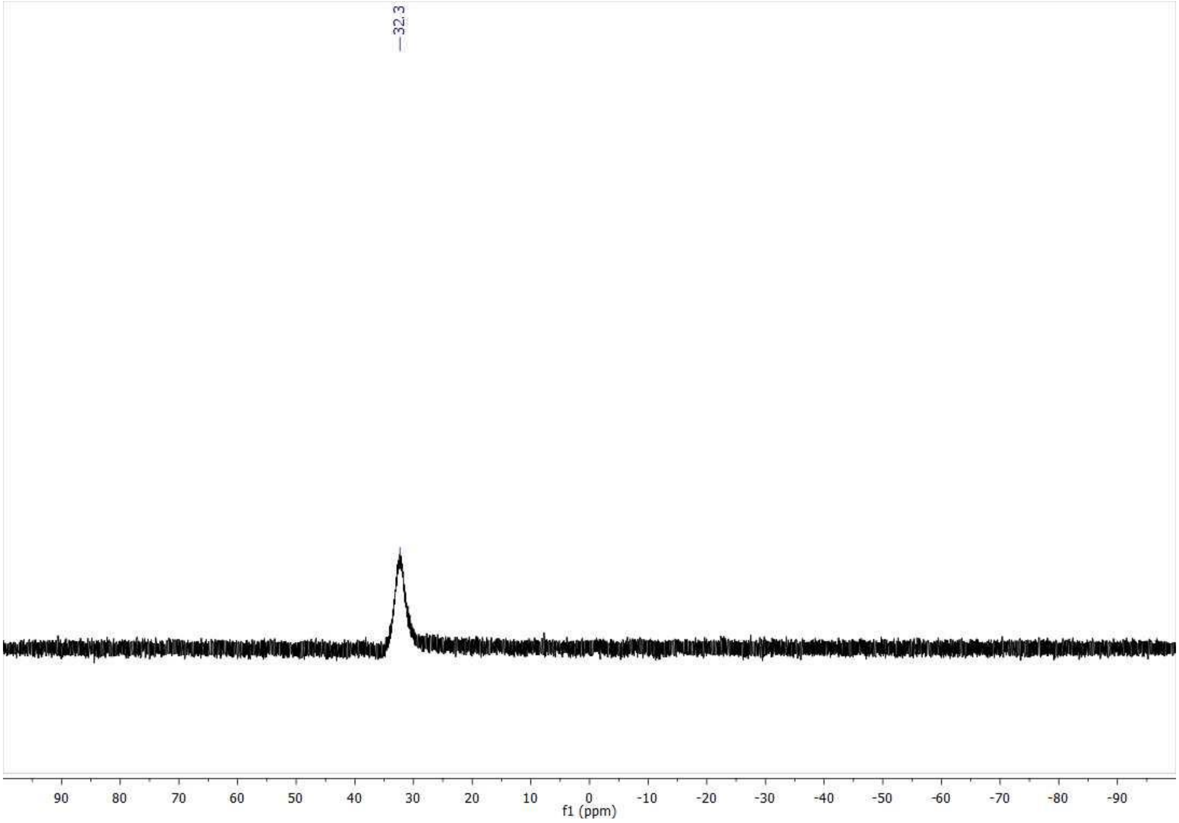

2-((1*S*,2*S*,3*R*)-2-(*p*-Chlorophenyl)-3-(trifluoromethyl)cyclopropyl)-4,4,5,5-tetramethyl-1,3,2-dioxoborolane (2h)

^{1}H NMR (500 MHz, $CDCl_3$)

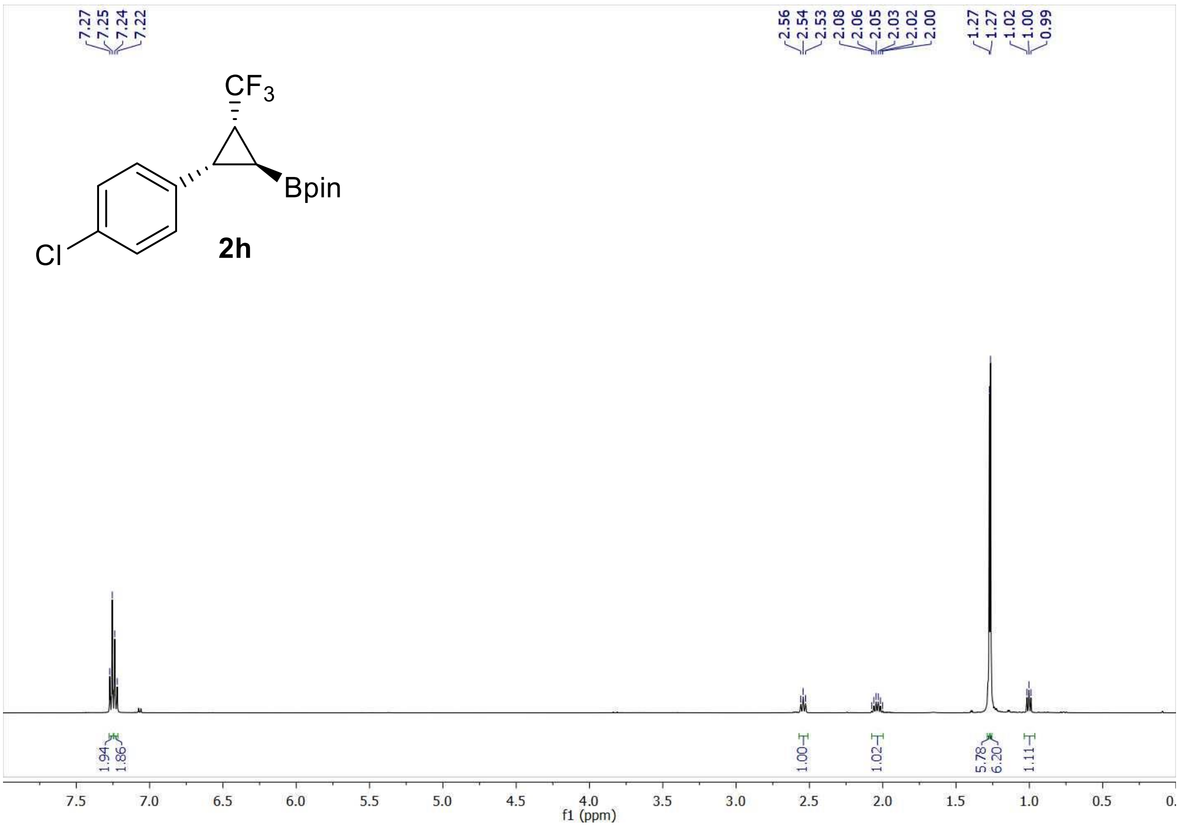

^{13}C NMR (126 MHz, $CDCl_3$)

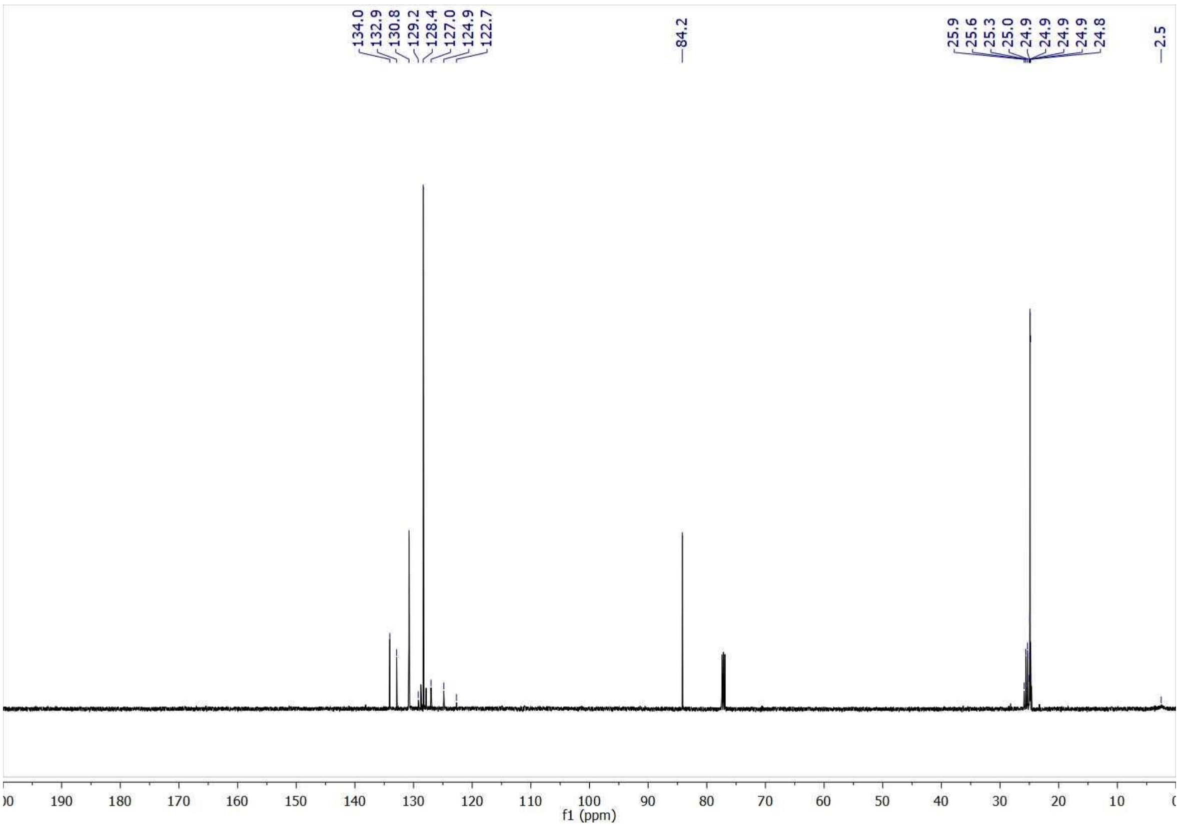

^{19}F NMR (282 MHz, $CDCl_3$)

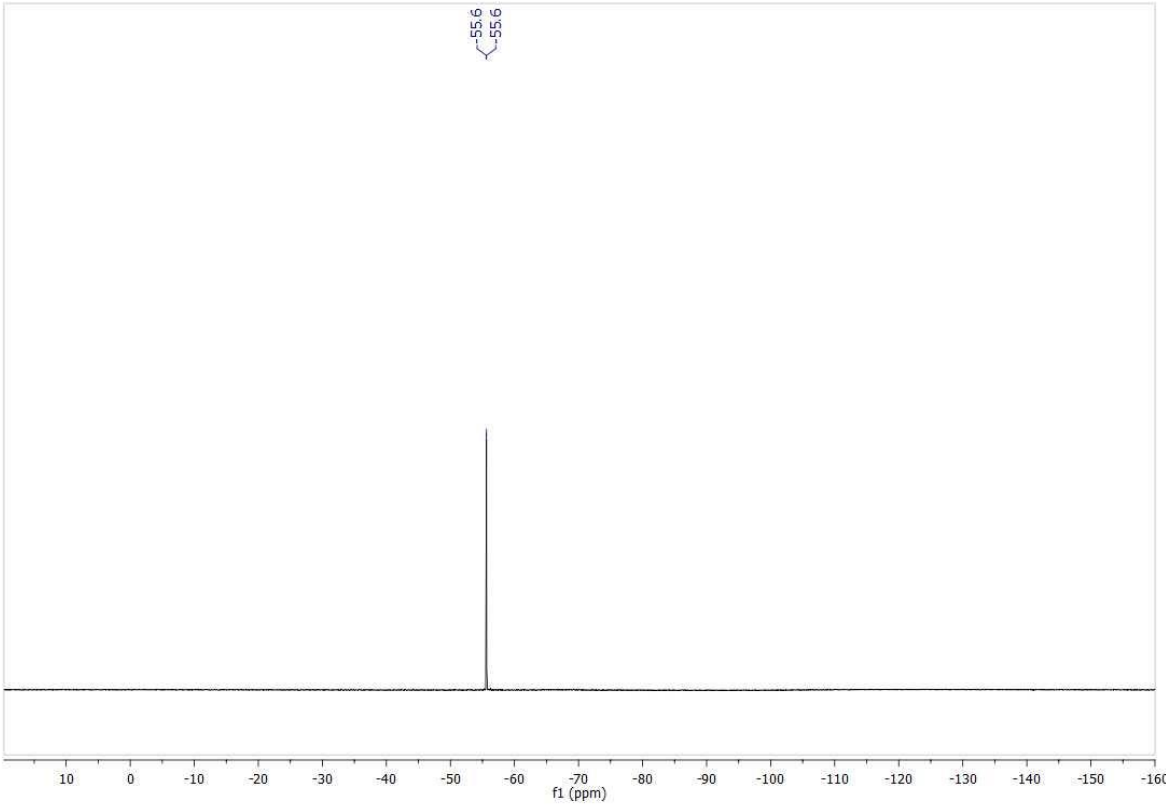

^{11}B NMR (160 MHz, $CDCl_3$)

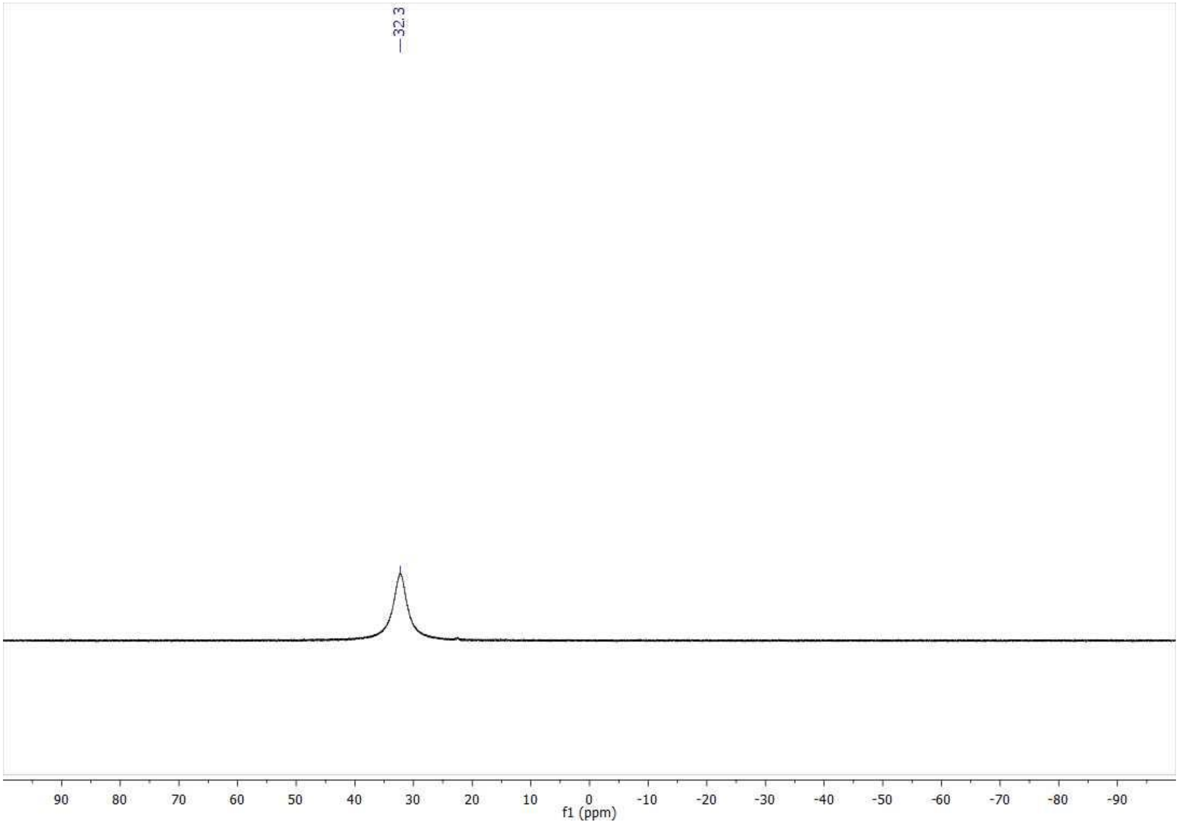

2-((1*S*,2*S*,3*R*)-2-(*p*-Bromophenyl)-3-(trifluoromethyl)cyclopropyl)-4,4,5,5-tetramethyl-1,3,2-dioxoborolane (2i)

^{1}H NMR (300 MHz, $CDCl_3$)

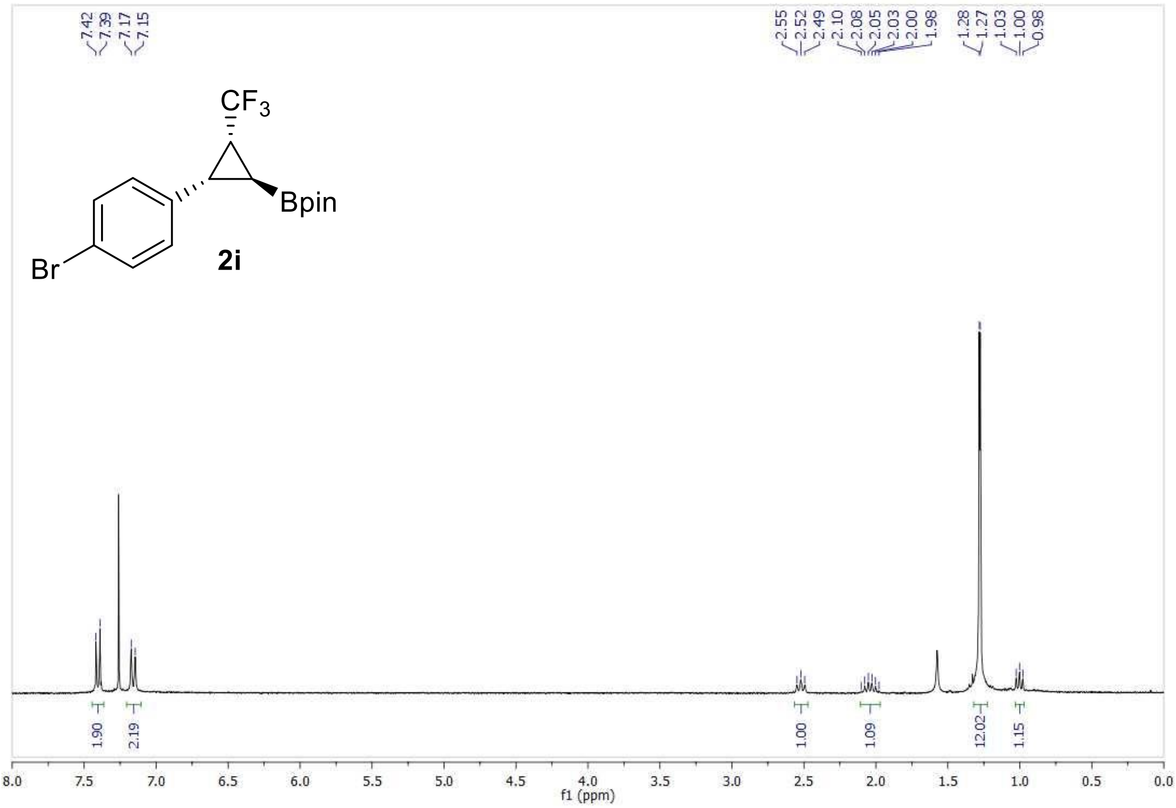

^{13}C NMR (126 MHz, $CDCl_3$)

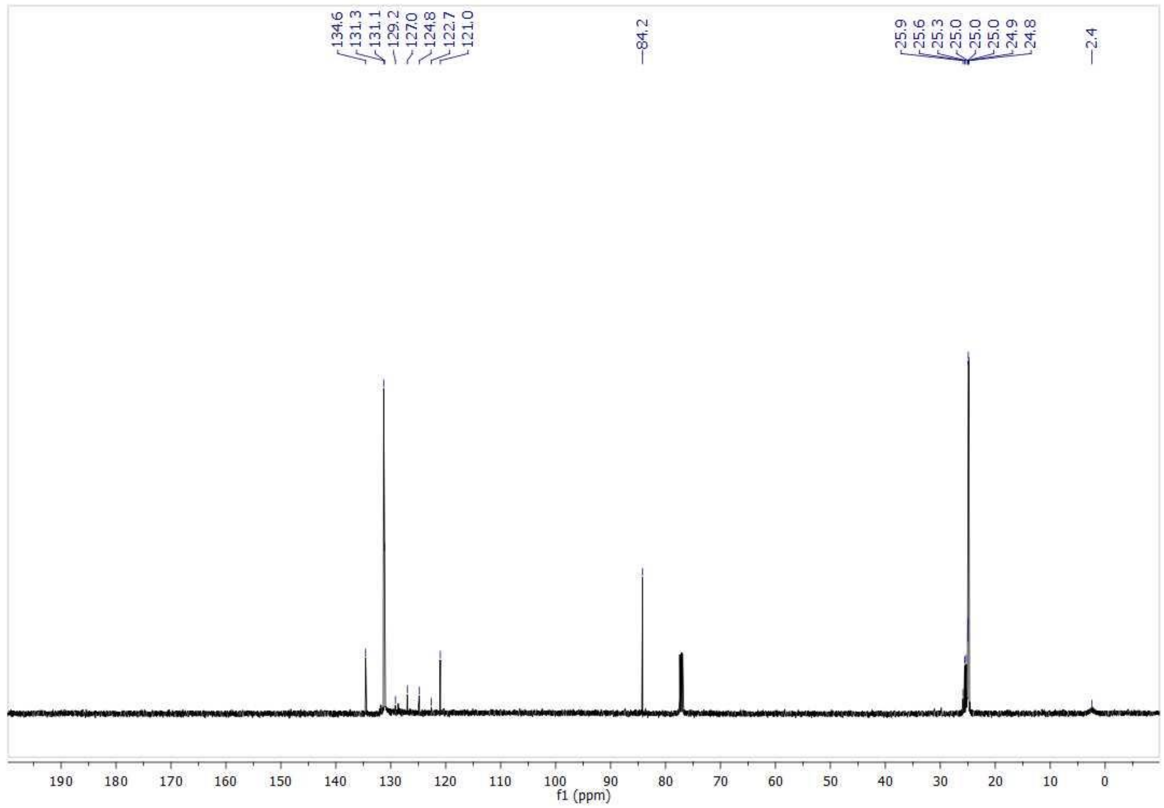

^{19}F NMR (282 MHz, $CDCl_3$)

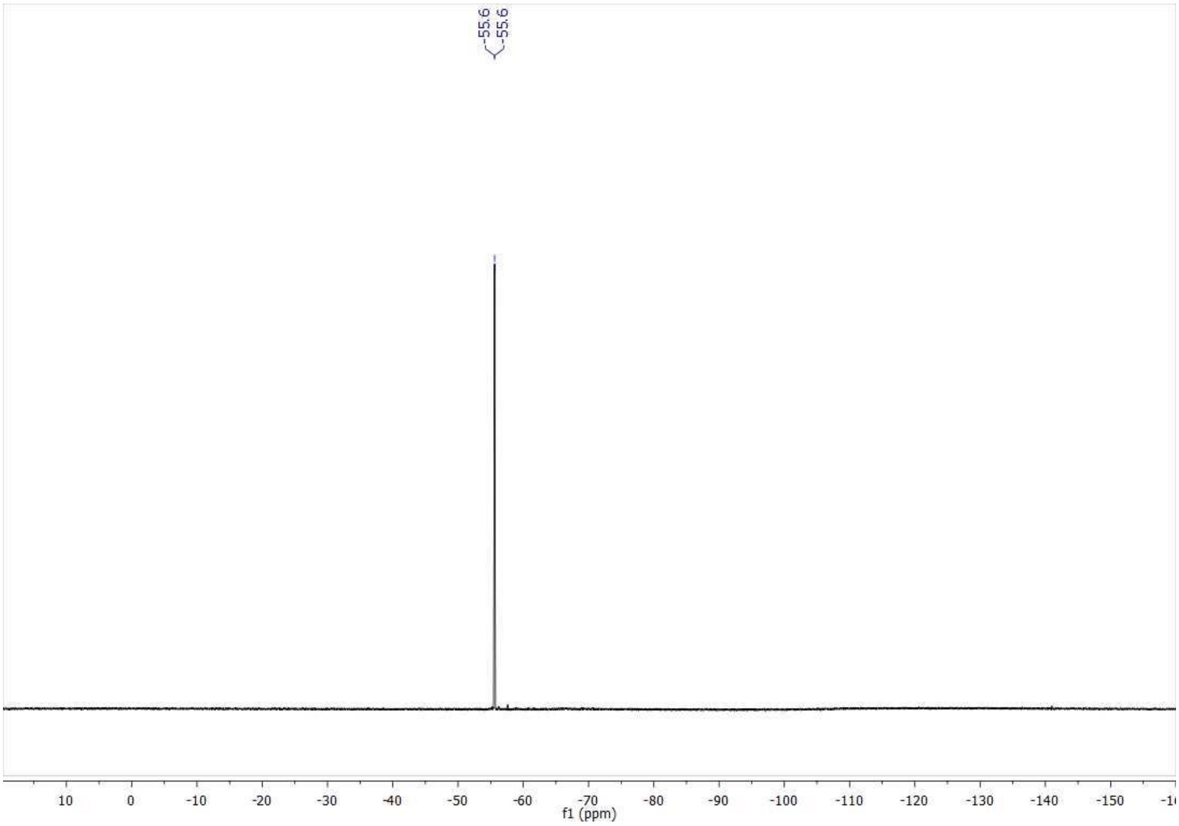

^{11}B NMR (160 MHz, $CDCl_3$)

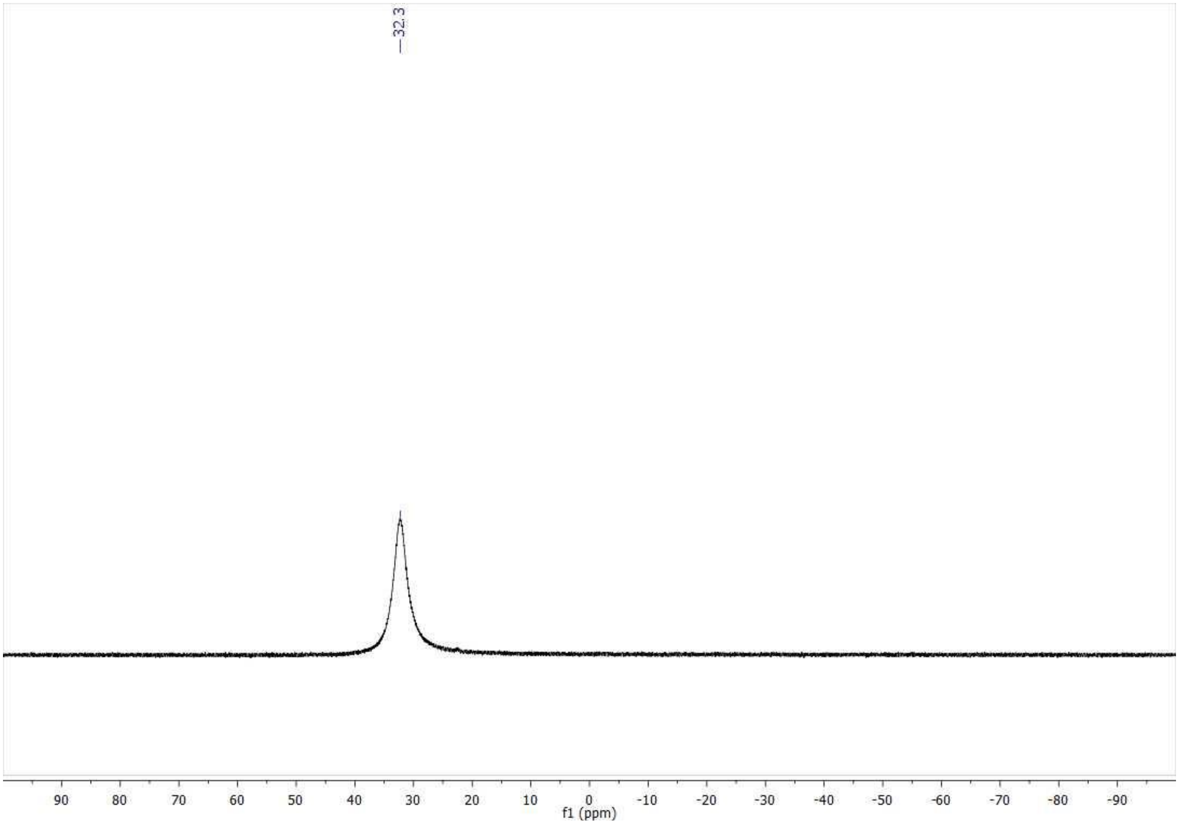

2-((1*S*,2*S*,3*R*)-2-(*p*-Trifluoromethylphenyl)-3-(trifluoromethyl)cyclopropyl)-4,4,5,5-tetramethyl-1,3,2-dioxoborolane (2j)

^{1}H NMR (500 MHz, $CDCl_3$)

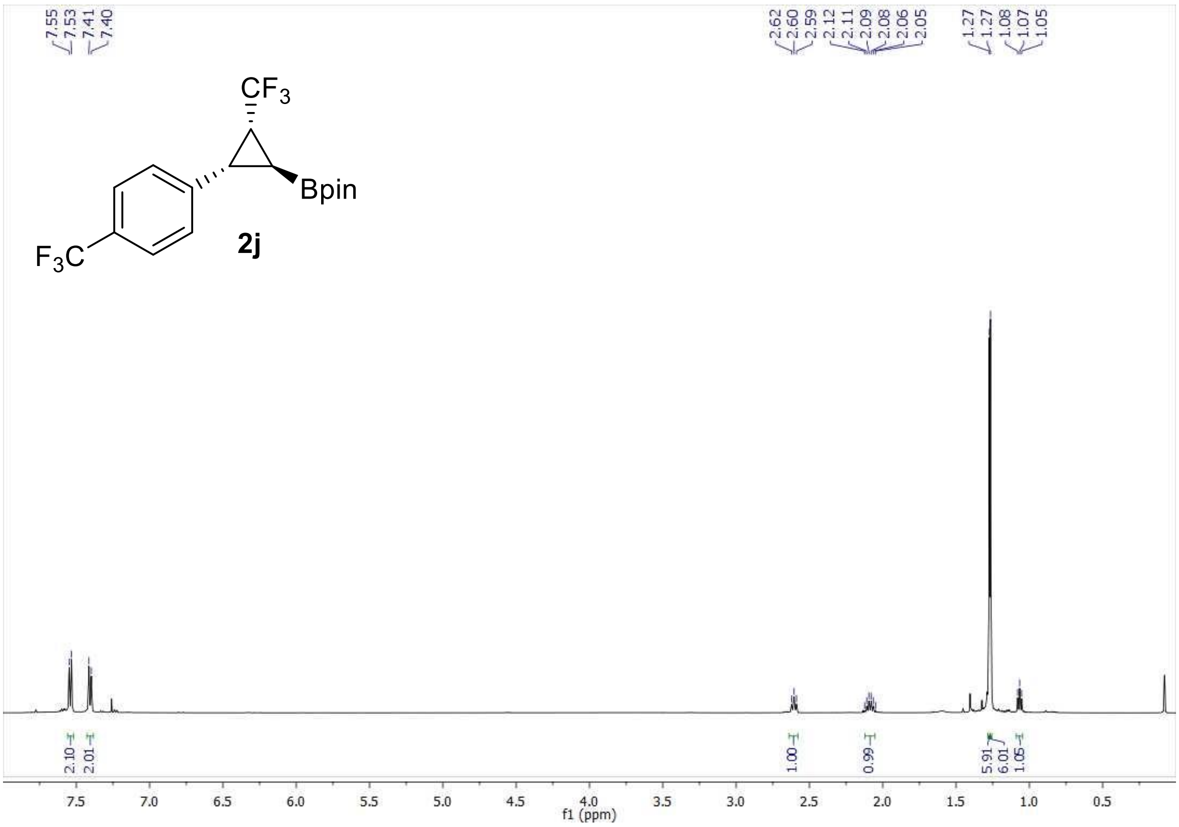

^{13}C NMR (126 MHz, $CDCl_3$)

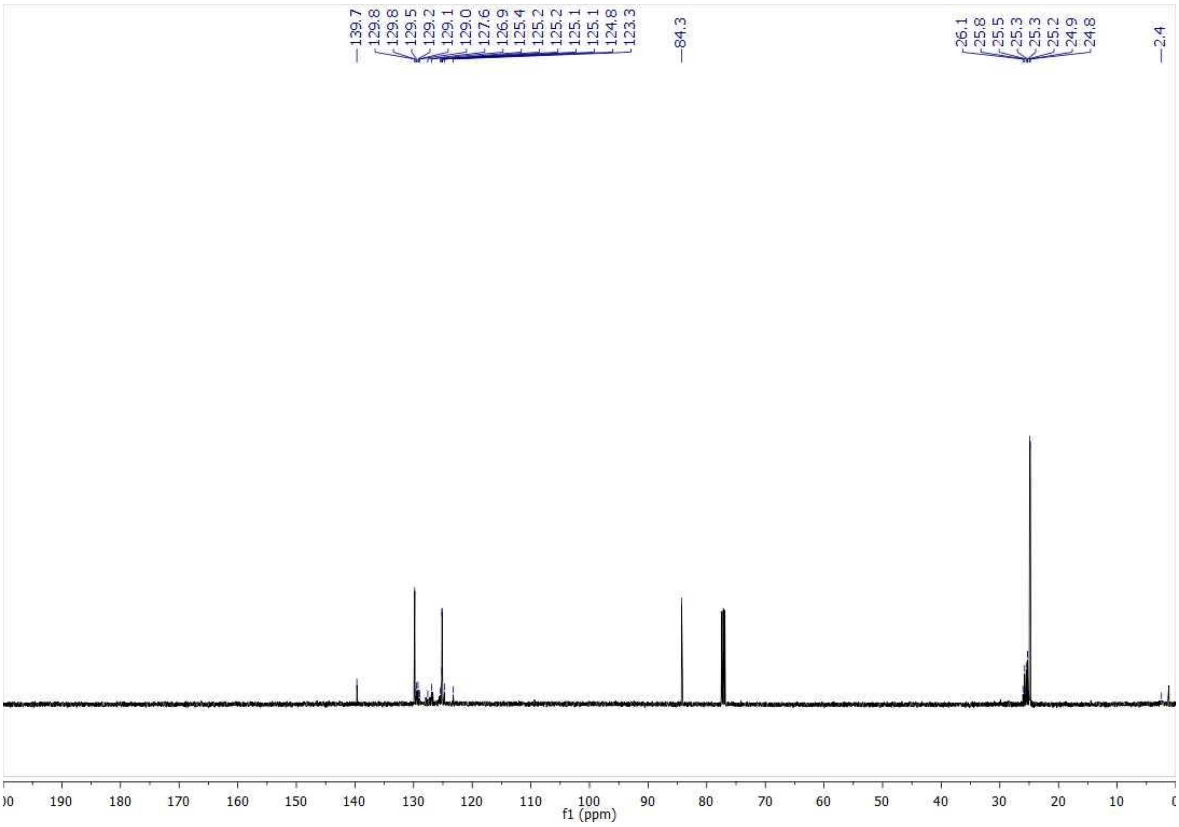

^{19}F NMR (282 MHz, $CDCl_3$)

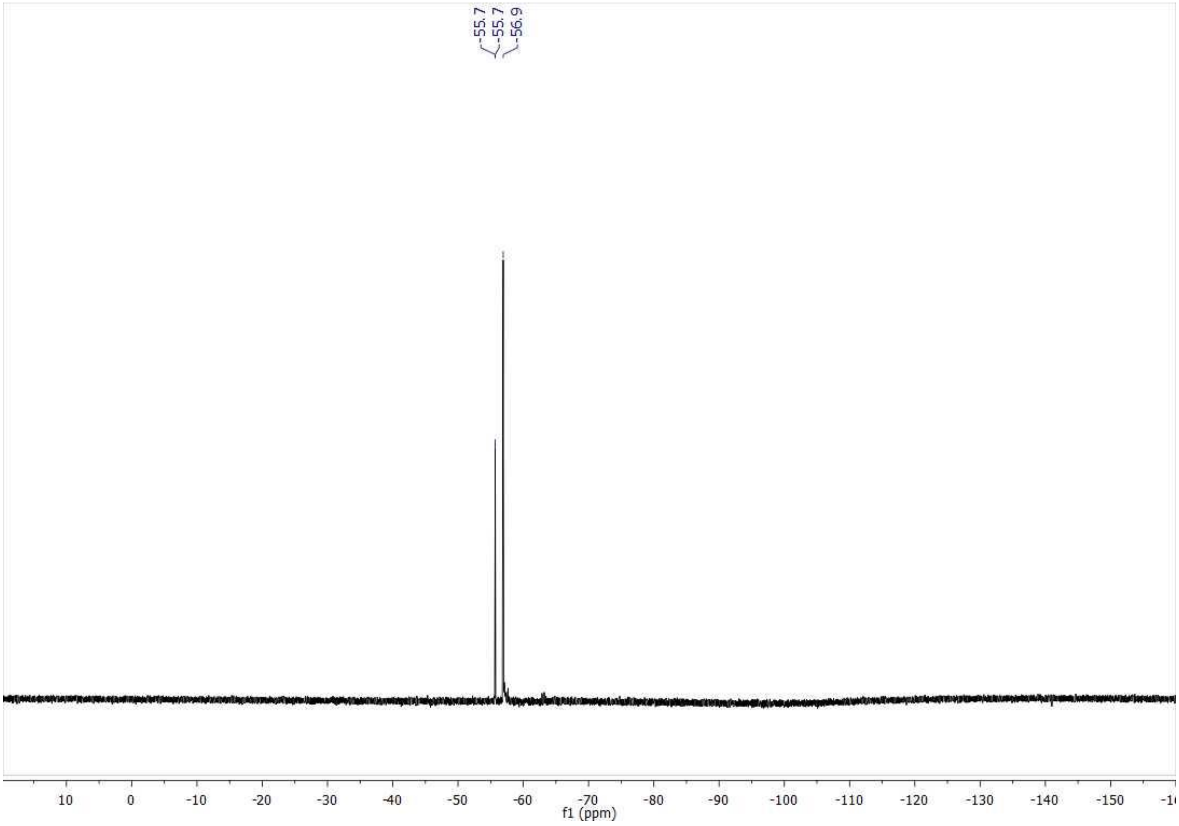

^{11}B NMR (160 MHz, $CDCl_3$)

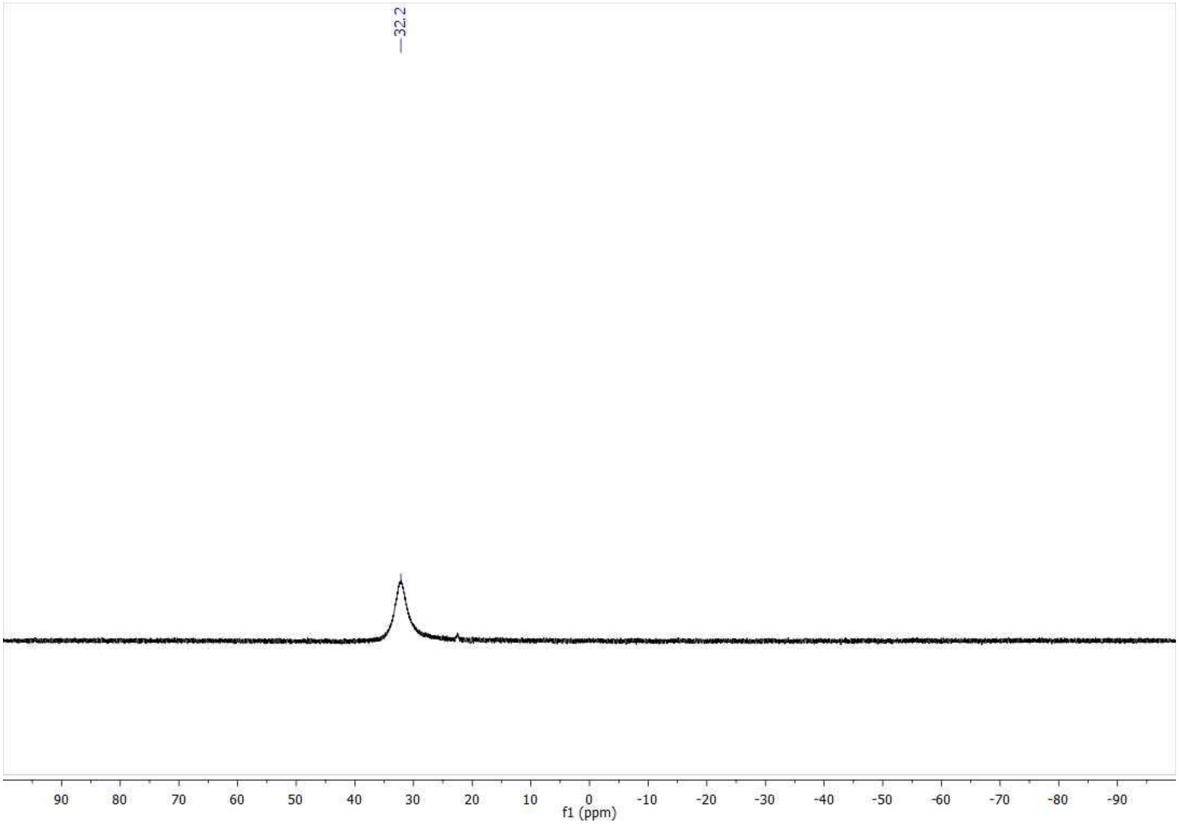

2-((1*S*,2*S*,3*R*)-2-(*p*-*Tert*butylphenyl)-3-(trifluoromethyl)cyclopropyl)-4,4,5,5-tetramethyl-1,3,2-dioxoborolane (2k)

^{1}H NMR (500 MHz, $CDCl_3$)

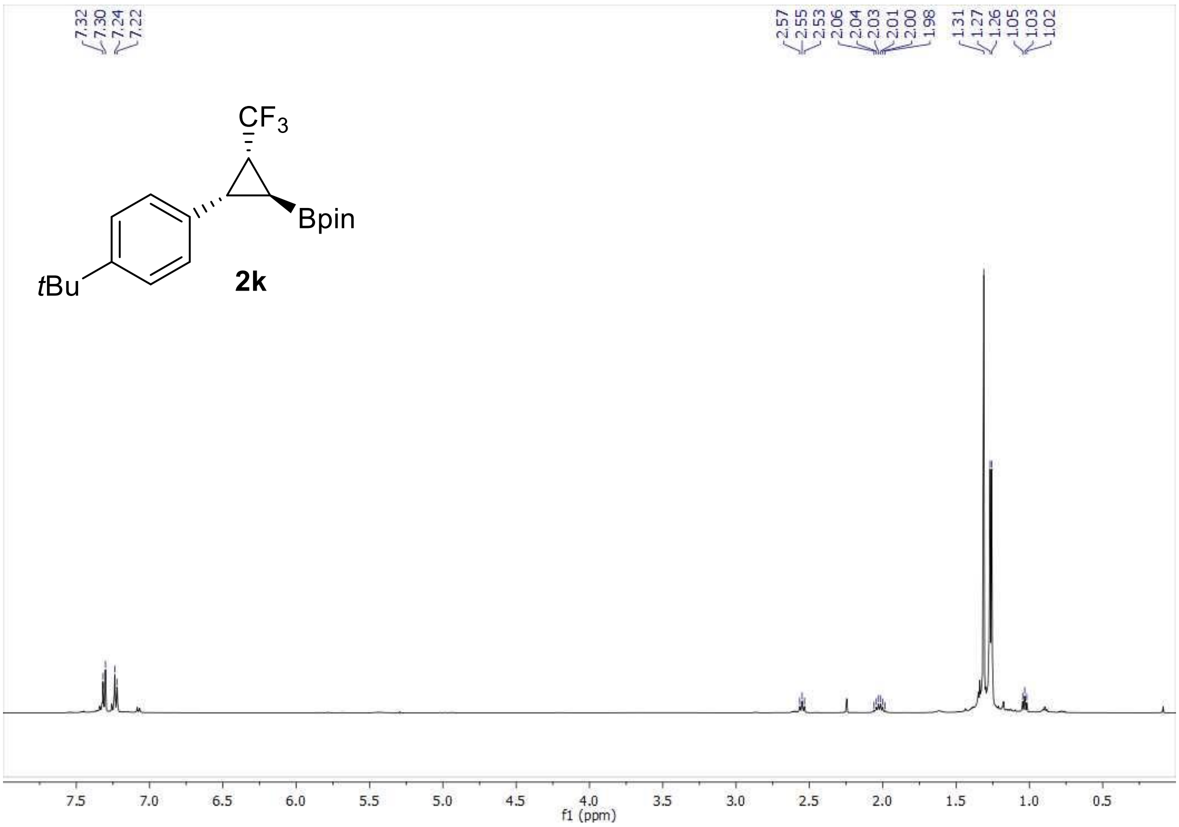

^{13}C NMR (126 MHz, $CDCl_3$)

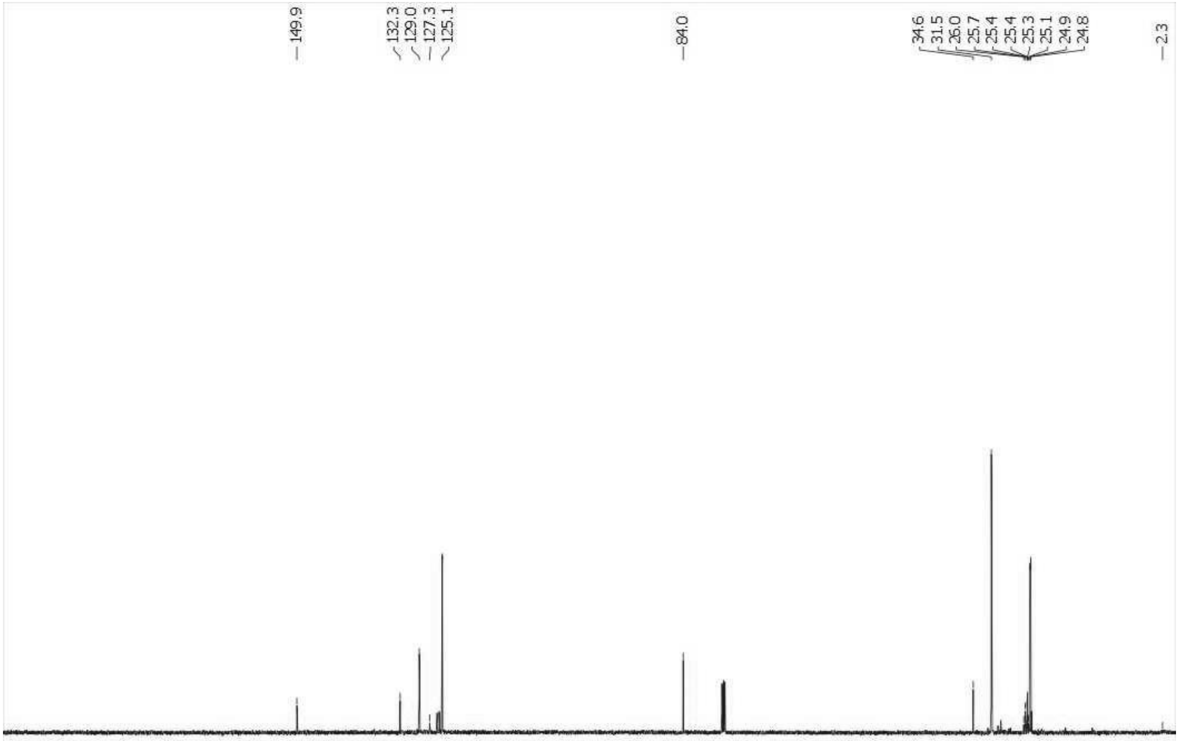

^{19}F NMR (282 MHz, $CDCl_3$)

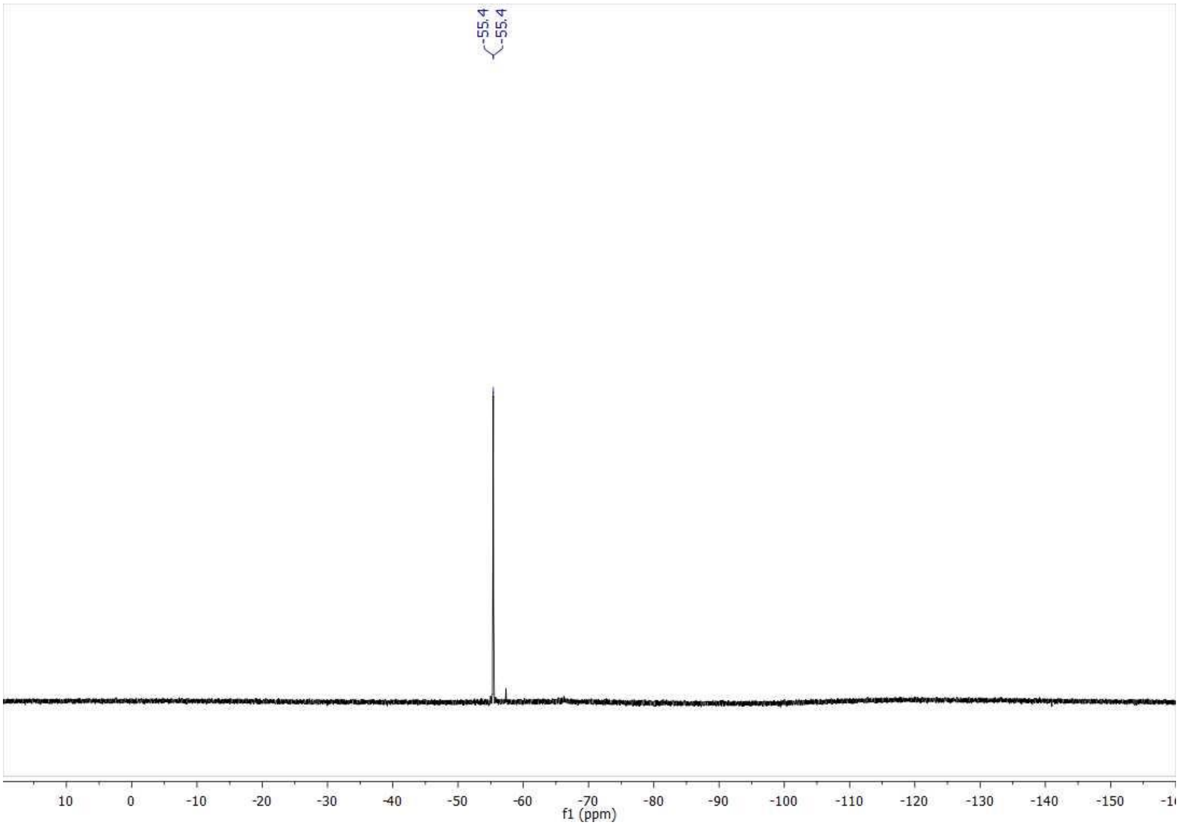

^{11}B NMR (160 MHz, $CDCl_3$)

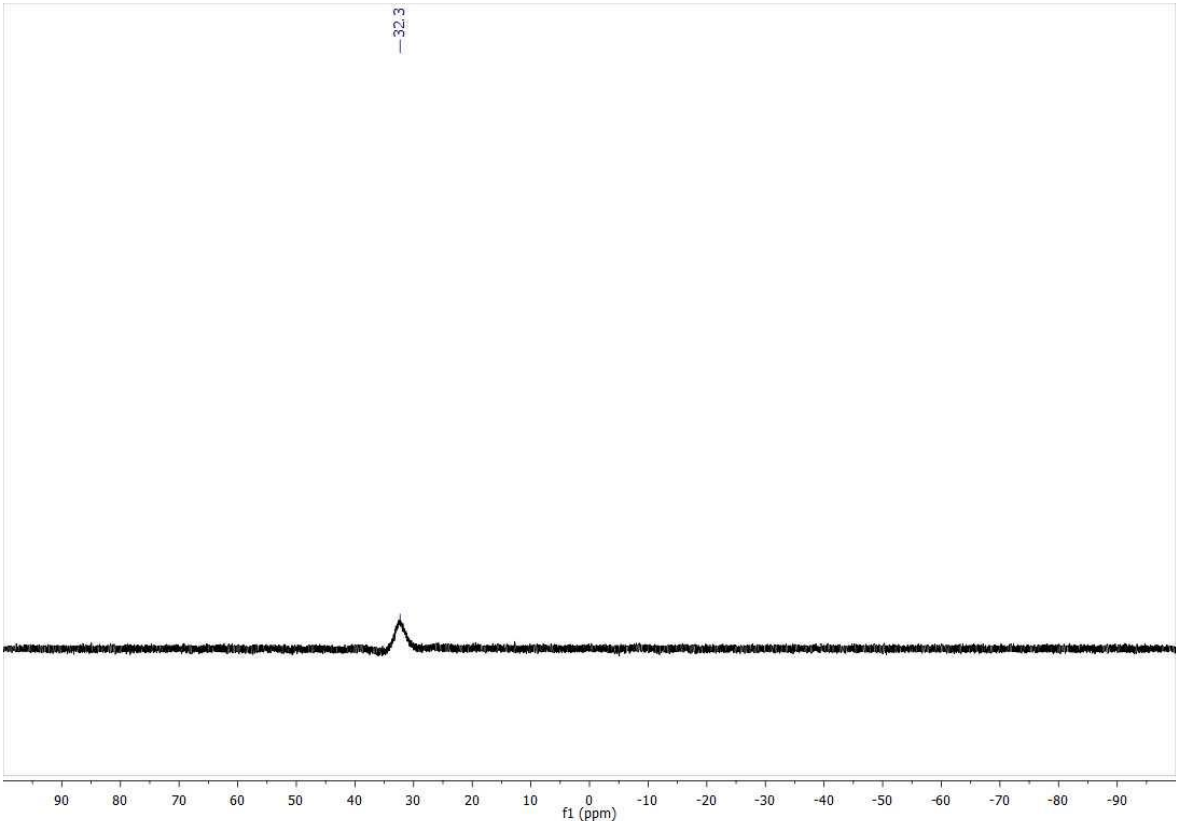

2-((1*S*,2*S*,3*R*)-2-(*p*-Methoxy)-3-(trifluoromethyl)cyclopropyl)-4,4,5,5-tetramethyl-1,3,2-dioxoborolane (2l)

^{1}H NMR (500 MHz, $CDCl_3$)

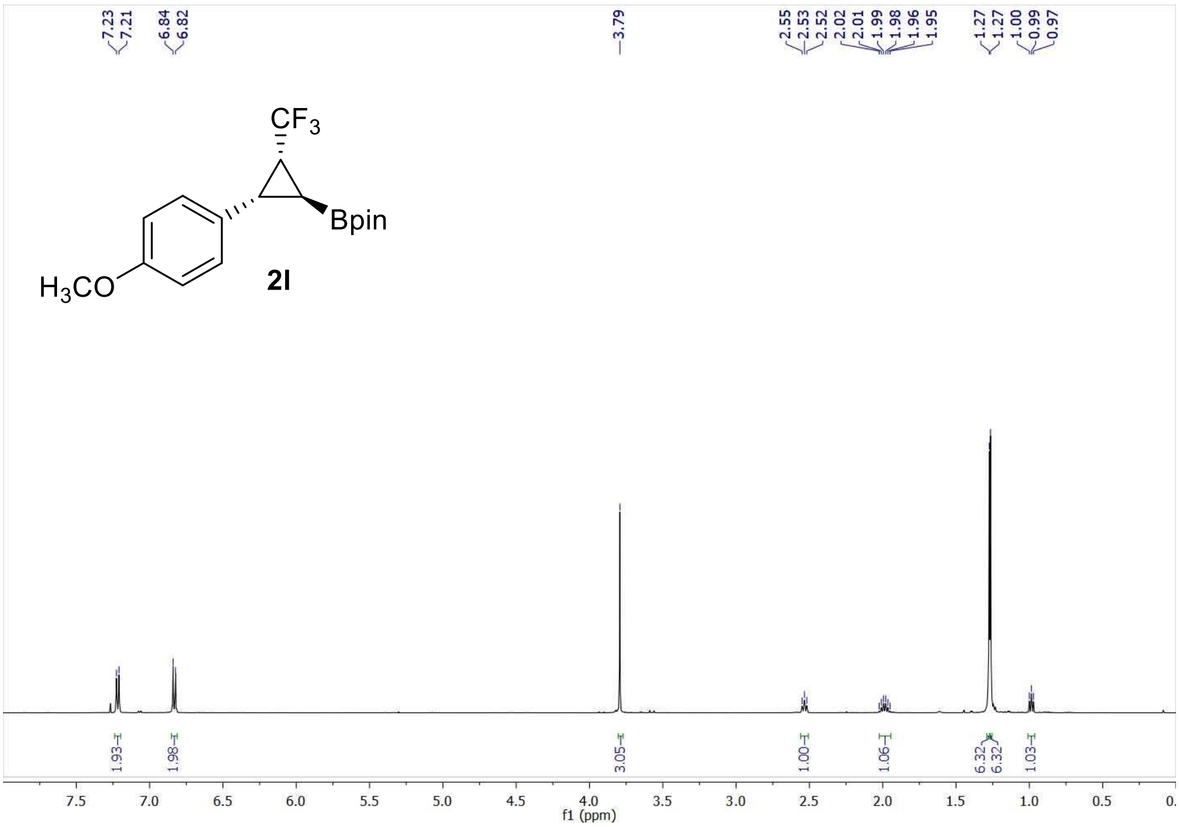

^{13}C NMR (126 MHz, $CDCl_3$)

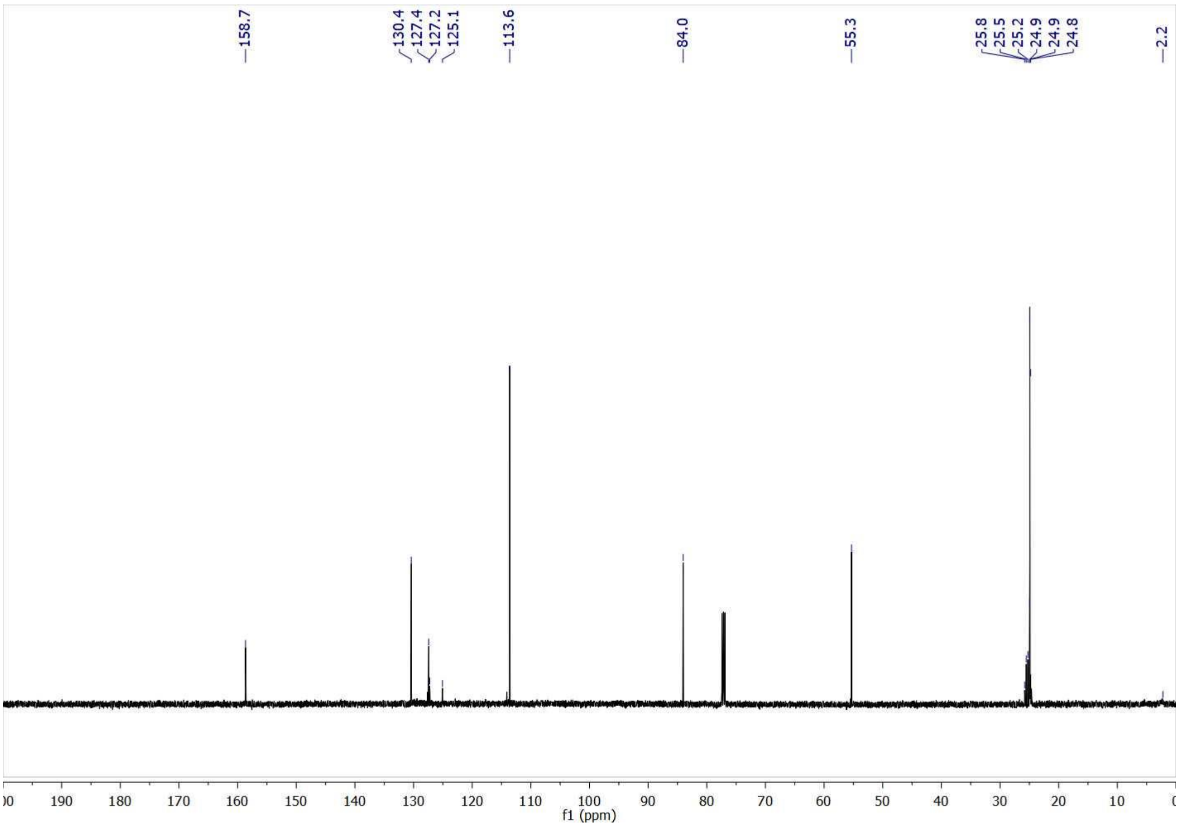

^{19}F NMR (282 MHz, $CDCl_3$)

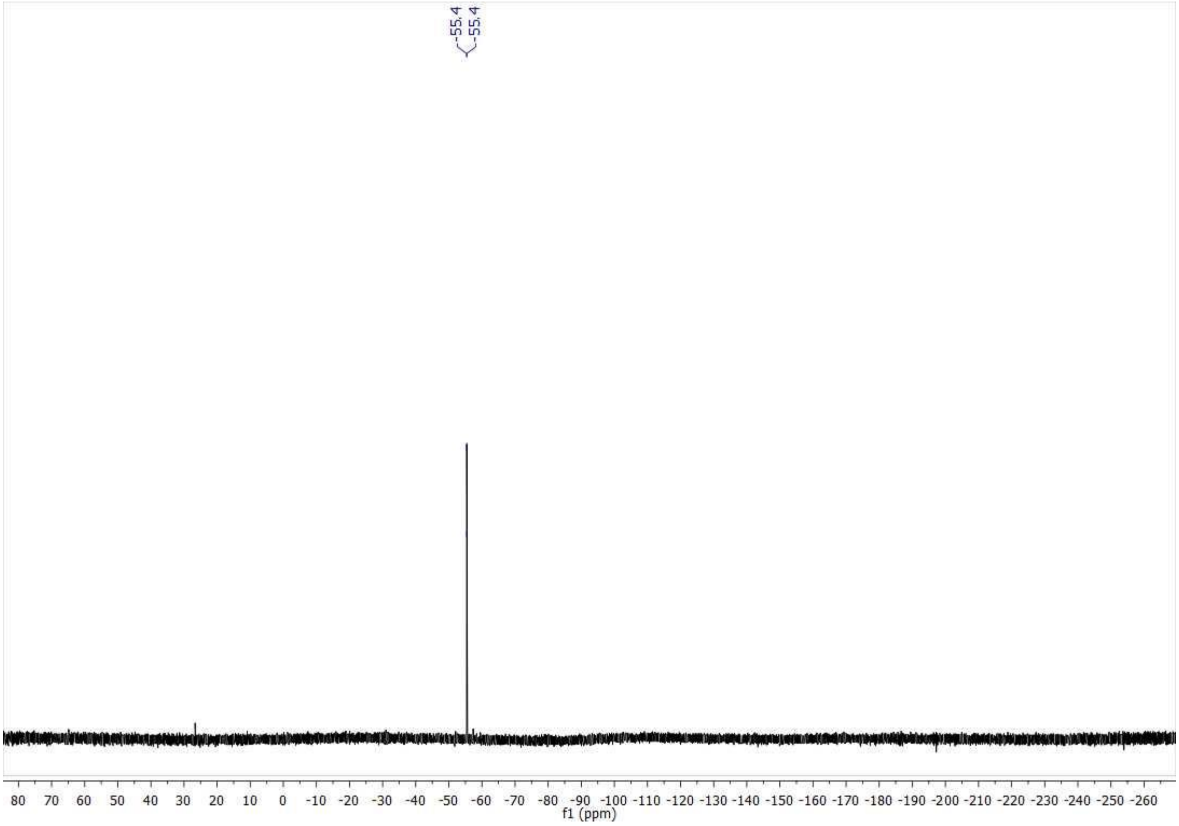

^{11}B NMR (160 MHz, $CDCl_3$)

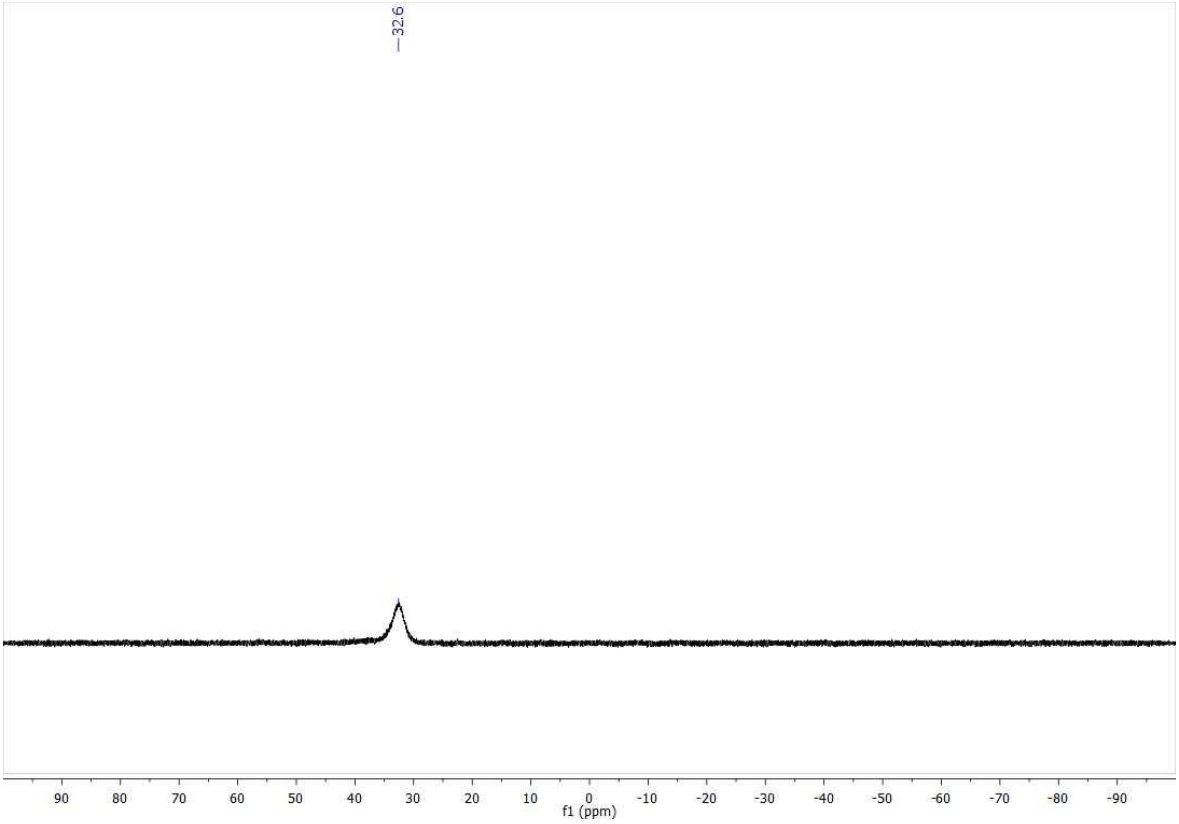

Methyl 4-((1*S*,2*S*,3*R*)-2-(4,4,5,5-tetramethyl-1,3,2-dioxoborolan-2-yl)-3-(trifluoromethyl)cyclopropyl)benzoate (2o)

^{1}H NMR (400 MHz, $CDCl_3$)

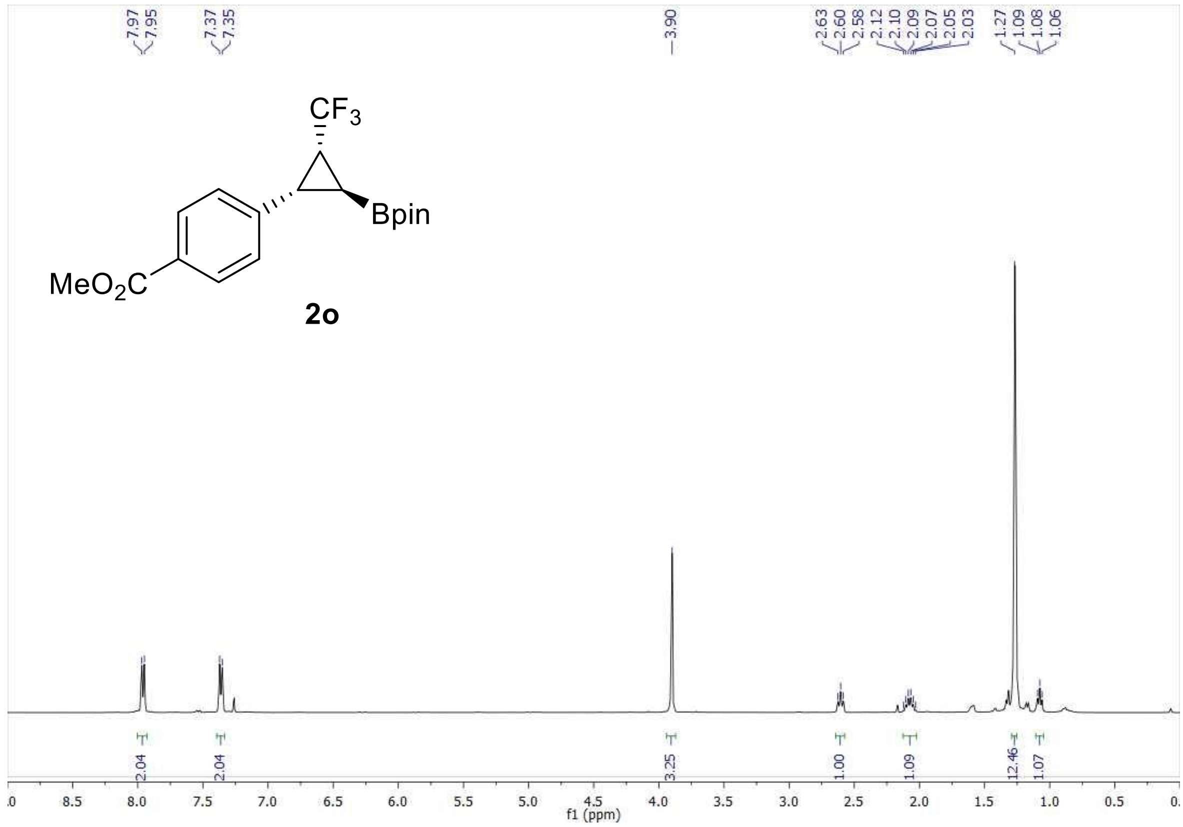

^{13}C NMR (101 MHz, $CDCl_3$)

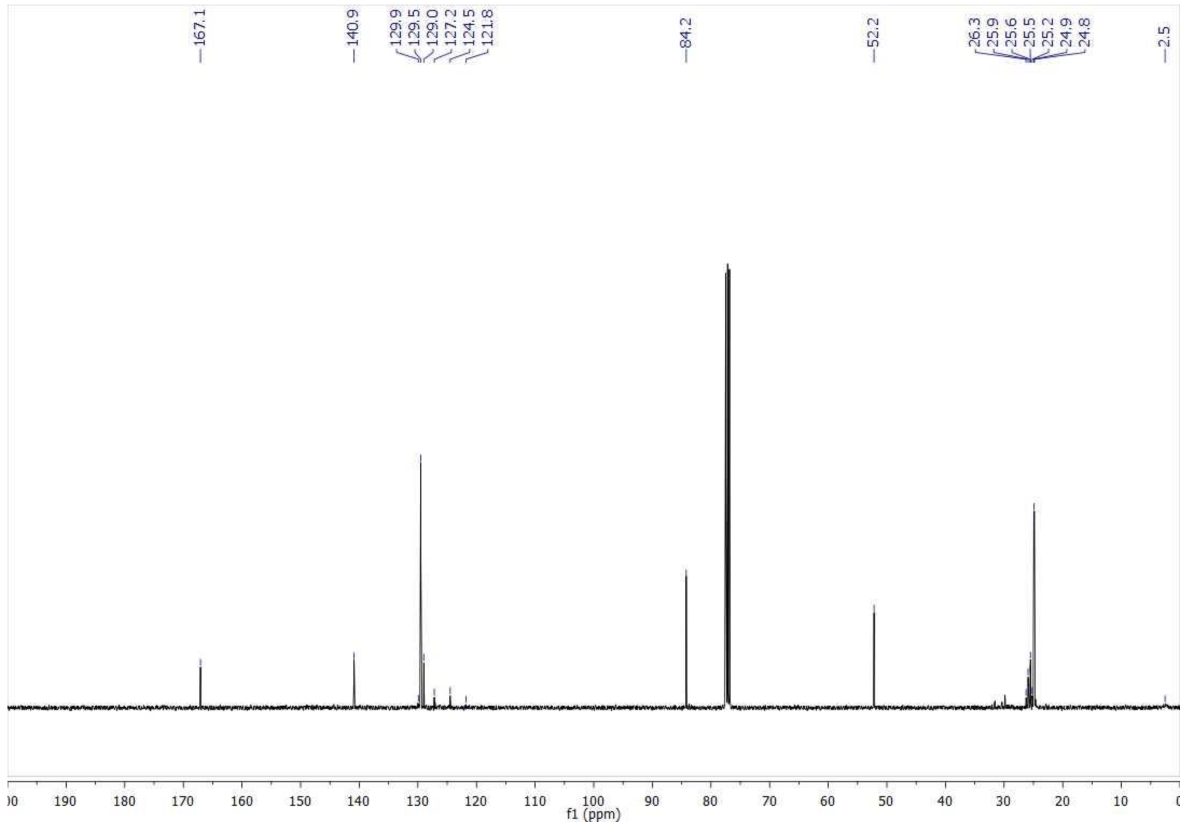

^{19}F NMR (376 MHz, $CDCl_3$)

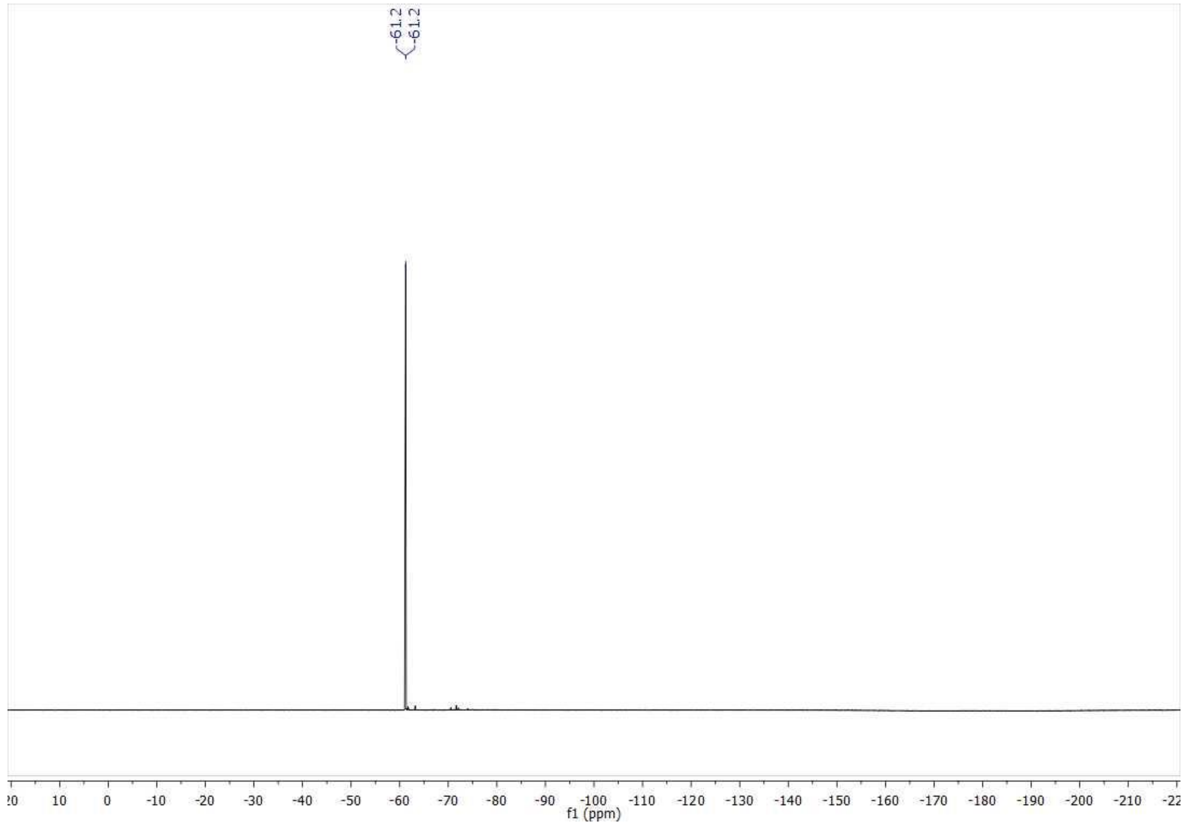

^{11}B NMR (160 MHz, $CDCl_3$)

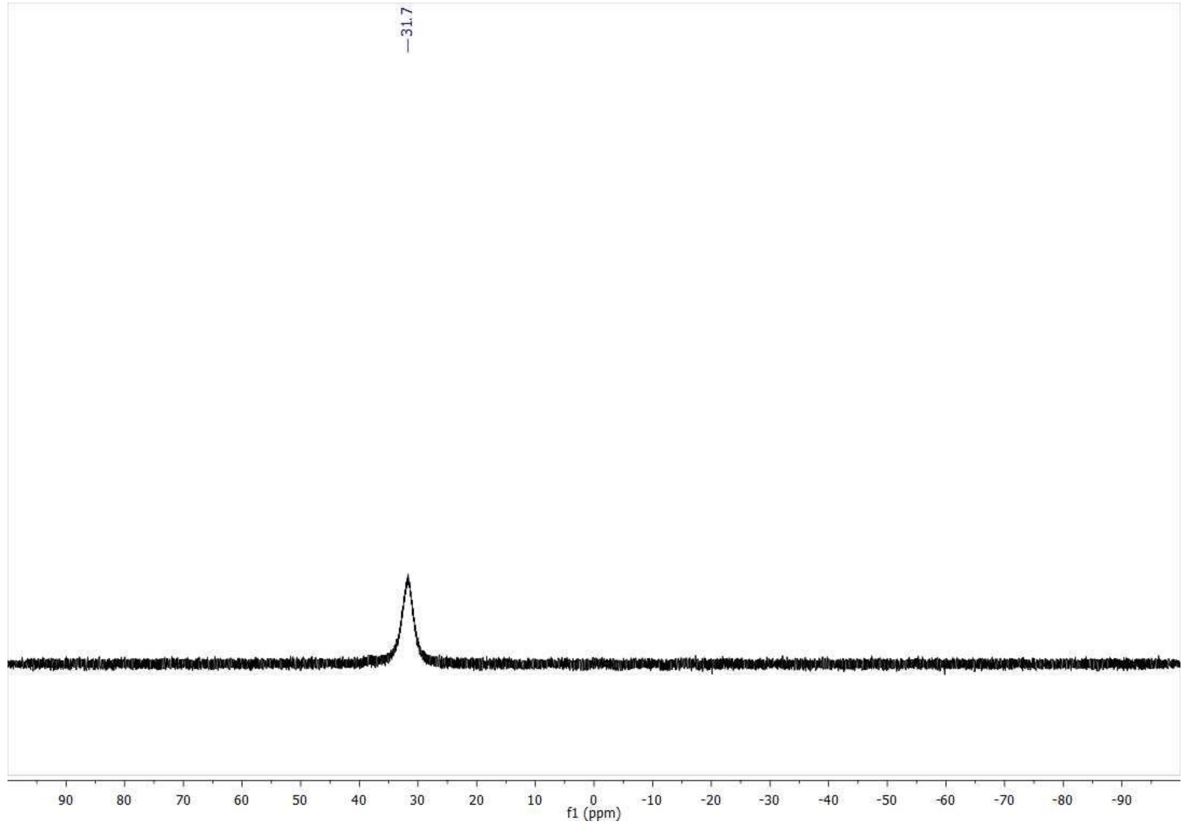

2-((1*S*,2*S*,3*R*)-2-(2,4,5-trimethylphenyl)-3-(trifluoromethyl)cyclopropyl)-4,4,5,5-tetramethyl-1,3,2-dioxoborolane (2p)

^{1}H NMR (400 MHz, $CDCl_3$)

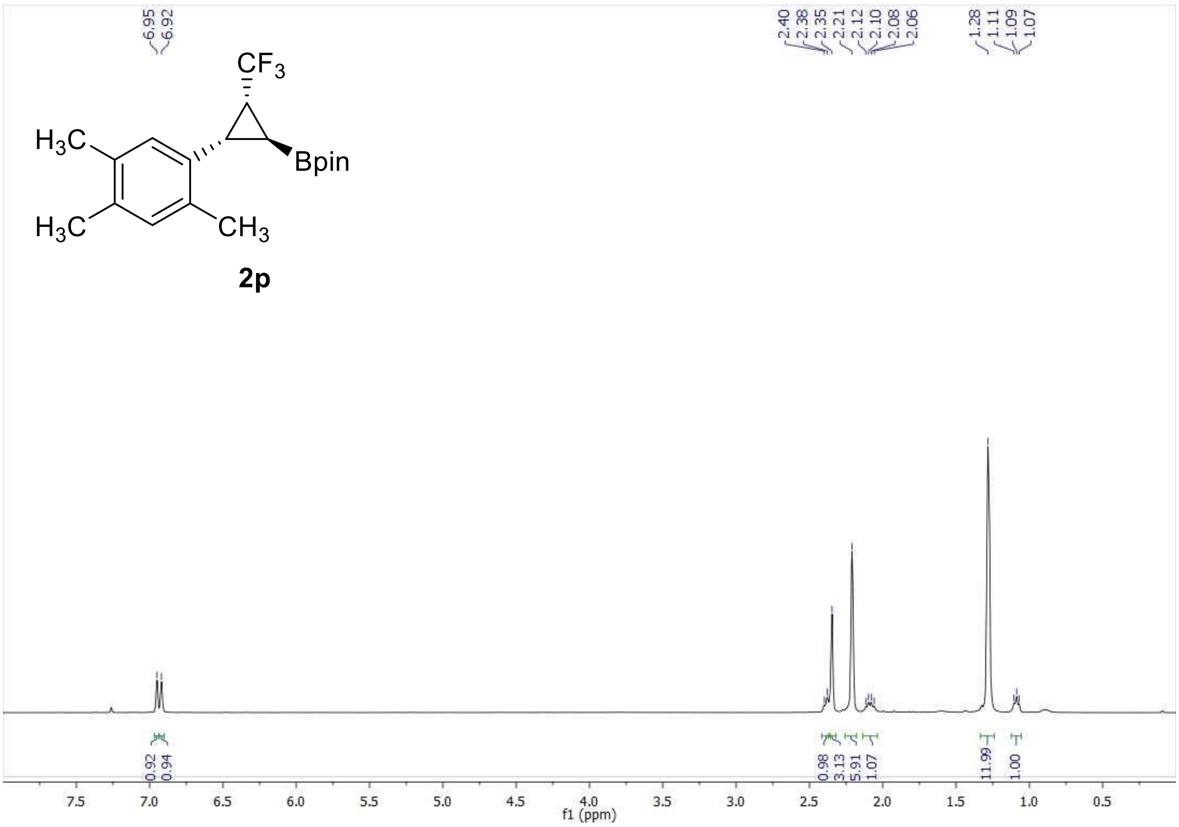

^{13}C NMR (101 MHz, $CDCl_3$)

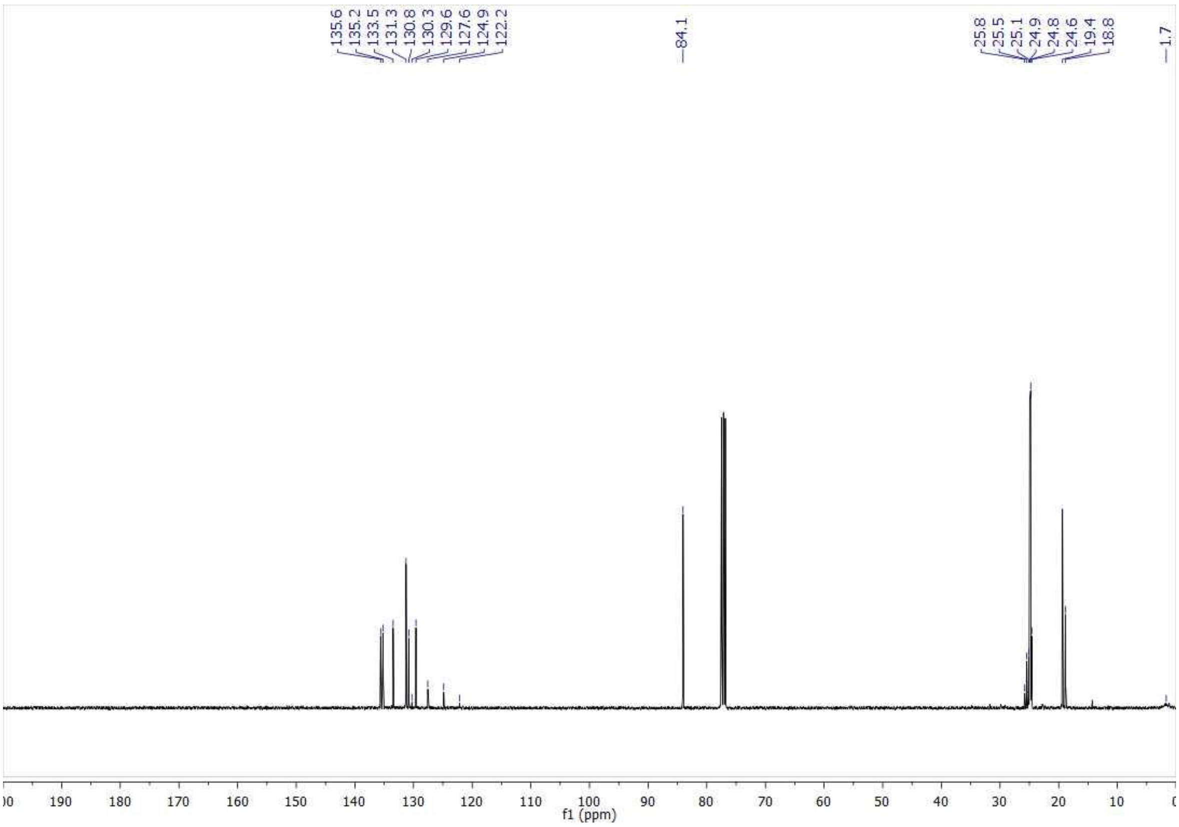

^{19}F NMR (376 MHz, $CDCl_3$)

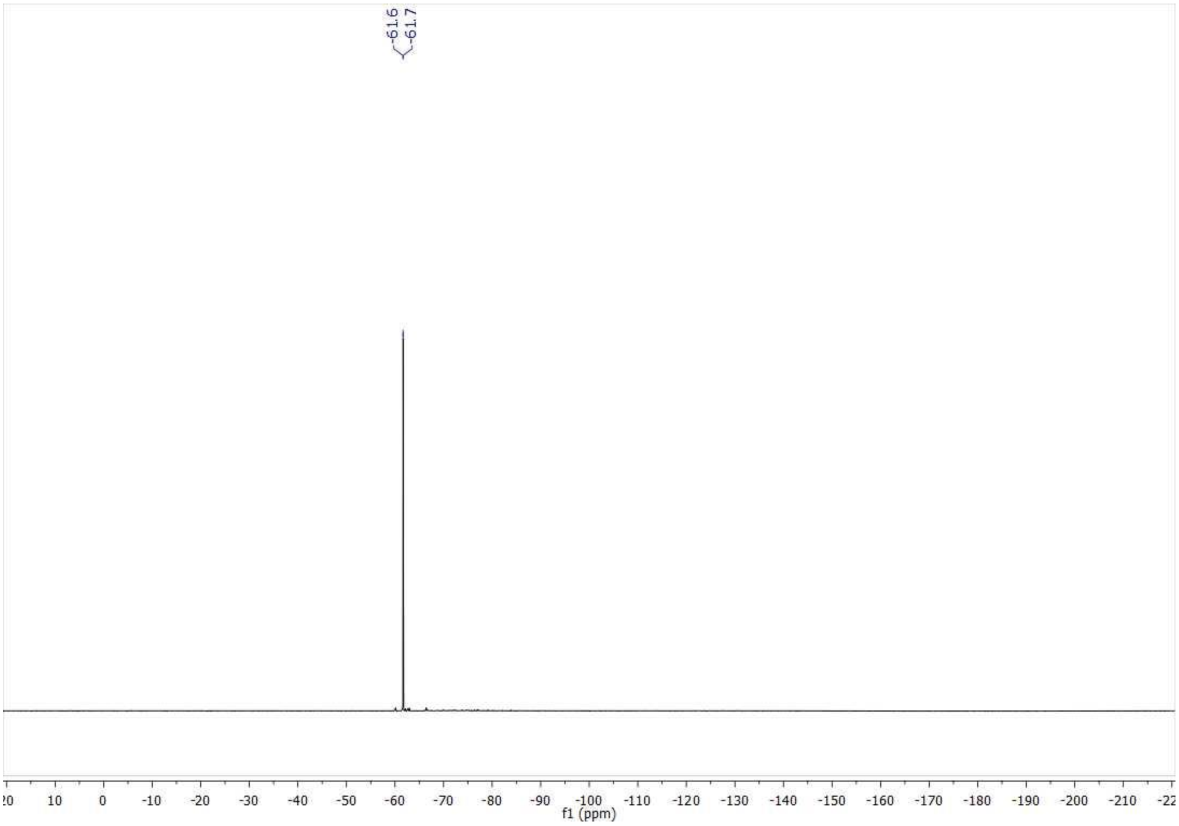

^{11}B NMR (160 MHz, $CDCl_3$)

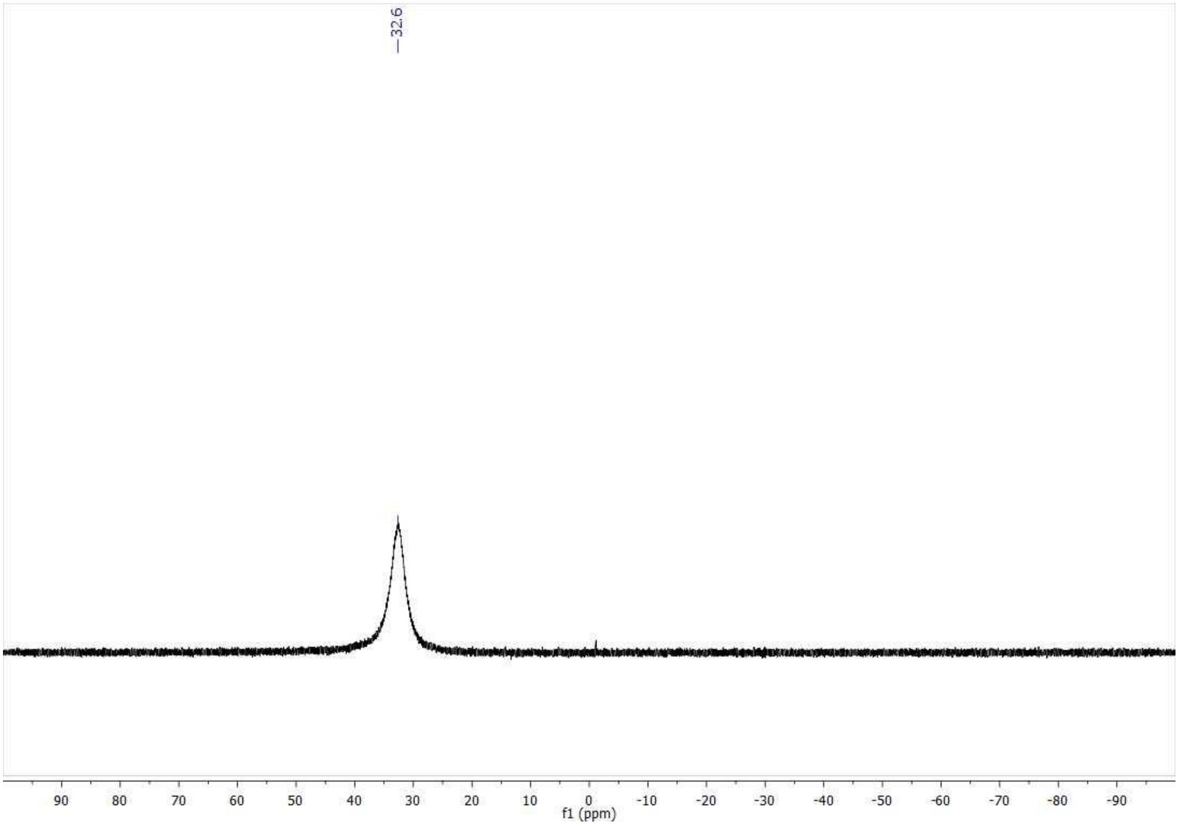

2-((1*S*,2*S*,3*R*)-2-(Thiophen-3-yl)-3-(trifluoromethyl)cyclopropyl)-4,4,5,5-tetramethyl-1,3,2-dioxoborolane (2q)

^{1}H NMR (500 MHz, $CDCl_3$)

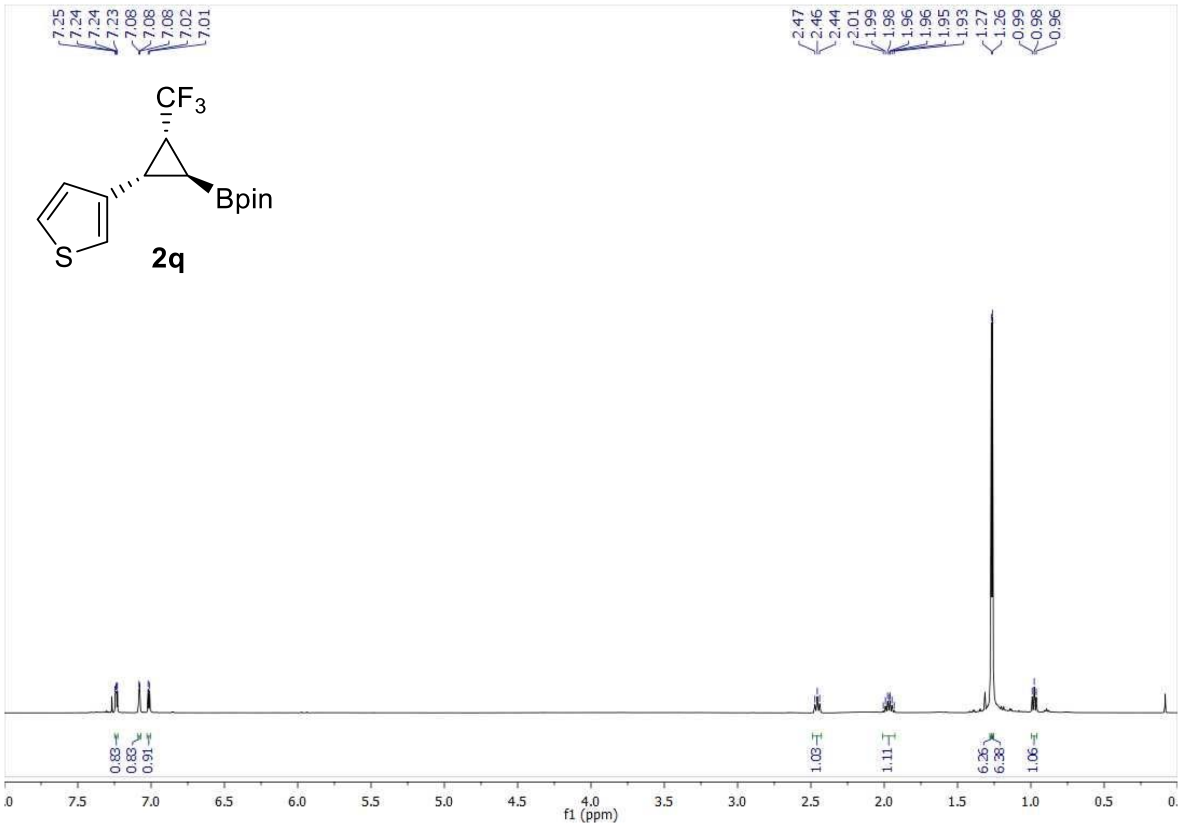

^{13}C NMR (126 MHz, $CDCl_3$)

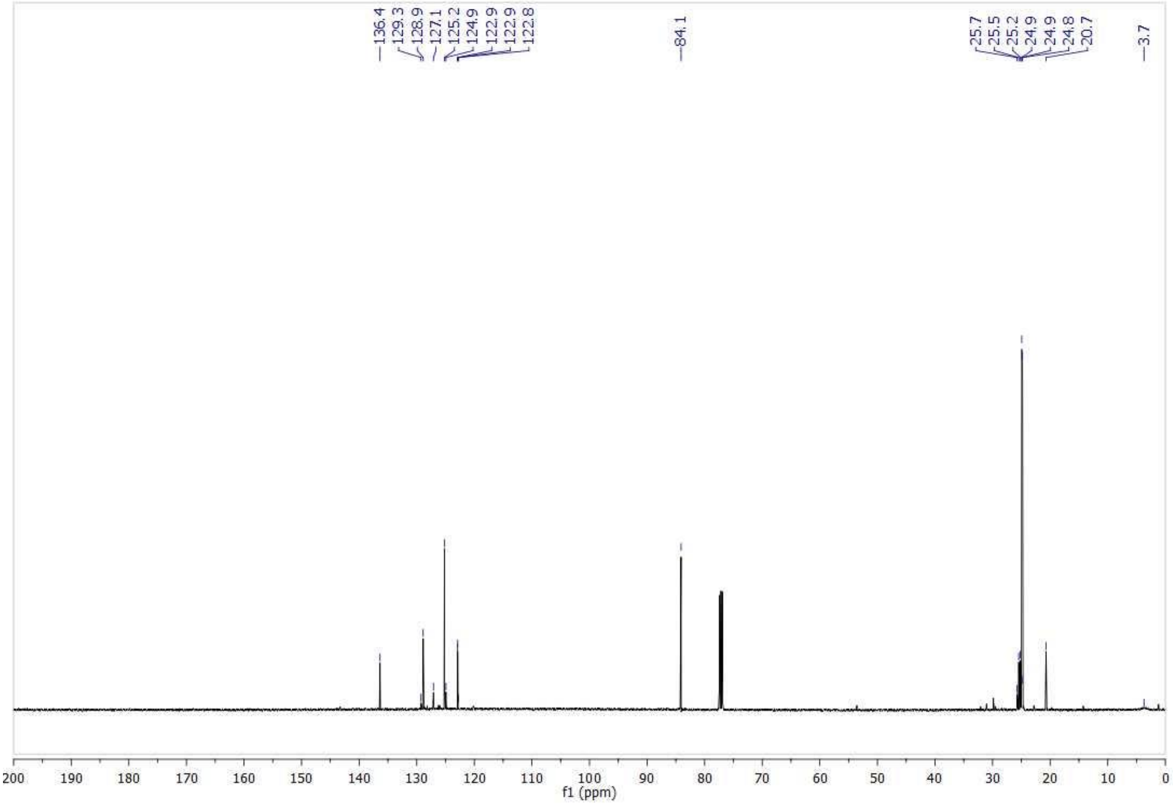

^{19}F NMR (282 MHz, $CDCl_3$)

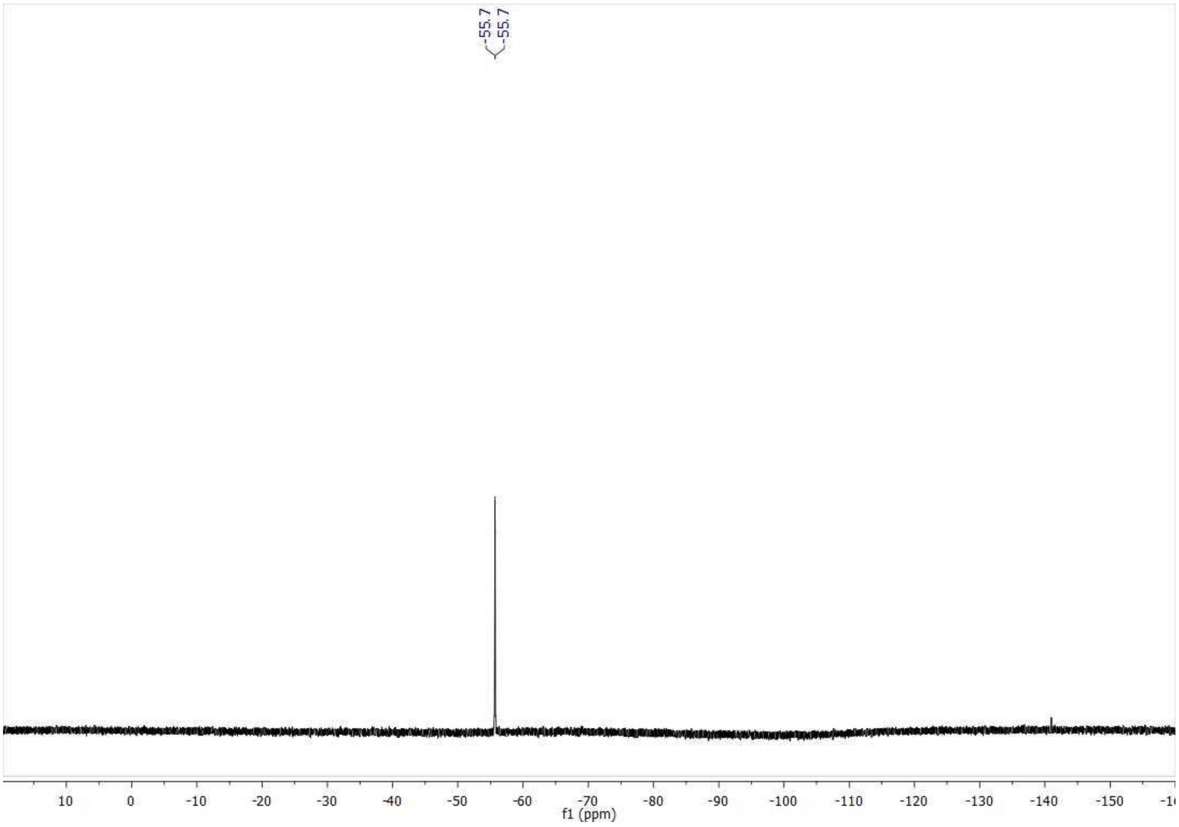

^{11}B NMR (160 MHz, $CDCl_3$)

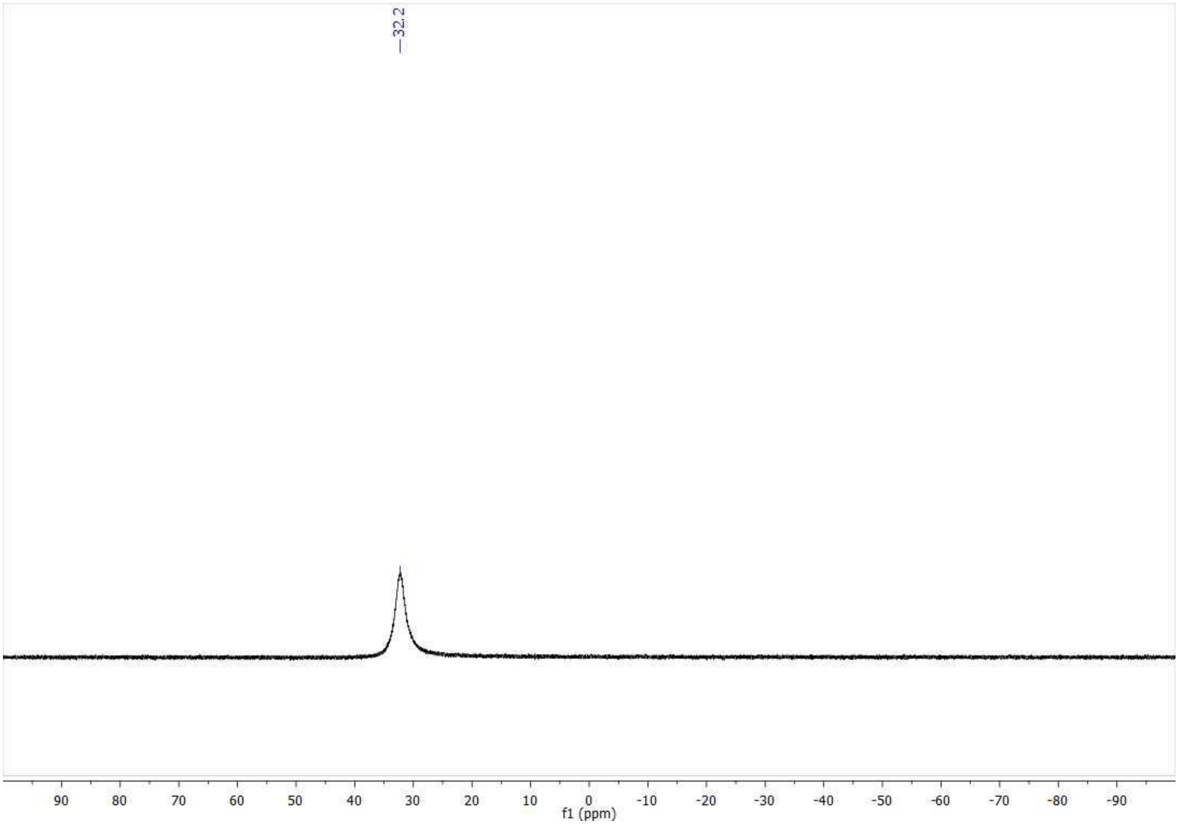

2-((1*S*,2*S*,3*R*)-2-Phenethyl-3-(trifluoromethyl)cyclopropyl)-4,4,5,5-tetramethyl-1,3,2-dioxoborolane (2r)

^{1}H NMR (500 MHz, $CDCl_3$)

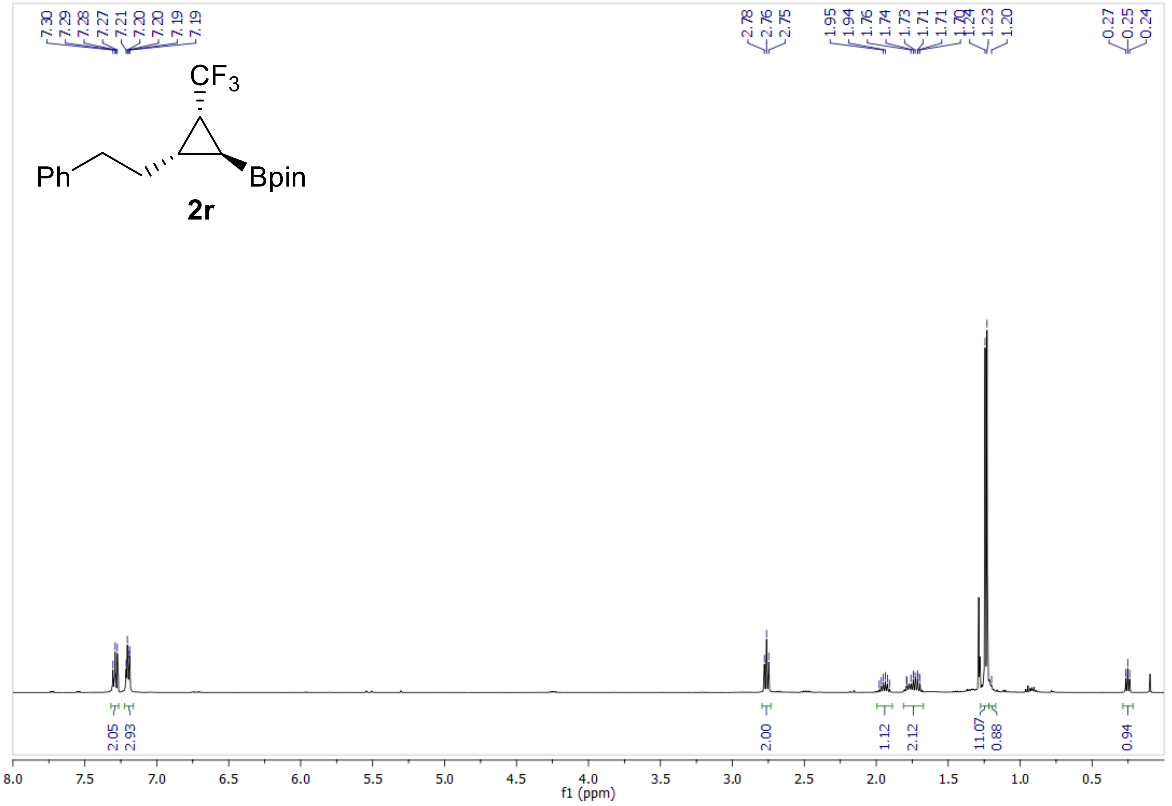

^{13}C NMR (126 MHz, $CDCl_3$)

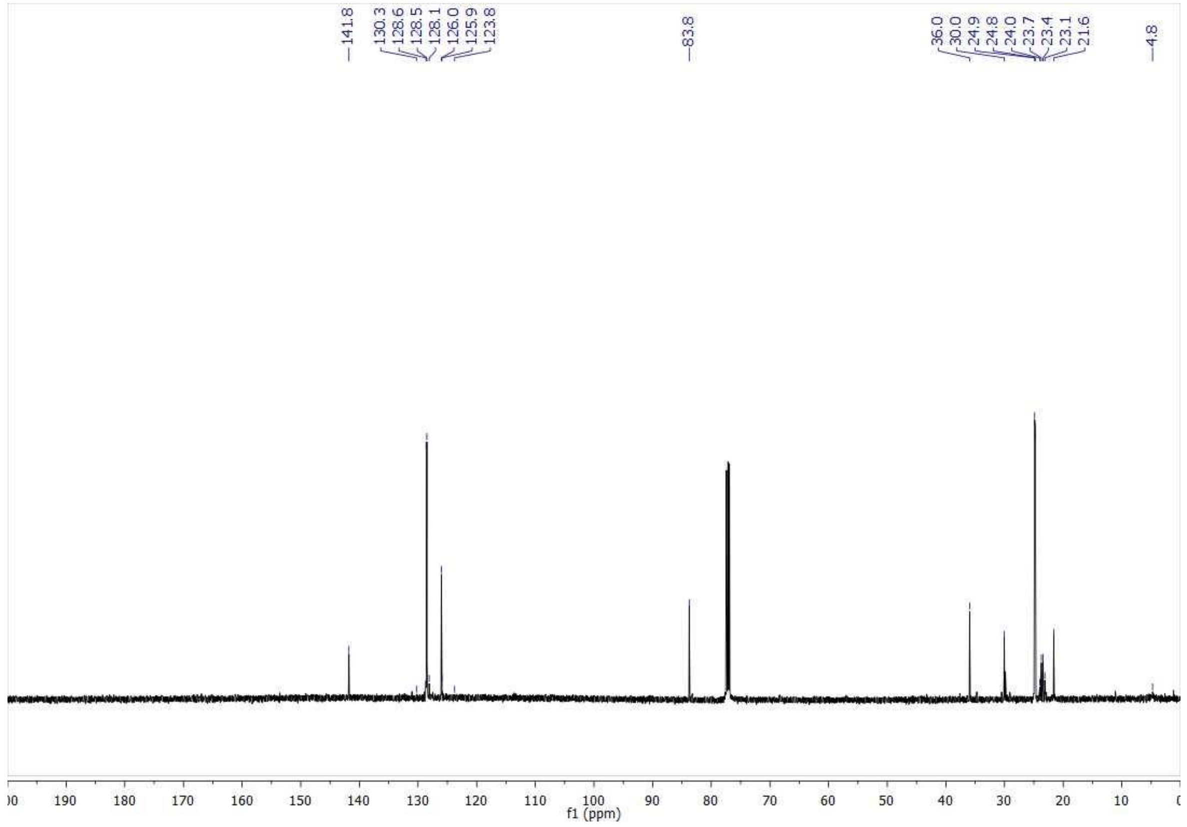

^{19}F NMR (282 MHz, $CDCl_3$)

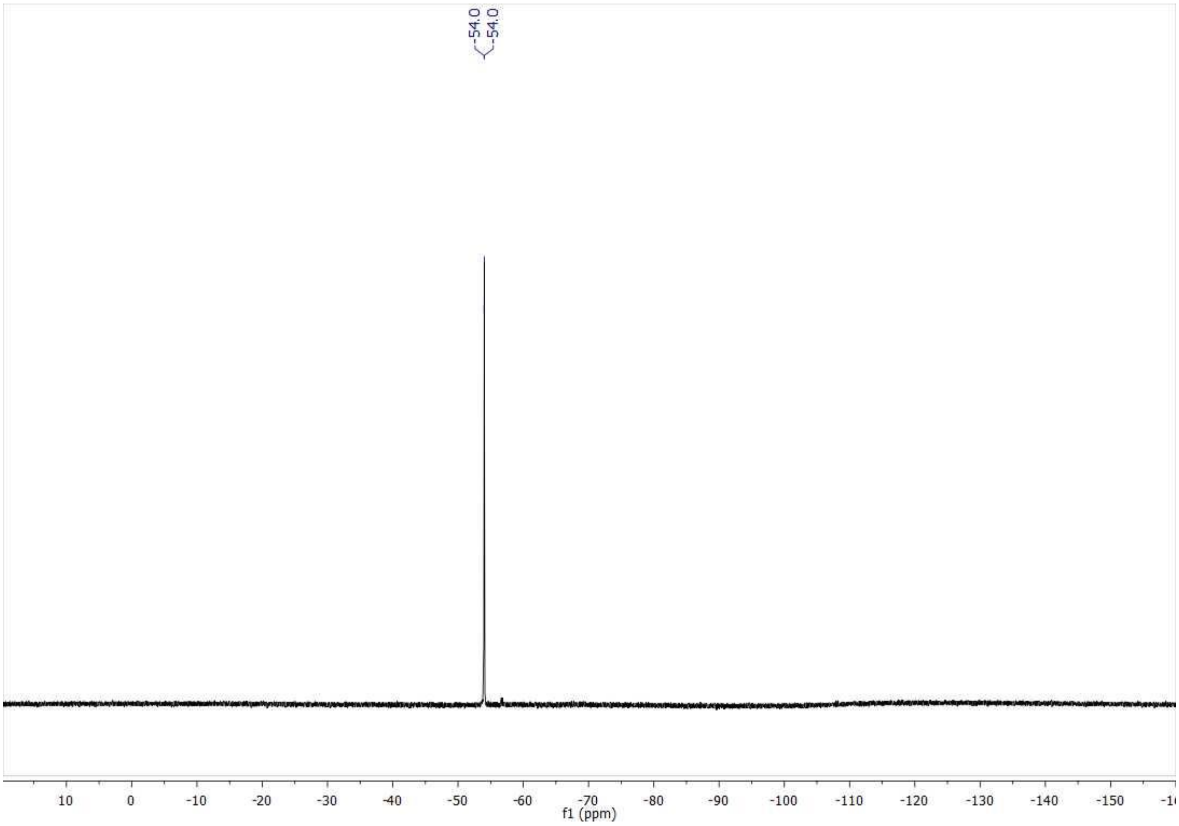

^{11}B NMR (160 MHz, $CDCl_3$)

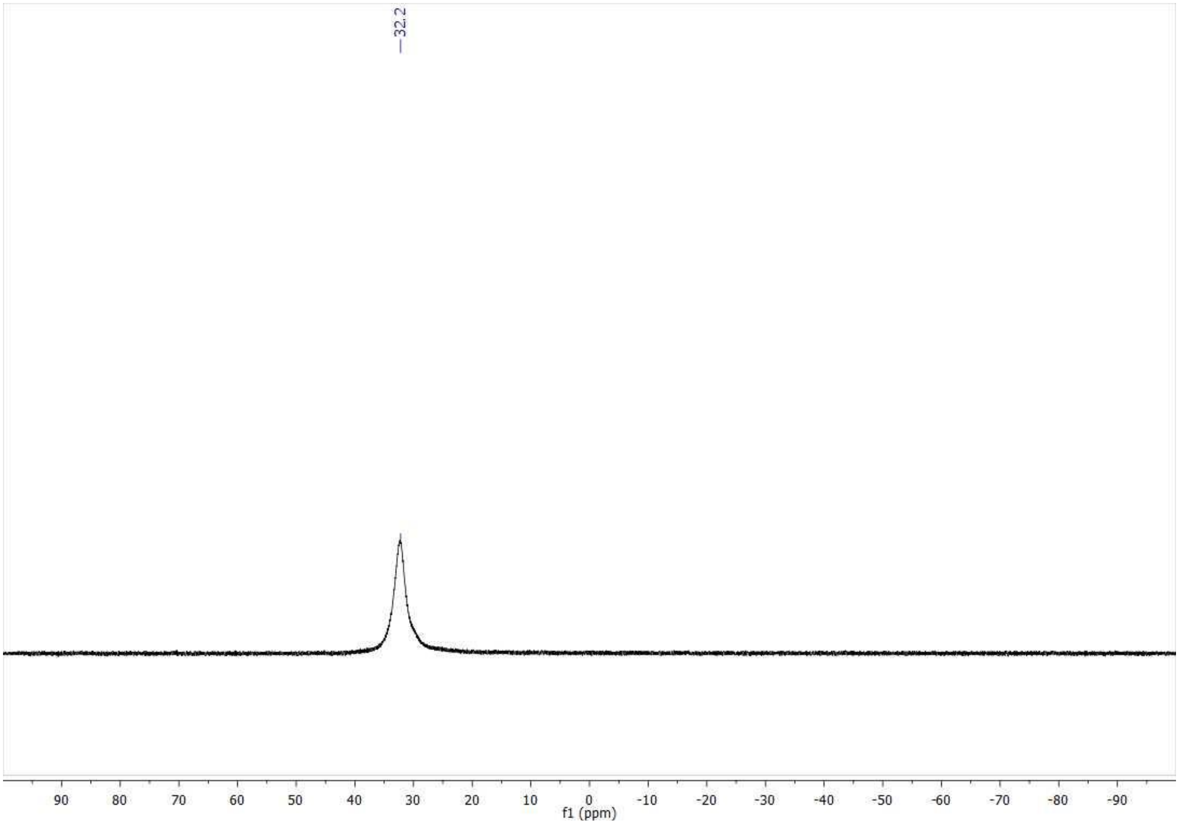

(1*S*,2*S*,3*R*)-2-Phenyl-3-(trifluoromethyl)cyclopropyl)boronic acid (3)

^{1}H NMR (300 MHz, CD_3OD)

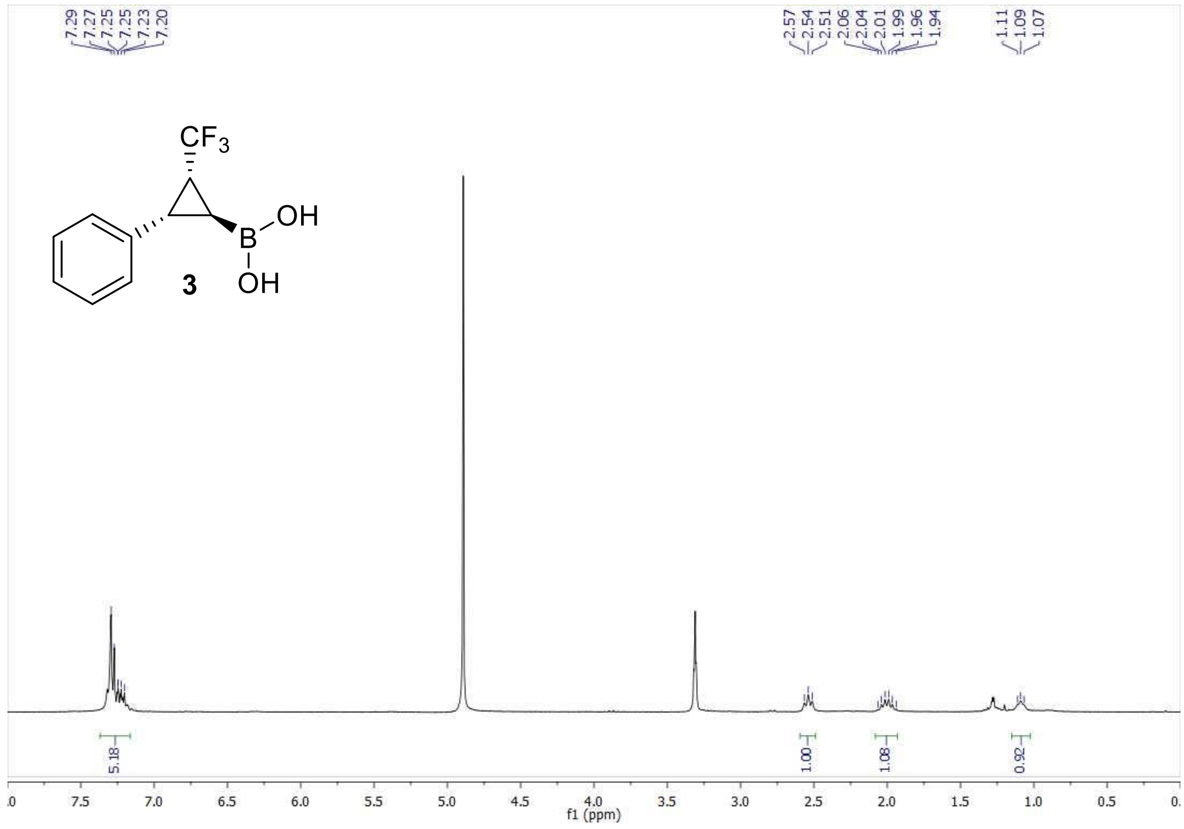

^{13}C NMR (126 MHz, CD_3OD)

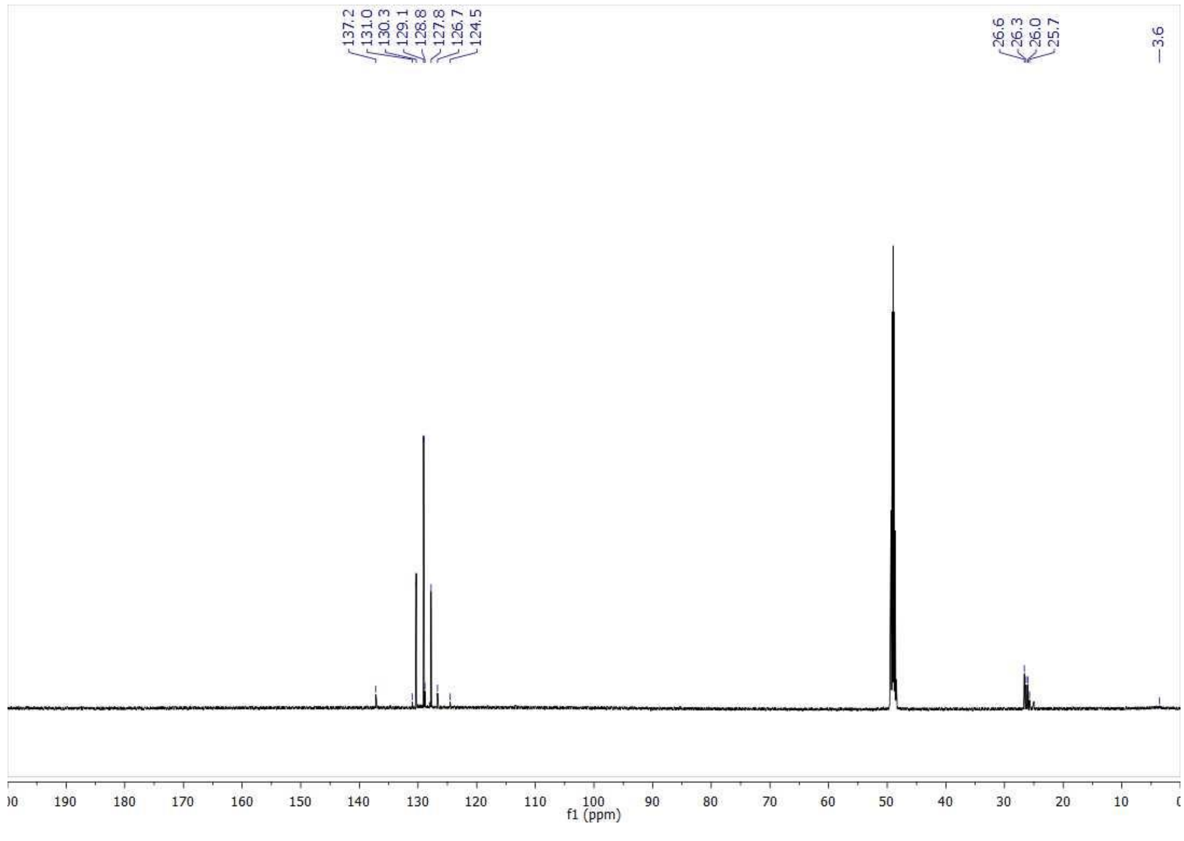

^{19}F NMR (282 MHz, $CDCl_3$)

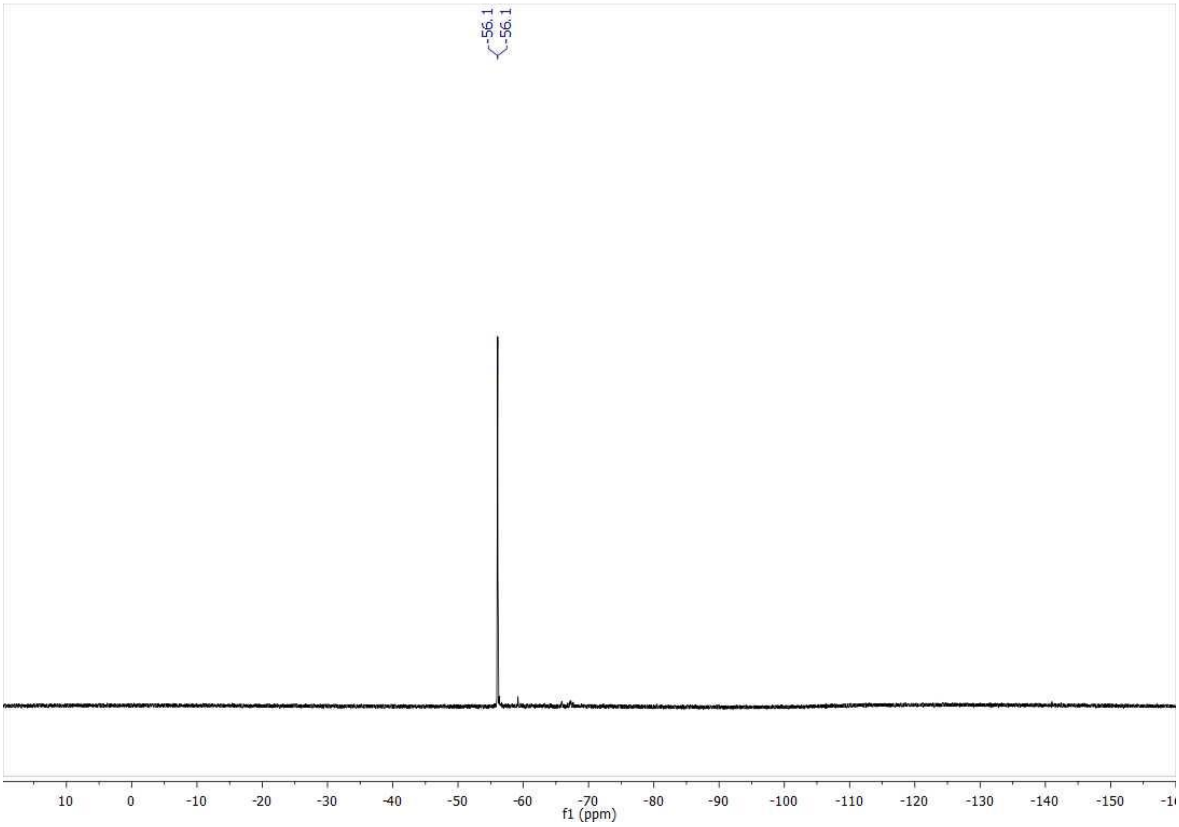

1-Methoxy-4-((1*S*, 2*S*, 3*R*)-2-phenyl-3-(trifluoromethyl)cyclopropyl)benzene (4)

^{1}H NMR (500 MHz, $CDCl_3$)

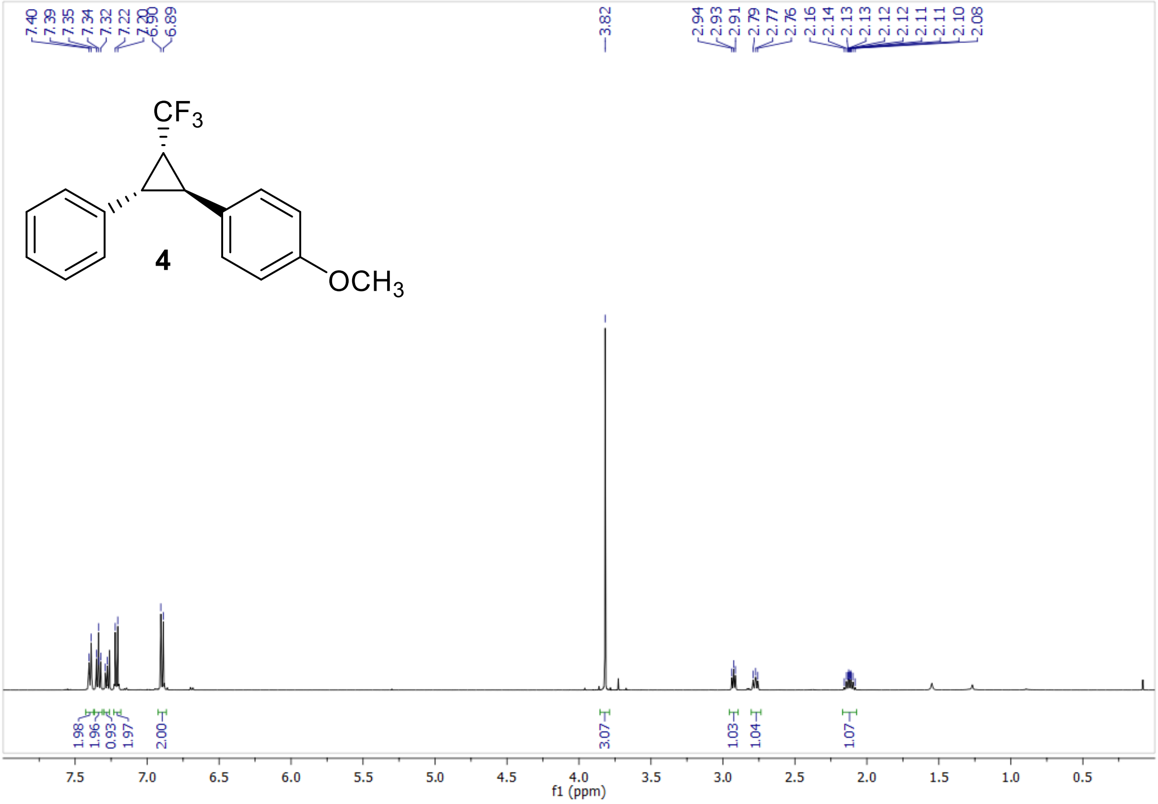

^{13}C NMR (126 MHz, $CDCl_3$)

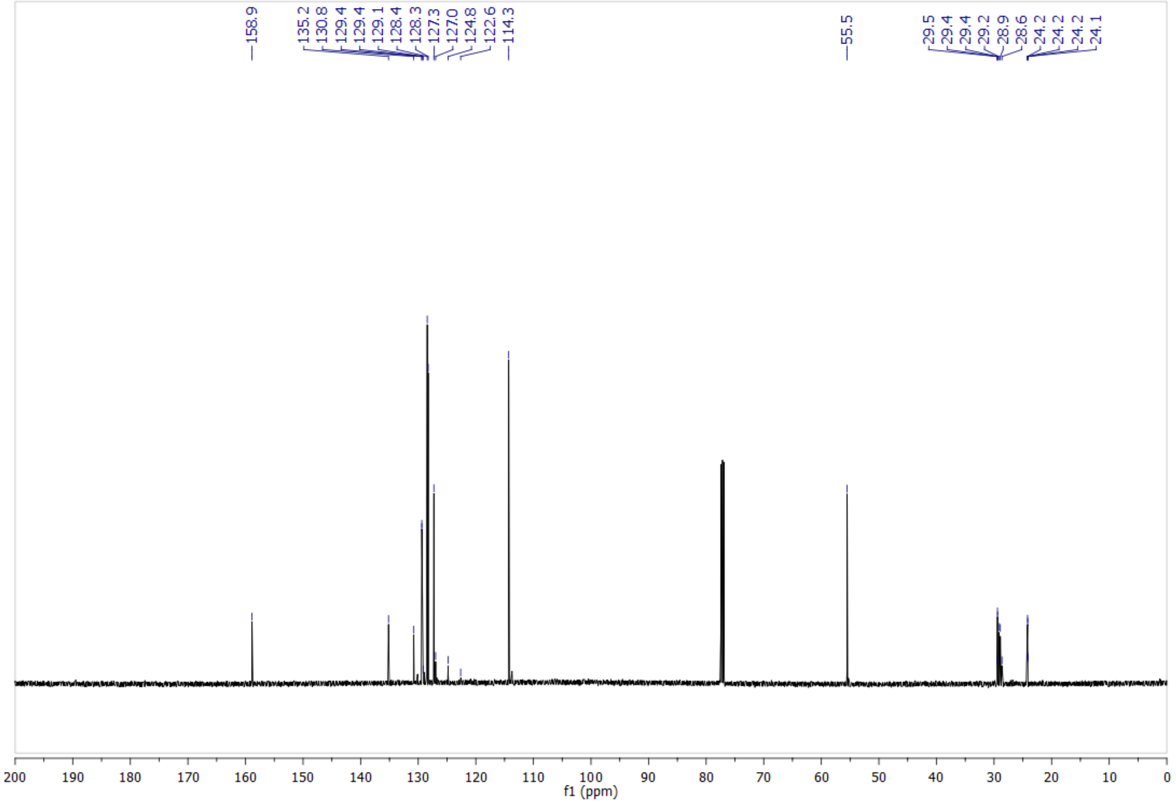

^{19}F NMR (282 MHz, $CDCl_3$)

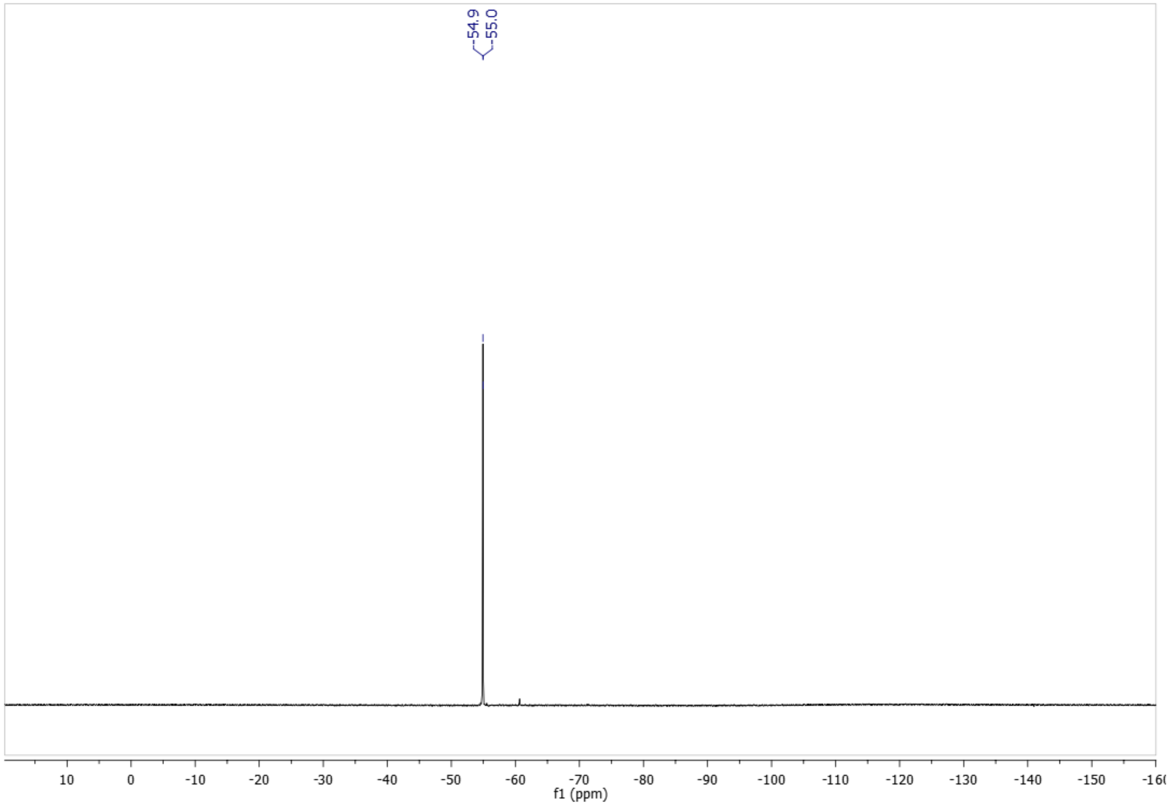

(1*S*,2*S*,3*R*)-2-Phenyl-3-(trifluoromethyl)cyclopropanol (5)

^{1}H NMR (300 MHz, $CDCl_3$)

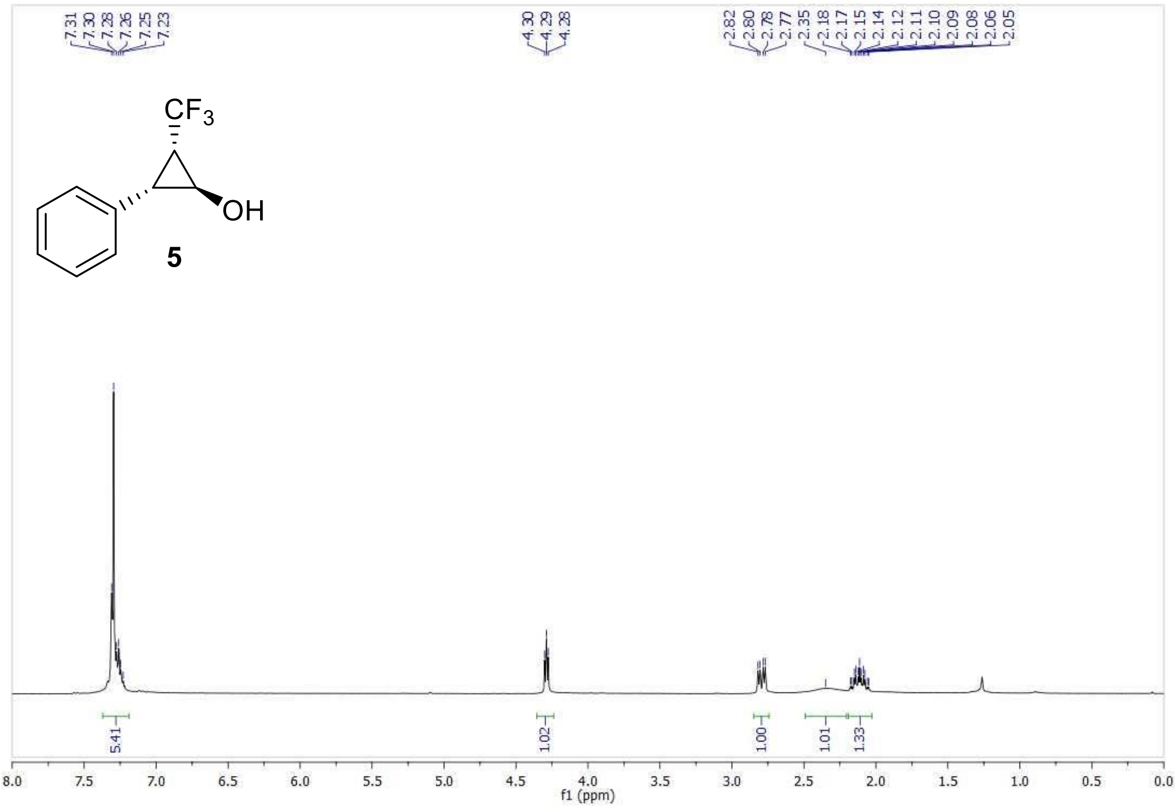

^{13}C NMR (75 MHz, $CDCl_3$)

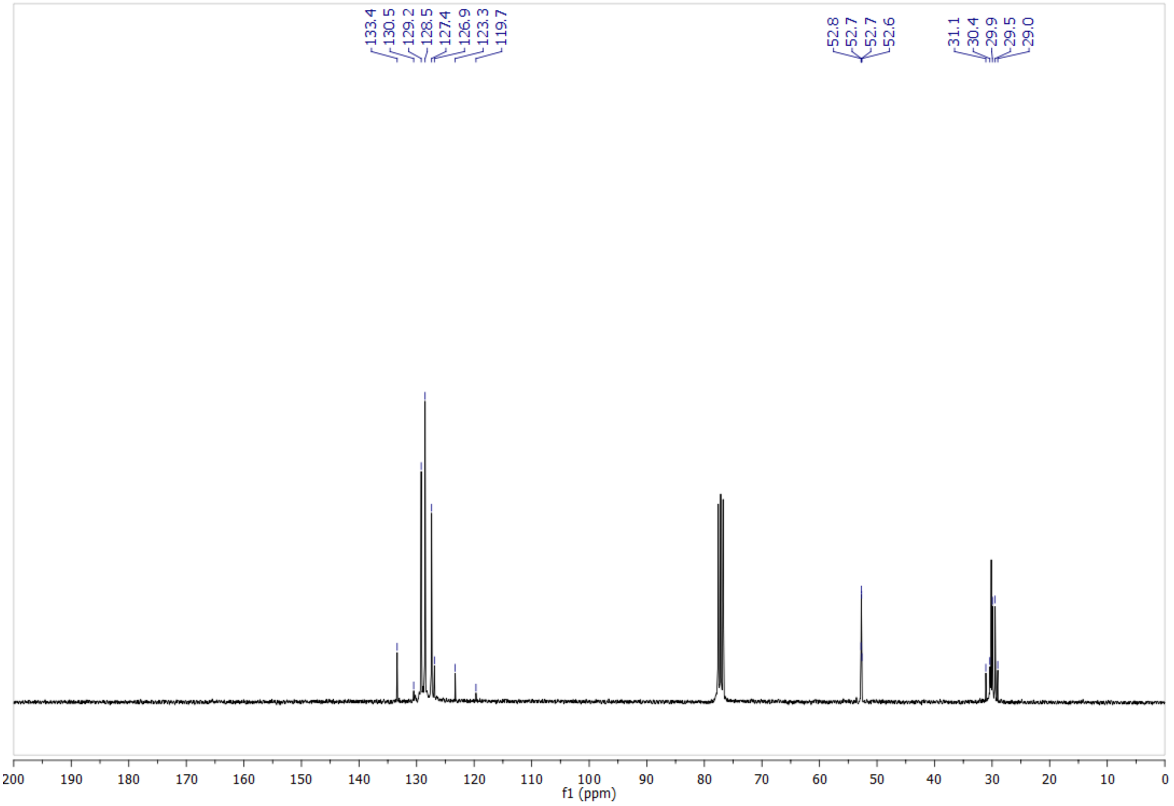

^{19}F NMR (282 MHz, $CDCl_3$)

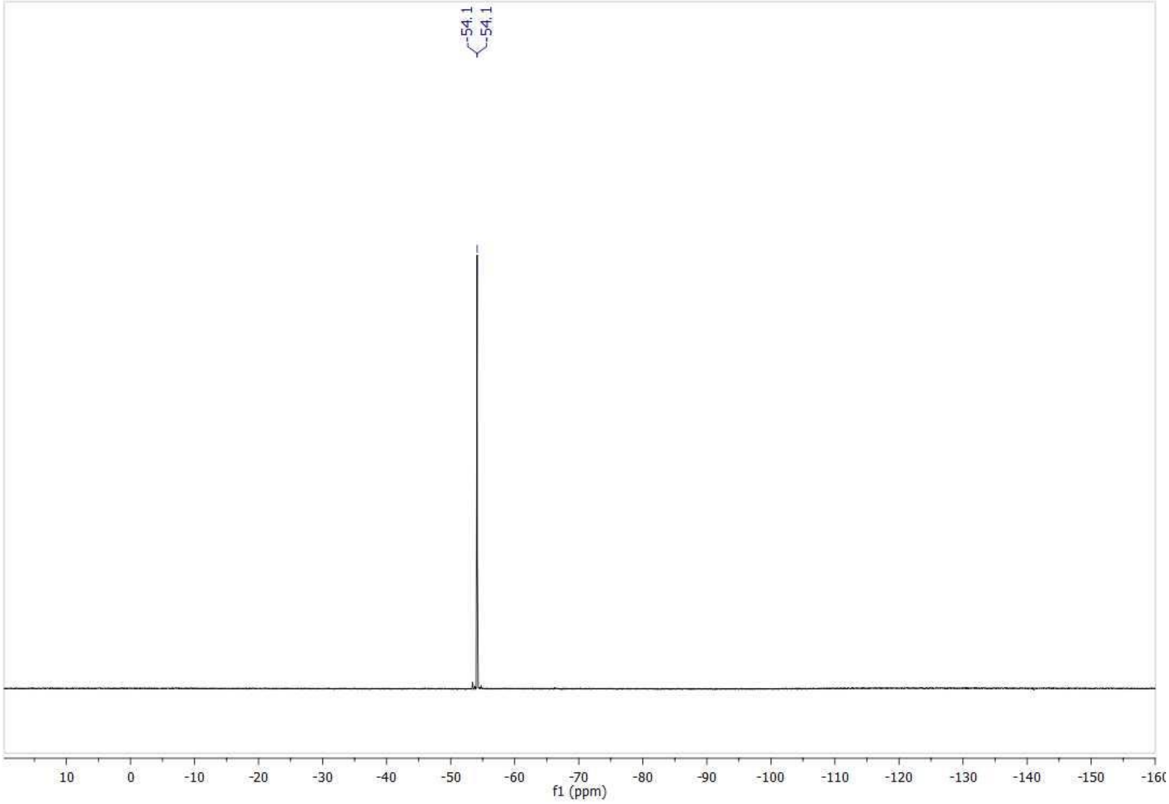

(1*S*,2*S*,3*S*)-*N*-Benzyl-2-phenyl-3-(trifluoromethyl)cyclopropan-1-amine (6)

^{1}H NMR (300 MHz, $CDCl_3$)

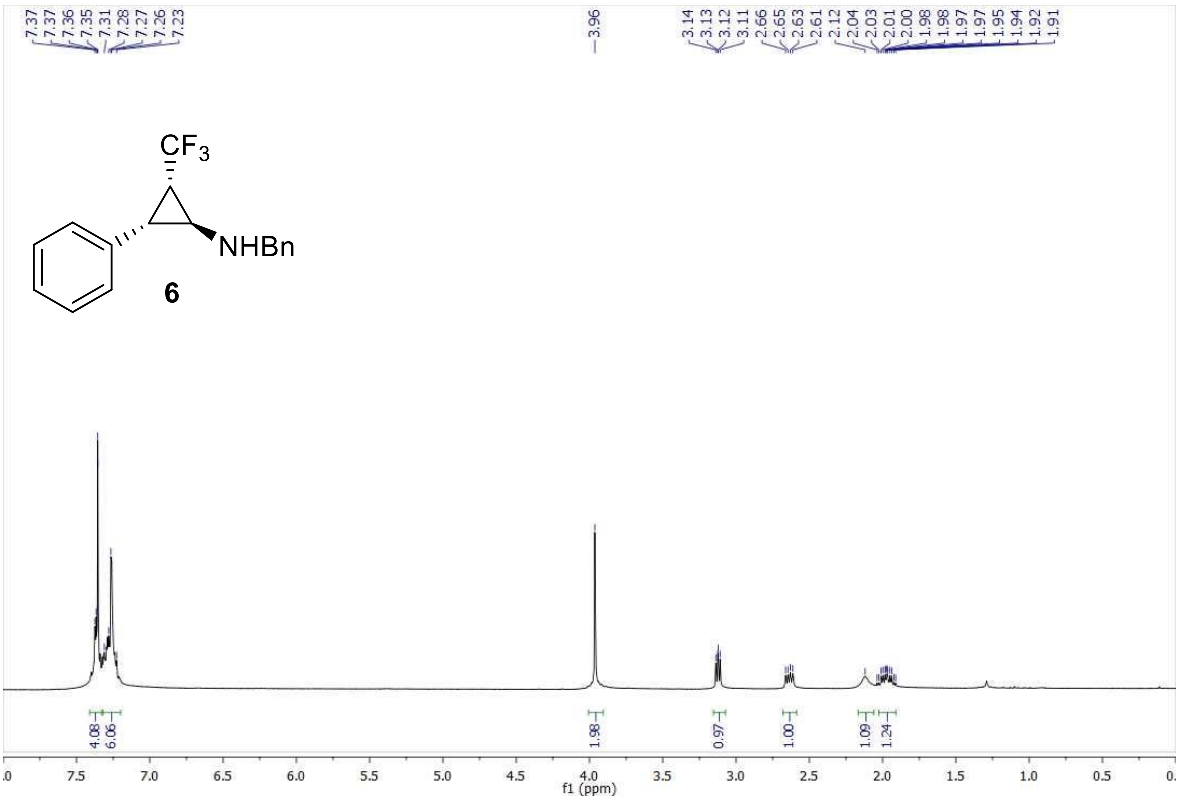

^{13}C NMR (126 MHz, $CDCl_3$)

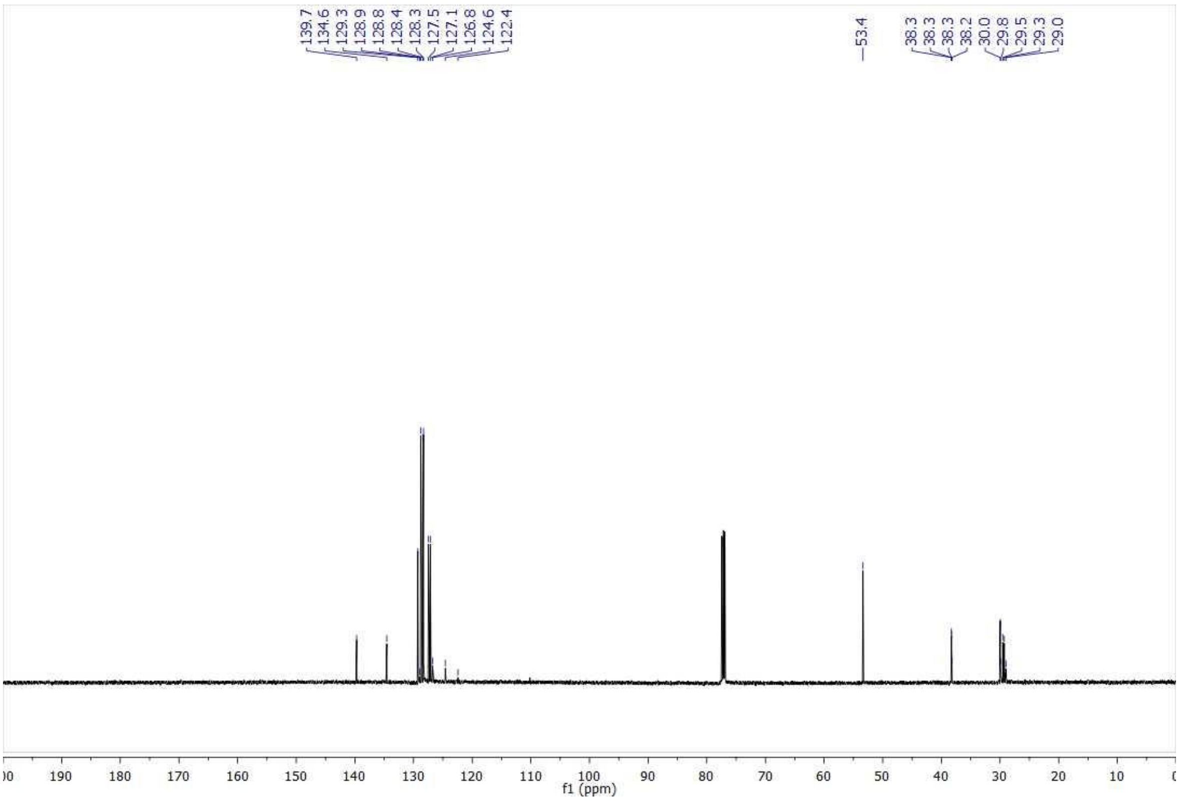

^{19}F NMR (282 MHz, $CDCl_3$)

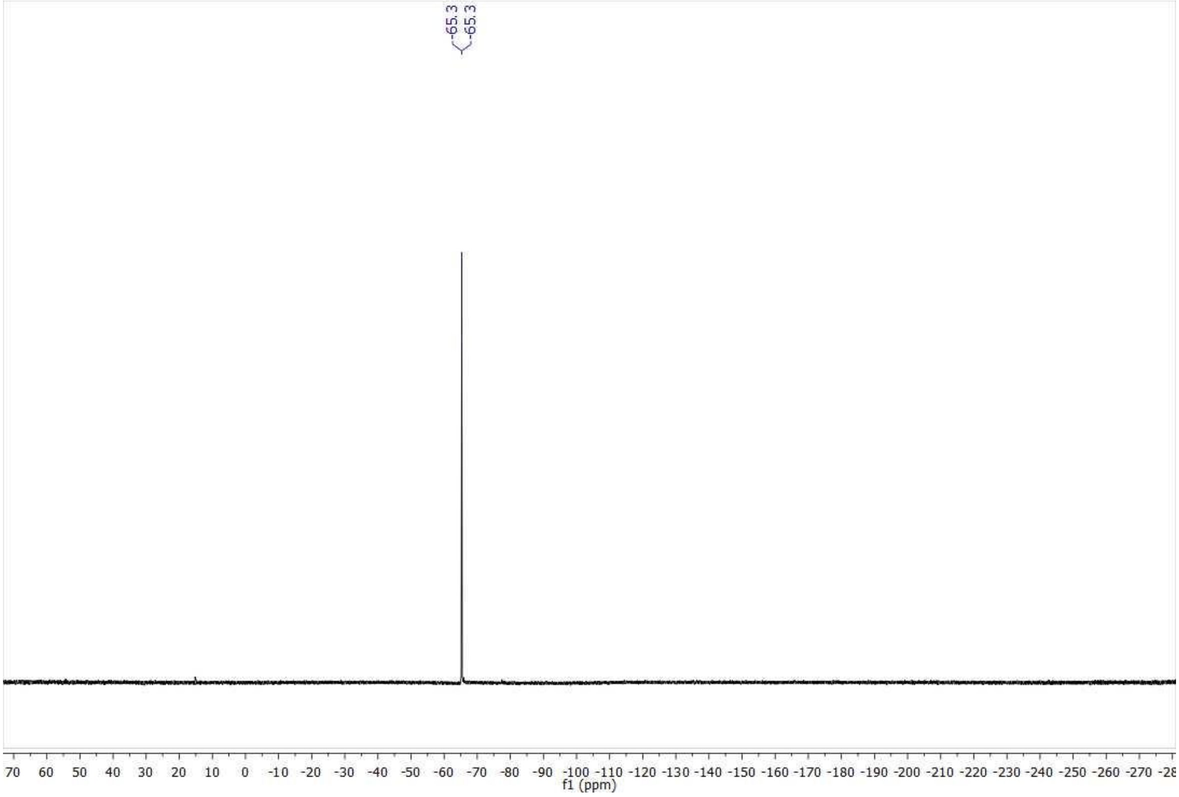

3-(Azidomethyl)-2-methoxypyridine (7)

^{1}H NMR (300 MHz, $CDCl_3$)

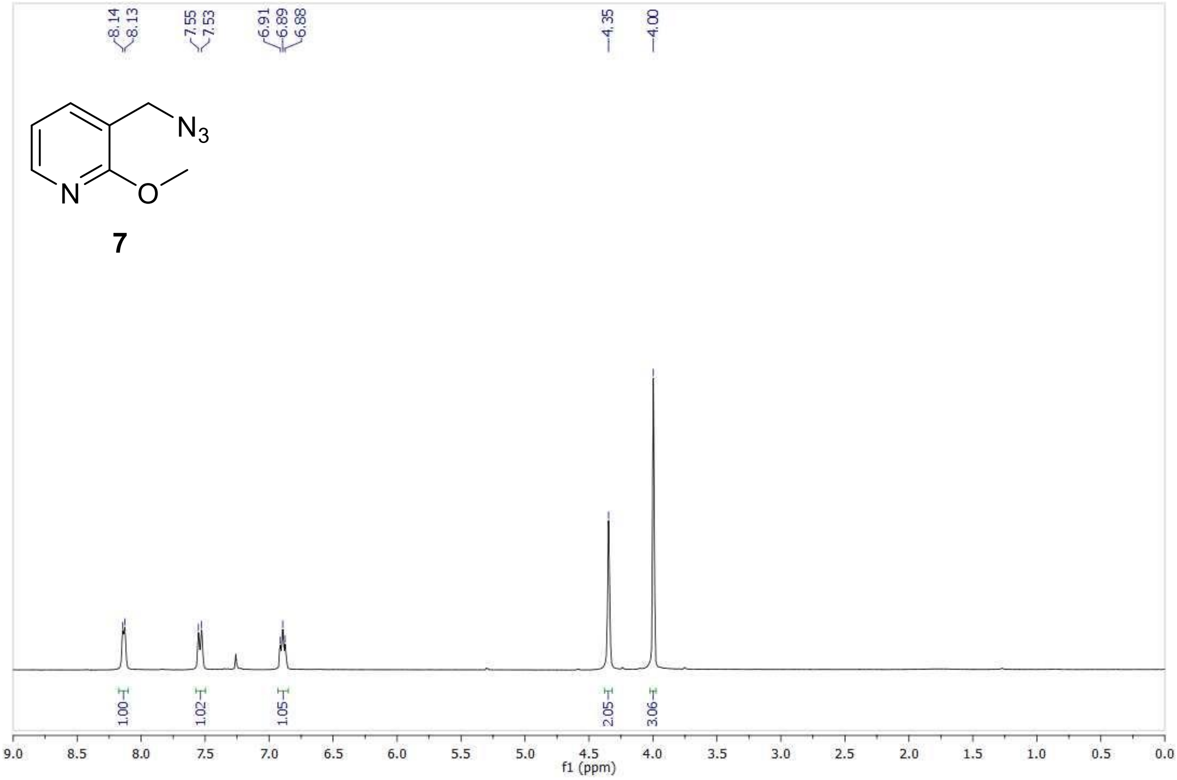

^{13}C NMR (75 MHz, $CDCl_3$)

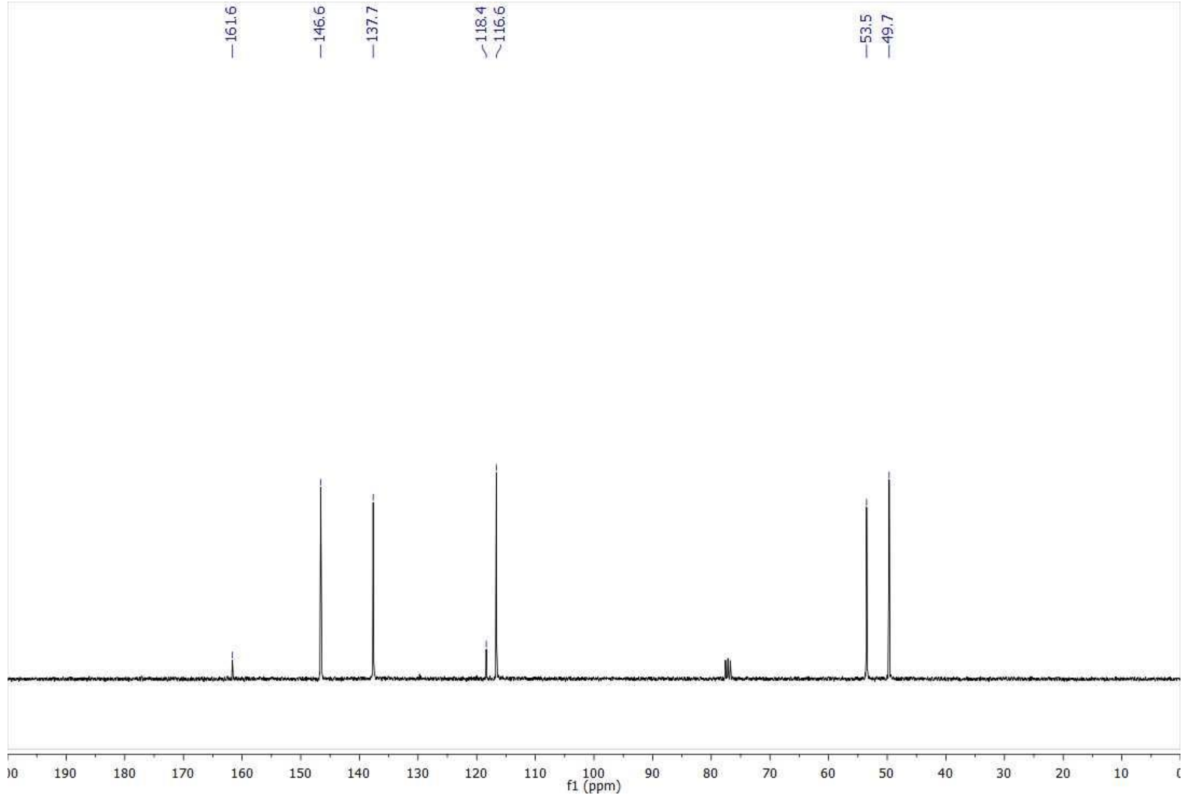

(1*S*,2*S*,3*S*)-*N*-((2-Methoxypyridin-3-yl)methyl)-2-phenyl-3-(trifluoromethyl)cyclopropan-1-amine (8)

^{1}H NMR (500 MHz, CDCl$_3$)

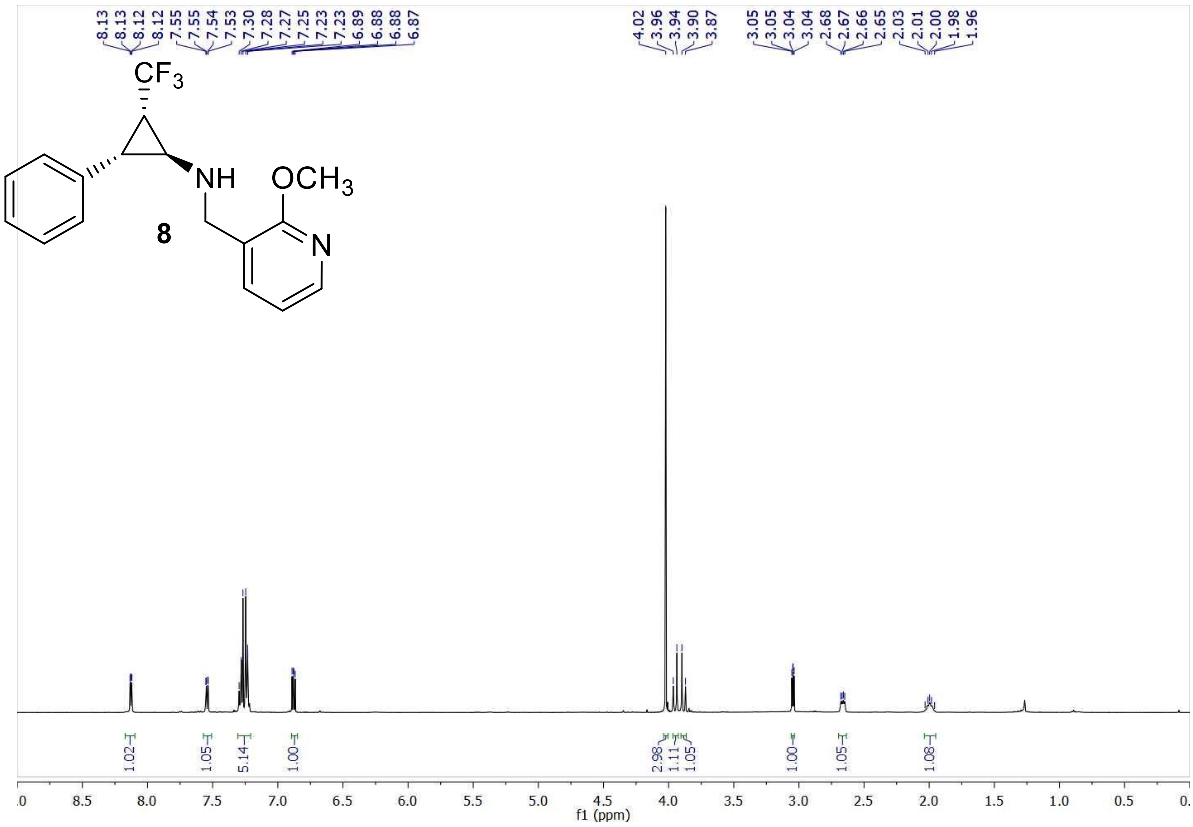

^{13}C NMR (126 MHz, CDCl$_3$)

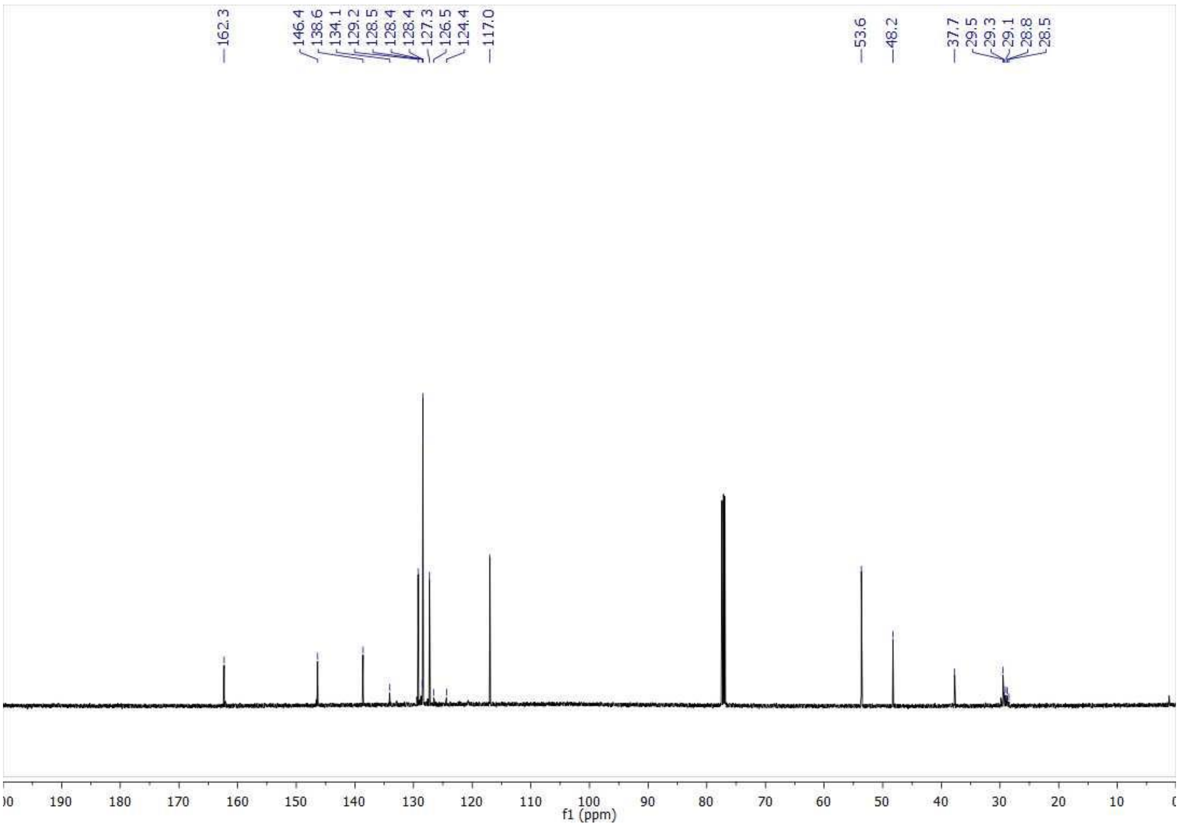

^{19}F NMR (282 MHz, $CDCl_3$)

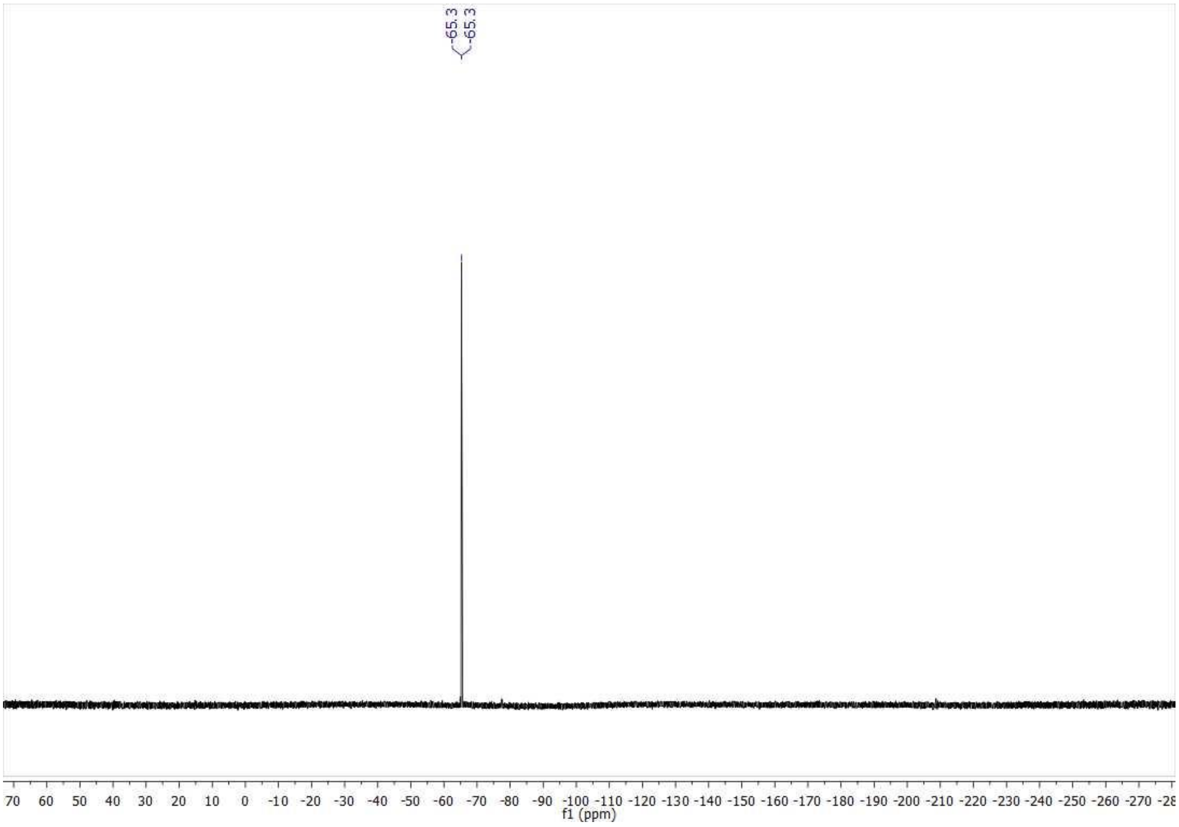

HPLC analyses of borocyclopropanes

2-((1*S*,2*S*,3*R*)-2-Phenyl-3-(trifluoromethyl)cyclopropyl)-4,4,5,5-tetramethyl-1,3,2-dioxoborolane (2a)

Chiral HPLC using Chiralpak® IB N-3 [Heptane/MTBE (99:1), 1 mL/min]

Copper(I)-bisoxazoline L3 catalyzed reaction

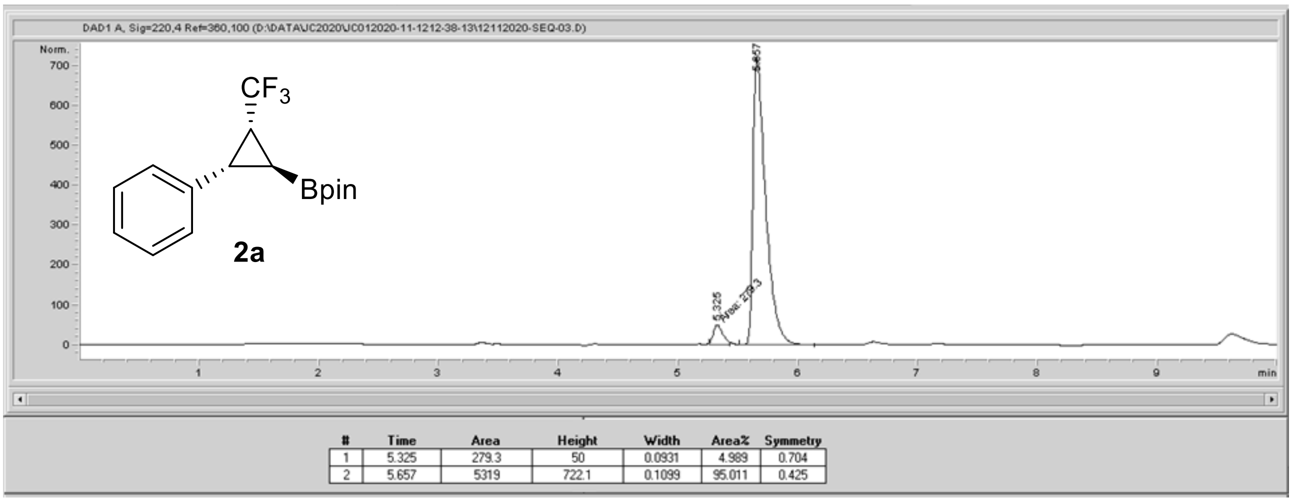

#	Time	Area	Height	Widht	Area%	Symmetry
**1**	5,325	279,3	50	0,0931	**4,989**	0,704
**2**	5,657	5319	722,1	0,1099	**95,011**	0,425

Pd(OAc)$_2$ catalyzed reaction

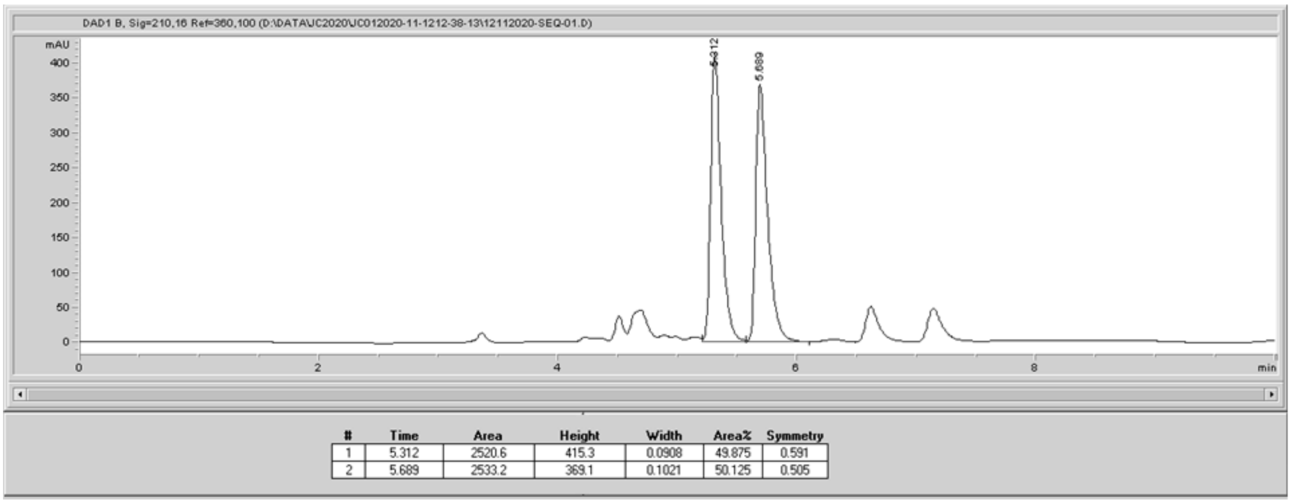

#	Time	Area	Height	Widht	Area%	Symmetry
**1**	5,312	2520,60	415,3	0,0908	49,875	0,591
**2**	5,689	2533,20	369,1	0,1021	50,125	0,505

Copper(I)-bisoxazoline L1 catalyzed reaction

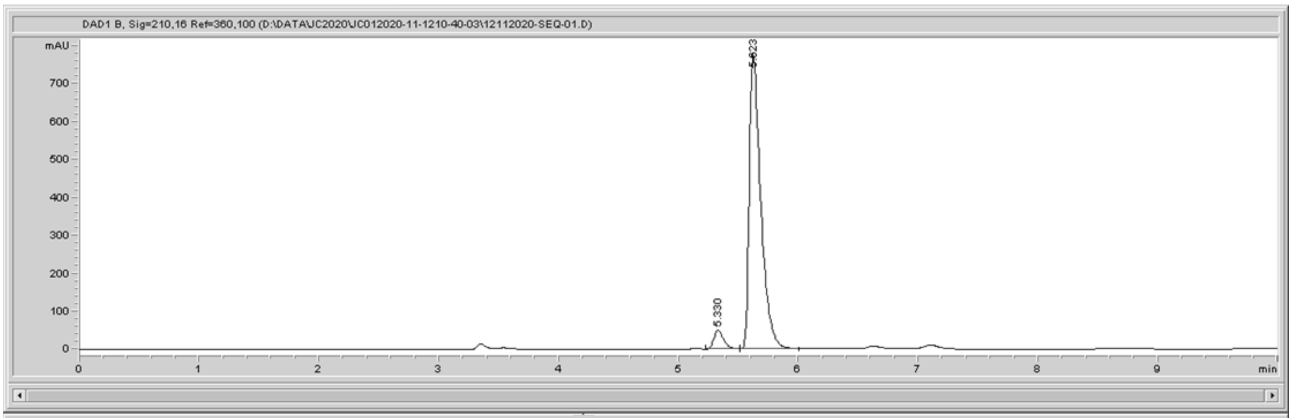

#	Time	Area	Height	Width	Area%	Symmetry
1	5.33	285.1	49.9	0.0867	5.191	0.689
2	5.623	5207.2	778.7	0.1	94.809	0.493

#	Time	Area	Height	Widht	Area%	Symmetry
**1**	5,33	285,1	49,9	0,0867	**5.191**	0,689
**2**	5,623	5207,2	778,7	0,1	**94,809**	0,493

Copper(I)-bisoxazoline L2 catalyzed reaction

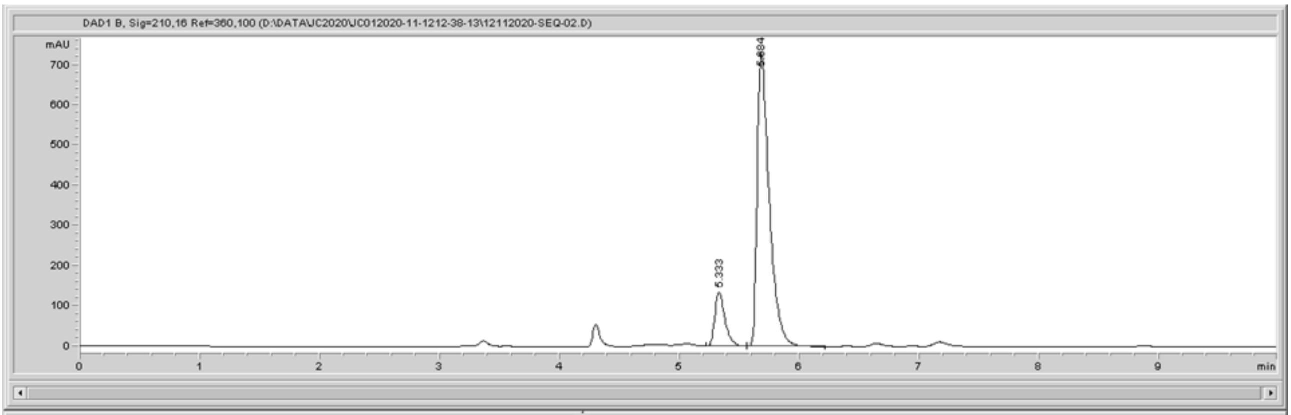

#	Time	Area	Height	Width	Area%	Symmetry
1	5.333	819	136.7	0.0899	13.673	0.649
2	5.684	5170.8	732.8	0.1063	86.327	0.46

#	Time	Area	Height	Widht	Area%	Symmetry
**1**	5,333	819	136,7	0,0899	**13,673**	0,649
**2**	5,684	5170,8	732,8	0,1063	**86,327**	0,46

2-((1*S*,2*S*,3*R*)-2-(2-Fluorophenyl)-3-(trifluoromethyl)cyclopropyl)-4,4,5,5-tetramethyl-1,3,2-dioxoborolane (2b)

Chiral HPLC using Chiralpak® IB N-3 [Heptane/MTBE (99:1), 1 mL/min]

Copper(I)-bisoxazoline L3 catalyzed reaction

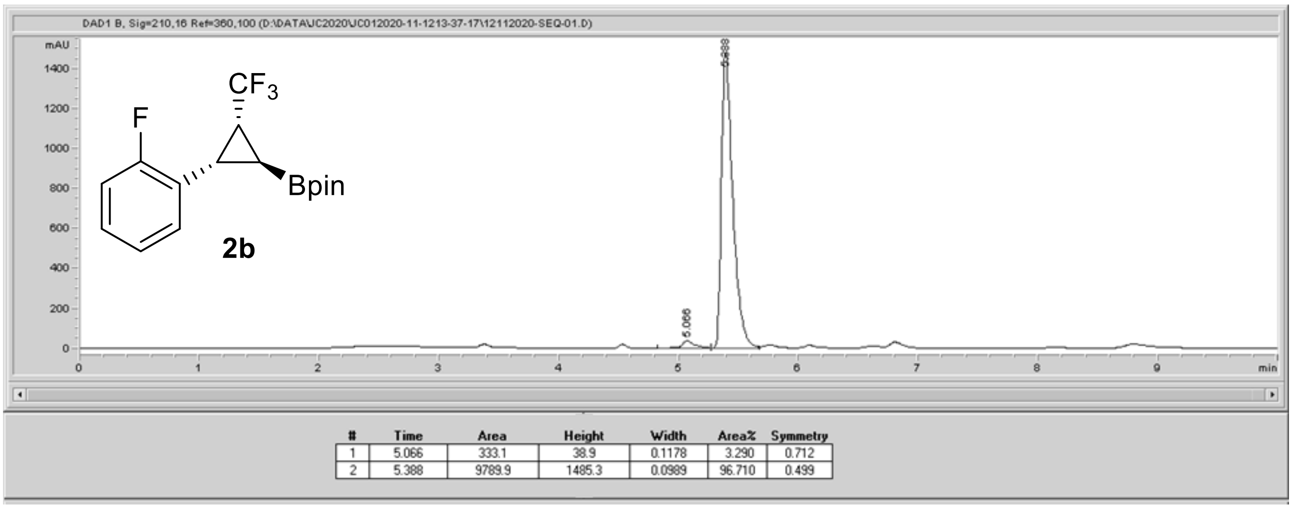

#	Time	Area	Height	Widht	Area%	Symmetry
**1**	5,066	333,1	38,9	0,1178	**3,290**	0,712
**2**	5,388	9789,9	1485,3	0,0989	**96,710**	0,499

Pd(OAc)$_2$ catalyzed reaction

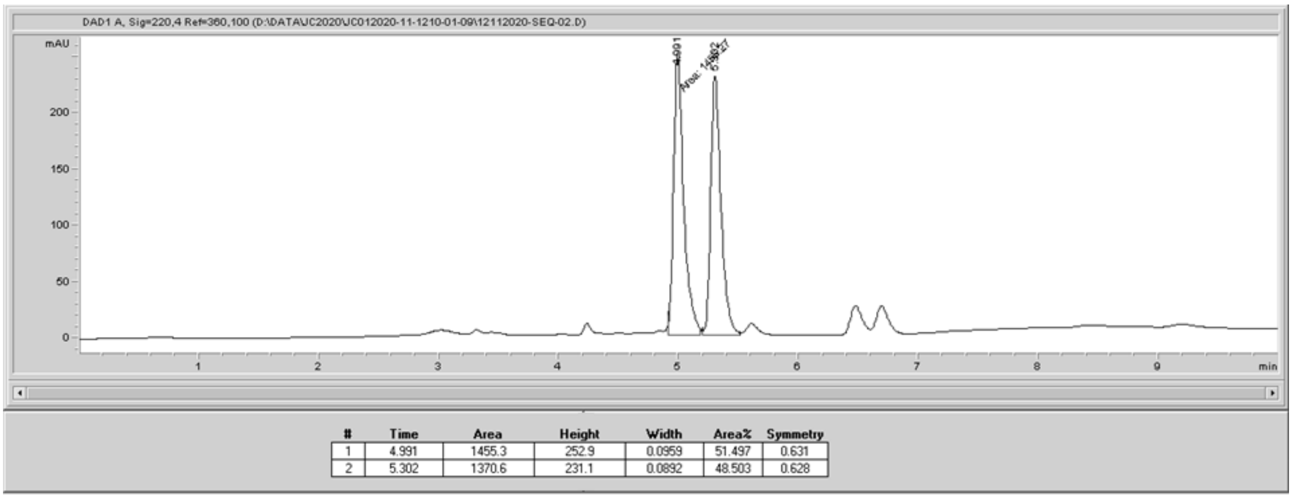

#	Time	Area	Height	Widht	Area%	Symmetry
**1**	4,991	1455,3	252,9	0,0959	**51,497**	0,631
**2**	5,302	1370,6	231,1	0,0892	**48,503**	0,628

2-((1*S*,2*S*,3*R*)-2-(*o*-Tolyl)-3-(trifluoromethyl)cyclopropyl)-4,4,5,5-tetramethyl-1,3,2-dioxoborolane (2c)

Chiral HPLC using Chiralpak® IB N-3 [MeOH/H_2O (75:25), 0.8 mL/min]

Copper(I)-bisoxazoline L3 catalyzed reaction

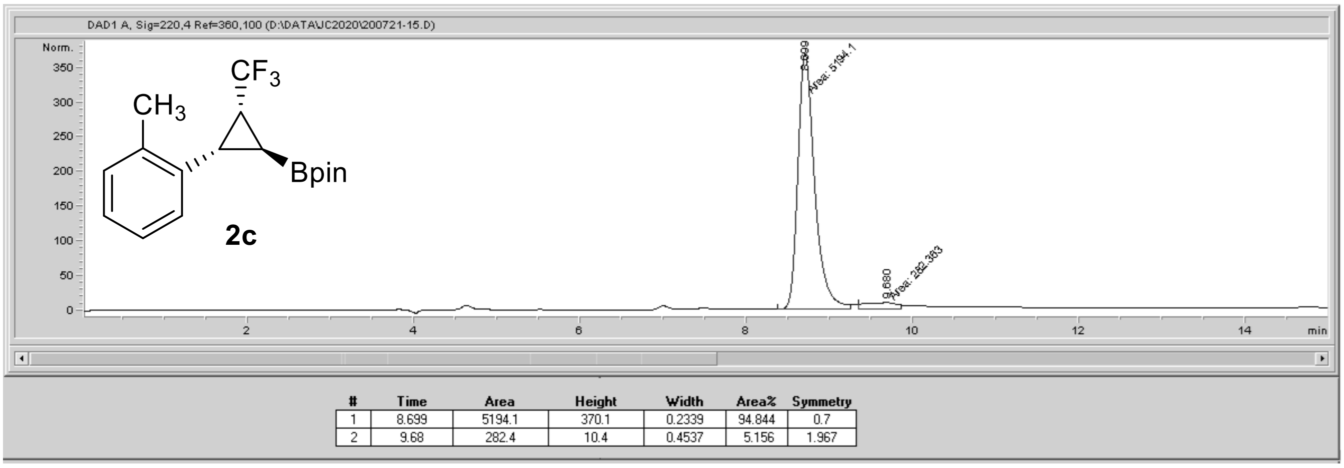

#	Time	Area	Height	Width	Area%	Symmetry
1	8.699	5194.1	370.1	0.2339	94.844	0.7
2	9.68	282.4	10.4	0.4537	5.156	1.967

#	Time	Area	Height	Widht	Area%	Symmetry
**1**	8,699	5194,1	370,1	0,2339	**94,844**	0,7
**2**	9,68	282,4	10,4	0,4537	**5,156**	1,967

Pd(OAc)$_2$ catalyzed reaction

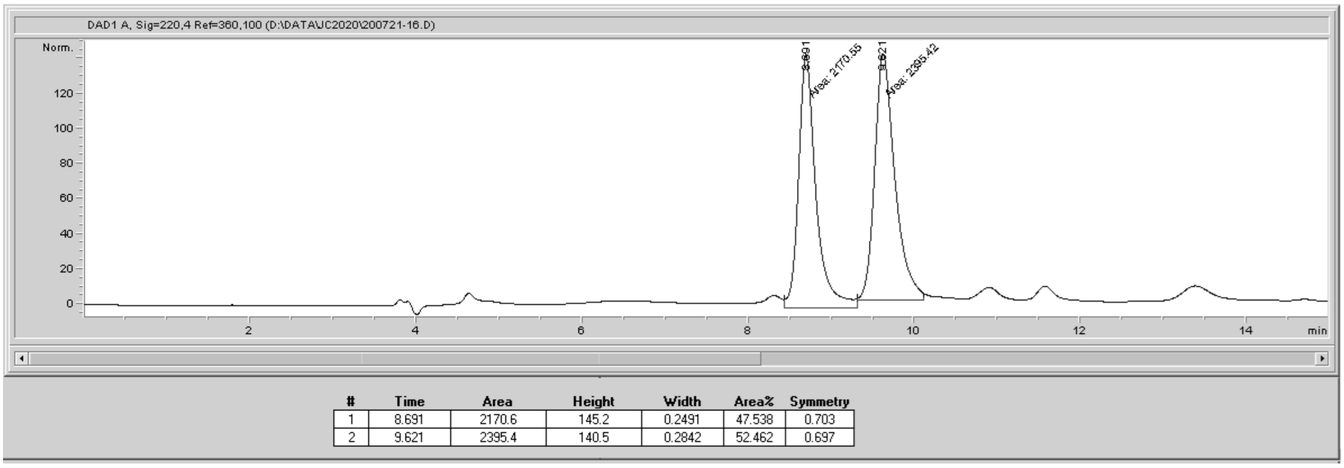

#	Time	Area	Height	Width	Area%	Symmetry
1	8.691	2170.6	145.2	0.2491	47.538	0.703
2	9.621	2395.4	140.5	0.2842	52.462	0.697

#	Time	Area	Height	Widht	Area%	Symmetry
**1**	8,691	2170,6	145,2	0,2491	**47,538**	0,703
**2**	9,621	2395,4	140,5	0,2842	**52,462**	0,697

2-((1*S*,2*S*,3*R*)-2-(*m*-Tolyl)-3-(trifluoromethyl)cyclopropyl)-4,4,5,5-tetramethyl-1,3,2-dioxoborolane (2d)

Chiral HPLC using Chiralpak® IB N-3 [H_2O/ACN (60:40), 1 mL/min]

Copper(I)-bisoxazoline L3 catalyzed reaction

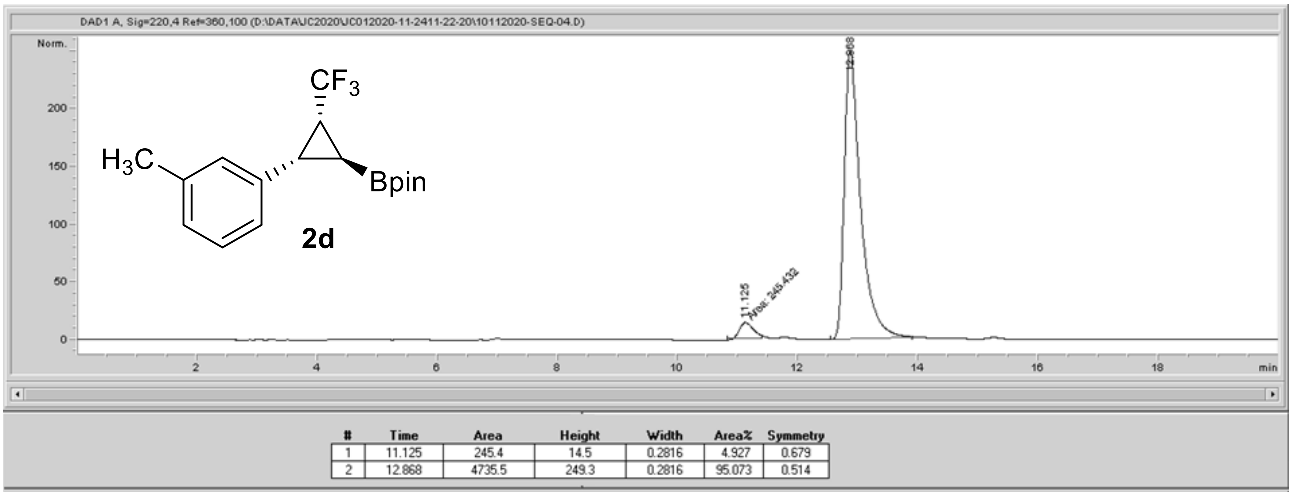

#	Time	Area	Height	Widht	Area%	Symmetry
**1**	11,125	245,4	14,5	0,2816	**4,927**	0,679
**2**	12,868	4735,5	249,3	0,2816	**95,073**	0,514

Pd(OAc)$_2$ catalyzed reaction

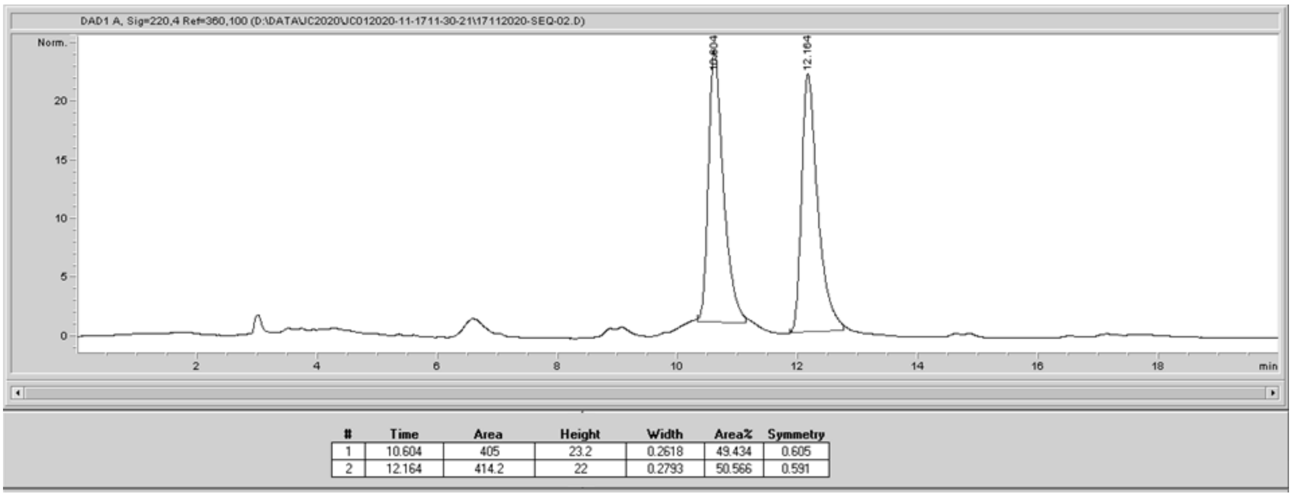

#	Time	Area	Height	Widht	Area%	Symmetry
**1**	10,604	405	23,2	0,2618	**49,434**	0,605
**2**	12,164	414,2	22	0,2793	**50,566**	0,591

2-((1*S*,2*S*,3*R*)-2-(3-Fluorophenyl)-3-(trifluoromethyl)cyclopropyl)-4,4,5,5-tetramethyl-1,3,2-dioxoborolane (2e)

Chiral HPLC using Chiralpak® IB N-3 [MeOH/H_2O (60:40), 0.7 mL/min]

Copper(I)-bisoxazoline L3 catalyzed reaction

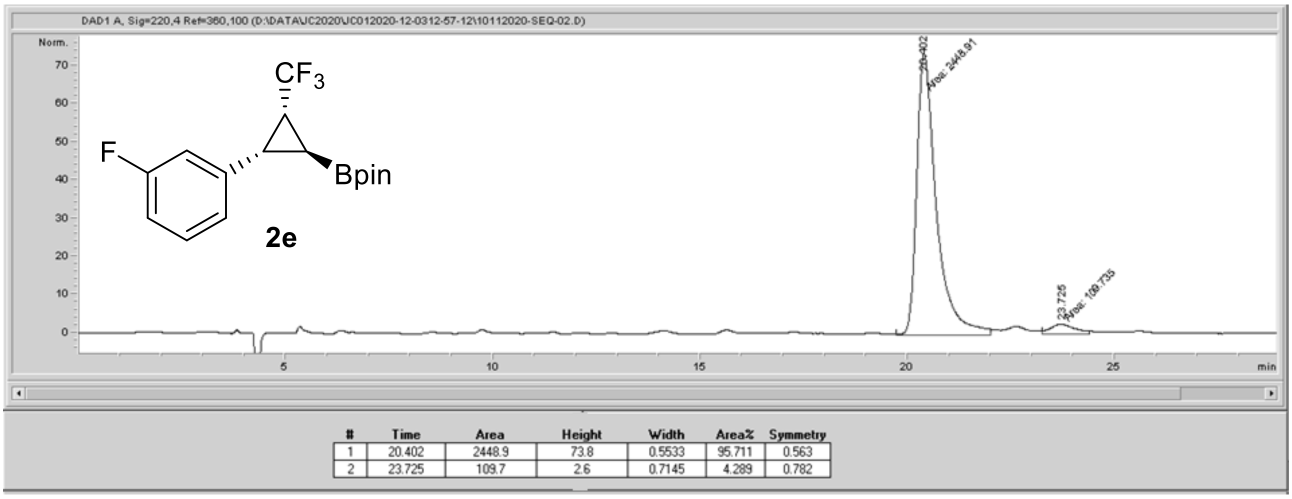

#	Time	Area	Widht	Height	Area%	Symmetry

#	Time	Area	Height	Widht	Area%	Symmetry
**1**	20,402	2448,9	73,8	0,5533	**95,711**	0,563
**2**	23,725	109,7	2,6	0,7145	**4,289**	0,782

Pd(OAc)$_2$ catalyzed reaction

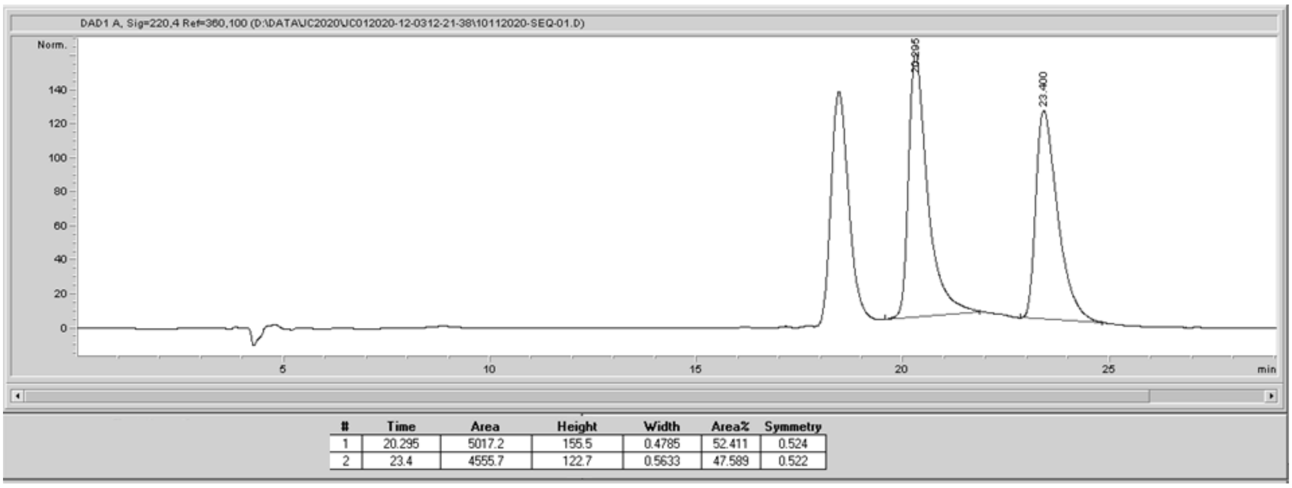

#	Time	Area	Height	Widht	Area%	Symmetry
**1**	20,295	5017,2	155,5	0,4785	**52,411**	0,524
**2**	23,4	4555,7	122,7	0,5633	**47,589**	0,522

3-((1*S*,2*S*,3*R*)-2--4,4,5,5-Tetramethyl-1,3,2-dioxoborolane-3-(trifluoromethyl)cyclopropyl) phenyl acetate (2f)

Chiral HPLC using Chiralpak® IB N-3 [Heptane/MTBE (97:3), 1 mL/min]

Copper(I)-bisoxazoline L3 catalyzed reaction

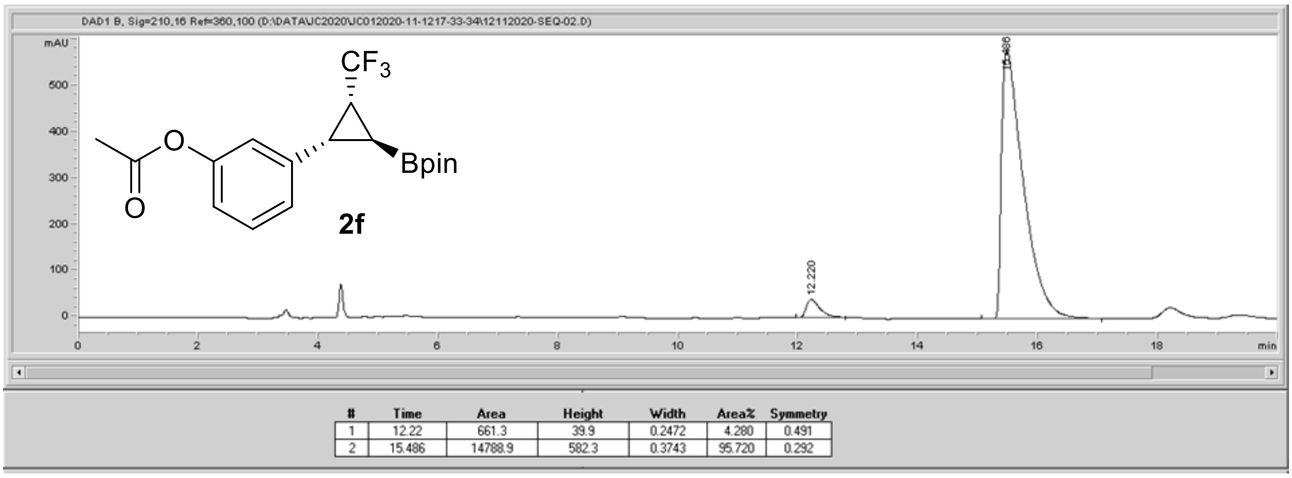

#	Time	Area	Height	Widht	Area%	Symmetry
**1**	12,22	661,3	39,9	0,2472	**4,280**	0,491
**2**	15,486	14788,9	582,3	0,3743	**95,720**	0,292

Pd(OAc)$_2$ catalyzed reaction

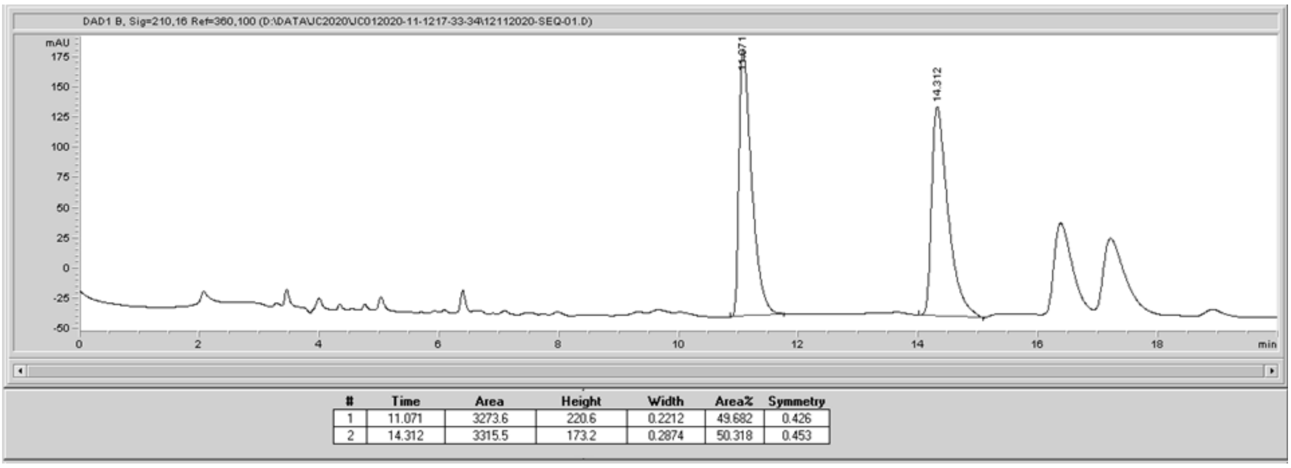

#	Time	Area	Height	Widht	Area%	Symmetry
**1**	11,071	3273,6	220,6	0,2212	**49,682**	0,426
**2**	14,312	3315,5	173,2	0,2874	**50,318**	0,453

2-((1*S*,2*S*,3*R*)-2-(*p*-Tolyl)-3-(trifluoromethyl)cyclopropyl)-4,4,5,5-tetramethyl-1,3,2-dioxoborolane (2g)

Chiral HPLC using Chiralpak® IB N-3 [MeOH/H_2O (80:20), 0.9 mL/min]

Copper(I)-bisoxazoline L3 catalyzed reaction

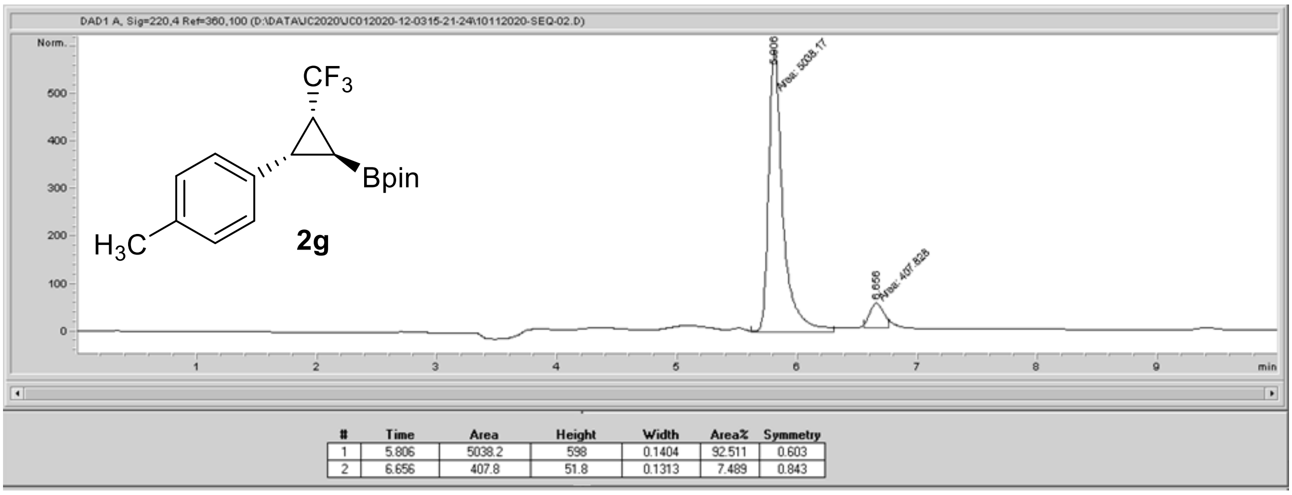

#	Time	Area	Height	Widht	Area%	Symmetry
**1**	5,806	5038,2	598	0,1404	**92,511**	0,603
**2**	6,656	407,8	51,8	0,1313	**7,489**	0,843

Pd(OAc)$_2$ catalyzed reaction

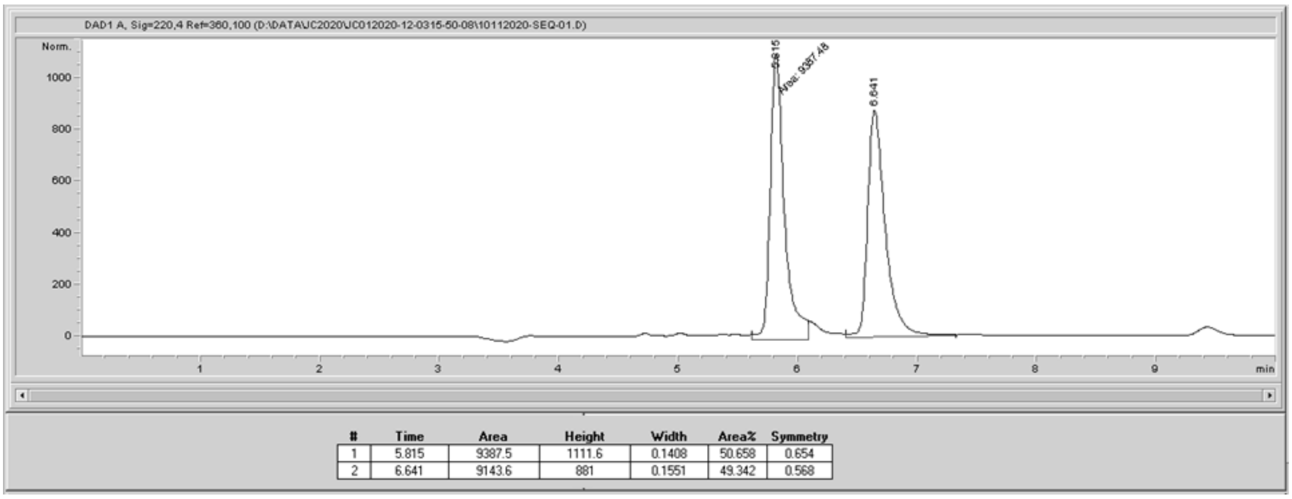

#	Time	Area	Height	Widht	Area%	Symmetry
**1**	5,815	9387,5	1111,6	0,1408	**50,658**	0,654
**2**	6,641	9143,6	881	0,1551	**49,342**	0,568

2-((1*S*,2*S*,3*R*)-2-(*p*-Chlorophenyl)-3-(trifluoromethyl)cyclopropyl)-4,4,5,5-tetramethyl-1,3,2-dioxoborolane (2h)

Chiral HPLC using Chiralpak® IB N-3 [H_2O/ACN (60:40), 1 mL/min]

Copper(I)-bisoxazoline L3 catalyzed reaction

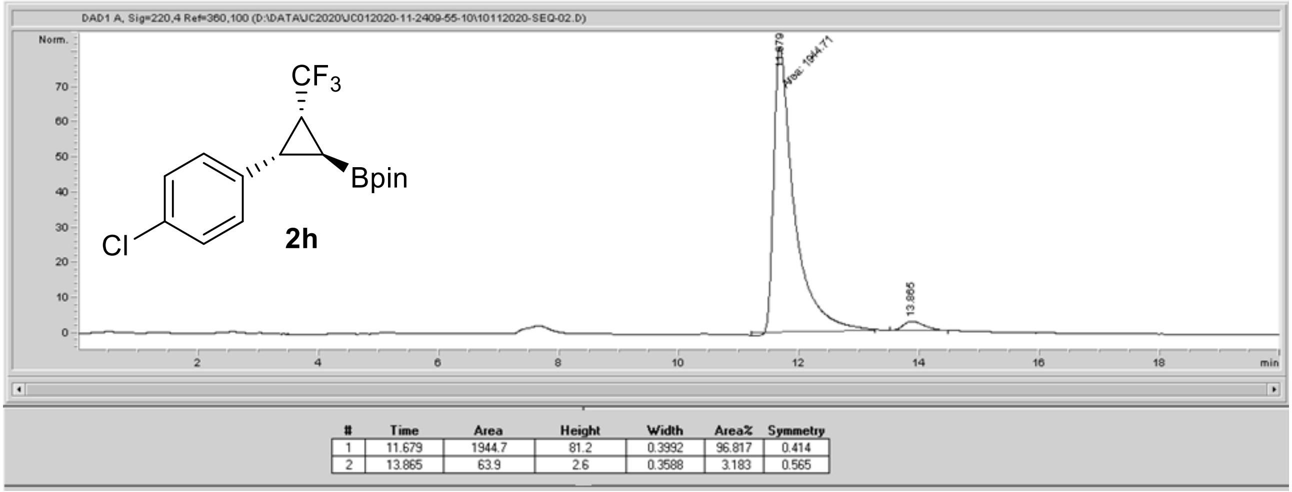

#	Time	Area	Height	Widht	Area%	Symmetry
**1**	11,679	1944,7	81,2	0,3992	**96,817**	0,414
**2**	13,865	63,9	2,6	0,3588	**3,183**	0,565

Pd(OAc)$_2$ catalyzed reaction

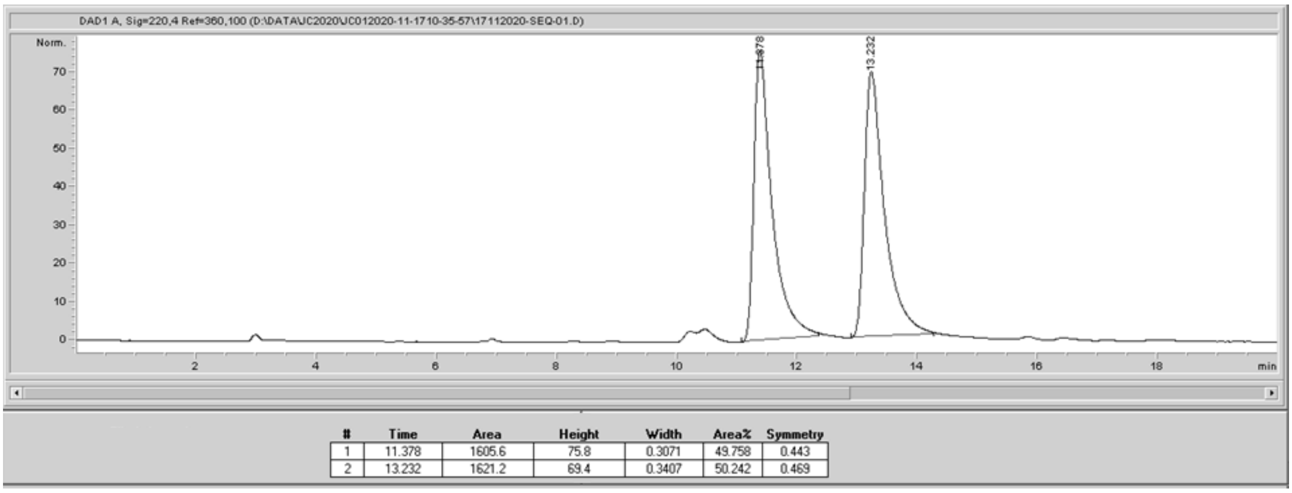

#	Time	Area	Height	Widht	Area%	Symmetry
**1**	11,378	1605,6	75,8	0,3071	**49,758**	0,443
**2**	13,232	1621,2	69,4	0,3407	**50,242**	0,469

2-((1*S*,2*S*,3*R*)-2-(*p*-Bromophenyl)-3-(trifluoromethyl)cyclopropyl)-4,4,5,5-tetramethyl-1,3,2-dioxoborolane (2i)

Chiral HPLC using Chiralpak® IB N-3 [H_2O/ACN (60:40), 1 mL/min]

Copper(I)-bisoxazoline L3 catalyzed reaction

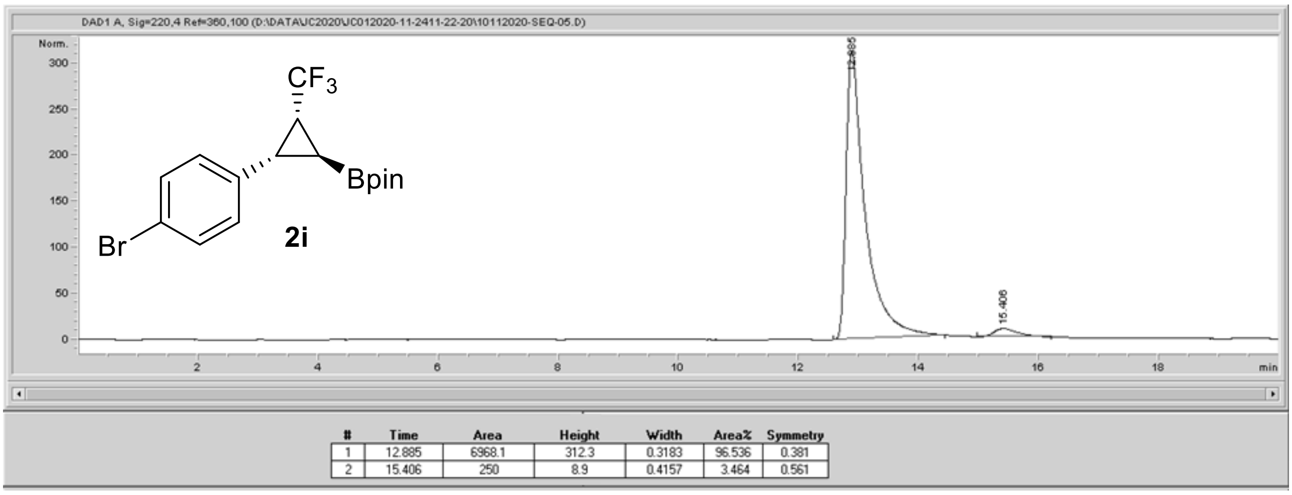

#	Time	Area	Height	Widht	Area%	Symmetry
**1**	12,885	6968,1	312,3	0,3183	**96,536**	0,381
**2**	15,406	250	8,9	0,4157	**3,464**	0,561

$Pd(OAc)_2$ was not compatible with the alkenyl boronate **1i** to obtain the racemic sample.

2-((1*S*,2*S*,3*R*)-2-(*p*-Trifluoromethylphenyl)-3-(trifluoromethyl)cyclopropyl)-4,4,5,5-tetramethyl-1,3,2-dioxoborolane (2j)

Chiral HPLC using Chiralpak® IB N-3 [H_2O/ACN (60:40), 1 mL/min]

Copper(I)-bisoxazoline L3 catalyzed reaction

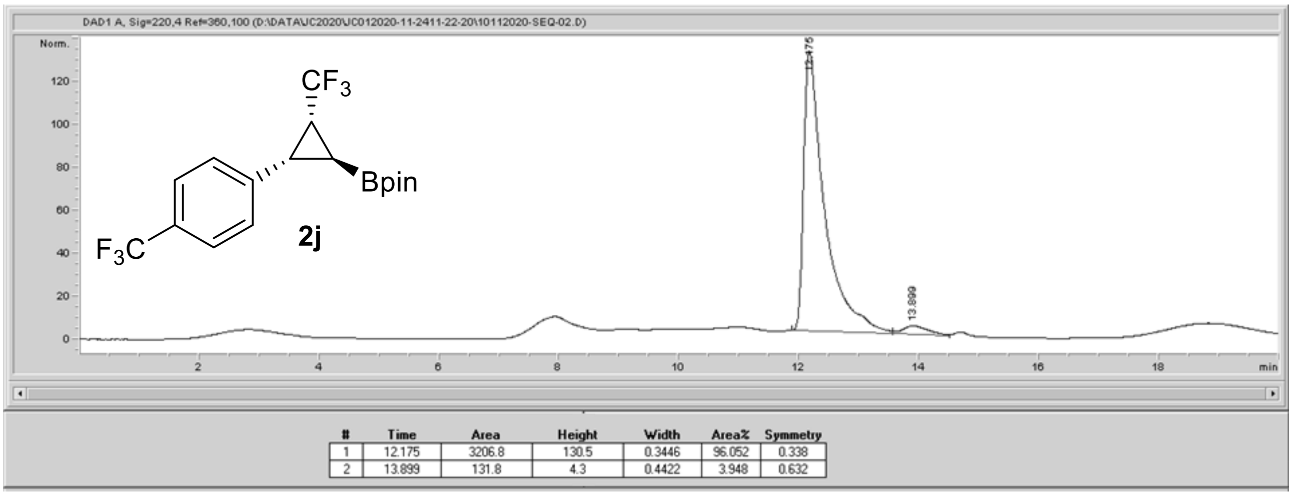

#	Time	Area	Height	Widht	Area%	Symmetry
**1**	12,175	3206,8	130,5	0,3446	**96,052**	0,338
**2**	13,899	131,8	4,3	0,4422	**3,948**	0,632

Pd(OAc)$_2$ catalyzed reaction

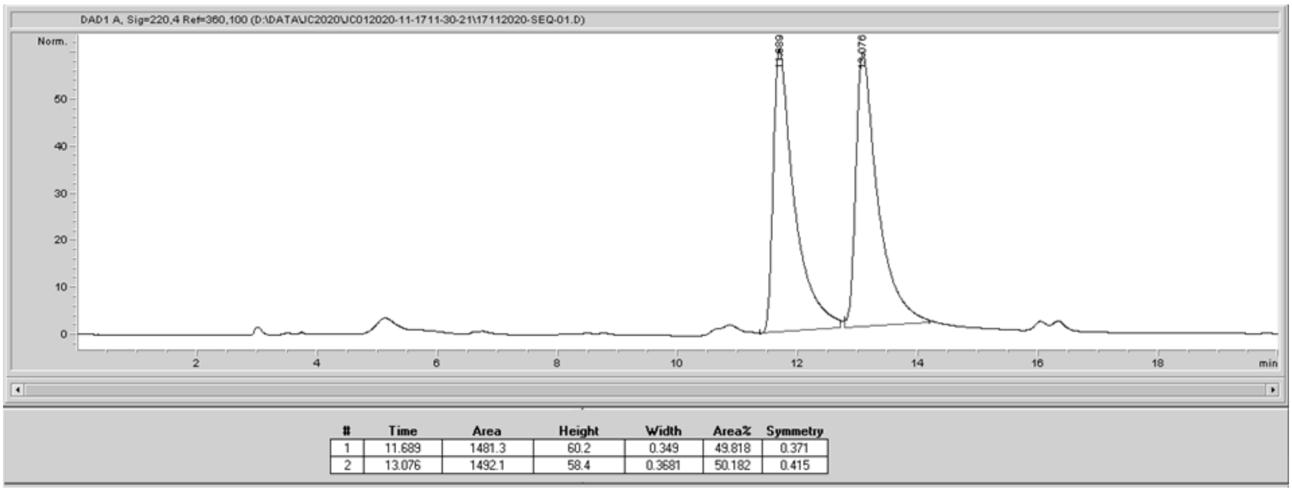

#	Time	Area	Height	Widht	Area%	Symmetry
**1**	11,689	1481,3	60,2	0,349	49,818	0,371
**2**	13,076	1492,1	58,4	0,3681	50,182	0,415

2-((1*S*,2*S*,3*R*)-2-(*p*-*Tert*butylphenyl)-3-(trifluoromethyl)cyclopropyl)-4,4,5,5-tetramethyl-1,3,2-dioxoborolane (2k)

Chiral HPLC using Chiralpak® IB N-3 [MeOH/H_2O (80:20), 0.9 mL/min]

Copper(I)-bisoxazoline L3 catalyzed reaction

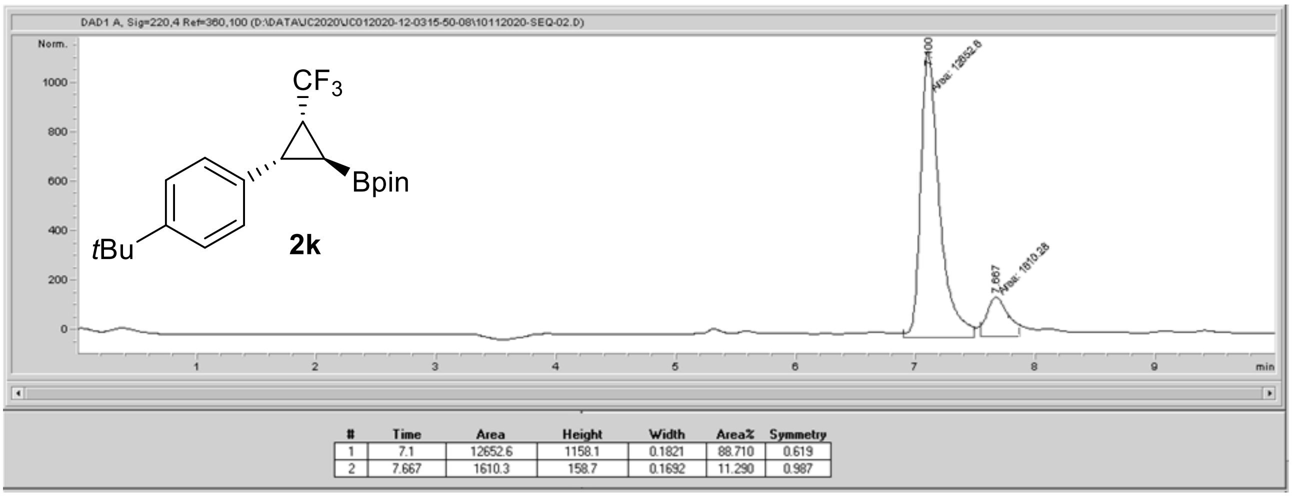

#	Time	Area	Height	Widht	Area%	Symmetry
**1**	7,1	12652,6	1158,1	0,1821	**88,710**	0,619
**2**	7,667	1610,3	158,7	0,1692	**11,290**	0,987

Pd(OAc)₂ catalyzed reaction

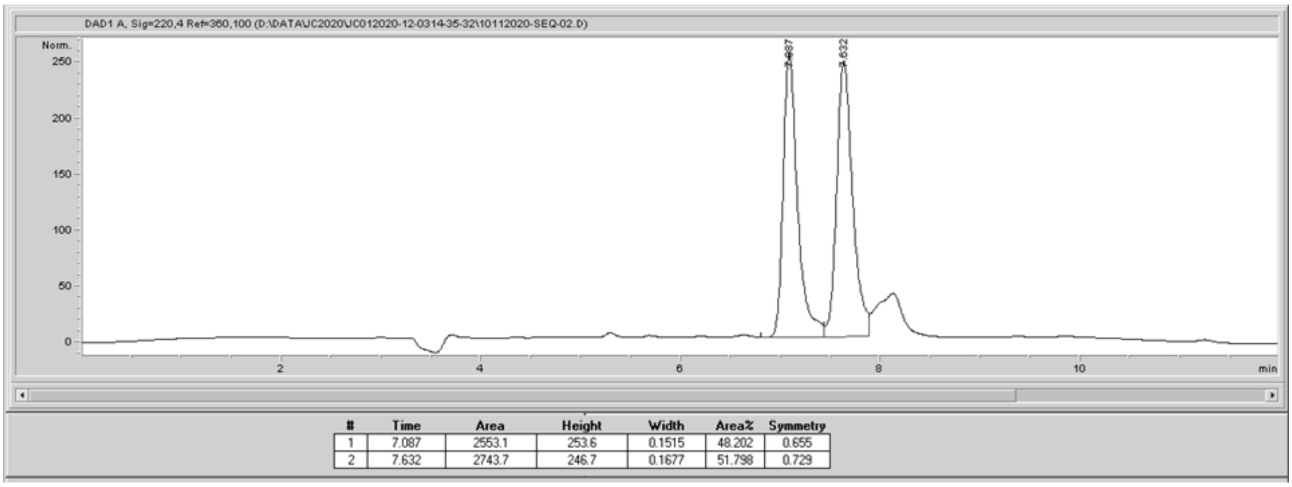

#	Time	Area	Height	Widht	Area%	Symmetry
**1**	7,087	2553,1	253,6	0,1515	**48,202**	0,655
**2**	7,632	2743,7	246,7	0,1677	**51,798**	0,729

2-((1*S*,2*S*,3*R*)-2-(*p*-Methoxy)-3-(trifluoromethyl)cyclopropyl)-4,4,5,5-tetramethyl-1,3,2-dioxoborolane (2l)

Chiral HPLC using Chiralpak® IB N-3 [H_2O/ACN (60:40), 1 mL/min]

Copper(I)-bisoxazoline L3 catalyzed reaction

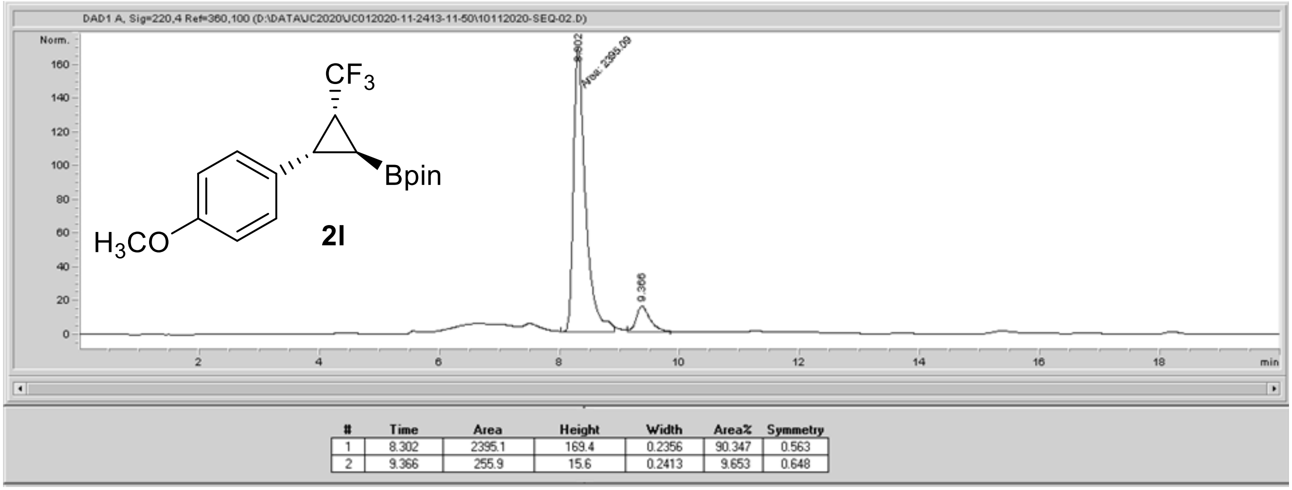

#	Time	Area	Height	Widht	Area%	Symmetry
**1**	8,302	2395,1	169,4	0,2356	**90,347**	0,563
**2**	9,366	255,9	15,6	0,2413	**9,653**	0,648

$Pd(OAc)_2$ catalyzed reaction

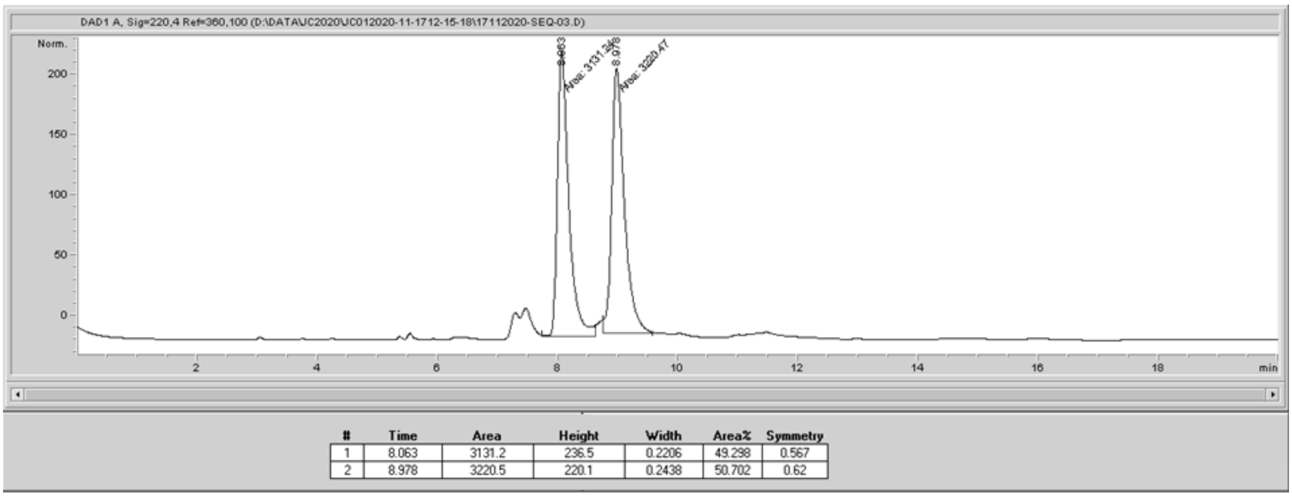

#	Time	Area	Height	Widht	Area%	Symmetry
**1**	8,063	3131,2	236,5	0,2206	**49,298**	0,567
**2**	8,978	3220,5	220,1	0,2438	**50,702**	0,62

Methyl 4-((1*S*,2*S*,3*R*)-2-(4,4,5,5-tetramethyl-1,3,2-dioxoborolan-2-yl)-3-(trifluoromethyl)cyclopropyl)benzoate (2o)

Chiral HPLC using Chiralpak® IB N-3 [MeOH/H_2O (80:20), 0.75 mL/min]

Copper(I)-bisoxazoline L3 catalyzed reaction

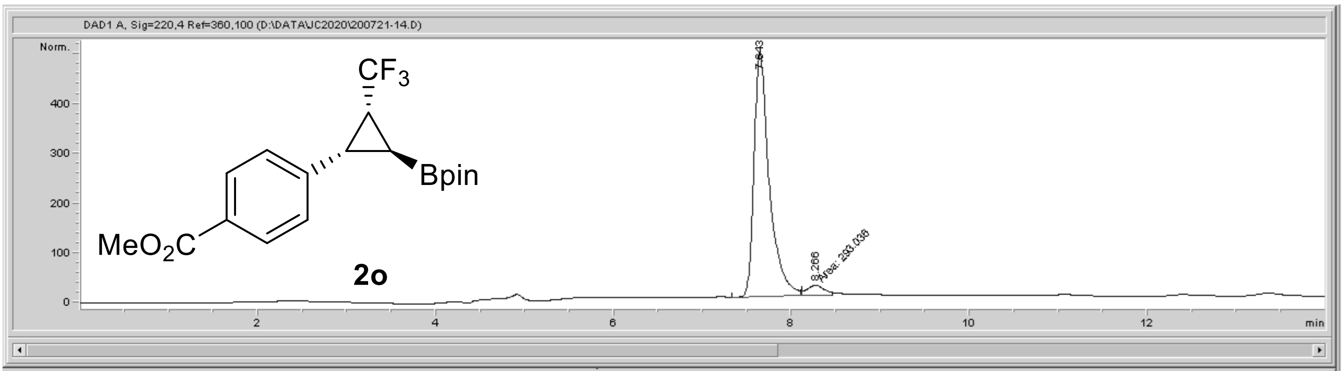

#	Time	Area	Height	Width	Area%	Symmetry
1	7.643	5912.7	490.8	0.1765	95.278	0.581
2	8.266	293	21	0.233	4.722	0.817

#	Time	Area	Height	Widht	Area%	Symmetry
**1**	7,643	5912,7	490,8	0,1765	**95,278**	0,581
**2**	8,266	293	21	0,233	**4,722**	0,817

Pd(OAc)$_2$ catalyzed reaction

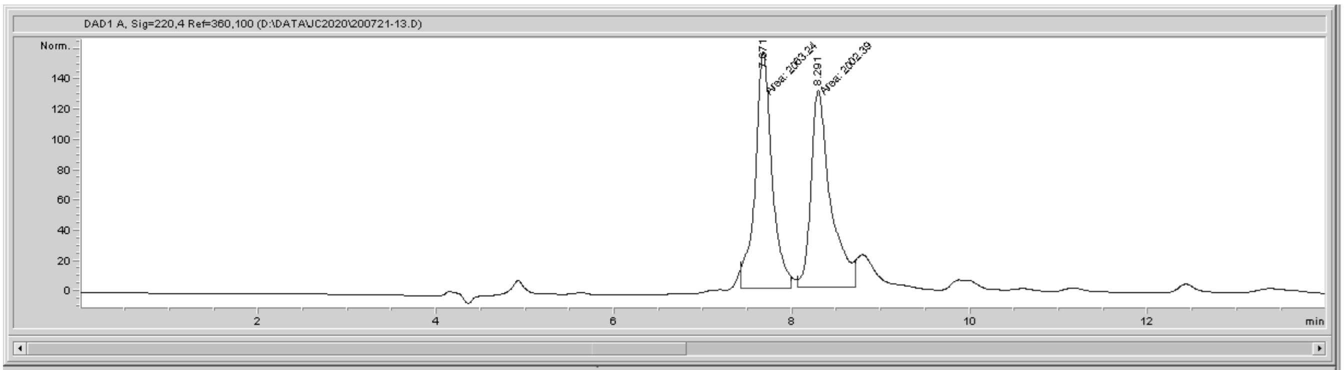

#	Time	Area	Height	Width	Area%	Symmetry
1	7.671	2063.2	157.2	0.2188	50.748	0.863
2	8.291	2002.4	131	0.2548	49.252	0.57

#	Time	Area	Height	Widht	Area%	Symmetry
**1**	7,671	2063,2	157,2	0,2188	**50,748**	0,863
**2**	8,291	2002,4	131	0,2548	**49,252**	0,57

2-((1*S*,2*S*,3*R*)-2-(2,4,5-trimethylphenyl)-3-(trifluoromethyl)cyclopropyl)-4,4,5,5-tetramethyl-1,3,2-dioxoborolane (2p)

Chiral HPLC using Chiralpak® IB N-3 [Heptane/MTBE (99:1), 0.8 mL/min]

Copper(I)-bisoxazoline L3 catalyzed reaction

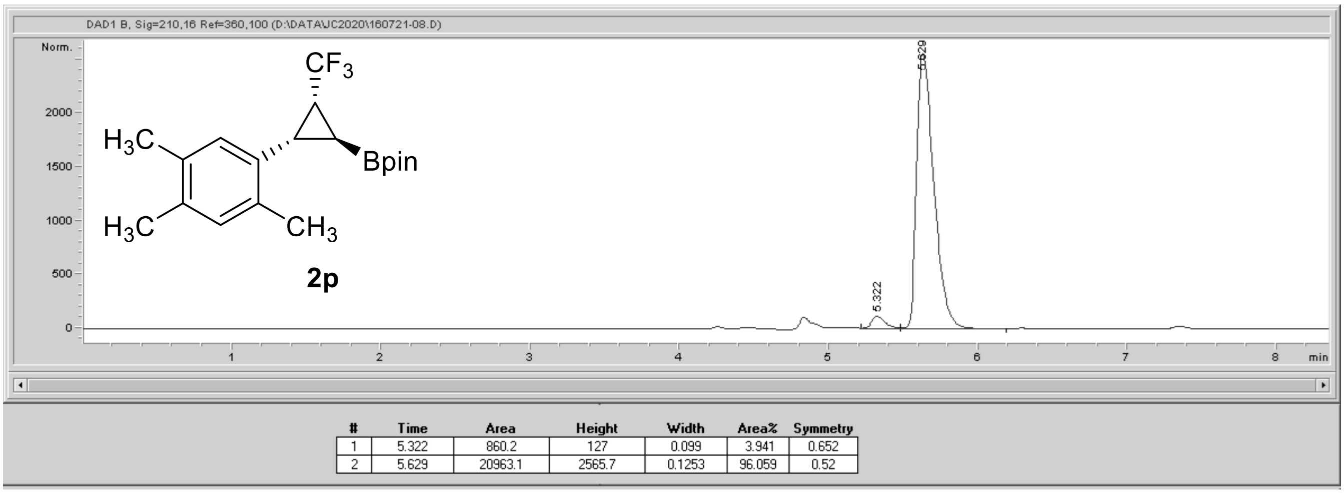

#	Time	Area	Height	Widht	Area%	Symmetry
**1**	5,322	860,2	127	0,099	**3,941**	0,652
**2**	5,629	20963,1	2565,7	0,1253	**96,059**	0,52

Pd(OAc)$_2$ catalyzed reaction

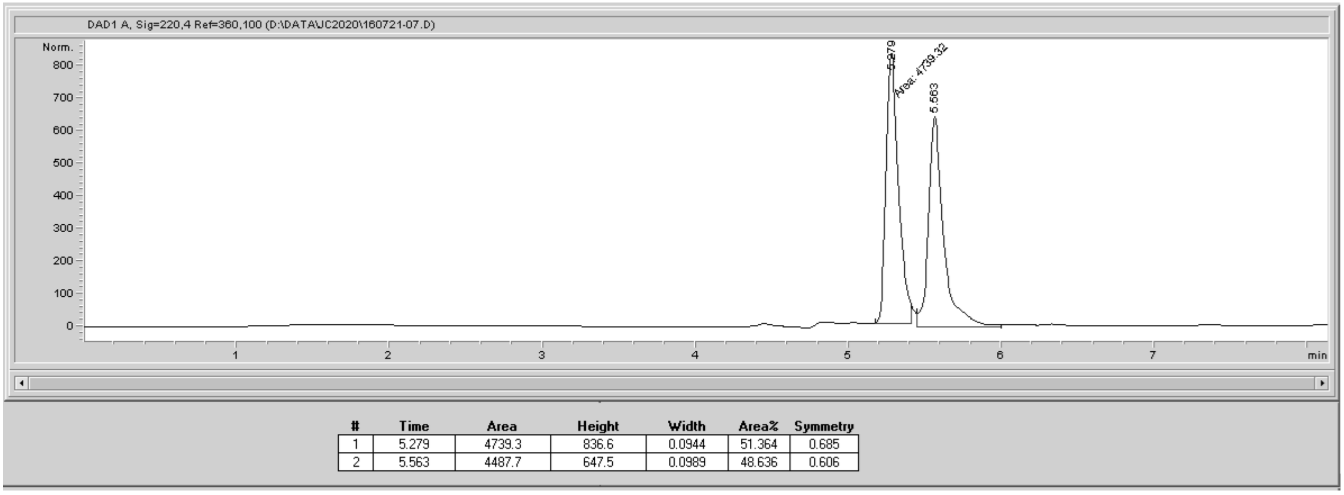

#	Time	Area	Height	Widht	Area%	Symmetry
**1**	5,279	4739,3	836,6	0,0944	**51,364**	0,685
**2**	5,563	4487,7	647,5	0,0989	**48,636**	0,606

2-((1*S*,2*S*,3*R*)-2-(Thiophen-3-yl)-3-(trifluoromethyl)cyclopropyl)-4,4,5,5-tetramethyl-1,3,2-dioxoborolane (2q)

Chiral HPLC using Chiralpak® IB N-3 [Heptane/MTBE (98:2), 1 mL/min]

Copper(I)-bisoxazoline L3 catalyzed reaction

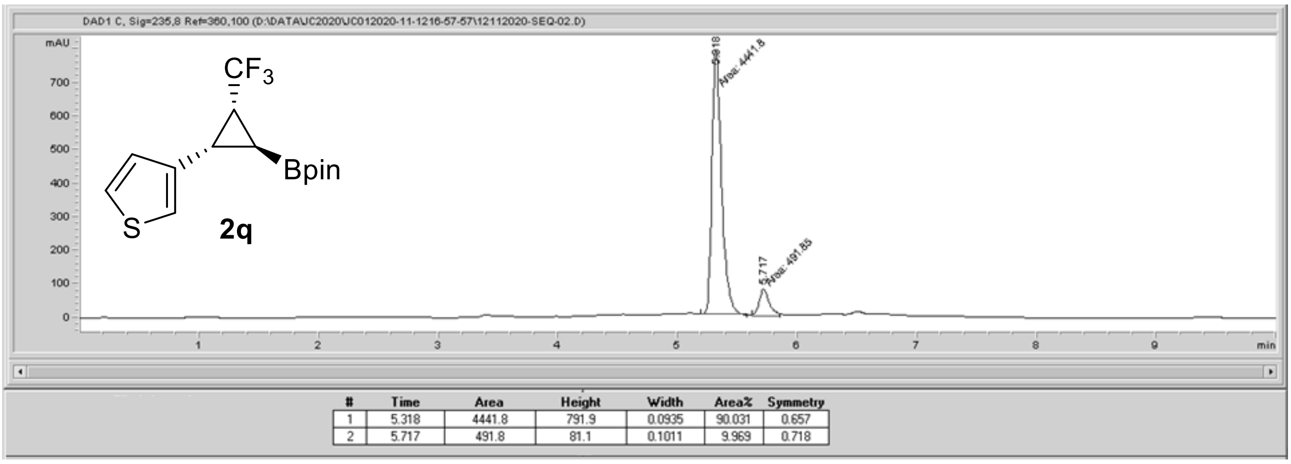

#	Time	Area	Height	Widht	Area%	Symmetry
**1**	5,318	4441,8	791,9	0,0935	**90,031**	0,657
**2**	5,717	491,8	81,1	0,1011	**9,969**	0,718

Pd(OAc)$_2$ catalyzed reaction

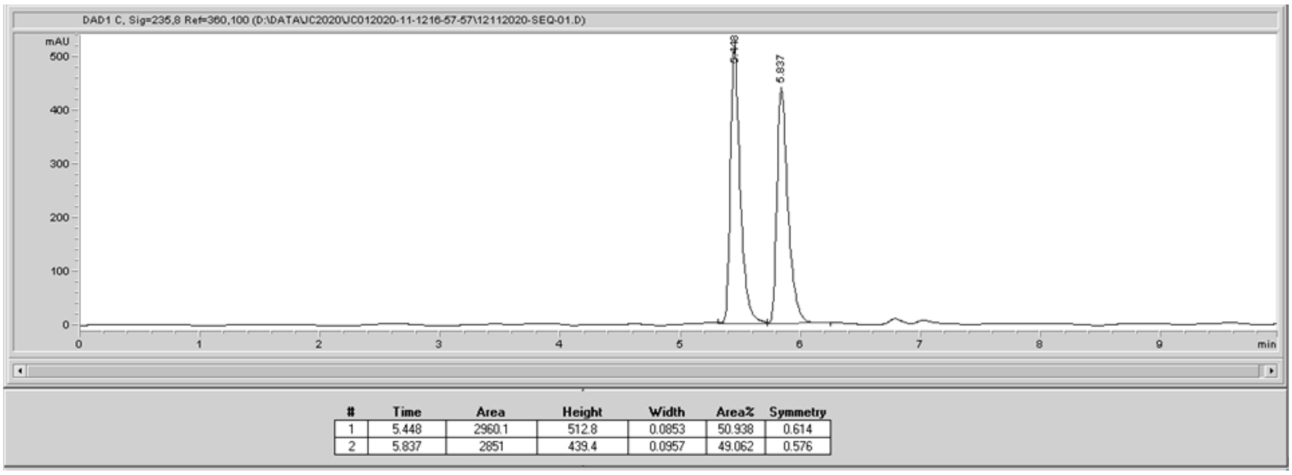

#	Time	Area	Height	Widht	Area%	Symmetry
**1**	5,448	2960,1	512,8	0,0853	**50,938**	0,614
**2**	5,837	2851	439,4	0,0957	**49,062**	0,576

2-((1*S*,2*S*,3*R*)-2-Phenethyl-3-(trifluoromethyl)cyclopropyl)-4,4,5,5-tetramethyl-1,3,2-dioxoborolane (2r)

Chiral HPLC using Chiralpak® IB N-3 [Heptane/MTBE (99:1), 1 mL/min]

Copper(I)-bisoxazoline L3 catalyzed reaction

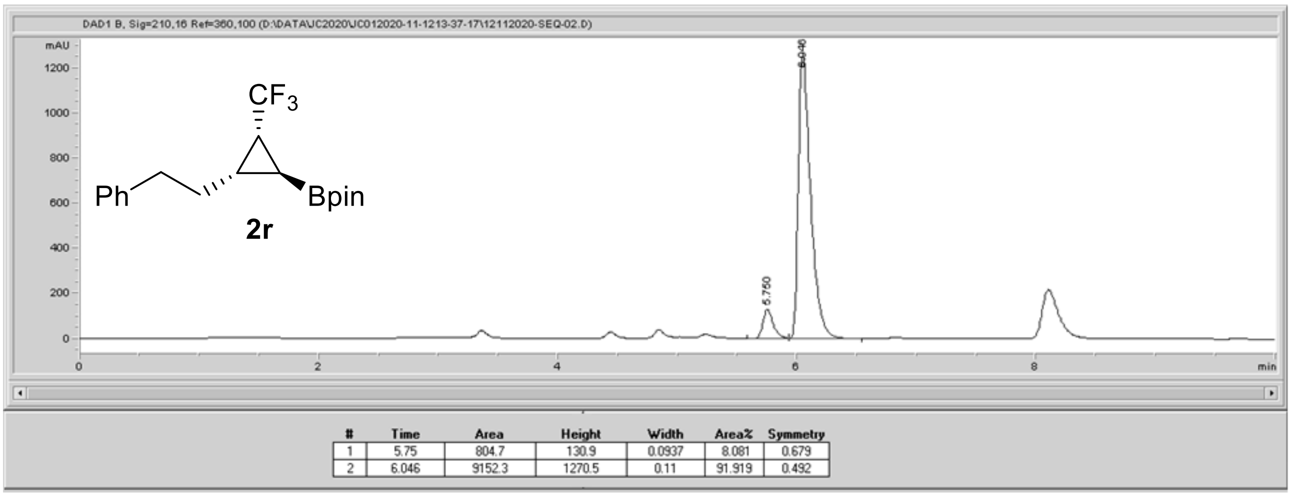

#	Time	Area	Height	Widht	Area%	Symmetry
**1**	5,75	804,7	130,9	0,0937	**8,081**	0,679
**2**	6,046	9152,3	1270,5	0,11	**91,919**	0,492

Pd(OAc)$_2$ catalyzed reaction

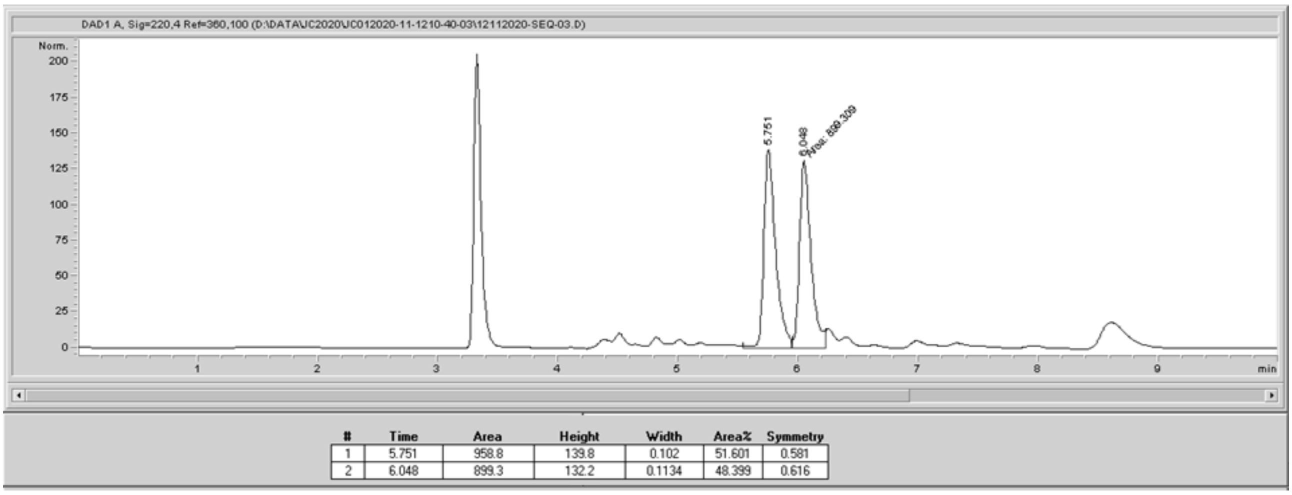

#	Time	Area	Height	Widht	Area%	Symmetry
**1**	5,751	958,8	139,8	0,102	**51,601**	0,581
**2**	6,048	899,3	132,2	0,1134	**48,399**	0,616

X-ray crystallographic data

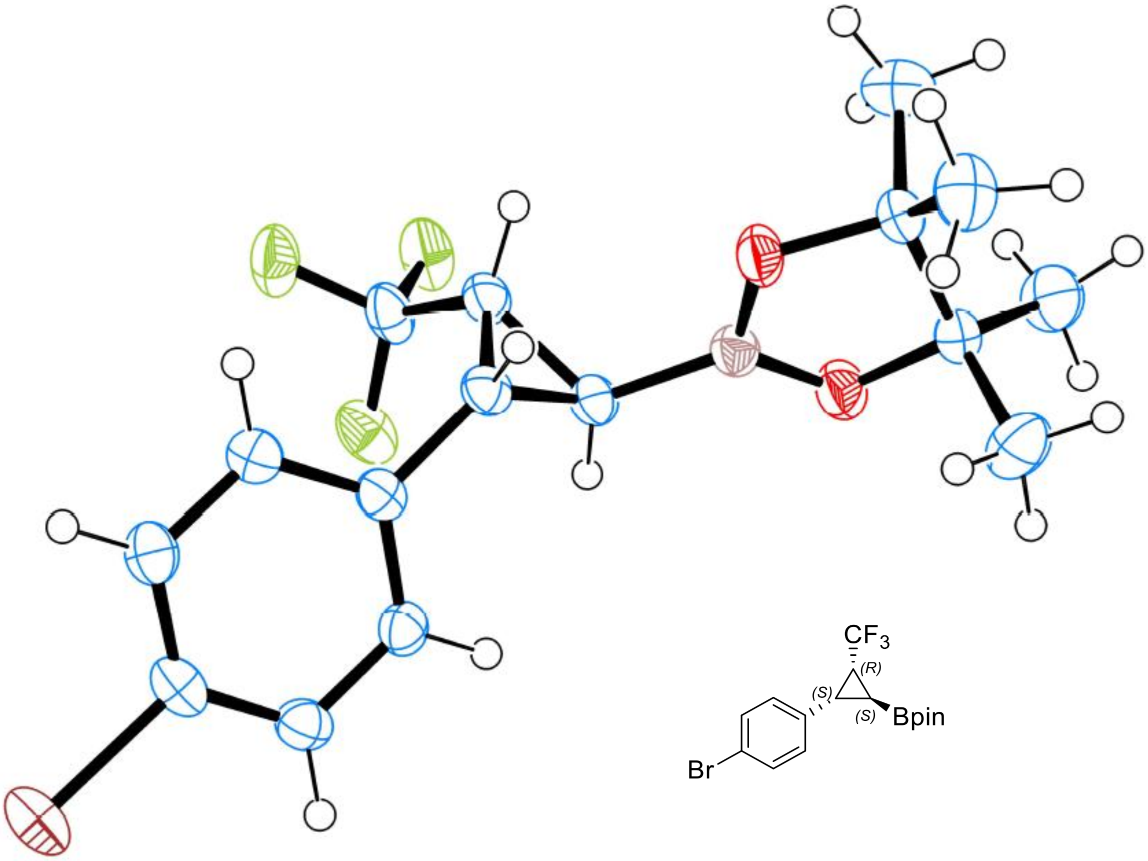

Thermal ellipsoids are drawn at the 50% probability level.

Table S2. Crystal data and structure refinement for **2i**.

CCDC number	2079481	Crystal colour	colourless
Empirical formula	$C_{16}H_{19}BBrF_3O_2$	Crystal shape	block
Formula weight	391.03	Radiation	Mo (λ=0.71073 Å)
Temperature [K]	100(2)	2Θ range [°]	3.79 to 59.26 (0.72 Å)
Crystal system	monoclinic	Index ranges	-14 ≤ h ≤ 14<br>-40 ≤ k ≤ 40<br>-15 ≤ l ≤ 15
Space group (number)	$P2_1$ (4)		
a [Å]	10.7745(6)	Reflections collected	322815
b [Å]	28.8838(18)	Independent reflections	19562<br>R_{int} = 0.0269<br>R_{sigma} = 0.0122
c [Å]	11.2111(8)		
α [°]	90	Completeness to Θ = 25.242°	100.0 %
β [°]	93.997(2)	Data / Restraints / Parameters	19562/1/845
γ [°]	90	Goodness-of-fit on F^2	1.024
Volume [Å³]	3480.5(4)		
Z	8	Final *R* indexes [$I \geq 2\sigma(I)$]	R_1 = 0.0289<br>wR_2 = 0.0731
ρ_{calc} [gcm^{-3}]	1.492	Final *R* indexes [all data]	R_1 = 0.0312<br>wR_2 = 0.0751
μ [mm^{-1}]	2.396	Largest peak/hole [eÅ^{-3}]	1.16/-0.63
F(000)	1584	Flack X parameter	-0.0013(8)
Crystal size [mm³]	0.287×0.257×0.118		

Single crystals of **2h** were obtained from MeOH by slow evaporation. The data for **2h** were collected from a shock-cooled single crystal at 100(2) K on a Bruker D8 VENTURE dual wavelength Mo/Ag four-circle diffractometer with a microfocus sealed X-ray tube using mirror optics as monochromator and a Bruker PHOTON III detector. The diffractometer was equipped with an Oxford Cryostream 800 low temperature device and used Mo radiation (λ = 0.71073 Å). All data were integrated with SAINT and a none absorption correction using SADABS was applied.[S4,S5] The structure were solved by dual methods using SHELXS-97 and refined by full-matrix least-squares methods against F2 by SHELXL-2014.[S6,S7] All non-hydrogen atoms were refined with anisotropic displacement parameters. The hydrogen atoms were refined isotropically on calculated positions using a riding model with their U_{iso} values constrained to 1.5 times the U_{eq} of their pivot atoms for terminal sp^3 carbon atoms and 1.2 times for all other carbon atoms. Crystallographic data for the structures reported in this paper have been deposited with the Cambridge Crystallographic Data Centre.[S8] CCDC 2079481 contain the supplementary crystallographic data for this paper. These data can be obtained free of charge from The Cambridge Crystallographic Data Centre via www.ccdc.cam.ac.uk/structures. This report and the CIF file were generated using FinalCif.[S9]

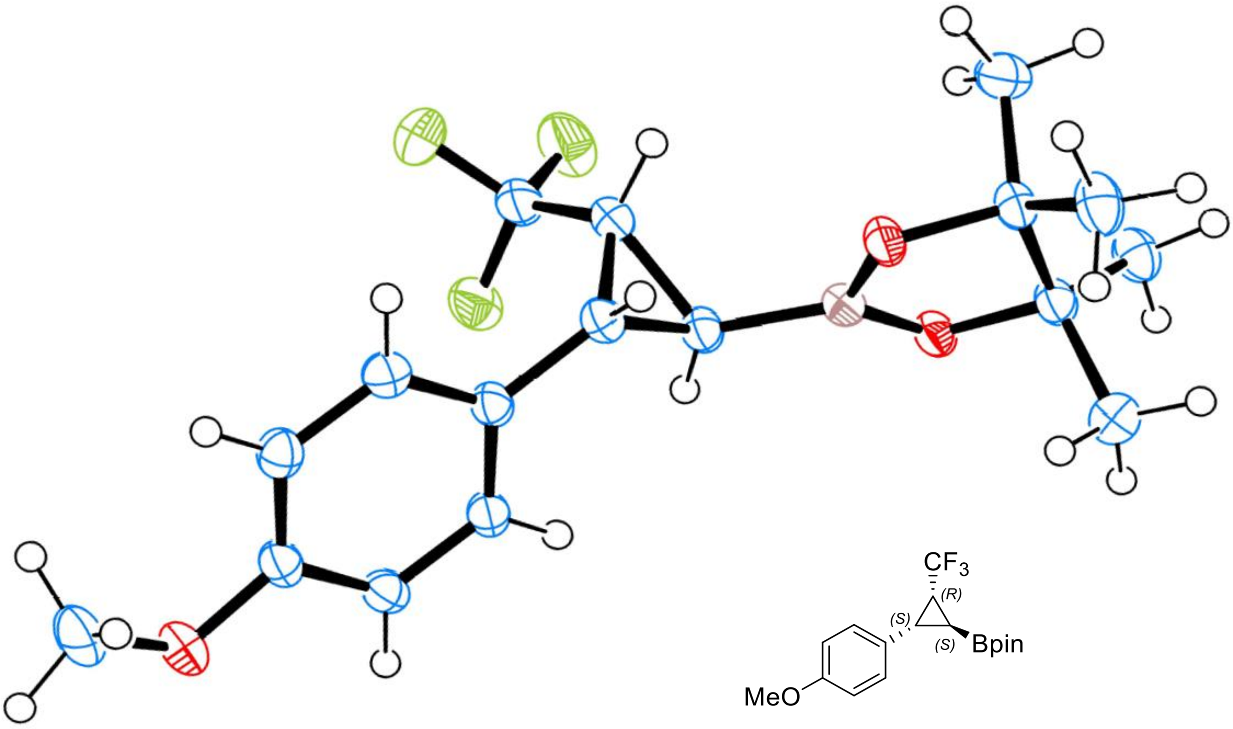

Thermal ellipsoids are drawn at the 50% probability level.

Table S3. Crystal data and structure refinement for **2l**.

CCDC number	2079480	Crystal colour	colourless
Empirical formula	$C_{17}H_{22}BF_3O_3$	Crystal shape	needle
Formula weight	342.15	Radiation	CuK_α (λ=1.54178 Å)
Temperature [K]	100.0	2Θ range [°]	6.78 to 159.92 (0.78 Å)
Crystal system	monoclinic	Index ranges	-25 ≤ h ≤ 25<br>-8 ≤ k ≤ 8<br>-20 ≤ l ≤ 20
Space group (number)	*C*2 (5)	Reflections collected	34534
a [Å]	20.3033(10)	Independent reflections	3820<br>R_{int} = 0.0268<br>R_{sigma} = 0.0129
b [Å]	6.7940(3)	Completeness to Θ = 67.679°	99.9 %
c [Å]	16.0858(8)	Data / Restraints / Parameters	3820/1/222
α [°]	90	Goodness-of-fit on F^2	1.071
β [°]	125.898(3)	Final *R* indexes [I≥2σ(I)]	R_1 = 0.0283<br>wR_2 = 0.0787
γ [°]	90	Final *R* indexes [all data]	R_1 = 0.0284<br>wR_2 = 0.0787
Volume [Å^3]	1797.43(16)	Largest peak/hole [eÅ^{-3}]	0.22/-0.16
Z	4	Flack X parameter	-0.05(3)
ρ_{calc} [gcm^{-3}]	1.264		
μ [mm^{-1}]	0.888		
F(000)	720		
Crystal size [mm^3]	0.999×0.122×0.078		

Single crystals of **2l** were obtained from MeOH by slow evaporation. The data for **2l** were collected from a shock-cooled single crystal at 100.0 K on a Bruker D8 VENTURE dual wavelength Mo/Cu four-circle diffractometer with a microfocus sealed X-ray tube using mirror optics as monochromator and a Bruker PHOTON III detector. The diffractometer was equipped with an Oxford Cryostream 800 low temperature device and used CuKα radiation (λ = 1.54178 Å). All data were integrated with SAINT and a multi-scan absorption correction using SADABS was applied.[S4,S5] The structure were solved by dual methods using XT and refined by full-matrix least-squares methods against F2 by SHELXL-2014.[S6,S7] All non-hydrogen atoms were refined with anisotropic displacement parameters. The hydrogen atoms were refined isotropically on calculated positions using a riding model with their U_{iso} values constrained to 1.5 times the U_{eq} of their pivot atoms for terminal sp^3 carbon atoms and 1.2 times for all other carbon atoms. Crystallographic data for the structures reported in this paper have been deposited with the Cambridge Crystallographic Data Centre.[S8] CCDC 2079480 contain the supplementary crystallographic data for this paper. These data can be obtained free of charge from The Cambridge Crystallographic Data Centre via www.ccdc.cam.ac.uk/structures. This report and the CIF file were generated using FinalCif.[S9]

[S4] Bruker, *SAINT*, *SAINT*, Bruker AXS Inc., Madison, Wisconsin, USA.
[S5] L. Krause, R. Herbst-Irmer, G. M. Sheldrick, D. Stalke, *J. Appl. Cryst.* **2015**, *48*, 3-10.
[S6] G. M. Sheldrick, *Acta Cryst.* **2008**, *A64*, 112–122.
[S7] G. M. Sheldrick, *Acta Cryst.* **2015**, *C71*, 3–8.
[S8] C. R. Groom, I. J. Bruno, M. P. Lightfoot, S. C. Ward, *Acta Cryst.* **2016**, *B72*, 171–179.
[S9] D. Kratzert, *FinalCif*, *V84*, https://www.xs3.uni-freiburg.de/research/finalcif
